# Changes in adolescents’ daily-life solitary experiences during the COVID-19 pandemic: an experience sampling study

**DOI:** 10.1186/s12889-024-18458-1

**Published:** 2024-04-26

**Authors:** Eva Bamps, Robin Achterhof, Ginette Lafit, Ana Teixeira, Zeynep Akcaoglu, Noëmi Hagemann, Karlijn S. F. M. Hermans, Anu P. Hiekkaranta, Julie J. Janssens, Aleksandra Lecei, Inez Myin-Germeys, Olivia J. Kirtley

**Affiliations:** 1 Center for Contextual Psychiatry, Research Group Psychiatry, Department of Neurosciences, KU Leuven, Herestraat 49– bus 1029, 3000 Leuven, Belgium; 2 KU Leuven Child & Youth Institute, KU Leuven, Leuven, Belgium; 3 Erasmus School of Social and Behavioural Sciences, Department of Psychology, Education & Child Studies, Erasmus University Rotterdam, Mandeville Building Room T15-10, 3000 DR Rotterdam, P.O. Box 1738, The Netherlands; 4 Research Group on Quantitative Psychology and Inaffiliationidual Differences, Faculty of Psychology, KU Leuven, Tiensestraat 102– bus 3713, 3000 Leuven, Belgium; 5 Research Group Adapted Physical Activity and Psychomotor Rehabilitation, Department of Rehabilitation Sciences, KU Leuven, Tervuursevest 101– bus 1500, 3000 Leuven, Belgium; 6 Strategy and Academic Affairs, Administration and Central Services, Leiden University, 2300 RA Leiden, PO Box 9500, The Netherlands; 7 Center for Clinical Psychiatry, Research Group Psychiatry, Department of Neurosciences, KU Leuven, Herestraat 49– bus 1029, 3000 Leuven, Belgium; 8 Leuven Brain Institute, KU Leuven, Leuven, Belgium

**Keywords:** Solitude, COVID-19, Adolescence, Daily life, Longitudinal, Experience sampling

## Abstract

**Background:**

Adolescent solitude was drastically impacted by the COVID-19 pandemic. As solitude is crucial for adolescent development through its association with both positive and negative developmental outcomes, it is critical to understand how adolescents’ daily-life solitary experiences changed as a result of the pandemic.

**Methods:**

Using three waves of Experience Sampling Method data from a longitudinal study, we compared adolescents’ daily-life solitary experiences in the early (*n*_*T1*_=100; *M*_*Age*_=16.1; *SD*_*Age*_=1.9; 93% girls) and mid-pandemic (*n*_*T2*_=204; *M*_*Age*_=16.5; *SD*_*Age*_=2.0; 79% girls) to their pre-pandemic experiences.

**Results:**

We found that adolescents with lower levels of pre-pandemic social support and social skills reported wanting to be alone less and feeling like an outsider more at both time points during the pandemic. In the mid-pandemic wave, adolescents with higher levels of pre-pandemic social support and social skills reported decreases in positive affect compared to the pre-pandemic wave.

**Conclusion:**

This study shows that adolescents’ daily-life solitary experiences worsened throughout the COVID-19 pandemic. There should be continued concern for the wellbeing of all adolescents, not only those already at risk, as effects of the pandemic on mental health might only manifest later.

**Supplementary Information:**

The online version contains supplementary material available at 10.1186/s12889-024-18458-1.

The COVID-19 pandemic and the associated governmental measures were a major disruption to adolescents’ development in general and to their social development specifically [1, 2]. Stay-at-home orders and social distancing were key measures taken against the spread of COVID-19. Therefore, one of the most drastically altered aspects of adolescents’ daily lives was the time they spent alone (i.e., in solitude in the definition of Larson [3]). Gaining insight into changes in solitude is critical for advancing our understanding of the impact of the pandemic on adolescents.

Increased solitude is part of normative adolescent development [3]. It is a necessary precondition to succeed at the developmental tasks of individuation and identity development, as spending time alone affords adolescents more freedom (e.g., over their activities), and allows them to reflect on themselves and on their future [3, 4]. However, it is also a risk factor for peer exclusion and victimization [5, 6], and the development of mental health problems [7, 8]. Given this association with both positive and negative developmental outcomes and because solitude was so drastically altered during the COVID-19 pandemic, it is critical to investigate how these *levels* of solitude changed during the COVID-19 pandemic and which adolescents were impacted most by these changes. Face-to-face interactions decreased during the COVID-19 lockdowns [2], but there is no information about how much time adolescents effectively spent alone. It is possible, for example, that adolescents spent a considerable amount of time following online classes in the same room where their parents were working remotely.

In addition to the amount of time adolescents spent alone, their *experiences* of solitude likely changed as well. Assessing these experiences (including affect and appraisals of being alone) is crucial, as they are likely more predictive of mental health outcomes than mere amounts of time spent alone [4, 7]. Multiple studies reported an increase in loneliness in adolescents in the early stages of the COVID-19 pandemic [9, 10] across both social and solitary situations. However, it is not clear to what extent this increased loneliness is driven by increased levels of solitude. To assess this, we need to examine loneliness specifically in solitary situations. In addition, there is currently no knowledge on changes in other solitary experiences, such as how pleasant adolescents found it to be alone or how much they wanted to be alone.

To accurately gauge the impact of the COVID-19 pandemic on adolescents’ solitary experiences, it is necessary to assess these experiences before and during the pandemic. In addition, investigating these experiences in daily life provides a unique perspective on adolescents’ altered social lives. To this end, the current study used longitudinal data collected with the Experience Sampling Method (ESM) [11, 12]. With ESM, data are collected in the flow of daily life by prompting participants multiple times per day to report on their current affect and social context [12]. Therefore, it can provide ecologically valid information about how adolescents experience solitude in their day-to-day life [12]. Here, ESM data were used from SIGMA, a large-scale longitudinal study with adolescents from Flanders, the Dutch-speaking part of Belgium [13]. This allowed us to compare pre-pandemic (2018–2019, T0) solitary experiences to solitary experiences in the early (2020, T1) and mid-pandemic (2021, T2) in daily life, which adds to the existing literature on the effects of the COVID-19 pandemic that has, so far, primarily focussed on the reduction of social contact during the pandemic [2].

A recent study using SIGMA data showed that before the COVID-19 pandemic, adolescents differed in their daily-life solitary experiences [14]. One group of adolescents reported primarily positive solitary experiences, including high levels of positive affect, while the other group reported primarily negative solitary experiences, including high levels of negative affect, loneliness and feeling like an outsider [14]. Therefore, it is expected that adolescents will have been impacted differently by the COVID-19 pandemic depending on their pre-pandemic solitary experiences. Other personal factors that are hypothesized to influence solitary experience include COVID-19-related stressors [15, 16], social support [16, 17] and social skills [17, 18]. Assessing the differential impact of COVID-19 on adolescents’ solitary experiences allows for the identification of adolescents that might be at increased risk of negative developmental outcomes.

In this study, we first assessed changes in the time adolescents spent alone and in their daily-life solitary experiences from pre-pandemic to the early and mid-pandemic. Secondly, we identified personal factors that moderated these changes to identify the adolescents most vulnerable for the negative impact of increased solitude due to COVID-19. A detailed overview of the specific hypotheses tested in this study, can be found in the post-registrations [19, 20]. The results of this study will give us important information on changes in a crucial aspect of adolescents’ daily lives that has been understudied in the context of the COVID-19 pandemic and they can help us gain insight into what factors exacerbate or mitigate these changes.

## Methods

### Sample

The sample included in the current study is a part of the SIGMA project [13], a large-scale study with a planned accelerated longitudinal design [21] and two follow-up periods in Flanders, the Dutch-speaking part of Belgium. The inclusion criteria for the initial measurement were (a) attending a school that participated in the SIGMA project, (b) being enrolled in the first, third, or fifth year of secondary school, and (c) having adequate command of the Dutch language. For the follow-up measurements, only participants who took part in the initial measurement were included.

The SIGMA project was approved by the Ethics Committee Research UZ/KU Leuven (S61395). Informed assent was obtained from underage participants, and informed consent was obtained from their parent or caregiver. Participants aged > 18 gave informed consent themselves.

The pre-pandemic (T0) sample of *n* = 1913 (63% girls) aged 11–20 (*M* = 13.8, *SD* = 1.9) was recruited between January 2018 and June 2019.

Early-pandemic (April 2020; T1), all SIGMA participants for whom we had valid contact data (*n* = 1758) were invited for a special COVID-follow-up of the SIGMA project. This resulted in a follow-up sample of *n* = 173 (89% girls; *M*_*Age*_=16.0, *SD*_*Age*_=1.9, *Min-Max*_*Age*_=13–20), *n* = 110 of whom also provided ESM data. All participants were assessed remotely in the week of May 4th 2020. At this time point, the governmental measures to combat the COVID-19 pandemic in Flanders were at the strictest level [22, 23]. Schools were closed, leaving the house was only permitted for essential activities (i.e., grocery shopping, exercise), and all public events were cancelled [22, 23].

The mid-pandemic (T2) sample (*n* = 277; 74% girls; *M*_*Age*_=16.5, *SD*_*Age*_=2.0, *Min-Max*_*Age*_=14–21) was followed up between January and June 2021 as part of the originally planned two-year follow-up for the SIGMA project. ESM data were provided by *n* = 231. The participants were assessed either in school or remotely under varying levels of restrictive measures [22, 23]. In January 2021, schools were open, but stay-at-home orders were still in place, and public events were still prohibited [22, 23]. The strictness of the governmental measures briefly increased in April 2021, when schools closed again, to decrease again in May and June 2021, when schools reopened and leaving the house for non-essential activities was permitted [22, 23].

#### Moment selection and final sample

In the analyses, we only considered moments when participants were alone (as indicated by the response *nobody* to the multiple-choice ESM item *Who is with me?*). Participants who were never alone were excluded. At T0, this resulted in final samples of *n* = 97 and *n* = 184 for comparison with T1 and T2, respectively. At T1, this resulted in a final sample of *n* = 100 and at T2, this resulted in a final sample of *n* = 204. Descriptive statistics for all final samples can be found in Table 1 (T0–T1) and Table 2 (T0–T2).

**Table 1:** Descriptive statistics of the T0 and T1 samples

Variable	T0					T1				
	*n*	*M*	*SD*	Median	Min - Max	*n*	*M*	*SD*	Median	Min - Max
Age	97	14.3	1.8	14.0	11.0–18.0	100	16.1	1.9	16.0	13.0–20.0
Gender (%female)	97	91.8%				100	93.0%			
Compliance^a^	97	0.6	0.2	0.6	0.2–1	100	0.5	0.3	0.5	0.03–1
Positive Affect	97	5.0	1.2	5.0	1.0–7.0	100	5.0	1.1	5.0	1.0–7.0
Negative Affect	97	2.1	1.0	1.9	1.0–6.2	100	2.2	1.2	1.7	1.0–7.0
Loneliness	97	2.1	1.4	1.6	1.0–7.0	100	2.1	1.4	1.4	1.0–7.0
Finding it Pleasant to be Alone	97	5.3	1.6	5.5	1.0–7.0	100	5.2	1.3	5.4	1.0–7.0
Feeling Like an Outsider	97	1.4	1.0	1.0	1.0–7.0	100	1.4	0.8	1.0	1.0–4.5
Wanting to be Alone	97	4.6	1.8	5.0	1.0–7.0	100	4.8	1.4	5.0	1.0–7.0
Proportion of Time Spent Alone	97	0.2	0.1	0.2	0.02–0.7	100	0.4	0.2	0.4	0.02–1
Social Support (T0)	93	22.2	6.4	21.0	5.0–36.0					
Social Skills	90	69.3	7.8	69.0	54.0–90.0					
Solitude Cluster Membership (% positive cluster)	97	74.2%								
Number of COVID-Related Stressors						100	10.3	2.6	11.0	5.0–16.0
Mean Burdensomeness of COVID-Related Stressors						100	3.2	0.5	3.2	2.1–4.7
Social Support (T1)						99	58.7	14.8	60.0	19.0–84.0

^a^ The compliance represents the proportion of non-missing momentary questionnaires.

**Table 2:** Descriptive statistics of the T0 and T2 samples

Variable	T0					T2				
	*n*	*M*	*SD*	Median	Min - Max	*n*	*M*	*SD*	Median	Min - Max
Age	183	13.9	1.8	14.0	11.0–19.0	204	16.5	2.0	16.0	14.0–21.0
Gender (%female)	184	75.5%				204	78.9%			
Compliance^a^	184	0.6	0.2	0.6	0.1–1	204	0.4	0.2	0.4	0.02–1
Positive Affect	184	5.3	1.1	5.4	1.5–7.0	204	4.6	1.1	4.6	1.3–7.0
Negative Affect	184	1.9	1.0	1.6	1.0–7.0	204	2.3	1.0	2.0	1.0–5.9
Loneliness	184	2.0	1.4	1.2	1.0–7.0	204	2.3	1.4	1.9	1.0–7.0
Finding it Pleasant to be Alone	184	5.2	1.6	5.5	1.0–7.0	204	4.9	1.5	5.0	1.0–7.0
Feeling Like an Outsider	184	1.4	0.8	1.0	1.0–7.0	204	1.5	0.9	1.1	1.0–7.0
Wanting to be Alone	184	4.6	1.7	4.7	1.0–7.0	204	4.4	1.5	4.5	1.0–7.0
Proportion of Time Spent Alone	184	0.2	0.1	0.1	0.02–0.7	204	0.4	0.2	0.4	0.03–1
Social Support (T0)	178	23.3	5.6	23.0	5.0–35.0					
Social Skills	169	70.0	7.0	69.9	54.0–90.0	191	68.6	8.1	68.0	43.0–89.0
Solitude Cluster Membership (% positive cluster)	184	76.1%								
Number of COVID-Related Stressors						195	10.7	3.2	11.0	1.0–17.0
Mean Burdensomeness of COVID-Related Stressors						194	3.2	0.6	3.3	1.0–5.0
Social Support (T2)						191	65.7	14.1	70.0	22.0–84.0

^a^ The compliance represents the proportion of non-missing momentary questionnaires.

The T1 and T2 samples only represent 5% and 11%, respectively, of the original T0 sample, which could have led to selection bias. Achterhof et al. [2] already investigated whether this was the case for the T1. They found that participants who identified as female, who were older, and who reported more psychopathology at T0 were more likely to re-enter the study at T1. We compared participants who did and did not re-enrol at T2 and found that participants with fewer missed momentary questionnaires at T0, those with more PA when alone, and those with better solitary experiences at T0 were more likely to re-enter the study at T2 [see Additional File 1].

### Procedure

#### T0 procedure

During an in-school session, the participants completed a questionnaire battery [13] on a tablet provided by the researchers. This session lasted for two class periods (100 min) and at the end, the participants received a study smartphone (Motorola Moto E4 with Android version 7.1.1) along with the instructions for the ESM period. The ESM period consisted of six consecutive days, starting the day after the in-school session, during which participants received 10 notifications per day with a prompt to fill out a short momentary questionnaire. The notifications occurred at semi-random time points within 90-minute time blocks between 7:30 AM and 10:30 PM, with a minimum of 15 min between two notifications. The ESM data were collected via the mobileQ application [24], and participants had 90 s to start the momentary questionnaire, and 90 s to complete each question, otherwise the questionnaire would time out automatically. The participants returned their study smartphones to the researchers at the end of the ESM period and subsequently received their reimbursement.

#### T1 procedure

Due to the restrictive measures in place at the time of T1 data collection, all data were collected online. Participants were sent the questionnaire battery through REDCap [25, 26]. On May 1st 2020, participants received instructions to download and set up the SEMA3 application [27] on their own smartphone for the ESM period. The same sampling scheme was used as at T0, with the exception that participants now had 10 min from the moment they received the notification to start and complete the questionnaire before it timed out automatically.

#### T2 procedure

The T2 data were collected in school or remotely, depending on the COVID-19 related measures in place at the time of data collection. If the questionnaire battery was completed in school, the procedure was identical to the procedure at T0. Otherwise, the procedure was identical to the procedure at T1. The ESM data were collected either via the study smartphones with the mobileQ application [24], or on participants’ own smartphones with the m-Path application [28]. Out of the 231 participants who provided ESM data mid-pandemic, 39 participants used mobileQ and 192 participants used m-Path. For both applications the same signal-contingent, semi-random sampling scheme as at T0 and T1 was used. Participants who used their own smartphone had 15 min to initiate the questionnaire after the notification. Once initiated, the questionnaire did not time out automatically.

### Measures

The correlations between all variables measured at the different time points are available in an additional file [see Additional File 2].

#### Independent variables

##### Number of COVID-Related Stressors and Mean Burdensomeness of COVID-Related Stressors (T1 and T2)

We used a measure developed for the DynaCORE survey on resilience during the early stages of the COVID-19 pandemic (part of the DynaMORE project) [29] to assess the total number of COVID-related stressors reported by participants and the mean burdensomeness of these events. We adapted the original DynaCORE stressors measure to make it more suitable for use in an adolescent sample. Specifically, this meant that we removed items that we did not consider relevant for our study population (e.g., difficulties combining work with childcare, business travel not possible). The adapted questionnaire used at T1 and T2 consisted of 22 items (out of the original 29 items) [29] assessing stressors that might have arisen due to the COVID-19 pandemic. Examples include contracting COVID-19, not being able to see a lot of people, parental conflict and financial stress. At T1 and T2, participants were asked to indicate whether the event had happened to them in the previous two weeks on a binary response scale (0 being *This event has not happened*, 1 being *This event has happened*). An “number of COVID-related stressors” score was calculated separately for T1 (*α* = 0.49, *ω* = 0.53) and T2 (*α* = 0.66, *ω* = 0.67) by summing all items that participants had endorsed. If participants endorsed an item, they were presented with a conditional item assessing the burdensomeness of the event. They were asked to what extent the event in question had troubled them and rated this on a 5-point Likert scale (0 being *No burden at all* and 4 being *Very burdensome*). Subsequently, the mean burdensomeness of COVID-related events was calculated separately for T1 and T2 by taking the mean of all burdensomeness scores per participant.

##### Social Support (T0)

At T0, the social support score was calculated as the sum score of all 12 items on the Sociale Steun Lijst-Interacties (Social Support List-Interactions; SSL-I-12) [30]. This Dutch-language questionnaire has been validated in an older sample and is deemed to also be useful in an adolescent population by the developers of the questionnaire [31]. The questionnaire consists of three subscales: Everyday Support (e.g. *How often does it happen that people show interest in you?*), Support With Problems (e.g. *How often does it happen that someone gives you advice?*), and Appreciation (e.g. *How often does it happen that someone compliments you?*). All items are scored on a scale of 1–4, with 1 being *Rarely or never* and 4 being *Very often*. The reliability of the social support score was good, both when using the smaller T0 sample for comparison to the T1 sample (*α* = 0.86, *ω* = 0.89) and when using the larger T0 sample for comparison to the T2 sample (*α* = 0.82, *ω* = 0.85).

##### Social Support (T1 and T2)

At T1 and T2, the Multidimensional Scale of Perceived Social Support (MSPSS) [32] replaced the SSL as the social support measure, because it was better suited for the study population. This questionnaire has been validated in an adolescent sample [33] and in Dutch [34]. The MSPSS consists of 12 items across three subscales: Significant Others (e.g. *There is a special person with whom I can share my happiness and my worries*), Family (e.g. *My family gives me the emotional support I need*) and Friends (e.g. *I can count on my friends when things go wrong*). At T1, participants were asked to report on the period since the beginning of the COVID-19 measures. At T2, there was no specific timeframe mentioned in the instructions. All items were scored on a 7-point Likert scale (1 being *Completely disagree* and 7 being *Completely agree*). The social support score was calculated separately for T1 (*α* = 0.87, *ω* = 0.95) and T2 (*α* = 0.90, *ω* = 0.97) by taking the sum of all 12 items.

##### Social Skills (T0)

Social skills were measured as the sum score on the Interpersonal Skills subscale of the Vragenlijst Persoonlijke Vaardigheden (Personal Skills Questionnaire; VPV), which is a Dutch-language questionnaire that has been validated for use in an adolescent sample [35]. The Interpersonal Skills subscale consists of 18 items across two subscales: Relational Skills (e.g. *I have no trouble interacting with others*), and Affective Skills (e.g. *I understand when I said something hurtful*). All items are scored on a 5-point Likert scale (1 being *Completely disagree* and 5 being *Completely agree*). The reliability of the social skills score was good, both when using the smaller T0 sample for comparison to the T1 sample (*α* = 0.83, *ω* = 0.87) and when using the larger T0 sample for comparison to the T2 sample (*α* = 0.80, *ω* = 0.85).

##### Pre-Pandemic Daily-Life Solitary Experiences (T0)

Participants’ pre-pandemic daily-life solitary experiences, hereafter referred to as solitude cluster membership, was based on the clusters identified in a previous study [14]. This cluster membership is an indication of how participants generally experienced their time alone in daily life at T0. In our previous study, we identified a group characterized by high levels of positive affect (the positive solitude cluster) and a group characterized by high levels of negative affect, loneliness and feeling like an outsider (the negative solitude cluster) [14]. The variable was coded as follows: 0 for participants in the positive solitude cluster at T0 and 1 for participants in the negative solitude cluster at T0.

#### Dependent variables

All affective and experience items were scored on a 7-point Likert scale (From 1 *Not at all* to 7 *Very much*).

##### Amount of Time Spent Alone

The person-level amount of time spent alone was calculated at each wave as the proportion of all completed momentary questionnaires in which a participant indicated to be alone.

##### Momentary Positive Affect, Negative Affect and Loneliness

Momentary positive affect (PA) was measured as the mean of the ESM items *I feel cheerful*, *I feel satisfied*, *I feel relaxed*, and *In general, I feel well at the moment*. Reliability was high at T0 (*ω*_withinT1_ = 0.77, *ω*_betweenT1_ = 0.95, *ω*_withinT2_ = 0.78, *ω*_betweenT2_ = 0.94), T1 (*ω*_within_ = 0.82, *ω*_between_ = 0.97) and T2 (*ω*_within_ = 0.80, *ω*_between_ = 0.95).

Momentary negative affect (NA) was measured as the mean of the ESM items *I feel irritated*, *I feel anxious*, *I feel insecure*, *I feel paranoid*, *I feel sad*, *I feel stressed*, and *I feel restless*. Reliability was high at T0 (*ω*_withinT1_ = 0.73, *ω*_betweenT1_ = 0.93, *ω*_withinT2_ = 0.70, *ω*_betweenT2_ = 0.91), T1 (*ω*_within_ = 0.78, *ω*_between_ = 0.97) and T2 (*ω*_within_ = 0.77, *ω*_between_ = 0.92).

Momentary loneliness was measured with the ESM item *I feel lonely*.

##### Momentary Finding it Pleasant to be Alone, Wanting to be Alone and Feeling Like an Outsider

At T0, T1 and T2, solitary experiences were measured with the ESM items *I find it pleasant to be alone*, *I want to be alone* and *I feel like an outsider*.

### Open science practices

This study was post-registered [19, 20] on the Open Science Framework (OSF) using the ESM registration template [36], which means that the hypotheses and data analysis plan were registered after data collection, but before data access [37]. Changes and additions to the post-registrations are detailed in an additional file [see Additional File 3]. The code for all analyses is available in an additional file [see Additional File 4] and on the OSF project page for this study [38].

### Statistical analyses

All analyses were carried out in R version 4.1.2 [39] using the packages *nlme* [40], *stats* [39], *mice* [41] and *mitml* [42].

#### Simulation-based power analyses

Simulation-based power analyses [43] for hypotheses H1a through H1g [19] and H3a through H3g [20] were conducted after data collection but before data was accessed. The simulation-based approach was chosen due to the absence of parameters in the literature on COVID-19 related changes in solitude. In order to obtain the power estimates for hypotheses H1a through H1g, data were used from 1167 participants who provided ESM data when alone at T0 but did not re-enter the study at T1. Similarly, data from 1026 participants who provided ESM data when alone at T0 but did not re-enter the study at T2 were used to obtain the power estimates for hypotheses H3a through H3g. For all hypotheses, data were simulated based on a range of plausible increases or decreases from T0 to T1 or T2 according to the direction of the hypothesis. For H1b through H1g and H3b through H3g, data were simulated for increases or decreases of 1%, 5%, 10%, 15% and 20%. For H1a and H3a, increases of 25%, 30%, 35%, 40%, 45% and 50% were added based on previous reports of sharp decreases in adolescent social interactions from T0 to T1 in the same sample [2]. For every effect size, 1000 datasets were simulated and analysed with linear regression models (H1a and H3a) or linear mixed-effects regression models (H1b through H1g and H3b through H3g). The proportion of datasets in which each null hypothesis was rejected at *α* < 0.007 (Bonferroni-corrected, initial *α* = 0.05) was taken as the power of a specific effect size. For H1a, there was sufficient power to detect increases of 40% and above. For H1b through H1g, there was sufficient power to detect increases or decreases of 5% and above. For H3A, there was sufficient power to detect increases of 30% and above. For H3b through H3g, there was sufficient power to detect in- or decreases of 5% and above.

#### Analysis strategy

A detailed description of the missing data imputation, and of all analyses is available in an additional file [see Additional File 5].

In brief, we first imputed missing data in the SSL, VPV, and MSPSS questionnaires using Multiple Imputation by Chained Equations (MICE). To test all hypotheses, we used either linear mixed-effects models with random intercepts or regular linear regression models. A binary variable timepoint was the predictor, with age and gender added as control variables. When examining the moderating effect of SSL- and VPV-score, an interaction between these variables and timepoint was added to the models. In the models examining the effect of the Number of COVID-Related Stressors, the Mean Burdensomeness of COVID-Related Stressors, and MSPSS-score, the dependent variables were converted into relative change scores. Detailed information on how this score was computed, is available in an additional file [see Additional File 3].

## Results

### Change in daily-life solitary experiences from pre-pandemic (T0) to early-pandemic (T1)

**Fig. 1:**
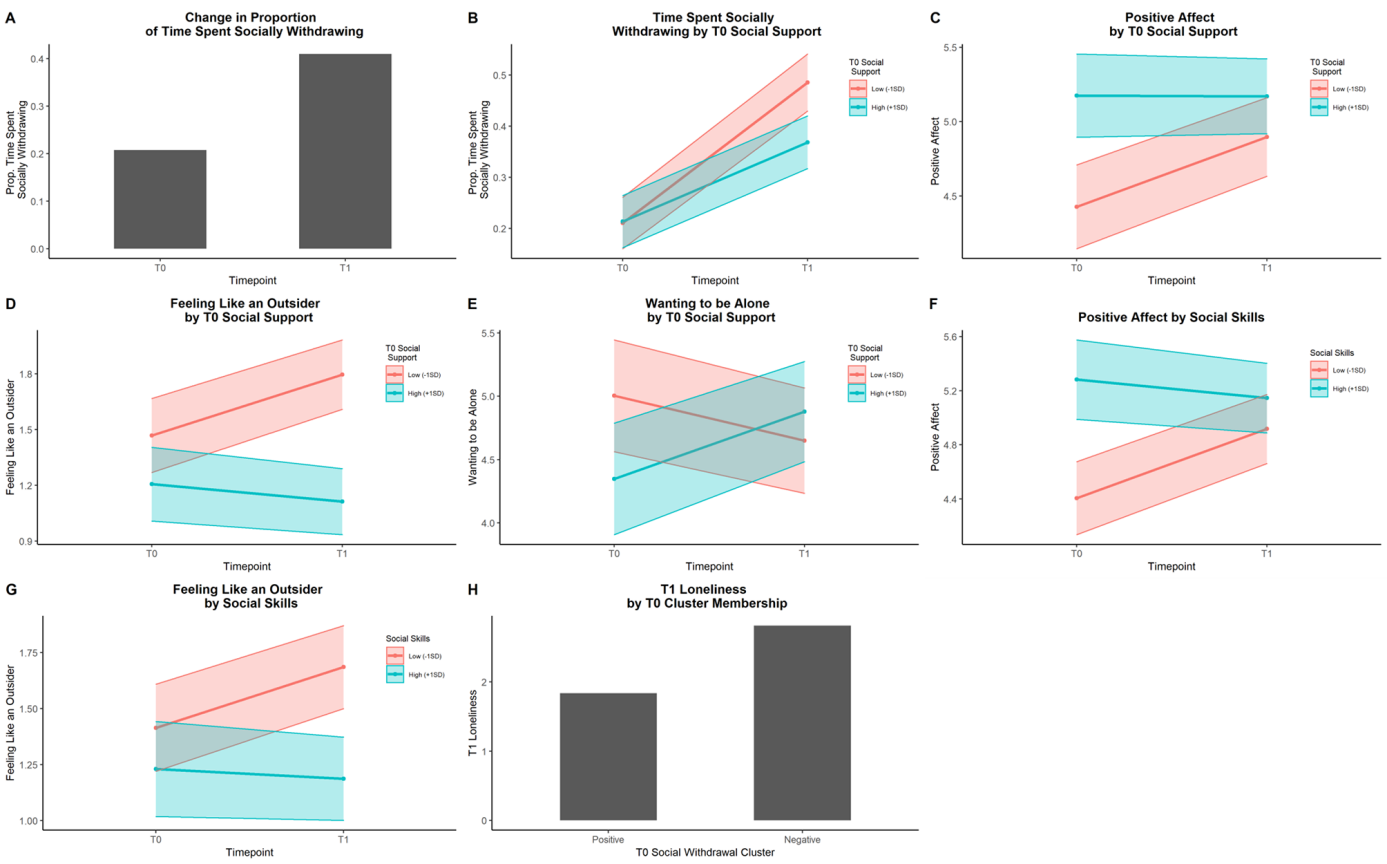
Change in Daily-Life Solitary experiences from T0 to T1 and moderators of change

The proportion of time spent alone increased significantly from T0 to T1 (*B*(*SE*) = 0.20 (0.03), *p* <.001; Fig. 1A). PA, NA, loneliness, and all three solitary experiences did not change significantly from T0 to T1.

### Moderators of change in daily-life solitary experiences from pre-pandemic (T0) to early-pandemic (T1)

There were several significant moderating effects of T0 social support. Less T0 social support was associated with steeper increases in the proportion of time spent alone (*B*(*SE*)=-0.01(0.00), *p* =.007; Fig. 1B), PA (*B*(*SE*)=-0.04(0.01), *p* <.001; Fig. 1C) and feeling like an outsider at T1 (*B*(*SE*)=-0.03(0.01), *p* <.001; Fig. 1D). Less T0 social support was also associated with a decrease in wanting to be alone at T1, while more T0 social support were associated with an increase in wanting to be alone at T1 (*B*(*SE*) = 0.07(0.02), *p* <.001; Fig. 1E).

There were also significant moderating effects of T0 social skills. Low levels of T0 social skills were associated with increases in PA (*B*(*SE*)=-0.04(0.01), *p* <.001; Fig. 1F) and feeling like an outsider (*B*(*SE*)=-0.02(0.01), *p* <.001; Fig. 1G), while high levels of T0 social skills were associated with decreases in both variables.

Solitude cluster membership was significantly associated with T1 loneliness (*B*(*SE*) = 0.97(0.32), *p* =.003; Fig. 1H). Participants in the negative solitude cluster reported more loneliness at T1 compared to participants in the positive solitude cluster.

### Change in daily-life solitary experiences from pre-pandemic (T0) to mid-pandemic (T2)

**Fig. 2:**
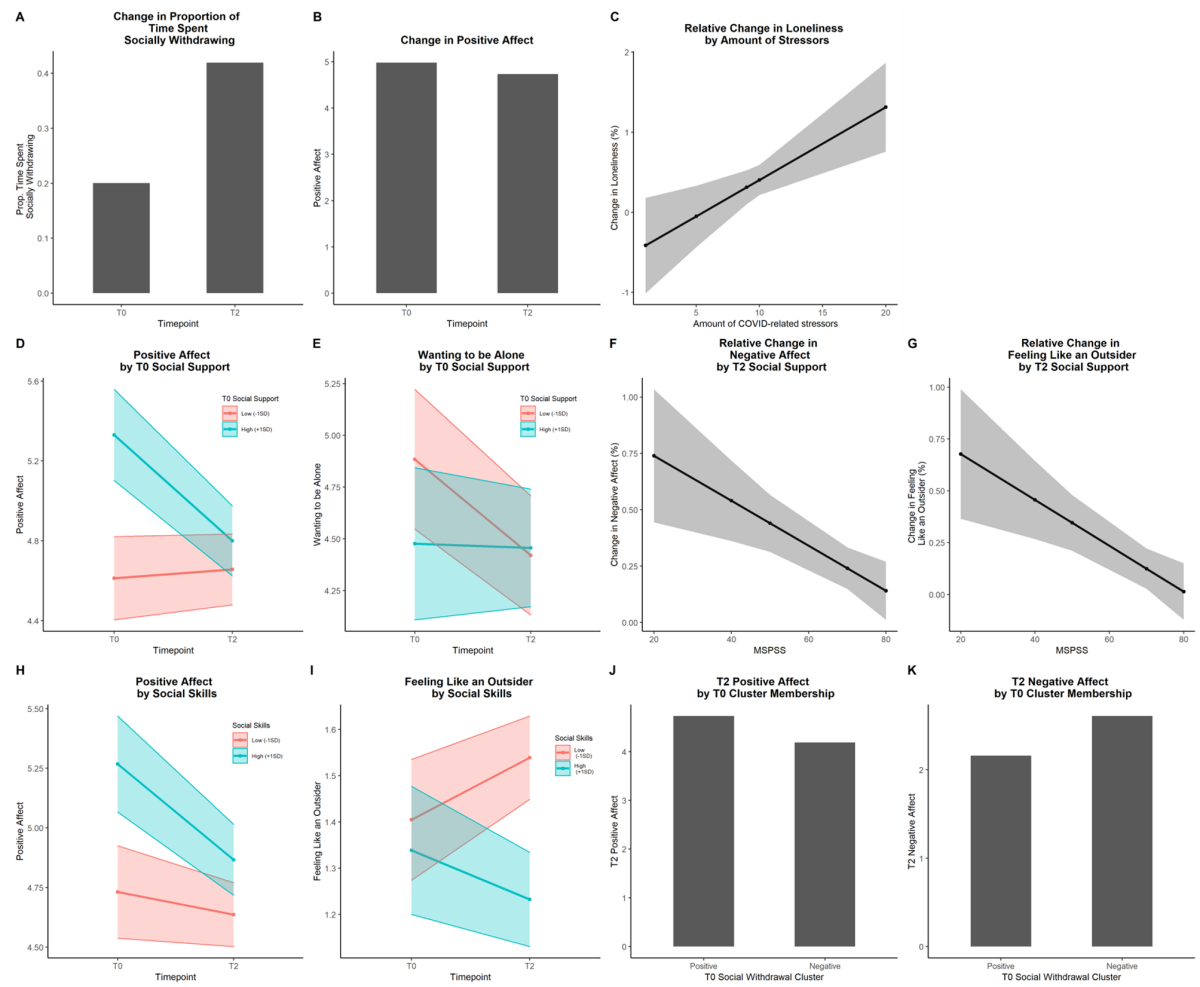
Change in Daily-Life Solitary experiences from T0 to T2 and moderators of change

The proportion of time spent alone increased significantly from T0 to T2 (*B*(*SE*) = 0.15(0.02), *p* <.001; Fig. 2A) and PA decreased significantly from T0 to T2 (*B*(*SE*)=-0.24(0.09), *p* =.006; Fig. 2B). There were no significant changes in NA, loneliness, finding it pleasant to be alone, wanting to be alone and feeling like an outsider from T0 to T2.

### Moderators of change in daily-life solitary experiences from pre-pandemic (T0) to mid-pandemic (T2)

The number of COVID-related stressors at T2 was significantly associated with the relative change in loneliness from T0 to T2 (*B*(*SE*) = 0.09(0.03), *p* =.002; Fig. 2C). Participants who reported more COVID-related stressors, reported more momentary loneliness at T2 relative to their T0 person-level mean of loneliness. However, there was no main effect of time point on changes in loneliness from T0 to T2.

There was a significant interaction between T0 social support and time point for PA (*B*(*SE*)=-0.05(0.01), *p* <.001; Fig. 2D) and wanting to be alone (*B*(*SE*) = 0.04 (0.02), *p* =.006; Fig. 2E). More T0 social support was associated with a steeper decrease in PA, while less T0 social support were associated with a steeper decrease in wanting to be alone.

T2 social support was significantly associated with relative change in NA (*B*(*SE*)=-0.01 (0.00), *p* =.003; Fig. 2F) and feeling like an outsider (*B*(*SE*)=-0.01(0.00), *p* =.001; Fig. 2G). More T2 social support were associated with less momentary NA and less momentary feeling like an outsider compared to the T0 person-level mean of these variables.

There was a significant interaction between T0 social skills and time point for PA (*B*(*SE*)=-0.02(0.01), *p* =.004; Fig. 2H) and feeling like an outsider (*B*(*SE*)=-0.02 (0.01), *p* =.001; Fig. 2I). Less T0 social skills were associated with an increase in feeling like an outsider, while more T0 social skills were associated with a decrease in feeling like an outsider. More T0 social skills were also associated with a steeper decrease in PA. Solitude cluster membership was significantly associated with T2 PA (*B*(*SE*)=-0.54 (0.17), *p* =.002; Fig. 2J) and NA (*B*(*SE*) = 0.45 (0.16), *p* =.007; Fig. 2K). Participants in the negative cluster had lower levels of PA and higher levels of NA at T2 compared to participants in the positive cluster.

### Sensitivity analyses

The attrition in the sample has resulted in some selection bias [see Additional File 1]. To assess the effect of this sampling bias, we conducted a sensitivity analysis for the T0– T2 analyses with a smaller subsample consisting of the participants who provided ESM data at all timepoints [see Additional File 6]. The sensitivity analysis showed that the overall trend of participants with fewer social resources (i.e., social support and social skills) reporting more negative solitary experiences at T2 was sustained.

## Discussion

The current study investigated changes in the proportion of adolescent time spent alone and daily-life solitary experiences from before the COVID-19 pandemic to the early and mid-pandemic period. The proportion of time spent alone had increased significantly from before to during the pandemic. Furthermore, the number of COVID-related stressors, social support, social skills, and general pre-pandemic solitary experiences were related to changes in solitary experiences at one or both stages of the pandemic.

Compared to the pre-pandemic period, the proportion of time spent alone increased 20% during the early-pandemic period in 2020. This increase does not fully overlap with the decrease in social interactions (29%) in the same sample [2]. This suggests that adolescents also spent a considerable amount of time in the presence of others without interacting with them. In the mid-pandemic period, adolescents spent around 22% more time alone compared to the pre-pandemic period. Adolescents’ social lives had seemingly not “returned to normal” (yet), despite the relaxation of certain measures during most of the mid-pandemic measurement period [22, 23]. In addition, adolescents reported significantly less positive affect when alone mid-pandemic, which suggests that solitary moments were experienced less positively as the pandemic continued.

The solitary experiences of adolescents with fewer social resources (i.e., social support and social skills) were impacted most at the early and mid-pandemic. At both time points, less pre-pandemic social support and fewer social skills were related to decreased levels of wanting to be alone and feeling more like an outsider. The increase in the proportion of time spent alone was also exacerbated for these adolescents in the early pandemic. The experiences of adolescents with more social resources, on the other hand, remained relatively positive throughout the pandemic, with decreases in feeling like an outsider at both time points and even an increase in wanting to be alone in the early pandemic. Therefore, it seems that social resources can buffer against the negative impact of the COVID-19 pandemic on solitary experiences. These finding are in line with our knowledge about the importance of social connection (especially with peers) during adolescence [44–46]– and with expectations about the socially disruptive effect of the COVID-19 pandemic [1].

A recent ESM study [47] showed that people who have an unmet need to belong — a motivational mechanism that moves people to form and maintain social relationships [48] — reported less PA and more NA when socially withdrawing, and lower levels of general wellbeing. It is possible that adolescents with more social resources could fulfil their need to belong even with fewer opportunities for social connection. Their pre-pandemic social resources potentially acted like a “reserve” to fulfil their need to belong during the lockdowns and restrictions, thereby reducing the negative impact of the pandemic on their solitary experiences (cf. evidence for the protective effect of social capital in adolescents; 49). The “reserve” of adolescents with fewer social resources might not have been as large, therefore making it harder for these adolescents to fulfil their need to belong during the pandemic.

However, the buffering effect of social resources seemed to lessen with the increased duration of the pandemic. Mid-pandemic, adolescents with more social resources reported a decrease in PA when alone, potentially indicating that their “reserve” of social resources was depleting. This could in turn have affected their solitary experiences. This indicates that *all* adolescents seem to lose out when restrictions on daily life continue over a longer term, even those with more resources. Therefore, concern for negative effects of increased solitude during the pandemic should not be restricted to just those adolescents with fewer social resources. This concern pertains to all adolescents, especially with the extended duration of the pandemic. Future studies should investigate how adolescents’ solitary experiences evolved after the pandemic, to assess whether this negative trend persisted. Future research should also pay attention to the relationship between changes in solitary experiences and the development of mental health problems, as such long-lasting negative effects of the pandemic on adolescent mental health might only manifest later.

Contrary to our hypothesis, adolescents with fewer social resources reported an increase in PA when alone during the early pandemic. This finding seemingly contradicts the decrease in wanting to be alone and increase in feeling like an outsider reported in the same period. At the time of measurement, the government had announced a forthcoming relaxation of the restrictions, including re-opening the schools. Although highly speculative, it is possible that the prospect of more social contact had a positive effect on adolescents’ PA when alone (cf. the concept of anticipatory social pleasure; 50).

Adolescents with more negative pre-pandemic solitary experiences reported more loneliness in the early pandemic. Mid-pandemic, they reported less PA and more NA compared to adolescents with more positive pre-pandemic solitary experiences. This could indicate that there is stability in negative solitary experiences in the SIGMA sample. It is an interesting avenue for future longitudinal research to investigate stability in solitude group membership across different samples.

More COVID-related stressors were associated with more loneliness mid-pandemic. This is in line with previous research during the early pandemic [16], where stressors were associated with decreased mental health and overall life satisfaction. Therefore, this suggests that adolescents who experienced more COVID-related stressors might be at prolonged risk for negative developmental outcomes.

### Strengths and limitations

This study is among the first to investigate changes in adolescents’ daily-life solitary experiences and potential moderators at multiple time points before and during the COVID-19 pandemic. An additional strength of this longitudinal study is the use of open science practices [51]. The research questions, hypotheses and analysis plans were post-registered [19, 20, 37] on the OSF using the registration template for ESM studies [36], and the analysis code is available on the OSF project page for this study [38] and in an additional file [see Additional File 4].

Some important limitations also need mentioning. Compliance to the ESM protocol was relatively low across waves (T0: 55% and 58%; T1: 48%; T2: 42%; vs. 74% on average in other adolescent ESM studies [52]). At T0, participants received a study phone from the researchers and completed many momentary questionnaires at school. Possibly, this could have led to higher compliance of the participants, as they might have felt more involved in the study.

There was also significant attrition in the T1 and T2 samples (*n* = 100 and *n* = 204, respectively) compared to the original T0 sample of *n* = 1913. While this has led to some selection bias [see Additional File 1], the conclusion that participants with more social resources reported better solitary experiences during the COVID-19 pandemic remained intact in the sensitivity analyses [see Additional File 6].

Finally, there were some differences in the questionnaires administered at each time point. This was particularly the case for the social support questionnaires (SSL only at T0, MSPSS only at T1, and both SSL and MSPSS at T2). We therefore framed social support as measured with the SSL as “pre-pandemic social support”. The lack of the SSL measure at T1 might have hampered any longitudinal comparisons.

## Conclusion

The COVID-19 pandemic had a major impact on the proportion of adolescent solitude as well as their daily-life solitary experiences, specifically for adolescents with few social resources. Adolescents with more social resources were initially protected against negative solitary experiences. However, with the increased duration of the pandemic, the buffering effect of social resources diminished. Altogether, this indicates that there should be continued concern for the needs of all adolescents, including those with sufficient social resources. Future research should therefore continue to investigate adolescents’ daily-life solitary experiences and mental health in the aftermath of the pandemic.

### Electronic supplementary material

Below is the link to the electronic supplementary material.

Supplementary Material 1

Supplementary Material 2

Supplementary Material 3

Supplementary Material 4

Supplementary Material 5

Supplementary Material 6

Supplementary Material 7

## Data Availability

The datasets generated and analysed during the current study are not publicly available due to our decision to only release data following a request through the Data Curation for Open Science (DROPS) data access system [51]. In the DROPS system, authors are asked to first create a time-stamped registration of their study before gaining access to the data, thereby uniquely enabling the adherence to best Open Science practices when working with secondary data. All ESM items included in this study are available in the ESM Item Repository (dataset ‘sigma’ [53]) along with all other ESM items administered in the SIGMA project at T0, T1 and T2. The interactive codebook for the SIGMA project is available online [54].

## Abbreviations

ESM: Experience Sampling Method
SSL(-I-12): Social Support List(-Interactions-12)
MSPSS: Multidimensional Scale of Perceived Social Support
VPV: Vragenlijst Persoonlijke Vaardigheden (Personal Skills Questionnaire)
PA: Positive affect
NA: Negative affect
OSF: Open Science Framework
MICE: Multiple Imputation by Chained Equations
