# Supplementary material for: Changes in adolescents’ daily-life solitary experiences during the COVID-19 pandemic: an experience sampling study
Source: BMC Public Health. 2024 Apr 26;24:1172. doi: 10.1186/s12889-024-18458-1 (PMC11046767; doi:10.1186/s12889-024-18458-1)
Supplement: Supplementary file 1 — Supplementary Material 1 [file 12889_2024_18458_MOESM1_ESM.docx]

Overview of Additional Files

1. AF1_SampleBias_socwithdrCOVID.docx
Differences at T0 Between the T2 Subsample and the Larger T0 Sample: A between-groups comparison of T0 descriptive statistics of participants who did and did not re-enrol at T2. Contains Table S1.

2. AF2_CorrelationTables_socwithdrCOVID.docx
Correlation Tables: Correlations with confidence intervals for all time points. Contains Tables S2-S5.

3. AF3_TransparentChanges_socwithdrCOVID.docx
Transparent Changes to the Post-Registrations: An overview of all changes made to the analyses described in the post-registration documents (19, 20)

4. AF4_AnalysisCode_sociwithdrCOVID.docx
Analysis code for manuscript ‘Changes in Adolescents’ Daily-Life Solitary Experiences During the COVID-19 Pandemic’: R script detailing all code used to perform the analyses described in this study

5. AF5_Analyses_socwithdrCOVID.docx
Missing Data Imputation and Statistical Analyses: A detailed description of the missing data imputation and analyses performed in this study

6. AF6_SensitivityAnalysis_socwithdrCOVID.docx
T2 Sensitivity Analysis: A sensitivity analysis performed on the subgroup of participants who reported moments alone at T0, T1 and T2. Contains Table S6 and Figure S1.
