# Supplementary material for: Changes in adolescents’ daily-life solitary experiences during the COVID-19 pandemic: an experience sampling study
Source: BMC Public Health. 2024 Apr 26;24:1172. doi: 10.1186/s12889-024-18458-1 (PMC11046767; doi:10.1186/s12889-024-18458-1)
Supplement: Supplementary file 2 — Supplementary Material 2 [file 12889_2024_18458_MOESM2_ESM.docx]

**Additional File 2**

**Correlation Tables**

**Table S2**

*Correlations with Confidence Intervals for the T0 Sample Used for Comparisons with the T1 Sample*

| Variable | 1 | 2 | 3 | 4 | 5 | 6 | 7 | 8 | 9 |
| --- | --- | --- | --- | --- | --- | --- | --- | --- | --- |
|  |  |  |  |  |  |  |  |  |  |
| 1. Positive Affect |  |  |  |  |  |  |  |  |  |
|  |  |  |  |  |  |  |  |  |  |
| 2. Negative Affect | -.74** |  |  |  |  |  |  |  |  |
|  | [-.82, -.64] |  |  |  |  |  |  |  |  |
|  |  |  |  |  |  |  |  |  |  |
| 3. Loneliness | -.45** | .62** |  |  |  |  |  |  |  |
|  | [-.60, -.28] | [.49, .73] |  |  |  |  |  |  |  |
|  |  |  |  |  |  |  |  |  |  |
| 4. Finding it Pleasant to be Alone | .19 | -.18 | -.36** |  |  |  |  |  |  |
|  | [-.01, .37] | [-.37, .02] | [-.53, -.18] |  |  |  |  |  |  |
|  |  |  |  |  |  |  |  |  |  |
| 5. Feeling Like an Outsider | -.41** | .46** | .52** | -.26* |  |  |  |  |  |
|  | [-.56, -.23] | [.28, .60] | [.36, .65] | [-.43, -.06] |  |  |  |  |  |
|  |  |  |  |  |  |  |  |  |  |
| 6. Wanting to be Alone | -.12 | .10 | -.11 | .65** | .01 |  |  |  |  |
|  | [-.31, .08] | [-.11, .29] | [-.31, .09] | [.51, .75] | [-.19, .20] |  |  |  |  |
|  |  |  |  |  |  |  |  |  |  |
| 7. Proportion of Time Spent Alone | -.25* | .11 | .03 | .07 | -.06 | .10 |  |  |  |
|  | [-.43, -.06] | [-.10, .30] | [-.18, .22] | [-.13, .27] | [-.26, .14] | [-.10, .29] |  |  |  |
|  |  |  |  |  |  |  |  |  |  |
| 8. Social Skills | .35** | -.28** | -.16 | .08 | -.14 | .04 | -.17 |  |  |
|  | [.16, .52] | [-.46, -.08] | [-.35, .05] | [-.13, .28] | [-.34, .07] | [-.17, .24] | [-.36, .04] |  |  |
|  |  |  |  |  |  |  |  |  |  |
| 9. Social Support (T0) | .27** | -.26* | -.28** | .04 | -.19 | -.05 | .01 | .60** |  |
|  | [.07, .45] | [-.44, -.06] | [-.46, -.08] | [-.16, .24] | [-.38, .01] | [-.25, .16] | [-.19, .21] | [.44, .71] |  |
|  |  |  |  |  |  |  |  |  |  |
| 10. Age | -.35** | .22* | .08 | .07 | .05 | .09 | .26* | -.11 | -.06 |
|  | [-.52, -.17] | [.02, .40] | [-.12, .28] | [-.13, .27] | [-.15, .25] | [-.11, .29] | [.06, .43] | [-.31, .10] | [-.26, .15] |
|  |  |  |  |  |  |  |  |  |  |

*Note.* Values in square brackets indicate the 95% confidence interval for each correlation.

* *p* < .05, ** *p* < .01

**Table S3**

*Correlations with Confidence Intervals for the T1 Sample*

| Variable | 1 | 2 | 3 | 4 | 5 | 6 | 7 | 8 | 9 | 10 |
| --- | --- | --- | --- | --- | --- | --- | --- | --- | --- | --- |
|  |  |  |  |  |  |  |  |  |  |  |
| 1. Positive Affect |  |  |  |  |  |  |  |  |  |  |
|  |  |  |  |  |  |  |  |  |  |  |
| 2. Negative Affect | -.73** |  |  |  |  |  |  |  |  |  |
|  | [-.81, -.62] |  |  |  |  |  |  |  |  |  |
|  |  |  |  |  |  |  |  |  |  |  |
| 3. Loneliness | -.62** | .74** |  |  |  |  |  |  |  |  |
|  | [-.73, -.49] | [.63, .82] |  |  |  |  |  |  |  |  |
|  |  |  |  |  |  |  |  |  |  |  |
| 4. Finding it Pleasant to be Alone | .32** | -.24* | -.45** |  |  |  |  |  |  |  |
|  | [.13, .48] | [-.42, -.05] | [-.59, -.27] |  |  |  |  |  |  |  |
|  |  |  |  |  |  |  |  |  |  |  |
| 5. Feeling Like an Outsider | -.59** | .70** | .72** | -.26** |  |  |  |  |  |  |
|  | [-.71, -.45] | [.58, .79] | [.61, .80] | [-.43, -.06] |  |  |  |  |  |  |
|  |  |  |  |  |  |  |  |  |  |  |
| 6. Wanting to be Alone | .19 | -.09 | -.31** | .84** | -.16 |  |  |  |  |  |
|  | [-.01, .37] | [-.28, .11] | [-.48, -.12] | [.77, .89] | [-.34, .04] |  |  |  |  |  |
|  |  |  |  |  |  |  |  |  |  |  |
| 7. Proportion of Time Spent Alone | -.19 | .08 | .21* | -.17 | .20 | -.06 |  |  |  |  |
|  | [-.37, .01] | [-.12, .27] | [.02, .39] | [-.35, .03] | [-.00, .38] | [-.26, .13] |  |  |  |  |
|  |  |  |  |  |  |  |  |  |  |  |
| 8. Amount of COVID-Related Stressors | -.36** | .34** | .36** | -.10 | .23* | -.11 | .06 |  |  |  |
|  | [-.52, -.18] | [.16, .50] | [.17, .52] | [-.29, .10] | [.04, .41] | [-.30, .09] | [-.14, .25] |  |  |  |
|  |  |  |  |  |  |  |  |  |  |  |
| 9. Mean Burdensomeness of COVID-Related Stressors | -.38** | .35** | .37** | -.20* | .20* | -.11 | .10 | .21* |  |  |
|  | [-.54, -.20] | [.17, .52] | [.19, .53] | [-.39, -.01] | [.00, .38] | [-.30, .09] | [-.10, .29] | [.02, .39] |  |  |
|  |  |  |  |  |  |  |  |  |  |  |
|  |  |  |  |  |  |  |  |  |  |  |
| Variable | 1 | 2 | 3 | 4 | 5 | 6 | 7 | 8 | 9 | 10 |
|  |  |  |  |  |  |  |  |  |  |  |
| 10. Social Support (T1) | .29** | -.14 | -.16 | -.13 | -.27** | -.12 | -.11 | -.08 | -.11 |  |
|  | [.10, .46] | [-.32, .06] | [-.35, .04] | [-.32, .07] | [-.45, -.08] | [-.31, .08] | [-.30, .09] | [-.27, .12] | [-.30, .09] |  |
|  |  |  |  |  |  |  |  |  |  |  |
| 11. Age | -.37** | .11 | .02 | -.24* | .08 | -.27** | .03 | .02 | .18 | -.12 |
|  | [-.53, -.19] | [-.09, .30] | [-.17, .22] | [-.42, -.05] | [-.12, .27] | [-.45, -.08] | [-.16, .23] | [-.18, .21] | [-.02, .36] | [-.31, .08] |
|  |  |  |  |  |  |  |  |  |  |  |

*Note.* Values in square brackets indicate the 95% confidence interval for each correlation.

* *p* < .05, ** *p* < .01

**Table S4**

*Correlations with Confidence Intervals for the T0 Sample Used for Comparisons with the T2 Sample*

| Variable | 1 | 2 | 3 | 4 | 5 | 6 | 7 | 8 | 9 |
| --- | --- | --- | --- | --- | --- | --- | --- | --- | --- |
|  |  |  |  |  |  |  |  |  |  |
| 1. Positive Affect |  |  |  |  |  |  |  |  |  |
|  |  |  |  |  |  |  |  |  |  |
| 2. Negative Affect | -.54** |  |  |  |  |  |  |  |  |
|  | [-.63, -.43] |  |  |  |  |  |  |  |  |
|  |  |  |  |  |  |  |  |  |  |
| 3. Loneliness | -.23** | .47** |  |  |  |  |  |  |  |
|  | [-.36, -.08] | [.35, .58] |  |  |  |  |  |  |  |
|  |  |  |  |  |  |  |  |  |  |
| 4. Finding it Pleasant to be Alone | .05 | -.01 | -.18* |  |  |  |  |  |  |
|  | [-.10, .19] | [-.15, .14] | [-.32, -.04] |  |  |  |  |  |  |
|  |  |  |  |  |  |  |  |  |  |
| 5. Feeling Like an Outsider | -.23** | .30** | .24** | -.15* |  |  |  |  |  |
|  | [-.36, -.09] | [.16, .43] | [.10, .38] | [-.29, -.01] |  |  |  |  |  |
|  |  |  |  |  |  |  |  |  |  |
| 6. Wanting to be Alone | -.07 | .02 | -.18* | .70** | .02 |  |  |  |  |
|  | [-.22, .07] | [-.12, .17] | [-.32, -.04] | [.62, .77] | [-.12, .16] |  |  |  |  |
|  |  |  |  |  |  |  |  |  |  |
| 7. Proportion of Time Spent Alone | -.31** | .20** | .06 | .15* | .02 | .18* |  |  |  |
|  | [-.43, -.17] | [.06, .33] | [-.08, .21] | [.00, .29] | [-.13, .16] | [.04, .32] |  |  |  |
|  |  |  |  |  |  |  |  |  |  |
| 8. Social Skills | .25** | -.14 | -.07 | .05 | -.18* | .05 | -.23** |  |  |
|  | [.10, .39] | [-.29, .01] | [-.22, .08] | [-.10, .20] | [-.33, -.03] | [-.11, .20] | [-.36, -.08] |  |  |
|  |  |  |  |  |  |  |  |  |  |
| 9. Social Support (T0) | .23** | -.13 | -.03 | -.00 | -.22** | -.06 | -.14 | .51** |  |
|  | [.08, .36] | [-.27, .02] | [-.17, .12] | [-.15, .14] | [-.35, -.07] | [-.21, .09] | [-.28, .01] | [.39, .61] |  |
|  |  |  |  |  |  |  |  |  |  |
| 10. Age | -.39** | .27** | .10 | .17* | .11 | .20** | .39** | -.13 | -.12 |
|  | [-.51, -.26] | [.13, .40] | [-.05, .24] | [.02, .31] | [-.04, .25] | [.05, .33] | [.26, .51] | [-.27, .02] | [-.26, .03] |
|  |  |  |  |  |  |  |  |  |  |

*Note.* Values in square brackets indicate the 95% confidence interval for each correlation.

* *p* < .05, ** *p* < .01

**Table S5**

*Correlations with Confidence Intervals for the T2 Sample*

| Variable | 1 | 2 | 3 | 4 | 5 | 6 | 7 | 8 | 9 | 10 | 11 |
| --- | --- | --- | --- | --- | --- | --- | --- | --- | --- | --- | --- |
|  |  |  |  |  |  |  |  |  |  |  |  |
| 1. Positive Affect |  |  |  |  |  |  |  |  |  |  |  |
|  |  |  |  |  |  |  |  |  |  |  |  |
| 2. Negative Affect | -.74** |  |  |  |  |  |  |  |  |  |  |
|  | [-.80, -.68] |  |  |  |  |  |  |  |  |  |  |
|  |  |  |  |  |  |  |  |  |  |  |  |
| 3. Loneliness | -.52** | .67** |  |  |  |  |  |  |  |  |  |
|  | [-.61, -.41] | [.58, .74] |  |  |  |  |  |  |  |  |  |
|  |  |  |  |  |  |  |  |  |  |  |  |
| 4. Finding it Pleasant to be Alone | .27** | -.25** | -.43** |  |  |  |  |  |  |  |  |
|  | [.14, .39] | [-.37, -.11] | [-.53, -.31] |  |  |  |  |  |  |  |  |
|  |  |  |  |  |  |  |  |  |  |  |  |
| 5. Feeling Like an Outsider | -.43** | .48** | .44** | -.19** |  |  |  |  |  |  |  |
|  | [-.54, -.31] | [.37, .58] | [.32, .54] | [-.32, -.05] |  |  |  |  |  |  |  |
|  |  |  |  |  |  |  |  |  |  |  |  |
| 6. Wanting to be Alone | .08 | -.04 | -.29** | .80** | .03 |  |  |  |  |  |  |
|  | [-.05, .22] | [-.18, .10] | [-.41, -.16] | [.75, .85] | [-.10, .17] |  |  |  |  |  |  |
|  |  |  |  |  |  |  |  |  |  |  |  |
| 7. Proportion of Time Alone | -.10 | .13 | .12 | .05 | .02 | .02 |  |  |  |  |  |
|  | [-.24, .04] | [-.00, .27] | [-.02, .25] | [-.09, .18] | [-.12, .16] | [-.12, .15] |  |  |  |  |  |
|  |  |  |  |  |  |  |  |  |  |  |  |
| 8. Social Skills | .26** | -.18* | -.21** | .03 | -.33** | -.05 | -.09 |  |  |  |  |
|  | [.12, .39] | [-.31, -.04] | [-.34, -.07] | [-.11, .17] | [-.45, -.20] | [-.19, .09] | [-.23, .05] |  |  |  |  |
|  |  |  |  |  |  |  |  |  |  |  |  |
| 9. Amount of COVID-Related Stressors | -.16* | .16* | .14 | -.04 | -.01 | -.05 | .03 | -.09 |  |  |  |
|  | [-.29, -.02] | [.02, .30] | [-.00, .28] | [-.18, .10] | [-.15, .13] | [-.19, .09] | [-.11, .17] | [-.23, .06] |  |  |  |
| Variable | 1 | 2 | 3 | 4 | 5 | 6 | 7 | 8 | 9 | 10 | 11 |
|  |  |  |  |  |  |  |  |  |  |  |  |
| 10. Mean Burdensomeness of COVID-Related Stressors | -.24** | .20** | .15* | -.11 | .04 | -.15* | .11 | -.12 | .21** |  |  |
|  | [-.36, -.10] | [.06, .33] | [.01, .29] | [-.25, .03] | [-.10, .18] | [-.28, -.01] | [-.03, .25] | [-.26, .02] | [.07, .34] |  |  |
|  |  |  |  |  |  |  |  |  |  |  |  |
| 11. Social Support (T1) | .34** | -.27** | -.22** | .02 | -.35** | -.07 | -.22** | .47** | -.07 | -.24** |  |
|  | [.21, .46] | [-.40, -.14] | [-.36, -.09] | [-.12, .17] | [-.47, -.22] | [-.21, .07] | [-.35, -.09] | [.35, .57] | [-.21, .07] | [-.37, -.10] |  |
|  |  |  |  |  |  |  |  |  |  |  |  |
| 12. Age | -.12 | .05 | .07 | -.08 | .07 | -.10 | .15* | -.08 | -.01 | .29** | -.18* |
|  | [-.26, .01] | [-.08, .19] | [-.06, .21] | [-.22, .05] | [-.07, .20] | [-.24, .03] | [.02, .29] | [-.22, .06] | [-.15, .13] | [.15, .41] | [-.31, -.04] |
|  |  |  |  |  |  |  |  |  |  |  |  |

*Note.* Values in square brackets indicate the 95% confidence interval for each correlation.

* *p* < .05, ** *p* < .01
