# Supplementary material for: Changes in adolescents’ daily-life solitary experiences during the COVID-19 pandemic: an experience sampling study
Source: BMC Public Health. 2024 Apr 26;24:1172. doi: 10.1186/s12889-024-18458-1 (PMC11046767; doi:10.1186/s12889-024-18458-1)
Supplement: Supplementary file 3 — Supplementary Material 3 [file 12889_2024_18458_MOESM3_ESM.docx]

Additional File 1

Differences at T0 Between the T2 Subsample and the Larger T0 Sample

**Table S1**

*Descriptive Statistics at T0 (Including Statistical Differences) for the n = 219 Participants Who Reported Moments Alone at T0 and Re-Enrolled at T2, and the n = 1026 Participants Who Reported Moments Alone at T0 but Did Not Re-Enrol at T2.*

| Variable | T2 sample | | | | | Non-T2 sample | | | | |  |
| --- | --- | --- | --- | --- | --- | --- | --- | --- | --- | --- | --- |
|  | *n* | *M* | *SD* | Median | Min - Max | *n* | *M* | *SD* | Median | Min - Max | Statistical Difference |
| Age | 218 | 13.9 | 1.9 | 14.0 | 11.0 – 19.0 | 1024 | 13.8 | 1.9 | 13.0 | 11.0 – 20.0 | *t* (*df*) = 0.49 (313.02), *p* = .62 |
| Gender (%female) | 219 | 74.9% |  |  |  | 1026 | 62.2% |  |  |  | ***χ^2^* (*df*) = 12.16 (1), *p* < .001** |
| Compliance^a^ | 219 | 0.6 | 0.2 | 0.5 | 0.13 – 0.98 | 1026 | 0.4 | 0.2 | 0.4 | 0.02 – 0.97 | ***t* (*df*) = 7.34 (330.20), *p* < .001** |
| Positive Affect | 219 | 5.1 | 1.2 | 5.3 | 1.5 – 7.0 | 1026 | 4.9 | 1.4 | 5.1 | 1.0 – 7.0 | ***t* (*df*) = 2.03 (358.27), *p* = .04** |
| Negative Affect | 219 | 2.1 | 1.1 | 1.7 | 1.0 – 7.0 | 1026 | 2.1 | 1.1 | 1.9 | 1.0 – 7.0 | *t* (*df*) = -0.96 (335.23), *p* = .34 |
| Loneliness | 219 | 2.2 | 1.5 | 1.5 | 1.0 – 7.0 | 1026 | 2.2 | 1.6 | 1.6 | 1.0 – 7.0 | *t* (*df*) = -0.50 (331.20), *p* = .62 |
|  |  |  |  |  |  |  |  |  |  |  |  |
| Variable | T2 sample | | | | | Non-T2 sample | | | | |  |
|  | *n* | *M* | *SD* | Median | Min - Max | *n* | *M* | *SD* | Median | Min - Max | Statistical Difference |
| Finding it Pleasant to be Alone | 219 | 5.1 | 1.7 | 5.4 | 1.0 – 7.0 | 1026 | 4.5 | 1.9 | 4.9 | 1.0 – 7.0 | ***t* (*df*) = 4.28 (350.48), *p* < .001** |
| Feeling Like an Outsider | 219 | 1.4 | 0.9 | 1.0 | 1.0 – 7.0 | 1026 | 1.6 | 1.2 | 1.0 | 1.0 – 7.0 | ***t* (*df*) = -2.33 (393.58), *p* = .02** |
| Wanting to be Alone | 219 | 4.6 | 1.7 | 4.8 | 1.0 – 7.0 | 1026 | 3.8 | 2.0 | 4.0 | 1.0 – 7.0 | ***t* (*df*) = 6.04 (349.40), *p* < .001** |
| Proportion of Time Spent Alone | 219 | 0.2 | 0.1 | 0.1 | 0.02 – 0.7 | 1026 | 0.2 | 0.2 | 0.1 | 0.02 – 1 | *t* (*df*) = -0.48 (362.94), *p* = .63 |
| Social Support (T0) | 212 | 23.3 | 5.7 | 23.0 | 5.0 – 35.0 | 962 | 22.6 | 6.2 | 24.0 | 0.0 – 36.0 | *t* (*df*) = 1.69 (334.22), *p* = .09 |
| Social Skills | 201 | 69.9 | 7.1 | 70.0 | 53.0 – 90.0 | 845 | 69.4 | 7.7 | 70.0 | 18.0 – 89.0 | *t* (*df*) = 0.96 (323.32), *p* = .34 |
| Solitude Cluster Membership (% positive cluster) | 219 | 72.1% |  |  |  | 1026 | 71.2% |  |  |  | *χ^2^* (*df*) = 0.05 (1), *p* = .83 |

*Note.* *p*-values < .05 are highlighted in bold.

^a^ The compliance represents the proportion of non-missing momentary questionnaire

Table S1 shows that there are statistical differences between the group of participants who did re-enrol at T2 and those who did not re-enrol at T2. The group of participants who re-enrolled consists of more girls and of participants with higher compliance at T0. These participants also reported higher levels of PA when alone at T0, as well as higher levels of finding it pleasant to be alone and wanting to be alone, and lower levels of feeling like an outsider. The groups did not differ significantly in the proportion of time they spent alone at T0.
