# Supplementary material for: Changes in adolescents’ daily-life solitary experiences during the COVID-19 pandemic: an experience sampling study
Source: BMC Public Health. 2024 Apr 26;24:1172. doi: 10.1186/s12889-024-18458-1 (PMC11046767; doi:10.1186/s12889-024-18458-1)
Supplement: Supplementary file 4 — Supplementary Material 4 [file 12889_2024_18458_MOESM4_ESM.docx]

Additional File 3

Transparent Changes to the Post-Registrations

Both in the original post-registration and in the addendum, we mentioned that the moderating role of the number of COVID-related stressors, the mean burdensomeness of COVID-related stressors, social support (at T0, T1 and T2) and social skills would be investigated by adding an interaction of each variable with Timepoint to the regression models. However, we realised during the data analysis that this approach would only be possible for social support at T0 and social skills.

The measures used for COVID-related stressors and social support at T1 and T2 were not administered at T0. Therefore, it was not possible to add an interaction between these variables and Timepoint. In order to still make inferences about the effect of these variables on the change in outcome variables across time points, we opted to calculate a relative change score for each outcome variable. For the person-level outcome (amount of time spent socially withdrawing), this relative change score at T1 was calculated by subtracting the amount of time spent socially withdrawing at T0 from the amount of time spent socially withdrawing at T1 and then dividing the outcome by the amount of time spent socially withdrawing at T0. The relative change score was calculated for each participant. The same procedure was followed to calculate the relative change score for amount of time spent socially withdrawing at T2. Positive relative change scores indicate a higher amount of time spent socially withdrawing at T0, while negative relative change scores indicate a higher amount of time spent socially withdrawing at T1 or T2. For the moment-level outcomes, the relative change score was calculated as follows (using PA at T1 as an example): for every moment-level value of PA at T1, the participant’s person-level mean of PA at T0 was subtracted from this value and the outcome was divided by the participant’s person-level mean of PA at T0. Here, positive relative change scores indicate that the participant’s momentary PA at T1 was higher than their mean PA at T0, while negative relative change scores indicate that the participant’s momentary PA at T1 was lower than their mean PA at T0. The relative change scores for the other moment-level outcomes (NA, loneliness, finding it pleasant to be alone, wanting to be alone and feeling like an outsider) at T1 and T2 were calculated in the same way.

Given that the pre-pandemic solitude clusters were formed based on the T0 values of the outcome variables (1), it was not possible to add an interaction between solitude cluster membership and Timepoint to the regression models in order to assess the moderating role of solitude cluster membership on the outcome variables. If we had taken this approach, as was originally planned in the post-registrations, the results would have been mainly driven by the association between the T0 values of the outcome variables and the clusters. We realized this during data analysis. In order to completely exclude any confounding effects of T0 outcome values, we decided to remove Timepoint as a predictor and only investigate the effect of solitude cluster membership on T1 and T2 outcomes.

References

1. Bamps E, Teixeira A, Lafit G, Achterhof R, Hagemann N, Hermans KSFM, et al. Identifying clusters of adolescents based on their daily-life social withdrawal experience. J Youth Adolesc. 2022 Jan 23;51:915-926. Available from: https://doi.org/10.1007/s10964-021-01558-1
