# Supplementary material for: Changes in adolescents’ daily-life solitary experiences during the COVID-19 pandemic: an experience sampling study
Source: BMC Public Health. 2024 Apr 26;24:1172. doi: 10.1186/s12889-024-18458-1 (PMC11046767; doi:10.1186/s12889-024-18458-1)

Additional File 6

T2 Sensitivity Analysis

As described in the Addendum to the post-registration (1), we conducted a sensitivity analysis for the analyses comparing T0 to T2. The sensitivity analysis was added because there is no direct comparison between T1 and T2 included in the paper. This direct comparison was not deemed feasible by the researchers because of expected sample size issues (and related power issues) due to the large attrition at T1 and T2. The full T2 sample consisted of all participants who provided ESM data when alone at T2, regardless of participation at T1. For this sensitivity analysis, a subsample of the T2 sample was used with only those participants who provided ESM data when alone at T0, T1 and T2. This subsample consisted of *n* = 60 at T0 and *n* = 64 at T2. Descriptive statistics for this subsample can be found in Table 6.1.

Pre-Pandemic (T0) to Mid-Pandemic (T2) Change in Daily-Life Solitary Experiences

The results showed that the proportion of time spent socially withdrawing increased significantly from T0 to T2 (*B*(*SE*) = 0.16 (0.04), *p* < .001, Fig. 6.1 A), which is in line with the results in the full T2 sample. In contrast with the results from the full sample, there was no significant change in PA from T0 to T2. However, there was a significant decrease in finding it pleasant to be alone (*B*(*SE*) = -0.58 (0.22), *p* = .0068, Fig. 6.1 B) and a significant increase in loneliness from T0 to T2 (*B*(*SE*) = 0.72 (0.22), *p* = .001, Fig. 6.1 C).

Moderators of Change in Daily-Life Solitary Experiences from T0 to T2

There was a significant association between the number of COVID-related stressors at T2 and relative change in loneliness from T0 to T2 (*B*(*SE*) = 0.19 (0.05), *p* < .001, Fig. 6.1 D), similar to the results in the full T2 sample. This indicates that participants in the subsample who reported more COVID-related stressors at T2, also reported more momentary loneliness at T2 compared to their person-mean level of loneliness at T0.

Contrary to the results in the full T2 sample, there was no significant interaction between social support at T0 and time point for PA. However, the significant interaction between social support at T0 and time point for wanting to be alone was replicated in the subsample (*B*(*SE*) = 0.10 (0.02), *p* < .001, Fig. 6.1 E). There was also a significant interaction between social support at T0 and time point for finding it pleasant to be alone (*B*(*SE*) = 0.07 (0.02), *p* < .001, Fig.6.1 F) that was not present in the results in the full T2 sample. Lower levels of T0 social support were associated with steeper decreases in wanting to be alone and finding it pleasant to be alone.

There was no significant association of T2 social support on relative change in any of the outcomes. This means that the significant association between T2 social support and relative change in NA and in feeling like an outsider in the full T2 sample could not be replicated in the subsample.

The significant interaction between social skills and time point for PA and feeling like an outsider in the full T2 sample could not be replicated in the subsample. However, there was a significant interaction between social skills and time point for finding it pleasant to be alone (*B*(*SE*) = 0.05 (0.02), *p* = .002, Fig. 6.1 G) and for wanting to be alone (*B*(*SE*) = 0.06 (0.02), *p* < .001, Fig. 6.1 H). Lower levels of social skills were associated with steeper decreases in both finding it pleasant to be alone and wanting to be alone.

Contrary to the results in the full T2 sample, there was no significant association between solitude cluster membership and T2 PA in the subsample. There was also no significant association between solitude cluster membership and T2 NA in the subsample.

Overall, the results of the sensitivity analysis showed that the specific significant outcomes differed somewhat between the full T2 sample and the sensitivity subsample. However, the general trend of participants with less social resources (i.e. social support and social skills) reporting worse solitary experiences at T2 is sustained in the smaller sensitivity sample.

References

1. Bamps E, Achterhof R, Teixeira A, Myin-Germeys I. Adolescents’ Daily-Life Social Withdrawal Experiences During the COVID-19 Lockdown – Addendum to the post-registration. <https://osf.io/kryxu/?view_only=9929b7eca5384367a9045677e49e6c9d>

**Table S6**

*Descriptive Statistics of the T0 and T2 Subsamples Used for the Sensitivity Analysis*

| Variable | T0 | | | | | T2 | | | | |
| --- | --- | --- | --- | --- | --- | --- | --- | --- | --- | --- |
|  | *n* | *M* | *SD* | Median | Min - Max | *n* | *M* | *SD* | Median | Min - Max |
| Age | 60 | 14.5 | 1.7 | 14.0 | 12.0 – 18.0 | 64 | 17.0 | 1.9 | 17.0 | 14.0 – 20.0 |
| Gender (%female) | 60 | 95.0% |  |  |  | 64 | 95.3% |  |  |  |
| Compliance^a^ | 60 | 0.6 | 0.2 | 0.6 | 0.2 – 0.98 | 64 | 0.5 | 0.2 | 0.4 | 0.07 – 0.98 |
| Positive Affect | 60 | 4.9 | 1.2 | 4.9 | 1.0 – 6.9 | 64 | 4.3 | 1.1 | 4.3 | 1.8 – 6.4 |
| Negative Affect | 60 | 2.1 | 1.1 | 1.8 | 1.0 – 6.2 | 64 | 2.6 | 1.3 | 2.3 | 1.0 – 5.6 |
| Loneliness | 60 | 2.1 | 1.6 | 1.3 | 1.0 – 7.0 | 64 | 2.5 | 1.5 | 1.9 | 1.0 – 6.5 |
| Finding it Pleasant to be Alone | 60 | 5.6 | 1.6 | 6.1 | 1.0 – 7.0 | 64 | 4.9 | 1.3 | 5.0 | 1.0 – 7.0 |
| Feeling Like an Outsider | 60 | 1.5 | 1.2 | 1.0 | 1.0 – 7.0 | 64 | 1.5 | 0.7 | 1.2 | 1.0 – 4.0 |
| Wanting to be Alone | 60 | 5.1 | 1.6 | 5.5 | 1.0 – 7.0 | 64 | 4.6 | 1.3 | 4.8 | 1.0 – 7.0 |
| Proportion of Time Spent Socially Withdrawing | 60 | 0.2 | 0.1 | 0.2 | 0.03 – 0.7 | 64 | 0.4 | 0.2 | 0.4 | 0.07 – 1 |
| Social Support (T0) | 58 | 22.4 | 6.3 | 21.5 | 5.0 – 34.0 |  |  |  |  |  |
| Social Skills | 58 | 69.7 | 7.5 | 70.5 | 55.0 – 90.0 | 61 | 68.7 | 8.4 | 69.0 | 43.0 – 84.0 |
| Social Withdrawal Cluster Membership (% positive cluster) | 51 | 86.3% |  |  |  |  |  |  |  |  |
| Amount of COVID-Related Stressors |  |  |  |  |  | 57 | 11.1 | 3.3 | 12.0 | 1.0 – 17.0 |
| Mean Burdensomeness of COVID-Related Stressors |  |  |  |  |  | 56 | 3.4 | 0.5 | 3.3 | 2.2 – 5.0 |
| Variable | T0 | | | | | T1 | | | | |
|  | *n* | *M* | *SD* | Median | Min - Max | *n* | *M* | *SD* | Median | Min - Max |
| Social Support (T2) |  |  |  |  |  | 60 | 62.5 | 16.0 | 66.5 | 22.0 – 84.0 |

^a^ The compliance represents the proportion of non-missing momentary questionnaires.

**Figure S1**

*Results of the Sensitivity Analysis*


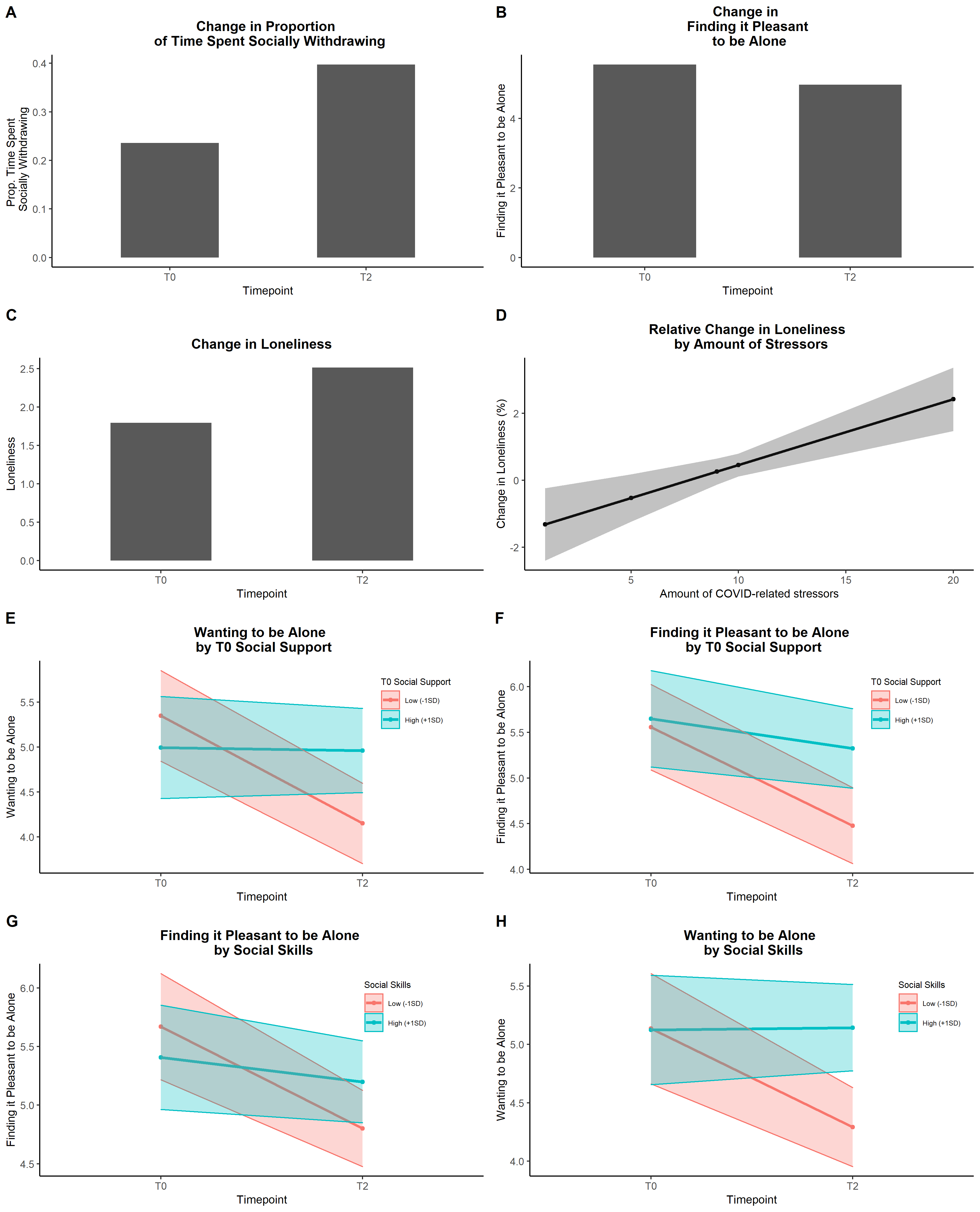

Supplement: Supplementary file 5 — Supplementary Material 5 [file 12889_2024_18458_MOESM5_ESM.docx]
