# Supplementary material for: Changes in adolescents’ daily-life solitary experiences during the COVID-19 pandemic: an experience sampling study
Source: BMC Public Health. 2024 Apr 26;24:1172. doi: 10.1186/s12889-024-18458-1 (PMC11046767; doi:10.1186/s12889-024-18458-1)
Supplement: Supplementary file 6 — Supplementary Material 6 [file 12889_2024_18458_MOESM6_ESM.docx]

Additional File 5

Missing Data Imputation and Statistical Analyses

Missing Data Imputation

There was a considerable amount of missing data in the retrospective questionnaires, most likely due to a lack of time when participants were filling out the questionnaires. This missingness could not be considered Missing Completely At Random (MCAR), and was therefore considered Missing At Random (MAR), which makes it possible to impute the data for the questionnaires. The SSL, VPV and MSPSS were imputed at the item level using Multiple Imputation by Chained Equations (MICE). The imputation was carried out using the R package *mice* (1). For each questionnaire, 20 imputed datasets were created. The pooled estimates, following Rubin’s rules (2), are reported in the Results section.

The COVID-related stressors questionnaire could not be imputed because of the conditional nature of some of the questions, which makes that not all missingness in this questionnaire is MAR.

Statistical Analyses

Changes in Daily-Life Solitary Experiences from Pre-Pandemic to Early and Mid-Pandemic

To analyse the change in the proportion of time spent socially withdrawing, PA, NA, loneliness, finding it pleasant to be alone, wanting to be alone and feeling like an outsider from T0 to T1 and from T0 to T2, linear mixed-effects models with random intercepts were estimated using the *lme* function from the R package *nlme* (3). In each model, the binary variable timepoint was the predictor, and gender and age were added as covariates.

The Effect of the Amount of COVID-Related Stressors, the Mean Burdensomeness of COVID-Related Stressors and MSPSS-Score on Changes in Daily-Life Solitary Experiences from Pre-Pandemic to Early and Mid-Pandemic

In order to examine the effect of these variables on the proportion of time spent socially withdrawing, PA, NA, loneliness, finding it pleasant to be alone, wanting to be alone and feeling like an outsider, relative change scores were computed for each dependent variable at T1 and T2. For the proportion of time spent socially withdrawing, a person-level relative change score was computed, while for the moment-level variables, a momentary change score was computed relative to the T0-baseline level of the respective variable. Detailed information on how the relative change scores were computed is available in Additional File 2.

To assess the effect of the Number of COVID-Related Stressors, the Mean Burdensomeness of COVID-Related Stressors and MSPSS-score on the relative change in the proportion of time spent socially withdrawing at T1 and T2, regular linear regression models were estimated using the *lm* function of the R package *stats* (4). To assess the effect of the Number of COVID-Related Stressors, the Mean Burdensomeness of COVID-Related Stressors and MSPSS-score on the relative change in PA, NA, loneliness, finding it pleasant to be alone, wanting to be alone and feeling like an outsider, linear mixed-effects models with random intercepts were computed using the *lme* function of the R package *nlme* (3). Age and gender were added as covariates in all models.

The Effect of Solitude Cluster Membership on Daily-Life Solitary Experiences During the Early and Mid-Pandemic

In order to assess the effect of solitude cluster membership on the proportion of time spent socially withdrawing at T1 and T2, regular linear regression models were estimated using the *lm* function of the R package *stats* (4). To assess the effect of solitude cluster membership on PA, NA, loneliness, finding it pleasant to be alone, wanting to be alone and feeling like an outsider at T1 and T2, linear mixed-effect models with random intercepts were estimated using the *lme* function of the R package *nlme* (3). In all models, gender and age were added as covariates.

SSL- and VPV-Score as Moderators in the Changes in Daily-Life Solitary Experiences from Pre-Pandemic to Early and Mid-Pandemic

To examine the possible moderating effect of SSL- and VPV-score on changes in the proportion of time spent socially withdrawing, PA, NA, loneliness, finding it pleasant to be alone, wanting to be alone and feeling like an outsider from T0 to T1 and from T0 to T2, linear mixed-effects models with random intercepts were estimated using the *lme* function of the R package *nlme* (3). The main effect of interest were the interactions between SSL-score and Timepoint and VPV-score and Timepoint. In all models, age and gender were added as covariates.

References

1. van Buuren S, Groothuis-Oudshoorn K. mice: Multivariate Imputation by Chained Equations in R. J Stat Softw. 2011;45(3):1-67. Available from: https://doi.org/10.18637/jss.v045.i03.

2. Rubin DB. Multiple Imputation for Nonresponse in Surveys. Hoboken, NJ: John Wiley & Sons; 1987.

3. Pinheiro J, Bates D, R Core Team. nlme: Linear and nonlinear mixed effects models [Computer software]. R package version 3.1-153. 2022. Available from: https://cran.r-project.org/web/packages/nlme/index.html

4. R Core Team. R: A language and environment for statistical computing [Computer software]. Version 4.1.2. 2019. Available from: https://cran.r-project.org/
