# Supplementary material for: Changes in adolescents’ daily-life solitary experiences during the COVID-19 pandemic: an experience sampling study
Source: BMC Public Health. 2024 Apr 26;24:1172. doi: 10.1186/s12889-024-18458-1 (PMC11046767; doi:10.1186/s12889-024-18458-1)
Supplement: Supplementary file 7 — Supplementary Material 7 [file 12889_2024_18458_MOESM7_ESM.docx]

Additional File 4

Analysis code for manuscript ‘Changes in Adolescents’ Daily-Life Solitary Experiences During the COVID-19 Pandemic’

August 31st, 2022

Pre-Pandemic (T0) to Early-Pandemic (T1) Analyses

rm(list = ls(all.names = TRUE))

#######################################################################################################
##SETUP##

#Set seed
set.seed(1996)

#Set WD
setwd("C:/Users/u0131909/OneDrive - KU Leuven/Papers/COVID SIGMA/Analyses/Main analyses")

#Install and load packages
if (!suppressWarnings(require(remotes, quietly=TRUE)))
 install.packages("remotes")
if (!suppressWarnings(require(esmpack, quietly=TRUE)))
 remotes::install_github("wviechtb/esmpack")

pckgs <- c("esmpack", "dplyr", "tidyr", "psych", "nlme", "multilevelTools", "naniar", "apaTables", "GPArotation", "mitml", "lmerTest", "mice",
 "ggplot2", "ggsignif", "effects", "r2glmm", "cowplot")

lapply(pckgs, library, character.only = TRUE)

##
## Attaching package: 'dplyr'

## The following object is masked from 'package:esmpack':
##
## rename

## The following objects are masked from 'package:stats':
##
## filter, lag

## The following objects are masked from 'package:base':
##
## intersect, setdiff, setequal, union

##
## Attaching package: 'nlme'

## The following object is masked from 'package:dplyr':
##
## collapse

## *** This is beta software. Please report any bugs!
## *** See the NEWS file for recent changes.

## Loading required package: lme4

## Loading required package: Matrix

##
## Attaching package: 'Matrix'

## The following objects are masked from 'package:tidyr':
##
## expand, pack, unpack

##
## Attaching package: 'lme4'

## The following object is masked from 'package:nlme':
##
## lmList

##
## Attaching package: 'lmerTest'

## The following object is masked from 'package:lme4':
##
## lmer

## The following object is masked from 'package:stats':
##
## step

##
## Attaching package: 'mice'

## The following object is masked from 'package:stats':
##
## filter

## The following objects are masked from 'package:base':
##
## cbind, rbind

##
## Attaching package: 'ggplot2'

## The following objects are masked from 'package:psych':
##
## %+%, alpha

## Loading required package: carData

## lattice theme set by effectsTheme()
## See ?effectsTheme for details.

## [[1]]
## [1] "esmpack" "remotes" "rmarkdown" "stats" "graphics" "grDevices" "utils" "datasets" "methods"
## [10] "base"
##
## [[2]]
## [1] "dplyr" "esmpack" "remotes" "rmarkdown" "stats" "graphics" "grDevices" "utils" "datasets"
## [10] "methods" "base"
##
## [[3]]
## [1] "tidyr" "dplyr" "esmpack" "remotes" "rmarkdown" "stats" "graphics" "grDevices" "utils"
## [10] "datasets" "methods" "base"
##
## [[4]]
## [1] "psych" "tidyr" "dplyr" "esmpack" "remotes" "rmarkdown" "stats" "graphics" "grDevices"
## [10] "utils" "datasets" "methods" "base"
##
## [[5]]
## [1] "nlme" "psych" "tidyr" "dplyr" "esmpack" "remotes" "rmarkdown" "stats" "graphics"
## [10] "grDevices" "utils" "datasets" "methods" "base"
##
## [[6]]
## [1] "multilevelTools" "nlme" "psych" "tidyr" "dplyr" "esmpack"
## [7] "remotes" "rmarkdown" "stats" "graphics" "grDevices" "utils"
## [13] "datasets" "methods" "base"
##
## [[7]]
## [1] "naniar" "multilevelTools" "nlme" "psych" "tidyr" "dplyr"
## [7] "esmpack" "remotes" "rmarkdown" "stats" "graphics" "grDevices"
## [13] "utils" "datasets" "methods" "base"
##
## [[8]]
## [1] "apaTables" "naniar" "multilevelTools" "nlme" "psych" "tidyr"
## [7] "dplyr" "esmpack" "remotes" "rmarkdown" "stats" "graphics"
## [13] "grDevices" "utils" "datasets" "methods" "base"
##
## [[9]]
## [1] "GPArotation" "apaTables" "naniar" "multilevelTools" "nlme" "psych"
## [7] "tidyr" "dplyr" "esmpack" "remotes" "rmarkdown" "stats"
## [13] "graphics" "grDevices" "utils" "datasets" "methods" "base"
##
## [[10]]
## [1] "mitml" "GPArotation" "apaTables" "naniar" "multilevelTools" "nlme"
## [7] "psych" "tidyr" "dplyr" "esmpack" "remotes" "rmarkdown"
## [13] "stats" "graphics" "grDevices" "utils" "datasets" "methods"
## [19] "base"
##
## [[11]]
## [1] "lmerTest" "lme4" "Matrix" "mitml" "GPArotation" "apaTables"
## [7] "naniar" "multilevelTools" "nlme" "psych" "tidyr" "dplyr"
## [13] "esmpack" "remotes" "rmarkdown" "stats" "graphics" "grDevices"
## [19] "utils" "datasets" "methods" "base"
##
## [[12]]
## [1] "mice" "lmerTest" "lme4" "Matrix" "mitml" "GPArotation"
## [7] "apaTables" "naniar" "multilevelTools" "nlme" "psych" "tidyr"
## [13] "dplyr" "esmpack" "remotes" "rmarkdown" "stats" "graphics"
## [19] "grDevices" "utils" "datasets" "methods" "base"
##
## [[13]]
## [1] "ggplot2" "mice" "lmerTest" "lme4" "Matrix" "mitml"
## [7] "GPArotation" "apaTables" "naniar" "multilevelTools" "nlme" "psych"
## [13] "tidyr" "dplyr" "esmpack" "remotes" "rmarkdown" "stats"
## [19] "graphics" "grDevices" "utils" "datasets" "methods" "base"
##
## [[14]]
## [1] "ggsignif" "ggplot2" "mice" "lmerTest" "lme4" "Matrix"
## [7] "mitml" "GPArotation" "apaTables" "naniar" "multilevelTools" "nlme"
## [13] "psych" "tidyr" "dplyr" "esmpack" "remotes" "rmarkdown"
## [19] "stats" "graphics" "grDevices" "utils" "datasets" "methods"
## [25] "base"
##
## [[15]]
## [1] "effects" "carData" "ggsignif" "ggplot2" "mice" "lmerTest"
## [7] "lme4" "Matrix" "mitml" "GPArotation" "apaTables" "naniar"
## [13] "multilevelTools" "nlme" "psych" "tidyr" "dplyr" "esmpack"
## [19] "remotes" "rmarkdown" "stats" "graphics" "grDevices" "utils"
## [25] "datasets" "methods" "base"
##
## [[16]]
## [1] "r2glmm" "effects" "carData" "ggsignif" "ggplot2" "mice"
## [7] "lmerTest" "lme4" "Matrix" "mitml" "GPArotation" "apaTables"
## [13] "naniar" "multilevelTools" "nlme" "psych" "tidyr" "dplyr"
## [19] "esmpack" "remotes" "rmarkdown" "stats" "graphics" "grDevices"
## [25] "utils" "datasets" "methods" "base"
##
## [[17]]
## [1] "cowplot" "r2glmm" "effects" "carData" "ggsignif" "ggplot2"
## [7] "mice" "lmerTest" "lme4" "Matrix" "mitml" "GPArotation"
## [13] "apaTables" "naniar" "multilevelTools" "nlme" "psych" "tidyr"
## [19] "dplyr" "esmpack" "remotes" "rmarkdown" "stats" "graphics"
## [25] "grDevices" "utils" "datasets" "methods" "base"

#Read in data
dat <- read.csv("wave1covid110.csv", header = TRUE, sep = ";", na.strings = "")

#######################################################################################################
##DATA CLEANING##

#Remove (empty) RedCap variables + all esm_soc_who___ items except esm_soc_who___10 (= alone)
dat <- subset(dat, select = -c(redcap_event_.me, redcap_repeat_instance, redcap_repeat_instrument.y, redcap_repeat_instance.y, esm_soc_who___1, esm_soc_who___2,
 esm_soc_who___3, esm_soc_who___4, esm_soc_who___5, esm_soc_who___6, esm_soc_who___7, esm_soc_who___8, esm_soc_who___9, esm_soc_who___11))

#Add "obs" variable
dat <- dat %>%
 group_by(wave, record_id) %>%
 mutate(obs = row_number()-1) %>%
 ungroup()

#Spread person-level rows to beep level
dat <- dat %>%
 dplyr::group_by(wave, record_id) %>%
 tidyr::fill(c(agl_ado_age, agl_ado_sex, ssl_invite, ssl_visit, ssl_affection, ssl_comfort, ssl_compliment, ssl_interest, ssl_offerhelp, ssl_ease,
 ssl_advice, ssl_confidence, ssl_askhelp, ssl_positivecharacter, vpv_meetfriends, vpv_recogniseemotion, vpv_homework, vpv_disagreement,
 vpv_class, vpv_help, vpv_best, vpv_calm, vpv_easycontact, vpv_hurt, vpv_workleisure, vpv_polite, vpv_questions, vpv_sensitivities,
 vpv_attention, vpv_criticism, vpv_standup, vpv_said, vpv_money, vpv_think, vpv_seekcontact, vpv_wishes, vpv_order, vpv_lead, vpv_getalong,
 vpv_interest, vpv_dobest, vpv_calmcriticism, vpv_talkconcerns, vpv_adapt, vpv_usefulleisure, vpv_relax, vpv_goodcontact, vpv_learn,
 vpv_constant, vpv_adults,covid_symptoms_self, covid_symptoms_self_burden, covid_symptoms_family, covid_symptoms_family_burden,
 covid_risk_self, covid_risk_self_burden, covid_risk_family, covid_risk_family_burden, covid_job_self, covid_job_self_burden,
 covid_job_family, covid_job_family_burden, covid_healthcare, covid_healthcare_burden, covid_restriction, covid_restriction_burden,
 covid_lesspeople, covid_lesspeople_burden, covid_socialevents, covid_socialevents_burden, covid_hospital, covid_hospital_burden,
 covid_funeral, covid_funeral_burden, covid_hobby, covid_hobby_burden, covid_move, covid_move_burden, covid_tension_parents,
 covid_tension_parents_burden, covid_tension_self, covid_tension_self_burden, covid_siblings, covid_siblings_burden, covid_travel,
 covid_travel_burden, covid_basics, covid_basics_burden, covid_media, covid_media_burden, covid_school, covid_school_burden, covid_money,
 covid_money_burden, mspss_person_help, mspss_person_joy, mspss_family_help, mspss_family_support, mspss_person_comfort, mspss_friends_help,
 mspss_friends_count, mspss_family_talk, mspss_friends_joy, mspss_person_feelings, mspss_family_decide, mspss_friends_talk), .direction = "up" ) %>%
 ungroup()

#Remove rows with missingness (based on missingness in first ESM item)
dat <- subset(dat, is.na(esm_mood_cheerful)==0)

#Set values "88" as NA
dat <- dat %>%
 naniar::replace_with_na_all(condition = ~.x == 88)

#Calculate valid number of beeps per person - separately for T0 and T1
dat <- dat %>%
 group_by(wave, record_id) %>%
 mutate(bcount = length(obs)) %>%
 ungroup()

##VARIABLE CREATION##

#Momentary PA and NA
dat$momentPA <- combitems(c("esm_mood_cheerful", "esm_mood_relaxed", "esm_mood_satisfied", "esm_mood_generally_well"), dat)

## Computed 'mean' for these items: esm_mood_cheerful, esm_mood_relaxed, esm_mood_satisfied, esm_mood_generally_well.

dat$momentNA <- combitems(c("esm_mood_irritated", "esm_mood_anxious", "esm_mood_insecure", "esm_mood_paranoid", "esm_mood_sad", "esm_mood_stressed",
 "esm_mood_restless"), dat)

## Computed 'mean' for these items: esm_mood_irritated, esm_mood_anxious, esm_mood_insecure, esm_mood_paranoid, esm_mood_sad, esm_mood_stressed, esm_mood_restless.

# #Alternative if esmpack malfunctions
# dat$momentPA <- rowMeans(subset(dat,
# select=c("esm_mood_cheerful", "esm_mood_relaxed", "esm_mood_satisfied", "esm_mood_generally_well")), na.rm=T)
#
# dat$momentNA <- rowMeans(subset(dat,
# select=c("esm_mood_irritated", "esm_mood_anxious", "esm_mood_insecure", "esm_mood_paranoid", "esm_mood_sad", "esm_mood_stressed","esm_mood_restless")), na.rm=T)

#Within- and between-person reliability for PA and NA - separate for T0 and T1
omegaSEM(items = c("esm_mood_cheerful", "esm_mood_relaxed", "esm_mood_satisfied", "esm_mood_generally_well"), id = "record_id",
 data = dat[which(dat$wave == 'wave1'),])

## $Results
## label est ci.lower ci.upper
## 35 omega_within 0.768 0.755 0.781
## 42 omega_between 0.946 0.928 0.965

omegaSEM(items = c("esm_mood_irritated", "esm_mood_anxious", "esm_mood_insecure", "esm_mood_paranoid", "esm_mood_sad", "esm_mood_stressed",
 "esm_mood_restless"), id = "record_id", data = dat[which(dat$wave == "wave1"),])

## Warning in lav_data_full(data = data, group = group, cluster = cluster, : lavaan WARNING:
## Level-1 variable "esm_mood_irritated" has no variance within some
## clusters. The cluster ids with zero within variance are: 1636 2416
## 2567

## Warning in lav_data_full(data = data, group = group, cluster = cluster, : lavaan WARNING:
## Level-1 variable "esm_mood_anxious" has no variance within some
## clusters. The cluster ids with zero within variance are: 1053 1217
## 1273 1289 1340 1636 1872 1976 2140 2178 2228 2234 2416 2524 2567

## Warning in lav_data_full(data = data, group = group, cluster = cluster, : lavaan WARNING:
## Level-1 variable "esm_mood_insecure" has no variance within some
## clusters. The cluster ids with zero within variance are: 1289 1340
## 1976 2567 2955

## Warning in lav_data_full(data = data, group = group, cluster = cluster, : lavaan WARNING:
## Level-1 variable "esm_mood_paranoid" has no variance within some
## clusters. The cluster ids with zero within variance are: 1053 1054
## 1079 1110 1289 1636 1738 1976 2187 2271

## Warning in lav_data_full(data = data, group = group, cluster = cluster, : lavaan WARNING:
## Level-1 variable "esm_mood_sad" has no variance within some
## clusters. The cluster ids with zero within variance are: 1008 1053
## 1143 1289 1693 1976 2140 2187 2271

## Warning in lav_data_full(data = data, group = group, cluster = cluster, : lavaan WARNING:
## Level-1 variable "esm_mood_stressed" has no variance within some
## clusters. The cluster ids with zero within variance are: 1693 1976

## Warning in lav_data_full(data = data, group = group, cluster = cluster, : lavaan WARNING:
## Level-1 variable "esm_mood_restless" has no variance within some
## clusters. The cluster ids with zero within variance are: 1053 1079
## 1110 1738 1976 2178 2187 2524 2908

## $Results
## label est ci.lower ci.upper
## 56 omega_within 0.726 0.712 0.740
## 66 omega_between 0.925 0.902 0.948

omegaSEM(items = c("esm_mood_cheerful", "esm_mood_relaxed", "esm_mood_satisfied", "esm_mood_generally_well"), id = "record_id",
 data = dat[which(dat$wave == "covid"),])

## Warning in lav_data_full(data = data, group = group, cluster = cluster, : lavaan WARNING:
## Level-1 variable "esm_mood_cheerful" has no variance within some
## clusters. The cluster ids with zero within variance are: 1340 2325
## 2883

## Warning in lav_data_full(data = data, group = group, cluster = cluster, : lavaan WARNING:
## Level-1 variable "esm_mood_relaxed" has no variance within some
## clusters. The cluster ids with zero within variance are: 2325 2883

## Warning in lav_data_full(data = data, group = group, cluster = cluster, : lavaan WARNING:
## Level-1 variable "esm_mood_satisfied" has no variance within some
## clusters. The cluster ids with zero within variance are: 1097 2325
## 2883

## Warning in lav_data_full(data = data, group = group, cluster = cluster, : lavaan WARNING:
## Level-1 variable "esm_mood_generally_well" has no variance within
## some clusters. The cluster ids with zero within variance are: 1133
## 2325 2883

## $Results
## label est ci.lower ci.upper
## 35 omega_within 0.819 0.808 0.830
## 42 omega_between 0.974 0.964 0.983

omegaSEM(items = c("esm_mood_irritated", "esm_mood_anxious", "esm_mood_insecure", "esm_mood_paranoid", "esm_mood_sad", "esm_mood_stressed",
 "esm_mood_restless"), id = "record_id", data = dat[which(dat$wave == "covid"),])

## Warning in lav_data_full(data = data, group = group, cluster = cluster, : lavaan WARNING:
## Level-1 variable "esm_mood_irritated" has no variance within some
## clusters. The cluster ids with zero within variance are: 1340 1636
## 2325

## Warning in lav_data_full(data = data, group = group, cluster = cluster, : lavaan WARNING:
## Level-1 variable "esm_mood_anxious" has no variance within some
## clusters. The cluster ids with zero within variance are: 1008 1037
## 1054 1110 1289 1315 1340 1407 1418 1516 1636 1738 1811 1831 1961
## 1976 2032 2140 2178 2234 2247 2271 2281 2567 2865 2883 2890 3156
## 3211 3215 3231

## Warning in lav_data_full(data = data, group = group, cluster = cluster, : lavaan WARNING:
## Level-1 variable "esm_mood_insecure" has no variance within some
## clusters. The cluster ids with zero within variance are: 1037 1046
## 1315 1418 1636 1738 1872 1976 2228 2883 3156 3211 3231

## Warning in lav_data_full(data = data, group = group, cluster = cluster, : lavaan WARNING:
## Level-1 variable "esm_mood_paranoid" has no variance within some
## clusters. The cluster ids with zero within variance are: 1037 1046
## 1053 1054 1110 1143 1217 1289 1340 1418 1516 1636 1738 1960 1976
## 2032 2178 2187 2234 2271 2281 2416 2567 2793 2865 2883 2890 3211
## 3215 3231

## Warning in lav_data_full(data = data, group = group, cluster = cluster, : lavaan WARNING:
## Level-1 variable "esm_mood_sad" has no variance within some
## clusters. The cluster ids with zero within variance are: 1110 1133
## 1636 1738 1835 1872 1976 2012 2140 2281 2567 2883 3231

## Warning in lav_data_full(data = data, group = group, cluster = cluster, : lavaan WARNING:
## Level-1 variable "esm_mood_stressed" has no variance within some
## clusters. The cluster ids with zero within variance are: 1037 1636
## 2281 2883

## Warning in lav_data_full(data = data, group = group, cluster = cluster, : lavaan WARNING:
## Level-1 variable "esm_mood_restless" has no variance within some
## clusters. The cluster ids with zero within variance are: 1008 1037
## 1110 1340 1407 1636 1738 1872 2281 2793 2865 2883 3156 3211 3215

## $Results
## label est ci.lower ci.upper
## 56 omega_within 0.782 0.769 0.794
## 66 omega_between 0.968 0.958 0.977

#Proportion of time spent alone
#Make alone count variable
dat <- dat %>%
 group_by(wave, record_id) %>%
 mutate(alone_count = sum(esm_soc_who___10))%>%
 ungroup()

#Make proportion alone variable
dat$alone_prop <- dat$alone_count/dat$bcount

#Turn sex into a binary variable (by omitting participants who indicated gender = other)
dat <- subset(dat, agl_ado_sex != 2)
dat$sex_ismale <- dummy.code(dat$agl_ado_sex, group = 0)

#Grand-mean center age - separate for each wave
agedf <- dat %>%
 group_by(wave, record_id) %>%
 summarise(age=mean(dat$agl_ado_age, na.rm = TRUE))

## `summarise()` has grouped output by 'wave'. You can override using the `.groups` argument.

agemean <- mean(agedf$age)

dat$c_age <- dat$agl_ado_age - agemean

#Select beeps when participants are alone - only these beeps will be taken into account for the analyses
dat <- subset(dat, esm_soc_who___10==1)

#Create new obs variable - original obs variable has gaps because we selected only the rows for which esm_soc_who___10==1
n.i = unique(dat$record_id)
dat$obs = rep(0,nrow(dat))
for (i in n.i){
 dat$obs[which(dat$record_id==i)] = seq(1:length(which(dat$record_id==i)))
}

#Create bcount_alone variable
dat <- dat %>%
 group_by(wave, record_id) %>%
 mutate(bcount_alone = length(obs)) %>%
 ungroup()

######################################################################################################
##PERSON-LEVEL DATASET##

#Make a person-level dataset
PLdat <- dat %>%
 group_by(record_id, wave) %>%
 summarize(sex = first(as.factor(sex_ismale)),
 age = mean(agl_ado_age, na.rm=T),
 bcount = mean(bcount, na.rm=T),
 bcount_alone = mean(bcount_alone, na.rm=T),
 ssl_invite = mean(ssl_invite, na.rm=T), # SSL items
 ssl_visit = mean(ssl_visit, na.rm=T),
 ssl_affection = mean(ssl_affection, na.rm=T),
 ssl_comfort = mean(ssl_comfort, na.rm=T),
 ssl_compliment = mean(ssl_compliment, na.rm=T),
 ssl_interest = mean(ssl_interest, na.rm=T),
 ssl_offerhelp = mean(ssl_offerhelp, na.rm=T),
 ssl_ease = mean(ssl_ease, na.rm=T),
 ssl_advice = mean(ssl_advice, na.rm=T),
 ssl_confidence = mean(ssl_confidence, na.rm=T),
 ssl_askhelp = mean(ssl_askhelp, na.rm=T),
 ssl_positivecharacter = mean(ssl_positivecharacter, na.rm=T),
 vpv_meetfriends = mean(vpv_meetfriends, na.rm=T), # For the VPV, we only select items here from the interpersonal subscale
 vpv_recogniseemotion = mean(vpv_recogniseemotion, na.rm=T),
 vpv_class = mean(vpv_class, na.rm=T),
 vpv_help = mean(vpv_help, na.rm=T),
 vpv_easycontact = mean(vpv_easycontact, na.rm=T),
 vpv_hurt = mean(vpv_hurt, na.rm=T),
 vpv_questions = mean(vpv_questions, na.rm=T),
 vpv_sensitivities = mean(vpv_sensitivities, na.rm=T),
 vpv_standup = mean(vpv_standup, na.rm=T),
 vpv_said = mean(vpv_said, na.rm=T),
 vpv_seekcontact = mean(vpv_seekcontact, na.rm=T),
 vpv_wishes = mean(vpv_wishes, na.rm=T),
 vpv_getalong = mean(vpv_getalong, na.rm=T),
 vpv_interest = mean(vpv_interest, na.rm=T),
 vpv_talkconcerns = mean(vpv_talkconcerns, na.rm=T),
 vpv_adapt = mean(vpv_adapt, na.rm=T),
 vpv_goodcontact = mean(vpv_goodcontact, na.rm=T),
 vpv_learn = mean(vpv_learn, na.rm=T),
 covid_symptoms_self = mean(covid_symptoms_self, na.rm=T), #COVID stressors
 covid_symptoms_self_burden = mean(covid_symptoms_self_burden, na.rm=T),
 covid_symptoms_family = mean(covid_symptoms_family, na.rm=T),
 covid_symptoms_family_burden = mean(covid_symptoms_family_burden, na.rm=T),
 covid_risk_self = mean(covid_risk_self, na.rm=T),
 covid_risk_self_burden = mean(covid_risk_self_burden, na.rm=T),
 covid_risk_family = mean(covid_risk_family, na.rm=T),
 covid_risk_family_burden = mean(covid_risk_family_burden, na.rm=T),
 covid_job_family = mean(covid_job_family, na.rm=T),
 covid_job_family_burden = mean(covid_job_family_burden, na.rm=T),
 covid_job_self = mean(covid_job_self, na.rm=T),
 covid_job_self_burden = mean(covid_job_self_burden, na.rm=T),
 covid_healthcare = mean(covid_healthcare, na.rm=T),
 covid_healthcare_burden = mean(covid_healthcare_burden, na.rm=T),
 covid_restriction = mean(covid_restriction, na.rm=T),
 covid_restriction_burden = mean(covid_restriction_burden, na.rm=T),
 covid_lesspeople = mean(covid_lesspeople, na.rm=T),
 covid_lesspeople_burden = mean(covid_lesspeople_burden, na.rm=T),
 covid_socialevents = mean(covid_socialevents, na.rm=T),
 covid_socialevents_burden = mean(covid_socialevents_burden, na.rm=T),
 covid_hospital = mean(covid_hospital, na.rm=T),
 covid_hospital_burden = mean(covid_hospital_burden, na.rm=T),
 covid_funeral = mean(covid_funeral, na.rm=T),
 covid_funeral_burden = mean(covid_funeral_burden, na.rm=T),
 covid_hobby = mean(covid_hobby, na.rm=T),
 covid_hobby_burden = mean(covid_hobby_burden, na.rm=T),
 covid_move = mean(covid_move, na.rm=T),
 covid_move_burden = mean(covid_move_burden, na.rm=T),
 covid_tension_parents = mean(covid_tension_parents, na.rm=T),
 covid_tension_parents_burden = mean(covid_tension_parents_burden, na.rm=T),
 covid_tension_self = mean(covid_tension_self, na.rm=T),
 covid_tension_self_burden = mean(covid_tension_self_burden, na.rm=T),
 covid_siblings = mean(covid_siblings, na.rm=T),
 covid_siblings_burden = mean(covid_siblings_burden, na.rm=T),
 covid_travel = mean(covid_travel, na.rm=T),
 covid_travel_burden = mean(covid_travel_burden, na.rm=T),
 covid_basics = mean(covid_basics, na.rm=T),
 covid_basics_burden = mean(covid_basics_burden, na.rm=T),
 covid_media = mean(covid_media, na.rm=T),
 covid_media_burden = mean(covid_media_burden, na.rm=T),
 covid_school = mean(covid_school, na.rm=T),
 covid_school_burden = mean(covid_school_burden, na.rm=T),
 covid_money = mean(covid_money, na.rm=T),
 covid_money_burden = mean(covid_money_burden, na.rm=T),
 mspss_person_help = mean(mspss_person_help, na.rm=T), #T1 social support
 mspss_person_joy = mean(mspss_person_joy, na.rm=T),
 mspss_family_help = mean(mspss_family_help, na.rm=T),
 mspss_family_support = mean(mspss_family_support, na.rm=T),
 mspss_person_comfort = mean(mspss_person_comfort, na.rm=T),
 mspss_friends_help = mean(mspss_friends_help, na.rm=T),
 mspss_friends_count = mean(mspss_friends_count, na.rm=T),
 mspss_family_talk = mean(mspss_family_talk, na.rm=T),
 mspss_friends_joy = mean(mspss_friends_joy, na.rm=T),
 mspss_person_feelings = mean(mspss_person_feelings, na.rm=T),
 mspss_family_decide = mean(mspss_family_decide, na.rm=T),
 mspss_friends_talk = mean(mspss_friends_talk, na.rm=T),
 PL_momentPA = mean(momentPA, na.rm=T),
 PL_momentNA = mean(momentNA, na.rm=T),
 PL_loneliness = mean(esm_mood_lonely, na.rm=T),
 PL_pleasant = mean(esm_soc_alone_pleasant, na.rm=T),
 PL_excluded = mean(esm_soc_alone_excluded, na.rm=T),
 PL_want = mean(esm_soc_alone_want, na.rm=T),
 PL_aloneprop = mean(alone_prop, na.rm=T)) %>%
 ungroup()

## `summarise()` has grouped output by 'record_id'. You can override using the `.groups` argument.

#Set values "NaN" as NA
PLdat <- PLdat %>%
 mutate_all(~ifelse(is.nan(.), NA, .))

#Resetting the reference for study
dat$wave.f <- as.factor(dat$wave)
dat$wave.f <- relevel(dat$wave.f, ref="wave1")
PLdat$wave.f <- as.factor(PLdat$wave)
PLdat$wave.f <- relevel(PLdat$wave.f, ref="wave1")

T0 = which(PLdat$wave == "wave1")
T1 = which(PLdat$wave == "covid")

#Create total scores for questionnaires
#SSL - social support at T0
ssldata <- select(PLdat, record_id, starts_with("ssl"))
colnames(ssldata)

## [1] "record_id" "ssl_invite" "ssl_visit" "ssl_affection"
## [5] "ssl_comfort" "ssl_compliment" "ssl_interest" "ssl_offerhelp"
## [9] "ssl_ease" "ssl_advice" "ssl_confidence" "ssl_askhelp"
## [13] "ssl_positivecharacter"

total <- c("ssl_compliment", "ssl_confidence", "ssl_askhelp", "ssl_positivecharacter", "ssl_comfort", "ssl_offerhelp", "ssl_ease", "ssl_advice", "ssl_invite", "ssl_visit", "ssl_affection", "ssl_interest")

ssldata$ssl_total_miss <- apply(ssldata[,total], 1, function(x) sum(is.na(x)))
ssldata$ssl_total_mean_temp <- ifelse(ssldata$ssl_total_miss <= 4, rowMeans(ssldata[,total], na.rm = TRUE), NA)

ssldata$ssl_total_sum <- ifelse(ssldata$ssl_total_miss<=4, rowSums(ssldata[, total], na.rm = TRUE), NA)
ssldata$ssl_total_total <- ifelse(ssldata$ssl_total_miss<=4, ssldata$ssl_total_sum+(ssldata$ssl_total_miss*ssldata$ssl_total_mean_temp), ssldata$ssl_total_sum)
PLdat$ssl_total <- ssldata$ssl_total_total

#VPV - social skills at T0
vpvdata <- select(PLdat, record_id, starts_with("vpv"))
colnames(vpvdata)

## [1] "record_id" "vpv_meetfriends" "vpv_recogniseemotion" "vpv_class" "vpv_help"
## [6] "vpv_easycontact" "vpv_hurt" "vpv_questions" "vpv_sensitivities" "vpv_standup"
## [11] "vpv_said" "vpv_seekcontact" "vpv_wishes" "vpv_getalong" "vpv_interest"
## [16] "vpv_talkconcerns" "vpv_adapt" "vpv_goodcontact" "vpv_learn"

vpvdata$vpv_miss <- apply(vpvdata[,2:19], 1, function(x) sum(is.na(x)))
table(vpvdata$vpv_miss)

##
## 0 1 2 18
## 90 2 1 104

vpv_r <- c("vpv_meetfriends", "vpv_class", "vpv_easycontact", "vpv_questions", "vpv_standup", "vpv_seekcontact", "vpv_getalong", "vpv_talkconcerns", "vpv_goodcontact")
PLdat$vpv_r <- ifelse(vpvdata$vpv_miss==0, rowSums(vpvdata[, vpv_r]), NA)
vpv_a <- c("vpv_recogniseemotion", "vpv_help", "vpv_hurt", "vpv_sensitivities", "vpv_said", "vpv_wishes", "vpv_learn", "vpv_interest", "vpv_adapt")
PLdat$vpv_a <- ifelse(vpvdata$vpv_miss==0, rowSums(vpvdata[, vpv_a]), NA)
PLdat$vpv_interpers <- PLdat$vpv_a+PLdat$vpv_r

#Amount of COVID-related events
PLdat$stressors_amount <- PLdat$covid_symptoms_self + PLdat$covid_symptoms_family + PLdat$covid_risk_self + PLdat$covid_risk_family +
 PLdat$covid_healthcare + PLdat$covid_job_family + PLdat$covid_job_self + PLdat$covid_restriction + PLdat$covid_lesspeople +
 PLdat$covid_socialevents + PLdat$covid_hospital + PLdat$covid_funeral + PLdat$covid_hobby + PLdat$covid_move + PLdat$covid_tension_parents +
 PLdat$covid_tension_self + PLdat$covid_siblings + PLdat$covid_travel + PLdat$covid_basics + PLdat$covid_media + PLdat$covid_school + PLdat$covid_money

#Mean burdensomeness of COVID-related events
PLdat <- PLdat %>%
 group_by(wave, record_id) %>%
 mutate(stressors_burden = mean(c(covid_symptoms_self_burden, covid_symptoms_family_burden, covid_risk_self_burden,
 covid_risk_family_burden, covid_healthcare_burden, covid_job_family_burden, covid_job_self_burden,
 covid_restriction_burden, covid_lesspeople_burden, covid_socialevents_burden, covid_hospital_burden,
 covid_funeral_burden, covid_hobby_burden, covid_move_burden, covid_tension_parents_burden,
 covid_tension_self_burden, covid_siblings_burden, covid_travel_burden, covid_basics_burden,
 covid_media_burden, covid_school_burden, covid_money_burden), na.rm = TRUE)) %>%
 mutate_all(~ifelse(is.nan(.), NA, .)) %>%
 ungroup()

## `mutate_all()` ignored the following grouping variables:
## • Columns `wave`, `record_id`
## ℹ Use `mutate_at(df, vars(-group_cols()), myoperation)` to silence the message.

#MSPSS - social support at T1
PLdat$mspss_total <- PLdat$mspss_person_help + PLdat$mspss_person_joy + PLdat$mspss_family_help + PLdat$mspss_family_support +
 PLdat$mspss_person_comfort + PLdat$mspss_friends_help + PLdat$mspss_friends_count + PLdat$mspss_family_talk +
 PLdat$mspss_friends_joy + PLdat$mspss_person_feelings + PLdat$mspss_family_decide + PLdat$mspss_friends_talk

#Add T0 cluster membership to this dataset
#The cluster membership is calculated with code from the file "analyses_code_DLsocialwithdrawal" available on the OSF project page of the
#study "Identifying clusters of adolescents based on their daily-life social withdrawal experience" (https://osf.io/7htwv/?view_only=7fa5907478364f369766f51aa19041c4)
wave1clst <- read.csv("Wave1_Clustermeans.csv", header = TRUE, sep = ",")
rcrdid <- unique(PLdat$record_id)
clstdat <- wave1clst[which(wave1clst$record_id %in% rcrdid == TRUE),]
PLdat <- left_join(x = PLdat, y = wave1clst)

## Joining, by = "record_id"

#Grand-mean center age
PLdat$c_age <- PLdat$age - mean(PLdat$age, na.rm=T)

##DESCRIPTIVES##

#Calculate ESM compliance - not separate for T0 and T1, as both had a maximum of 60 beeps
PLdat$compliance_prop <- PLdat$bcount / 60

#Dependent variable descriptives
psych::describe(PLdat[T0, c("PL_momentPA", "PL_momentNA", "PL_pleasant", "PL_excluded", "PL_want", "PL_aloneprop", "PL_loneliness")])

## vars n mean sd median trimmed mad min max range skew kurtosis se
## PL_momentPA 1 97 4.98 1.17 5.00 5.05 1.11 1.00 7.00 6.00 -0.80 1.04 0.12
## PL_momentNA 2 97 2.08 1.00 1.86 1.95 0.87 1.00 6.17 5.17 1.50 2.93 0.10
## PL_pleasant 3 97 5.25 1.61 5.50 5.46 1.63 1.00 7.00 6.00 -0.94 0.15 0.16
## PL_excluded 4 97 1.43 0.98 1.00 1.20 0.00 1.00 7.00 6.00 3.57 14.61 0.10
## PL_want 5 97 4.62 1.81 5.00 4.75 1.98 1.00 7.00 6.00 -0.47 -0.91 0.18
## PL_aloneprop 6 97 0.20 0.14 0.19 0.19 0.14 0.02 0.71 0.70 0.91 0.72 0.01
## PL_loneliness 7 97 2.08 1.42 1.57 1.81 0.85 1.00 7.00 6.00 1.74 2.99 0.14

psych::describe(PLdat[T1, c("PL_momentPA", "PL_momentNA", "PL_pleasant", "PL_excluded", "PL_want", "PL_aloneprop", "PL_loneliness")])

## vars n mean sd median trimmed mad min max range skew kurtosis se
## PL_momentPA 1 100 4.96 1.08 5.00 5.02 0.97 1.00 6.95 5.95 -0.63 0.73 0.11
## PL_momentNA 2 100 2.16 1.15 1.72 1.98 0.80 1.00 7.00 6.00 1.59 2.84 0.11
## PL_pleasant 3 100 5.23 1.30 5.42 5.34 1.23 1.00 7.00 6.00 -0.87 0.71 0.13
## PL_excluded 4 100 1.44 0.79 1.00 1.24 0.00 1.00 4.50 3.50 2.05 3.36 0.08
## PL_want 5 100 4.79 1.44 4.95 4.88 1.41 1.00 7.00 6.00 -0.46 -0.17 0.14
## PL_aloneprop 6 100 0.42 0.21 0.41 0.42 0.23 0.02 1.00 0.98 0.22 -0.57 0.02
## PL_loneliness 7 100 2.07 1.39 1.44 1.80 0.65 1.00 7.00 6.00 1.66 2.45 0.14

boxplot(PL_momentPA ~ wave, dat=PLdat)


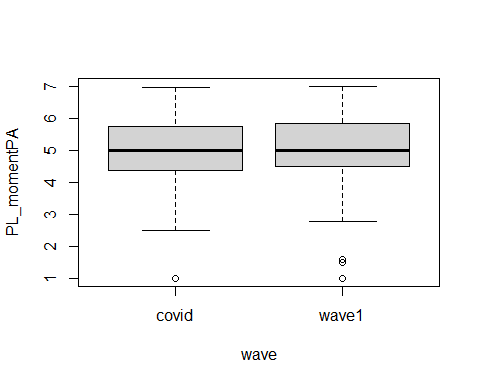


boxplot(PL_momentNA ~ wave, dat=PLdat)


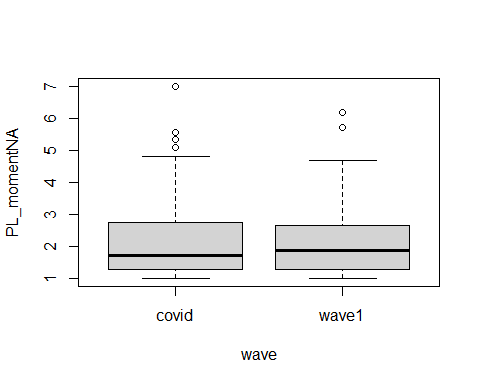


boxplot(PL_pleasant ~ wave, dat=PLdat)


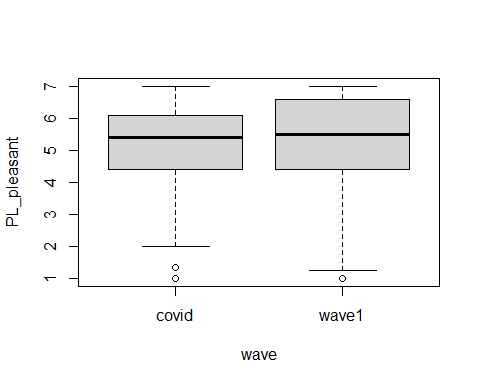


boxplot(PL_excluded ~ wave, dat=PLdat)


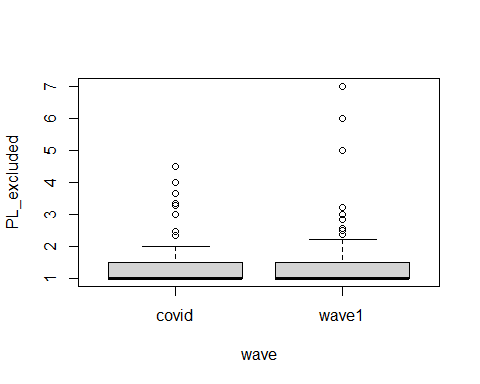


boxplot(PL_want ~ wave, dat=PLdat)


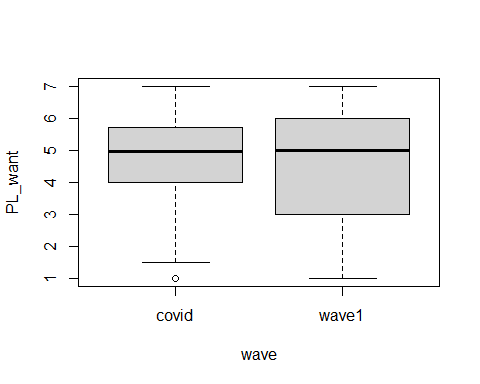


boxplot(PL_aloneprop ~ wave, dat=PLdat)


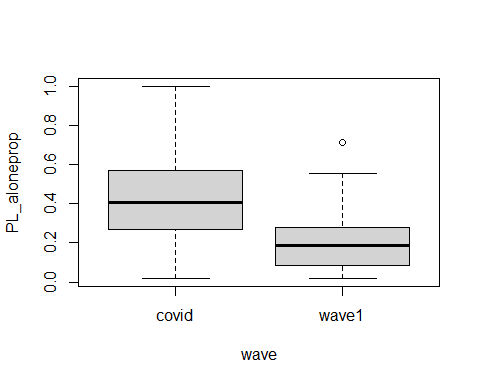


boxplot(PL_loneliness ~ wave, dat=PLdat)


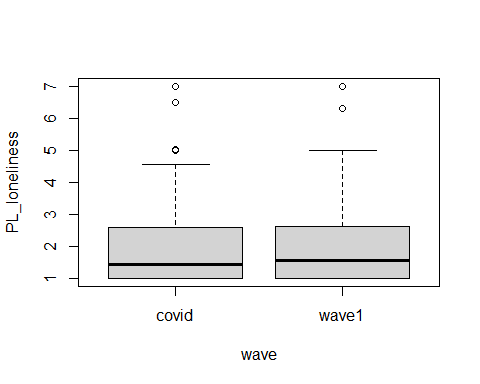


#Moderator descriptives
psych::describe(PLdat[T0, c("vpv_interpers", "ssl_total")])

## vars n mean sd median trimmed mad min max range skew kurtosis se
## vpv_interpers 1 90 69.28 7.75 69 69.12 7.41 54 90 36 0.18 -0.17 0.82
## ssl_total 2 93 22.21 6.42 21 22.13 7.41 5 36 31 0.08 -0.52 0.67

table(PLdat$groupMean[T0], useNA = "always")

##
## 0 1 <NA>
## 72 25 0

psych::describe(PLdat[T1, c("stressors_amount", "stressors_burden", "mspss_total")])

## vars n mean sd median trimmed mad min max range skew kurtosis se
## stressors_amount 1 100 10.31 2.62 11.00 10.38 2.97 5.00 16.00 11.00 -0.20 -0.77 0.26
## stressors_burden 2 100 3.20 0.48 3.18 3.19 0.49 2.12 4.69 2.57 0.22 0.10 0.05
## mspss_total 3 99 58.71 14.79 60.00 59.20 16.31 19.00 84.00 65.00 -0.33 -0.50 1.49

#Covariates + compliance descriptives
psych::describe(PLdat[T0, c("age", "compliance_prop")])

## vars n mean sd median trimmed mad min max range skew kurtosis se
## age 1 97 14.31 1.82 14.00 14.23 2.97 11.0 18.00 7.00 0.17 -1.13 0.18
## compliance_prop 2 97 0.58 0.20 0.58 0.58 0.25 0.2 0.98 0.78 -0.15 -1.09 0.02

table(PLdat$sex[T0], useNA = "always")

##
## 1 2 <NA>
## 89 8 0

psych::describe(PLdat[T1, c("age", "compliance_prop")])

## vars n mean sd median trimmed mad min max range skew kurtosis se
## age 1 100 16.14 1.92 16.00 16.12 2.97 13.00 20.00 7.00 0.06 -1.04 0.19
## compliance_prop 2 100 0.48 0.26 0.46 0.48 0.31 0.03 0.98 0.95 0.13 -1.09 0.03

table(PLdat$sex[T1], useNA = "always")

##
## 1 2 <NA>
## 93 7 0

#Correlation table
CorDatT0 <- subset(PLdat[T0, c("PL_momentPA", "PL_momentNA", "PL_loneliness", "PL_pleasant", "PL_excluded", "PL_want", "PL_aloneprop",
 "vpv_interpers", "ssl_total", "age")])
apa.cor.table(CorDatT0, filename = "COVID_cortable_T0.doc", table.number = 2)

##
##
## Table 2
##
## Means, standard deviations, and correlations with confidence intervals
##
##
## Variable M SD 1 2 3 4 5 6 7
## 1. PL_momentPA 4.98 1.17
##
## 2. PL_momentNA 2.08 1.00 -.74**
## [-.82, -.64]
##
## 3. PL_loneliness 2.08 1.42 -.45** .62**
## [-.60, -.28] [.49, .73]
##
## 4. PL_pleasant 5.25 1.61 .19 -.18 -.36**
## [-.01, .37] [-.37, .02] [-.53, -.18]
##
## 5. PL_excluded 1.43 0.98 -.41** .46** .52** -.26*
## [-.56, -.23] [.28, .60] [.36, .65] [-.43, -.06]
##
## 6. PL_want 4.62 1.81 -.12 .10 -.11 .65** .01
## [-.31, .08] [-.11, .29] [-.31, .09] [.51, .75] [-.19, .20]
##
## 7. PL_aloneprop 0.20 0.14 -.25* .11 .03 .07 -.06 .10
## [-.43, -.06] [-.10, .30] [-.18, .22] [-.13, .27] [-.26, .14] [-.10, .29]
##
## 8. vpv_interpers 69.28 7.75 .35** -.28** -.16 .08 -.14 .04 -.17
## [.16, .52] [-.46, -.08] [-.35, .05] [-.13, .28] [-.34, .07] [-.17, .24] [-.36, .04]
##
## 9. ssl_total 22.21 6.42 .27** -.26* -.28** .04 -.19 -.05 .01
## [.07, .45] [-.44, -.06] [-.46, -.08] [-.16, .24] [-.38, .01] [-.25, .16] [-.19, .21]
##
## 10. age 14.31 1.82 -.35** .22* .08 .07 .05 .09 .26*
## [-.52, -.17] [.02, .40] [-.12, .28] [-.13, .27] [-.15, .25] [-.11, .29] [.06, .43]
##
## 8 9
##
##
##
##
##
##
##
##
##
##
##
##
##
##
##
##
##
##
##
##
##
##
##
## .60**
## [.44, .71]
##
## -.11 -.06
## [-.31, .10] [-.26, .15]
##
##
## Note. M and SD are used to represent mean and standard deviation, respectively.
## Values in square brackets indicate the 95% confidence interval.
## The confidence interval is a plausible range of population correlations
## that could have caused the sample correlation (Cumming, 2014).
## * indicates p < .05. ** indicates p < .01.
##

CorDatT1 <- subset(PLdat[T1, c("PL_momentPA", "PL_momentNA", "PL_loneliness", "PL_pleasant", "PL_excluded", "PL_want", "PL_aloneprop",
 "stressors_amount", "stressors_burden", "mspss_total", "age")])
apa.cor.table(CorDatT1, filename = "COVID_cortableT1.doc", table.number = 3)

##
##
## Table 3
##
## Means, standard deviations, and correlations with confidence intervals
##
##
## Variable M SD 1 2 3 4 5 6
## 1. PL_momentPA 4.96 1.08
##
## 2. PL_momentNA 2.16 1.15 -.73**
## [-.81, -.62]
##
## 3. PL_loneliness 2.07 1.39 -.62** .74**
## [-.73, -.49] [.63, .82]
##
## 4. PL_pleasant 5.23 1.30 .32** -.24* -.45**
## [.13, .48] [-.42, -.05] [-.59, -.27]
##
## 5. PL_excluded 1.44 0.79 -.59** .70** .72** -.26**
## [-.71, -.45] [.58, .79] [.61, .80] [-.43, -.06]
##
## 6. PL_want 4.79 1.44 .19 -.09 -.31** .84** -.16
## [-.01, .37] [-.28, .11] [-.48, -.12] [.77, .89] [-.34, .04]
##
## 7. PL_aloneprop 0.42 0.21 -.19 .08 .21* -.17 .20 -.06
## [-.37, .01] [-.12, .27] [.02, .39] [-.35, .03] [-.00, .38] [-.26, .13]
##
## 8. stressors_amount 10.31 2.62 -.36** .34** .36** -.10 .23* -.11
## [-.52, -.18] [.16, .50] [.17, .52] [-.29, .10] [.04, .41] [-.30, .09]
##
## 9. stressors_burden 3.20 0.48 -.38** .35** .37** -.20* .20* -.11
## [-.54, -.20] [.17, .52] [.19, .53] [-.39, -.01] [.00, .38] [-.30, .09]
##
## 10. mspss_total 58.71 14.79 .29** -.14 -.16 -.13 -.27** -.12
## [.10, .46] [-.32, .06] [-.35, .04] [-.32, .07] [-.45, -.08] [-.31, .08]
##
## 11. age 16.14 1.92 -.37** .11 .02 -.24* .08 -.27**
## [-.53, -.19] [-.09, .30] [-.17, .22] [-.42, -.05] [-.12, .27] [-.45, -.08]
##
## 7 8 9 10
##
##
##
##
##
##
##
##
##
##
##
##
##
##
##
##
##
##
##
##
## .06
## [-.14, .25]
##
## .10 .21*
## [-.10, .29] [.02, .39]
##
## -.11 -.08 -.11
## [-.30, .09] [-.27, .12] [-.30, .09]
##
## .03 .02 .18 -.12
## [-.16, .23] [-.18, .21] [-.02, .36] [-.31, .08]
##
##
## Note. M and SD are used to represent mean and standard deviation, respectively.
## Values in square brackets indicate the 95% confidence interval.
## The confidence interval is a plausible range of population correlations
## that could have caused the sample correlation (Cumming, 2014).
## * indicates p < .05. ** indicates p < .01.
##

#Reliability of person-level questionnaires
ssldat <- PLdat[T0, c("ssl_invite", "ssl_visit", "ssl_affection", "ssl_comfort", "ssl_compliment", "ssl_interest", "ssl_offerhelp",
 "ssl_ease", "ssl_advice", "ssl_confidence", "ssl_askhelp")]
psych::alpha(ssldat, na.rm=T)

##
## Reliability analysis
## Call: psych::alpha(x = ssldat, na.rm = T)
##
## raw_alpha std.alpha G6(smc) average_r S/N ase mean sd median_r
## 0.86 0.86 0.88 0.36 6.2 0.021 1.9 0.53 0.35
##
## 95% confidence boundaries
## lower alpha upper
## Feldt 0.81 0.86 0.9
## Duhachek 0.82 0.86 0.9
##
## Reliability if an item is dropped:
## raw_alpha std.alpha G6(smc) average_r S/N alpha se var.r med.r
## ssl_invite 0.86 0.86 0.87 0.38 6.1 0.022 0.0094 0.38
## ssl_visit 0.85 0.85 0.86 0.36 5.7 0.023 0.0113 0.35
## ssl_affection 0.85 0.85 0.86 0.36 5.6 0.023 0.0116 0.35
## ssl_comfort 0.85 0.85 0.86 0.36 5.6 0.023 0.0121 0.34
## ssl_compliment 0.84 0.85 0.86 0.35 5.5 0.024 0.0121 0.32
## ssl_interest 0.85 0.85 0.86 0.36 5.6 0.023 0.0100 0.34
## ssl_offerhelp 0.85 0.85 0.86 0.36 5.7 0.023 0.0104 0.36
## ssl_ease 0.84 0.84 0.85 0.34 5.2 0.025 0.0095 0.32
## ssl_advice 0.84 0.84 0.85 0.35 5.4 0.024 0.0096 0.34
## ssl_confidence 0.85 0.85 0.86 0.36 5.7 0.023 0.0108 0.36
## ssl_askhelp 0.85 0.85 0.86 0.37 5.8 0.022 0.0106 0.37
##
## Item statistics
## n raw.r std.r r.cor r.drop mean sd
## ssl_invite 94 0.55 0.53 0.47 0.42 1.9 0.81
## ssl_visit 93 0.62 0.63 0.59 0.53 1.6 0.74
## ssl_affection 92 0.64 0.65 0.60 0.55 2.1 0.77
## ssl_comfort 92 0.65 0.65 0.60 0.55 1.8 0.86
## ssl_compliment 93 0.68 0.69 0.65 0.60 1.8 0.75
## ssl_interest 93 0.65 0.65 0.61 0.56 1.8 0.80
## ssl_offerhelp 91 0.63 0.63 0.59 0.54 1.8 0.87
## ssl_ease 93 0.75 0.75 0.73 0.68 1.9 0.86
## ssl_advice 93 0.70 0.71 0.69 0.63 2.0 0.73
## ssl_confidence 93 0.63 0.62 0.58 0.53 2.2 0.83
## ssl_askhelp 93 0.61 0.60 0.55 0.50 2.0 0.89
##
## Non missing response frequency for each item
## 0 1 2 3 miss
## ssl_invite 0.05 0.19 0.51 0.24 0.03
## ssl_visit 0.08 0.35 0.49 0.08 0.04
## ssl_affection 0.03 0.15 0.51 0.30 0.05
## ssl_comfort 0.07 0.29 0.42 0.22 0.05
## ssl_compliment 0.04 0.29 0.52 0.15 0.04
## ssl_interest 0.04 0.32 0.45 0.18 0.04
## ssl_offerhelp 0.09 0.24 0.47 0.20 0.06
## ssl_ease 0.04 0.27 0.40 0.29 0.04
## ssl_advice 0.01 0.25 0.51 0.24 0.04
## ssl_confidence 0.02 0.22 0.35 0.41 0.04
## ssl_askhelp 0.05 0.24 0.38 0.33 0.04

psych::omega(ssldat, na.rm=T)


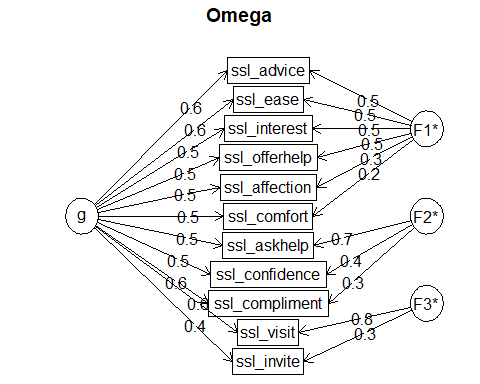


## Omega
## Call: omegah(m = m, nfactors = nfactors, fm = fm, key = key, flip = flip,
## digits = digits, title = title, sl = sl, labels = labels,
## plot = plot, n.obs = n.obs, rotate = rotate, Phi = Phi, option = option,
## covar = covar, na.rm = ..1)
## Alpha: 0.86
## G.6: 0.88
## Omega Hierarchical: 0.65
## Omega H asymptotic: 0.73
## Omega Total 0.89
##
## Schmid Leiman Factor loadings greater than 0.2
## g F1* F2* F3* h2 u2 p2
## ssl_invite 0.40 0.34 0.29 0.71 0.56
## ssl_visit 0.57 0.76 0.91 0.09 0.36
## ssl_affection 0.50 0.28 0.35 0.65 0.71
## ssl_comfort 0.50 0.25 0.35 0.65 0.72
## ssl_compliment 0.55 0.30 0.45 0.55 0.69
## ssl_interest 0.51 0.47 0.49 0.51 0.54
## ssl_offerhelp 0.48 0.45 0.44 0.56 0.52
## ssl_ease 0.62 0.48 0.62 0.38 0.62
## ssl_advice 0.58 0.50 0.58 0.42 0.57
## ssl_confidence 0.50 0.45 0.47 0.53 0.53
## ssl_askhelp 0.52 0.66 0.71 0.29 0.38
##
## With eigenvalues of:
## g F1* F2* F3*
## 3.03 1.09 0.76 0.78
##
## general/max 2.78 max/min = 1.43
## mean percent general = 0.56 with sd = 0.12 and cv of 0.21
## Explained Common Variance of the general factor = 0.54
##
## The degrees of freedom are 25 and the fit is 0.3
## The number of observations was 97 with Chi Square = 26.6 with prob < 0.38
## The root mean square of the residuals is 0.04
## The df corrected root mean square of the residuals is 0.06
## RMSEA index = 0.024 and the 10 % confidence intervals are 0 0.087
## BIC = -87.77
##
## Compare this with the adequacy of just a general factor and no group factors
## The degrees of freedom for just the general factor are 44 and the fit is 1.06
## The number of observations was 97 with Chi Square = 96.58 with prob < 8.3e-06
## The root mean square of the residuals is 0.13
## The df corrected root mean square of the residuals is 0.14
##
## RMSEA index = 0.111 and the 10 % confidence intervals are 0.081 0.142
## BIC = -104.7
##
## Measures of factor score adequacy
## g F1* F2* F3*
## Correlation of scores with factors 0.82 0.68 0.75 0.85
## Multiple R square of scores with factors 0.67 0.47 0.56 0.72
## Minimum correlation of factor score estimates 0.33 -0.07 0.12 0.44
##
## Total, General and Subset omega for each subset
## g F1* F2* F3*
## Omega total for total scores and subscales 0.89 0.83 0.75 0.72
## Omega general for total scores and subscales 0.65 0.52 0.42 0.32
## Omega group for total scores and subscales 0.18 0.30 0.34 0.41

vpvdat <- PLdat[T0, c("vpv_meetfriends", "vpv_recogniseemotion", "vpv_class", "vpv_help", "vpv_easycontact", "vpv_hurt", "vpv_questions",
 "vpv_sensitivities", "vpv_standup", "vpv_said", "vpv_seekcontact", "vpv_wishes", "vpv_getalong","vpv_interest",
 "vpv_talkconcerns","vpv_adapt", "vpv_goodcontact", "vpv_learn")]
psych::alpha(vpvdat, na.rm=T)

##
## Reliability analysis
## Call: psych::alpha(x = vpvdat, na.rm = T)
##
## raw_alpha std.alpha G6(smc) average_r S/N ase mean sd median_r
## 0.83 0.84 0.89 0.22 5.1 0.025 3.8 0.43 0.21
##
## 95% confidence boundaries
## lower alpha upper
## Feldt 0.78 0.83 0.87
## Duhachek 0.78 0.83 0.88
##
## Reliability if an item is dropped:
## raw_alpha std.alpha G6(smc) average_r S/N alpha se var.r med.r
## vpv_meetfriends 0.81 0.83 0.87 0.22 4.7 0.027 0.019 0.21
## vpv_recogniseemotion 0.82 0.83 0.88 0.23 5.0 0.026 0.021 0.22
## vpv_class 0.82 0.83 0.88 0.22 4.8 0.027 0.020 0.22
## vpv_help 0.82 0.83 0.88 0.22 4.9 0.026 0.021 0.22
## vpv_easycontact 0.83 0.84 0.89 0.23 5.2 0.024 0.019 0.22
## vpv_hurt 0.83 0.84 0.88 0.23 5.1 0.025 0.018 0.22
## vpv_questions 0.82 0.83 0.88 0.23 5.0 0.026 0.021 0.22
## vpv_sensitivities 0.82 0.82 0.87 0.21 4.6 0.027 0.018 0.21
## vpv_standup 0.82 0.83 0.87 0.22 4.8 0.027 0.021 0.21
## vpv_said 0.82 0.83 0.87 0.22 4.8 0.026 0.020 0.22
## vpv_seekcontact 0.82 0.83 0.88 0.22 4.8 0.027 0.021 0.21
## vpv_wishes 0.82 0.82 0.88 0.22 4.7 0.027 0.021 0.21
## vpv_getalong 0.82 0.83 0.88 0.22 4.8 0.027 0.021 0.21
## vpv_interest 0.82 0.82 0.87 0.21 4.6 0.027 0.021 0.21
## vpv_talkconcerns 0.81 0.82 0.87 0.21 4.6 0.028 0.020 0.21
## vpv_adapt 0.83 0.84 0.89 0.24 5.2 0.025 0.019 0.22
## vpv_goodcontact 0.81 0.82 0.87 0.21 4.6 0.028 0.019 0.21
## vpv_learn 0.82 0.82 0.87 0.22 4.7 0.027 0.020 0.21
##
## Item statistics
## n raw.r std.r r.cor r.drop mean sd
## vpv_meetfriends 93 0.61 0.58 0.56 0.52 4.1 0.96
## vpv_recogniseemotion 93 0.43 0.45 0.40 0.34 3.8 0.82
## vpv_class 93 0.56 0.51 0.48 0.45 3.7 1.09
## vpv_help 93 0.46 0.48 0.45 0.38 4.1 0.65
## vpv_easycontact 93 0.38 0.33 0.27 0.25 3.4 1.15
## vpv_hurt 93 0.36 0.39 0.36 0.26 3.9 0.83
## vpv_questions 93 0.45 0.42 0.38 0.34 3.2 0.93
## vpv_sensitivities 93 0.60 0.64 0.64 0.53 4.1 0.73
## vpv_standup 91 0.57 0.55 0.53 0.49 3.3 0.93
## vpv_said 93 0.50 0.51 0.49 0.41 4.0 0.84
## vpv_seekcontact 93 0.53 0.52 0.49 0.45 4.2 0.77
## vpv_wishes 93 0.55 0.59 0.56 0.48 3.9 0.73
## vpv_getalong 93 0.52 0.52 0.47 0.44 3.9 0.75
## vpv_interest 93 0.58 0.62 0.60 0.53 4.1 0.56
## vpv_talkconcerns 91 0.64 0.62 0.60 0.56 3.9 0.98
## vpv_adapt 93 0.26 0.31 0.25 0.18 3.9 0.67
## vpv_goodcontact 93 0.65 0.64 0.64 0.57 3.8 0.85
## vpv_learn 93 0.57 0.59 0.57 0.49 3.7 0.81
##
## Non missing response frequency for each item
## 1 2 3 4 5 miss
## vpv_meetfriends 0.01 0.04 0.20 0.28 0.46 0.04
## vpv_recogniseemotion 0.01 0.04 0.29 0.49 0.16 0.04
## vpv_class 0.02 0.16 0.14 0.41 0.27 0.04
## vpv_help 0.00 0.02 0.12 0.65 0.22 0.04
## vpv_easycontact 0.04 0.17 0.35 0.19 0.24 0.04
## vpv_hurt 0.01 0.02 0.27 0.45 0.25 0.04
## vpv_questions 0.02 0.20 0.35 0.35 0.06 0.04
## vpv_sensitivities 0.00 0.03 0.11 0.56 0.30 0.04
## vpv_standup 0.03 0.16 0.35 0.38 0.07 0.06
## vpv_said 0.01 0.04 0.16 0.52 0.27 0.04
## vpv_seekcontact 0.00 0.02 0.16 0.46 0.35 0.04
## vpv_wishes 0.00 0.04 0.18 0.59 0.18 0.04
## vpv_getalong 0.00 0.04 0.22 0.56 0.18 0.04
## vpv_interest 0.00 0.01 0.09 0.72 0.18 0.04
## vpv_talkconcerns 0.01 0.10 0.15 0.43 0.31 0.06
## vpv_adapt 0.00 0.01 0.23 0.58 0.18 0.04
## vpv_goodcontact 0.01 0.06 0.18 0.55 0.19 0.04
## vpv_learn 0.01 0.04 0.29 0.51 0.15 0.04

psych::omega(vpvdat, na.rm=T)


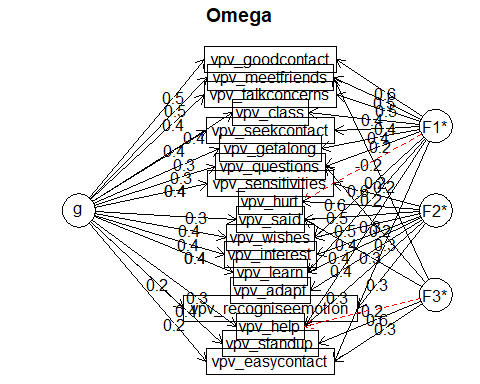


## Omega
## Call: omegah(m = m, nfactors = nfactors, fm = fm, key = key, flip = flip,
## digits = digits, title = title, sl = sl, labels = labels,
## plot = plot, n.obs = n.obs, rotate = rotate, Phi = Phi, option = option,
## covar = covar, na.rm = ..1)
## Alpha: 0.84
## G.6: 0.89
## Omega Hierarchical: 0.44
## Omega H asymptotic: 0.5
## Omega Total 0.87
##
## Schmid Leiman Factor loadings greater than 0.2
## g F1* F2* F3* h2 u2 p2
## vpv_meetfriends 0.47 0.49 0.25 0.53 0.47 0.41
## vpv_recogniseemotion 0.22 0.33 0.21 0.79 0.23
## vpv_class 0.39 0.43 0.36 0.64 0.42
## vpv_help 0.25 0.27 0.28 -0.24 0.27 0.73 0.23
## vpv_easycontact 0.25 0.26 0.28 0.25 0.75 0.25
## vpv_hurt -0.22 0.63 0.49 0.51 0.06
## vpv_questions 0.29 0.23 0.21 0.18 0.82 0.45
## vpv_sensitivities 0.41 0.64 0.61 0.39 0.28
## vpv_standup 0.44 0.65 0.65 0.35 0.30
## vpv_said 0.31 0.53 0.27 0.45 0.55 0.22
## vpv_seekcontact 0.35 0.42 0.30 0.70 0.42
## vpv_wishes 0.35 0.20 0.47 0.40 0.60 0.32
## vpv_getalong 0.35 0.37 0.28 0.72 0.43
## vpv_interest 0.42 0.40 0.39 0.61 0.44
## vpv_talkconcerns 0.45 0.48 0.44 0.56 0.46
## vpv_adapt 0.36 0.16 0.84 0.06
## vpv_goodcontact 0.49 0.55 0.55 0.45 0.44
## vpv_learn 0.38 0.20 0.39 0.35 0.65 0.42
##
## With eigenvalues of:
## g F1* F2* F3*
## 2.27 1.70 2.04 0.87
##
## general/max 1.11 max/min = 2.35
## mean percent general = 0.32 with sd = 0.13 and cv of 0.4
## Explained Common Variance of the general factor = 0.33
##
## The degrees of freedom are 102 and the fit is 1.9
## The number of observations was 97 with Chi Square = 165.23 with prob < 7.5e-05
## The root mean square of the residuals is 0.06
## The df corrected root mean square of the residuals is 0.08
## RMSEA index = 0.079 and the 10 % confidence intervals are 0.057 0.102
## BIC = -301.39
##
## Compare this with the adequacy of just a general factor and no group factors
## The degrees of freedom for just the general factor are 135 and the fit is 3.73
## The number of observations was 97 with Chi Square = 330.31 with prob < 2.2e-18
## The root mean square of the residuals is 0.16
## The df corrected root mean square of the residuals is 0.17
##
## RMSEA index = 0.122 and the 10 % confidence intervals are 0.106 0.14
## BIC = -287.28
##
## Measures of factor score adequacy
## g F1* F2* F3*
## Correlation of scores with factors 0.69 0.72 0.84 0.77
## Multiple R square of scores with factors 0.47 0.51 0.70 0.60
## Minimum correlation of factor score estimates -0.06 0.03 0.41 0.20
##
## Total, General and Subset omega for each subset
## g F1* F2* F3*
## Omega total for total scores and subscales 0.87 0.78 0.78 0.51
## Omega general for total scores and subscales 0.44 0.36 0.23 0.18
## Omega group for total scores and subscales 0.30 0.42 0.55 0.33

amountdat <- PLdat[T1, c("covid_symptoms_self", "covid_symptoms_family", "covid_risk_self", "covid_risk_family", "covid_job_family",
 "covid_job_self", "covid_healthcare", "covid_restriction", "covid_lesspeople", "covid_socialevents",
 "covid_hospital", "covid_funeral", "covid_hobby", "covid_move", "covid_tension_parents", "covid_tension_self",
 "covid_siblings", "covid_travel", "covid_basics", "covid_media", "covid_school", "covid_money")]
psych::alpha(amountdat, na.rm=T)

## Warning in psych::alpha(amountdat, na.rm = T): Some items were negatively correlated with the total scale and probably
## should be reversed.
## To do this, run the function again with the 'check.keys=TRUE' option

## Some items ( covid_risk_self covid_funeral covid_money ) were negatively correlated with the total scale and
## probably should be reversed.
## To do this, run the function again with the 'check.keys=TRUE' option

##
## Reliability analysis
## Call: psych::alpha(x = amountdat, na.rm = T)
##
## raw_alpha std.alpha G6(smc) average_r S/N ase mean sd median_r
## 0.49 0.45 0.59 0.036 0.83 0.071 0.47 0.12 0.029
##
## 95% confidence boundaries
## lower alpha upper
## Feldt 0.33 0.49 0.62
## Duhachek 0.35 0.49 0.63
##
## Reliability if an item is dropped:
## raw_alpha std.alpha G6(smc) average_r S/N alpha se var.r med.r
## covid_symptoms_self 0.46 0.42 0.56 0.033 0.72 0.076 0.014 0.025
## covid_symptoms_family 0.48 0.43 0.56 0.035 0.77 0.073 0.013 0.029
## covid_risk_self 0.52 0.49 0.62 0.045 0.98 0.068 0.014 0.037
## covid_risk_family 0.47 0.43 0.57 0.035 0.76 0.073 0.014 0.027
## covid_job_family 0.50 0.45 0.58 0.038 0.83 0.070 0.015 0.029
## covid_job_self 0.51 0.48 0.60 0.042 0.92 0.069 0.014 0.032
## covid_healthcare 0.49 0.46 0.59 0.038 0.84 0.072 0.015 0.032
## covid_restriction 0.45 0.42 0.56 0.033 0.72 0.077 0.014 0.022
## covid_lesspeople 0.49 0.44 0.57 0.036 0.78 0.072 0.015 0.026
## covid_socialevents 0.50 0.46 0.58 0.039 0.85 0.070 0.014 0.029
## covid_hospital 0.51 0.48 0.60 0.041 0.91 0.070 0.014 0.032
## covid_funeral 0.50 0.47 0.59 0.040 0.88 0.071 0.014 0.032
## covid_hobby 0.45 0.41 0.54 0.032 0.70 0.078 0.013 0.025
## covid_move 0.47 0.43 0.56 0.034 0.74 0.075 0.014 0.026
## covid_tension_parents 0.42 0.39 0.53 0.029 0.63 0.082 0.013 0.022
## covid_tension_self 0.43 0.41 0.55 0.032 0.69 0.080 0.013 0.026
## covid_siblings 0.46 0.44 0.57 0.035 0.77 0.075 0.013 0.029
## covid_travel 0.49 0.46 0.59 0.039 0.85 0.071 0.014 0.036
## covid_basics 0.48 0.45 0.58 0.037 0.81 0.072 0.015 0.026
## covid_media 0.46 0.41 0.55 0.032 0.70 0.075 0.014 0.027
## covid_school 0.47 0.42 0.56 0.033 0.72 0.074 0.015 0.019
## covid_money 0.52 0.48 0.60 0.043 0.93 0.068 0.014 0.036
##
## Item statistics
## n raw.r std.r r.cor r.drop mean sd
## covid_symptoms_self 100 0.403 0.397 0.365 0.2653 0.19 0.39
## covid_symptoms_family 100 0.339 0.321 0.300 0.1548 0.48 0.50
## covid_risk_self 100 -0.063 0.012 -0.164 -0.1657 0.08 0.27
## covid_risk_family 100 0.346 0.334 0.279 0.1685 0.63 0.49
## covid_job_family 100 0.255 0.229 0.132 0.0650 0.51 0.50
## covid_job_self 100 0.080 0.093 -0.028 -0.0497 0.13 0.34
## covid_healthcare 100 0.164 0.216 0.116 0.0736 0.06 0.24
## covid_restriction 100 0.426 0.401 0.372 0.2574 0.64 0.48
## covid_lesspeople 100 0.192 0.304 0.234 0.1020 0.94 0.24
## covid_socialevents 100 0.193 0.202 0.109 0.0318 0.77 0.42
## covid_hospital 100 0.167 0.116 -0.017 0.0029 0.24 0.43
## covid_funeral 100 0.078 0.156 0.052 -0.0053 0.05 0.22
## covid_hobby 100 0.461 0.424 0.427 0.3022 0.68 0.47
## covid_move 100 0.378 0.361 0.316 0.2033 0.63 0.49
## covid_tension_parents 100 0.564 0.529 0.565 0.4155 0.38 0.49
## covid_tension_self 100 0.505 0.445 0.443 0.3452 0.61 0.49
## covid_siblings 100 0.393 0.317 0.269 0.2150 0.55 0.50
## covid_travel 100 0.254 0.204 0.103 0.0820 0.71 0.46
## covid_basics 100 0.208 0.264 0.187 0.1120 0.07 0.26
## covid_media 100 0.386 0.431 0.421 0.2500 0.82 0.39
## covid_school 100 0.318 0.400 0.352 0.2484 0.96 0.20
## covid_money 100 0.054 0.075 -0.053 -0.0931 0.18 0.39
##
## Non missing response frequency for each item
## 0 1 miss
## covid_symptoms_self 0.81 0.19 0
## covid_symptoms_family 0.52 0.48 0
## covid_risk_self 0.92 0.08 0
## covid_risk_family 0.37 0.63 0
## covid_job_family 0.49 0.51 0
## covid_job_self 0.87 0.13 0
## covid_healthcare 0.94 0.06 0
## covid_restriction 0.36 0.64 0
## covid_lesspeople 0.06 0.94 0
## covid_socialevents 0.23 0.77 0
## covid_hospital 0.76 0.24 0
## covid_funeral 0.95 0.05 0
## covid_hobby 0.32 0.68 0
## covid_move 0.37 0.63 0
## covid_tension_parents 0.62 0.38 0
## covid_tension_self 0.39 0.61 0
## covid_siblings 0.45 0.55 0
## covid_travel 0.29 0.71 0
## covid_basics 0.93 0.07 0
## covid_media 0.18 0.82 0
## covid_school 0.04 0.96 0
## covid_money 0.82 0.18 0

psych::omega(amountdat, na.rm=T)


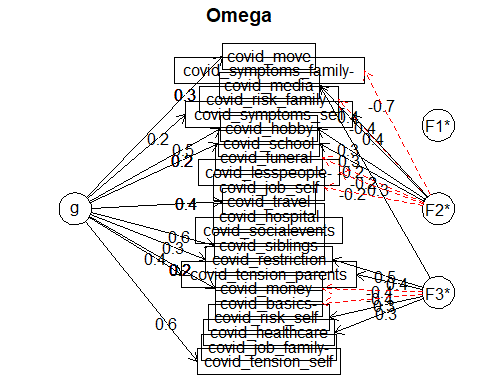


## Omega
## Call: omegah(m = m, nfactors = nfactors, fm = fm, key = key, flip = flip,
## digits = digits, title = title, sl = sl, labels = labels,
## plot = plot, n.obs = n.obs, rotate = rotate, Phi = Phi, option = option,
## covar = covar, na.rm = ..1)
## Alpha: 0.45
## G.6: 0.59
## Omega Hierarchical: 0.5
## Omega H asymptotic: 0.93
## Omega Total 0.53
##
## Schmid Leiman Factor loadings greater than 0.2
## g F1* F2* F3* h2 u2 p2
## covid_symptoms_self 0.23 0.36 0.19 0.81 0.29
## covid_symptoms_family- -0.69 0.49 0.51 0.00
## covid_risk_self- 0.25 0.09 0.91 0.22
## covid_risk_family- -0.39 0.16 0.84 0.00
## covid_job_family- 0.05 0.95 0.12
## covid_job_self -0.22 0.07 0.93 0.01
## covid_healthcare 0.25 0.07 0.93 0.05
## covid_restriction 0.25 0.49 0.30 0.70 0.21
## covid_lesspeople- -0.22 0.06 0.94 0.01
## covid_socialevents 0.02 0.98 0.21
## covid_hospital 0.02 0.98 0.10
## covid_funeral- -0.22 0.08 0.92 0.17
## covid_hobby 0.47 0.31 0.32 0.68 0.69
## covid_move 0.34 0.12 0.88 0.99
## covid_tension_parents 0.44 0.39 0.35 0.65 0.56
## covid_tension_self 0.59 0.36 0.64 0.96
## covid_siblings 0.58 0.34 0.66 0.98
## covid_travel 0.39 -0.20 0.19 0.81 0.79
## covid_basics- -0.37 0.14 0.86 0.00
## covid_media 0.43 0.29 0.27 0.73 0.00
## covid_school 0.21 0.27 0.12 0.88 0.37
## covid_money- 0.24 -0.39 0.22 0.78 0.26
##
## With eigenvalues of:
## g F1* F2* F3*
## 1.64 0.01 1.37 1.01
##
## general/max 1.2 max/min = 189.49
## mean percent general = 0.32 with sd = 0.35 and cv of 1.09
## Explained Common Variance of the general factor = 0.41
##
## The degrees of freedom are 168 and the fit is 1.95
## The number of observations was 100 with Chi Square = 173.31 with prob < 0.37
## The root mean square of the residuals is 0.07
## The df corrected root mean square of the residuals is 0.08
## RMSEA index = 0.015 and the 10 % confidence intervals are 0 0.049
## BIC = -600.36
##
## Compare this with the adequacy of just a general factor and no group factors
## The degrees of freedom for just the general factor are 209 and the fit is 2.83
## The number of observations was 100 with Chi Square = 255.16 with prob < 0.016
## The root mean square of the residuals is 0.1
## The df corrected root mean square of the residuals is 0.11
##
## RMSEA index = 0.046 and the 10 % confidence intervals are 0.022 0.066
## BIC = -707.32
##
## Measures of factor score adequacy
## g F1* F2* F3*
## Correlation of scores with factors 0.83 0.06 0.82 0.75
## Multiple R square of scores with factors 0.69 0.00 0.67 0.56
## Minimum correlation of factor score estimates 0.38 -0.99 0.35 0.12
##
## Total, General and Subset omega for each subset
## g F1* F2* F3*
## Omega total for total scores and subscales 0.53 0.12 0.32 0.34
## Omega general for total scores and subscales 0.50 0.12 0.30 0.31
## Omega group for total scores and subscales 0.01 0.00 0.01 0.03

# burdendat <- PLdat[T1, c("covid_symptoms_self_burden", "covid_symptoms_family_burden", "covid_risk_self_burden", "covid_risk_family_burden",
# "covid_healthcare_burden", "covid_job_family_burden", "covid_job_self_burden", "covid_restriction_burden",
# "covid_lesspeople_burden", "covid_socialevents_burden","covid_hospital_burden", "covid_funeral_burden",
# "covid_hobby_burden", "covid_move_burden", "covid_tension_parents_burden", "covid_tension_self_burden",
# "covid_siblings_burden", "covid_travel_burden", "covid_basics_burden", "covid_media_burden",
# "covid_school_burden", "covid_money_burden")]
# psych::alpha(burdendat, na.rm=T)
# psych::omega(burdendat, na.rm=T)

mspssdat <- PLdat[T1, c("mspss_person_help", "mspss_person_joy", "mspss_family_help", "mspss_family_support", "mspss_person_comfort",
 "mspss_friends_help", "mspss_friends_count", "mspss_family_talk", "mspss_friends_joy", "mspss_person_feelings",
 "mspss_family_decide", "mspss_friends_talk")]
psych::alpha(mspssdat, na.rm=T)

##
## Reliability analysis
## Call: psych::alpha(x = mspssdat, na.rm = T)
##
## raw_alpha std.alpha G6(smc) average_r S/N ase mean sd median_r
## 0.87 0.88 0.95 0.37 7.1 0.02 4.9 1.2 0.34
##
## 95% confidence boundaries
## lower alpha upper
## Feldt 0.83 0.87 0.91
## Duhachek 0.83 0.87 0.91
##
## Reliability if an item is dropped:
## raw_alpha std.alpha G6(smc) average_r S/N alpha se var.r med.r
## mspss_person_help 0.87 0.88 0.95 0.39 7.1 0.020 0.072 0.37
## mspss_person_joy 0.86 0.87 0.94 0.38 6.7 0.021 0.074 0.37
## mspss_family_help 0.85 0.86 0.94 0.36 6.2 0.023 0.071 0.32
## mspss_family_support 0.85 0.86 0.94 0.36 6.2 0.023 0.067 0.32
## mspss_person_comfort 0.87 0.88 0.94 0.39 7.1 0.020 0.069 0.37
## mspss_friends_help 0.85 0.86 0.94 0.36 6.1 0.023 0.077 0.32
## mspss_friends_count 0.86 0.86 0.94 0.36 6.3 0.022 0.072 0.32
## mspss_family_talk 0.86 0.86 0.94 0.36 6.3 0.023 0.068 0.32
## mspss_friends_joy 0.86 0.86 0.94 0.36 6.2 0.023 0.073 0.32
## mspss_person_feelings 0.87 0.88 0.95 0.39 7.1 0.020 0.073 0.37
## mspss_family_decide 0.86 0.87 0.94 0.37 6.5 0.022 0.065 0.32
## mspss_friends_talk 0.86 0.86 0.94 0.36 6.1 0.023 0.076 0.31
##
## Item statistics
## n raw.r std.r r.cor r.drop mean sd
## mspss_person_help 99 0.53 0.51 0.46 0.42 4.3 2.1
## mspss_person_joy 99 0.61 0.59 0.56 0.51 5.0 2.0
## mspss_family_help 99 0.73 0.72 0.72 0.66 4.3 2.0
## mspss_family_support 99 0.73 0.73 0.73 0.66 4.4 2.0
## mspss_person_comfort 99 0.52 0.50 0.46 0.41 4.6 2.0
## mspss_friends_help 99 0.73 0.75 0.74 0.67 4.9 1.8
## mspss_friends_count 99 0.67 0.70 0.69 0.60 5.6 1.6
## mspss_family_talk 99 0.70 0.69 0.69 0.61 4.5 2.1
## mspss_friends_joy 99 0.68 0.71 0.70 0.62 5.7 1.6
## mspss_person_feelings 99 0.51 0.49 0.45 0.40 5.0 2.1
## mspss_family_decide 99 0.65 0.64 0.64 0.56 4.8 1.9
## mspss_friends_talk 99 0.73 0.76 0.75 0.67 5.6 1.7
##
## Non missing response frequency for each item
## 1 2 3 4 5 6 7 miss
## mspss_person_help 0.16 0.11 0.04 0.19 0.10 0.19 0.20 0.01
## mspss_person_joy 0.10 0.08 0.04 0.11 0.12 0.26 0.28 0.01
## mspss_family_help 0.12 0.13 0.08 0.16 0.15 0.18 0.17 0.01
## mspss_family_support 0.11 0.11 0.08 0.25 0.09 0.14 0.21 0.01
## mspss_person_comfort 0.08 0.11 0.08 0.21 0.09 0.19 0.23 0.01
## mspss_friends_help 0.08 0.04 0.06 0.21 0.15 0.21 0.24 0.01
## mspss_friends_count 0.04 0.02 0.07 0.05 0.14 0.29 0.38 0.01
## mspss_family_talk 0.12 0.13 0.09 0.13 0.12 0.16 0.24 0.01
## mspss_friends_joy 0.04 0.03 0.04 0.05 0.15 0.33 0.35 0.01
## mspss_person_feelings 0.12 0.04 0.06 0.17 0.05 0.22 0.33 0.01
## mspss_family_decide 0.07 0.09 0.08 0.15 0.15 0.23 0.22 0.01
## mspss_friends_talk 0.04 0.04 0.06 0.07 0.11 0.30 0.37 0.01

psych::omega(mspssdat, na.rm=T)


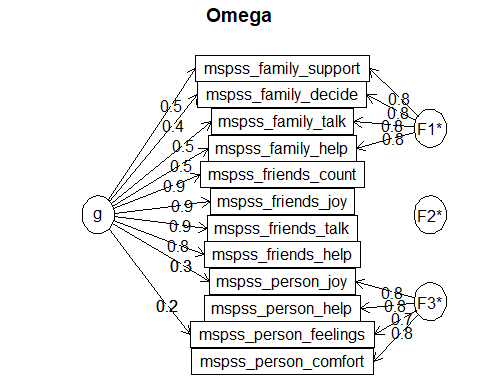


## Omega
## Call: omegah(m = m, nfactors = nfactors, fm = fm, key = key, flip = flip,
## digits = digits, title = title, sl = sl, labels = labels,
## plot = plot, n.obs = n.obs, rotate = rotate, Phi = Phi, option = option,
## covar = covar, na.rm = ..1)
## Alpha: 0.88
## G.6: 0.95
## Omega Hierarchical: 0.62
## Omega H asymptotic: 0.65
## Omega Total 0.95
##
## Schmid Leiman Factor loadings greater than 0.2
## g F1* F2* F3* h2 u2 p2
## mspss_person_help 0.77 0.62 0.38 0.04
## mspss_person_joy 0.28 0.82 0.76 0.24 0.10
## mspss_family_help 0.45 0.76 0.78 0.22 0.26
## mspss_family_support 0.48 0.83 0.92 0.08 0.25
## mspss_person_comfort 0.83 0.72 0.28 0.04
## mspss_friends_help 0.82 0.70 0.30 0.97
## mspss_friends_count 0.92 0.84 0.16 0.99
## mspss_family_talk 0.47 0.76 0.80 0.20 0.28
## mspss_friends_joy 0.89 0.80 0.20 1.00
## mspss_person_feelings 0.20 0.70 0.53 0.47 0.08
## mspss_family_decide 0.42 0.80 0.83 0.17 0.21
## mspss_friends_talk 0.86 0.74 0.26 0.99
##
## With eigenvalues of:
## g F1* F2* F3*
## 4.0 2.5 0.0 2.5
##
## general/max 1.63 max/min = 724.27
## mean percent general = 0.43 with sd = 0.42 and cv of 0.96
## Explained Common Variance of the general factor = 0.45
##
## The degrees of freedom are 33 and the fit is 0.8
## The number of observations was 100 with Chi Square = 73.57 with prob < 6.3e-05
## The root mean square of the residuals is 0.03
## The df corrected root mean square of the residuals is 0.04
## RMSEA index = 0.11 and the 10 % confidence intervals are 0.077 0.146
## BIC = -78.4
##
## Compare this with the adequacy of just a general factor and no group factors
## The degrees of freedom for just the general factor are 54 and the fit is 6.32
## The number of observations was 100 with Chi Square = 591.2 with prob < 2e-91
## The root mean square of the residuals is 0.27
## The df corrected root mean square of the residuals is 0.3
##
## RMSEA index = 0.315 and the 10 % confidence intervals are 0.294 0.34
## BIC = 342.52
##
## Measures of factor score adequacy
## g F1* F2* F3*
## Correlation of scores with factors 0.97 0.97 0.04 0.94
## Multiple R square of scores with factors 0.94 0.93 0.00 0.89
## Minimum correlation of factor score estimates 0.87 0.87 -1.00 0.78
##
## Total, General and Subset omega for each subset
## g F1* F2* F3*
## Omega total for total scores and subscales 0.95 0.95 NA 0.92
## Omega general for total scores and subscales 0.62 0.24 NA 0.59
## Omega group for total scores and subscales 0.33 0.71 NA 0.33

# ##GRAND MEAN CENTER MODERATORS & COVARIATES##
# PLdat$c_ssl_total <- PLdat$ssl_total - mean(PLdat$ssl_total, na.rm=T)
# PLdat$c_vpv_interpers <- PLdat$vpv_interpers - mean(PLdat$vpv_interpers, na.rm=T)
# PLdat$c_stressors_amount <- PLdat$stressors_amount - mean(PLdat$stressors_amount, na.rm=T)
# PLdat$c_stressors_burden <- PLdat$stressors_burden - mean(PLdat$stressors_burden, na.rm=T)
# PLdat$c_mspsss_total <- PLdat$mspss_total - mean(PLdat$mspss_total, na.rm=T)

######################################################################################################
##RQ1: CHANGES FROM T0 TO T1##

PLdat$wave.f = as.factor(PLdat$wave.f)
PLdat$sex = as.factor(PLdat$sex)
dat$wave.f = as.factor(dat$wave.f)
dat$sex = as.factor(dat$sex_ismale)

PLdat = data.frame(PLdat)
dat = data.frame(dat)

#Alpha = .007 (Bonferroni corrected, initial alpha of .05)

H1a <- lme(PL_aloneprop ~ wave.f + c_age + sex, data = PLdat, random = ~1| record_id)
summary(H1a)

## Linear mixed-effects model fit by REML
## Data: PLdat
## AIC BIC logLik
## -95.50339 -75.92725 53.75169
##
## Random effects:
## Formula: ~1 | record_id
## (Intercept) Residual
## StdDev: 0.09671927 0.1499653
##
## Fixed effects: PL_aloneprop ~ wave.f + c_age + sex
## Value Std.Error DF t-value p-value
## (Intercept) 0.21055349 0.02013446 107 10.457370 0.0000
## wave.f2 0.20224556 0.02594837 86 7.794153 0.0000
## c_age 0.01096474 0.00770482 86 1.423103 0.1583
## sex2 -0.04061042 0.05363695 107 -0.757135 0.4506
## Correlation:
## (Intr) wav.f2 c_age
## wave.f2 -0.676
## c_age 0.388 -0.547
## sex2 -0.273 0.098 -0.146
##
## Standardized Within-Group Residuals:
## Min Q1 Med Q3 Max
## -2.01866571 -0.61520085 0.01216243 0.53889500 2.81152299
##
## Number of Observations: 197
## Number of Groups: 109

H1b <- lme(momentPA ~ wave.f + c_age + sex, data = dat, random = ~1| record_id,
 method = "REML", na.action = na.omit, control = lmeControl(opt='optim'))
summary(H1b)

## Linear mixed-effects model fit by REML
## Data: dat
## AIC BIC logLik
## 5958.231 5991.417 -2973.115
##
## Random effects:
## Formula: ~1 | record_id
## (Intercept) Residual
## StdDev: 0.799205 1.116571
##
## Fixed effects: momentPA ~ wave.f + c_age + sex
## Value Std.Error DF t-value p-value
## (Intercept) 4.897556 0.1028219 1758 47.63144 0.0000
## wave.fcovid 0.240194 0.0998569 1758 2.40539 0.0163
## c_age -0.162475 0.0423292 1758 -3.83837 0.0001
## sex1 -0.073374 0.3274219 107 -0.22410 0.8231
## Correlation:
## (Intr) wv.fcv c_age
## wave.fcovid -0.522
## c_age 0.380 -0.798
## sex1 -0.289 0.125 -0.137
##
## Standardized Within-Group Residuals:
## Min Q1 Med Q3 Max
## -4.2380823 -0.5158796 0.1193948 0.6414152 3.0602170
##
## Number of Observations: 1869
## Number of Groups: 109

H1c <- lme(momentNA ~ wave.f + c_age + sex, data = dat, random = ~1| record_id,
 method = "REML", na.action = na.omit, control = lmeControl(opt='optim'))
summary(H1c)

## Linear mixed-effects model fit by REML
## Data: dat
## AIC BIC logLik
## 5087.731 5120.917 -2537.865
##
## Random effects:
## Formula: ~1 | record_id
## (Intercept) Residual
## StdDev: 0.86068 0.8702507
##
## Fixed effects: momentNA ~ wave.f + c_age + sex
## Value Std.Error DF t-value p-value
## (Intercept) 2.0339444 0.1030624 1758 19.735084 0.0000
## wave.fcovid 0.1327140 0.0921039 1758 1.440917 0.1498
## c_age 0.0273901 0.0416805 1758 0.657145 0.5112
## sex1 -0.0369138 0.3313434 107 -0.111407 0.9115
## Correlation:
## (Intr) wv.fcv c_age
## wave.fcovid -0.471
## c_age 0.379 -0.858
## sex1 -0.296 0.122 -0.129
##
## Standardized Within-Group Residuals:
## Min Q1 Med Q3 Max
## -3.4413464 -0.5417317 -0.1342199 0.4155864 4.5278355
##
## Number of Observations: 1869
## Number of Groups: 109

H1d <- lme(esm_soc_alone_pleasant ~ wave.f + c_age + sex, data = dat, random = ~1| record_id,
 method = "REML", na.action = na.omit, control = lmeControl(opt='optim'))
summary(H1d)

## Linear mixed-effects model fit by REML
## Data: dat
## AIC BIC logLik
## 6694.985 6728.165 -3341.493
##
## Random effects:
## Formula: ~1 | record_id
## (Intercept) Residual
## StdDev: 1.127397 1.353037
##
## Fixed effects: esm_soc_alone_pleasant ~ wave.f + c_age + sex
## Value Std.Error DF t-value p-value
## (Intercept) 5.460126 0.1398063 1756 39.05493 0.0000
## wave.fcovid -0.145946 0.1307582 1756 -1.11615 0.2645
## c_age -0.144697 0.0573147 1756 -2.52461 0.0117
## sex1 -0.180027 0.4472018 107 -0.40256 0.6881
## Correlation:
## (Intr) wv.fcv c_age
## wave.fcovid -0.498
## c_age 0.382 -0.828
## sex1 -0.293 0.124 -0.133
##
## Standardized Within-Group Residuals:
## Min Q1 Med Q3 Max
## -4.1865542 -0.4596522 0.1741967 0.5804040 2.3056720
##
## Number of Observations: 1867
## Number of Groups: 109

H1e <- lme(esm_soc_alone_want ~ wave.f + c_age + sex, data = dat, random = ~1| record_id,
 method = "REML", na.action = na.omit, control = lmeControl(opt='optim'))
summary(H1e)

## Linear mixed-effects model fit by REML
## Data: dat
## AIC BIC logLik
## 7321.952 7355.122 -3654.976
##
## Random effects:
## Formula: ~1 | record_id
## (Intercept) Residual
## StdDev: 1.21596 1.613776
##
## Fixed effects: esm_soc_alone_want ~ wave.f + c_age + sex
## Value Std.Error DF t-value p-value
## (Intercept) 4.794667 0.1544069 1753 31.052163 0.0000
## wave.fcovid 0.111453 0.1480979 1753 0.752562 0.4518
## c_age -0.147832 0.0635269 1753 -2.327072 0.0201
## sex1 0.216865 0.4924377 107 0.440390 0.6605
## Correlation:
## (Intr) wv.fcv c_age
## wave.fcovid -0.514
## c_age 0.381 -0.808
## sex1 -0.290 0.125 -0.136
##
## Standardized Within-Group Residuals:
## Min Q1 Med Q3 Max
## -3.3559642 -0.5233677 0.1666928 0.6156194 2.7282047
##
## Number of Observations: 1864
## Number of Groups: 109

H1f <- lme(esm_soc_alone_excluded ~ wave.f + c_age + sex, data = dat, random = ~1| record_id,
 method = "REML", na.action = na.omit, control = lmeControl(opt='optim'))
summary(H1f)

## Linear mixed-effects model fit by REML
## Data: dat
## AIC BIC logLik
## 4767.274 4800.441 -2377.637
##
## Random effects:
## Formula: ~1 | record_id
## (Intercept) Residual
## StdDev: 0.5736302 0.8152579
##
## Fixed effects: esm_soc_alone_excluded ~ wave.f + c_age + sex
## Value Std.Error DF t-value p-value
## (Intercept) 1.3207248 0.07413906 1752 17.814156 0.0000
## wave.fcovid 0.1188747 0.07232037 1752 1.643724 0.1004
## c_age -0.0055005 0.03052467 1752 -0.180198 0.8570
## sex1 0.0090085 0.23595351 107 0.038179 0.9696
## Correlation:
## (Intr) wv.fcv c_age
## wave.fcovid -0.525
## c_age 0.380 -0.794
## sex1 -0.288 0.125 -0.137
##
## Standardized Within-Group Residuals:
## Min Q1 Med Q3 Max
## -2.685763078 -0.254666111 -0.086286126 0.008654874 7.133744919
##
## Number of Observations: 1863
## Number of Groups: 109

H1g <- lme(esm_mood_lonely ~ wave.f + c_age + sex, data = dat, random = ~1| record_id,
 method = "REML", na.action = na.omit, control = lmeControl(opt='optim'))
summary(H1g)

## Linear mixed-effects model fit by REML
## Data: dat
## AIC BIC logLik
## 6715.376 6748.562 -3351.688
##
## Random effects:
## Formula: ~1 | record_id
## (Intercept) Residual
## StdDev: 0.9434008 1.370355
##
## Fixed effects: esm_mood_lonely ~ wave.f + c_age + sex
## Value Std.Error DF t-value p-value
## (Intercept) 1.8617356 0.1226463 1758 15.179710 0.0000
## wave.fcovid 0.2153953 0.1202309 1758 1.791513 0.0734
## c_age -0.0041368 0.0504908 1758 -0.081932 0.9347
## sex1 0.4093938 0.3900788 107 1.049516 0.2963
## Correlation:
## (Intr) wv.fcv c_age
## wave.fcovid -0.529
## c_age 0.379 -0.789
## sex1 -0.288 0.125 -0.137
##
## Standardized Within-Group Residuals:
## Min Q1 Med Q3 Max
## -2.9492501 -0.4262437 -0.2193715 0.1728490 4.1749590
##
## Number of Observations: 1869
## Number of Groups: 109

#Plot significant effects
effH1a <- as.data.frame(effect(term="wave.f",mod=H1a))[,c('wave.f','fit','se','lower','upper')]
effH1a$wave.f <- as.factor(effH1a$wave.f)
plot_H1a <- ggplot(effH1a, aes(x = wave.f, y= fit)) +
 geom_bar(stat="summary", fun="mean", position="dodge", width=.5)+
 #geom_signif(comparisons=list(c("1", "2")), map_signif_level = TRUE)+
 labs(title = "Change in Proportion \n of Time Spent Socially Withdrawing", x= "Timepoint",
 y="Prop. Time Spent \n Socially Withdrawing") + theme_classic() + theme(axis.title=element_text(size=10)) + theme(plot.title=element_text(size=12)) +
 theme(plot.title=element_text(face="bold")) + theme(plot.title=element_text(hjust=0.5)) + scale_x_discrete(labels=c("T0", "T1"))
plot_H1a


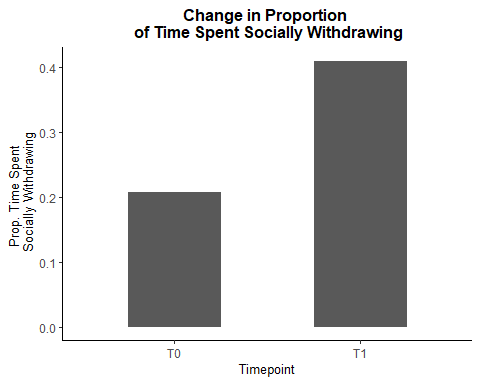


ggsave("H1a.png")

## Saving 5 x 4 in image

######################################################################################################
##PREPARE DATASETS FOR MODERATOR ANALYSES##
#For the moderators that were only measured at T1, we need to use difference scores as outcome variables in order to make inferences
#about the change from T0 to T1

#Create a difference score variable for aloneprop (delta_aloneprop = aloneprop[T1] - aloneprop[T0])
PLdat <- PLdat %>%
 group_by(record_id) %>%
 mutate(delta_aloneprop = ifelse(length(which(wave == 'wave1'))>0, (diff(PL_aloneprop)*-1)/PL_aloneprop[T0], NA)) %>%
 ungroup()

#Create "difference scores" for the beep-level outcomes
#We calculate the difference at T1 compared to the person-level mean at T0
#Create columns with T0 means
PLdat <- PLdat %>%
 group_by(record_id) %>%
 mutate(momentPA_wave1 = ifelse(length(which(wave == 'wave1'))>0, PL_momentPA[T0], NA),
 momentNA_wave1 = ifelse(length(which(wave == 'wave1'))>0, PL_momentNA[T0], NA),
 pleasant_wave1 = ifelse(length(which(wave == 'wave1'))>0, PL_pleasant[T0], NA),
 excluded_wave1 = ifelse(length(which(wave == 'wave1'))>0, PL_excluded[T0], NA),
 want_wave1 = ifelse(length(which(wave == 'wave1'))>0, PL_want[T0], NA),
 loneliness_wave1 = ifelse(length(which(wave == 'wave1'))>0, PL_loneliness[T0], NA)) %>%
 ungroup()

#Append columns from PLdat to dat
# dat$groupMean = as.integer(dat$groupMean)
# PLdat$groupMean = as.integer(PLdat$groupMean)
mergedat = subset(PLdat, select = c("record_id", "wave", "stressors_amount", "stressors_burden", "groupMean", "delta_aloneprop", "momentPA_wave1",
 "momentNA_wave1", "pleasant_wave1", "excluded_wave1", "want_wave1", "loneliness_wave1"))
dat = left_join(x = dat, y = mergedat)

## Joining, by = c("record_id", "wave")

#Create momentary "difference scores"
dat <- dat %>%
 group_by(record_id) %>%
 mutate(delta_PA = ifelse(wave == 'covid', (momentPA - momentPA_wave1)/momentPA_wave1, NA),
 delta_NA = ifelse(wave == 'covid', (momentNA - momentNA_wave1)/momentNA_wave1, NA),
 delta_pleasant = ifelse(wave == 'covid', (esm_soc_alone_pleasant - pleasant_wave1)/pleasant_wave1, NA),
 delta_excluded = ifelse(wave == 'covid', (esm_soc_alone_excluded - excluded_wave1)/excluded_wave1, NA),
 delta_want = ifelse(wave == 'covid', (esm_soc_alone_want - want_wave1)/want_wave1, NA),
 delta_loneliness = ifelse(wave == 'covid', (esm_mood_lonely - loneliness_wave1)/loneliness_wave1, NA)) %>%
 ungroup()

######################################################################################################
##RQ2 PART 1: MODERATORS IN CHANGES FROM T0 TO T1 (NON-IMPUTED MODERATORS)##

#Alpha per moderator = .007 (Bonferroni corrected, initial alpha of .05)

PLdat$groupMean = as.factor(PLdat$groupMean)
PLdat$wave.f = as.factor(PLdat$wave.f)
PLdat$sex = as.factor(PLdat$sex)
dat$groupMean = as.factor(dat$groupMean)
dat$wave.f = as.factor(dat$wave.f)
dat$sex = as.factor(dat$sex_ismale)

PLcovidDat <- subset(PLdat[PLdat$wave == 'covid',], select = c("record_id", "stressors_amount", "stressors_burden", "groupMean", "delta_aloneprop",
 "PL_aloneprop", "c_age", "sex"))
ESMcovidDat <- subset(dat[dat$wave == 'covid',], select = c("record_id", "stressors_amount", "stressors_burden", "groupMean", "delta_PA", "delta_NA",
 "delta_pleasant", "delta_excluded", "delta_want", "delta_loneliness", "momentPA", "momentNA",
 "esm_soc_alone_pleasant", "esm_soc_alone_excluded", "esm_soc_alone_want", "esm_mood_lonely", "c_age", "sex"))

PLcovidDat = as.data.frame(PLcovidDat)
ESMcovidDat = as.data.frame(ESMcovidDat)

#Amount of stressors - this data cannot be imputed due to the conditional nature of the questionnaire
H2a_amount <- lm(delta_aloneprop ~ stressors_amount + c_age + sex, data = PLcovidDat, na.action = na.omit)
summary(H2a_amount)

##
## Call:
## lm(formula = delta_aloneprop ~ stressors_amount + c_age + sex,
## data = PLcovidDat, na.action = na.omit)
##
## Residuals:
## Min 1Q Median 3Q Max
## -4.6721 -2.6835 -1.9241 -0.4752 21.6264
##
## Coefficients:
## Estimate Std. Error t value Pr(>|t|)
## (Intercept) 2.83437 2.54142 1.115 0.268
## stressors_amount 0.06112 0.24030 0.254 0.800
## c_age -0.31435 0.32358 -0.971 0.334
## sex2 1.79277 2.50339 0.716 0.476
##
## Residual standard error: 5.809 on 84 degrees of freedom
## (12 observations deleted due to missingness)
## Multiple R-squared: 0.01567, Adjusted R-squared: -0.01949
## F-statistic: 0.4457 on 3 and 84 DF, p-value: 0.721

H2b_amount <- lme(delta_PA ~ stressors_amount + c_age + sex, data = ESMcovidDat, random = ~1| record_id,
 method = "REML", na.action = na.omit, control = lmeControl(opt='optim'))
summary(H2b_amount)

## Linear mixed-effects model fit by REML
## Data: ESMcovidDat
## AIC BIC logLik
## 265.2785 295.2641 -126.6393
##
## Random effects:
## Formula: ~1 | record_id
## (Intercept) Residual
## StdDev: 0.3469438 0.2392848
##
## Fixed effects: delta_PA ~ stressors_amount + c_age + sex
## Value Std.Error DF t-value p-value
## (Intercept) 0.0869116 0.15768420 1010 0.5511751 0.5816
## stressors_amount -0.0090722 0.01490891 84 -0.6085101 0.5445
## c_age 0.0044802 0.02015913 84 0.2222415 0.8247
## sex1 0.5058347 0.16181302 84 3.1260443 0.0024
## Correlation:
## (Intr) strss_ c_age
## stressors_amount -0.960
## c_age -0.083 -0.036
## sex1 0.065 -0.111 -0.172
##
## Standardized Within-Group Residuals:
## Min Q1 Med Q3 Max
## -4.0660756 -0.3775885 0.0756098 0.4500661 5.2208596
##
## Number of Observations: 1098
## Number of Groups: 88

H2c_amount <- lme(delta_NA ~ stressors_amount + c_age + sex, data = ESMcovidDat, random = ~1| record_id,
 method = "REML", na.action = na.omit, control = lmeControl(opt='optim'))
summary(H2c_amount)

## Linear mixed-effects model fit by REML
## Data: ESMcovidDat
## AIC BIC logLik
## 1569.912 1599.898 -778.9562
##
## Random effects:
## Formula: ~1 | record_id
## (Intercept) Residual
## StdDev: 0.5428997 0.4392593
##
## Fixed effects: delta_NA ~ stressors_amount + c_age + sex
## Value Std.Error DF t-value p-value
## (Intercept) -0.3836085 0.24967904 1010 -1.5364065 0.1248
## stressors_amount 0.0597027 0.02361021 84 2.5286790 0.0133
## c_age -0.0456065 0.03196036 84 -1.4269701 0.1573
## sex1 -0.1604893 0.25895578 84 -0.6197555 0.5371
## Correlation:
## (Intr) strss_ c_age
## stressors_amount -0.960
## c_age -0.087 -0.032
## sex1 0.069 -0.114 -0.175
##
## Standardized Within-Group Residuals:
## Min Q1 Med Q3 Max
## -3.72962700 -0.40346178 -0.08036326 0.29724471 5.67561659
##
## Number of Observations: 1098
## Number of Groups: 88

H2d_amount <- lme(delta_pleasant ~ stressors_amount + c_age + sex, data = ESMcovidDat, random = ~1| record_id,
 method = "REML", na.action = na.omit, control = lmeControl(opt='optim'))
summary(H2d_amount)

## Linear mixed-effects model fit by REML
## Data: ESMcovidDat
## AIC BIC logLik
## 799.2684 829.243 -393.6342
##
## Random effects:
## Formula: ~1 | record_id
## (Intercept) Residual
## StdDev: 0.7011752 0.2947452
##
## Fixed effects: delta_pleasant ~ stressors_amount + c_age + sex
## Value Std.Error DF t-value p-value
## (Intercept) -0.18249984 0.31169447 1008 -0.5855087 0.5583
## stressors_amount 0.03456041 0.02945951 84 1.1731495 0.2441
## c_age -0.06725987 0.03974069 84 -1.6924687 0.0943
## sex1 0.29909848 0.31253455 84 0.9570093 0.3413
## Correlation:
## (Intr) strss_ c_age
## stressors_amount -0.961
## c_age -0.074 -0.044
## sex1 0.056 -0.103 -0.166
##
## Standardized Within-Group Residuals:
## Min Q1 Med Q3 Max
## -8.70706292 -0.31097993 0.03966661 0.39503721 5.10264099
##
## Number of Observations: 1096
## Number of Groups: 88

H2e_amount <- lme(delta_want ~ stressors_amount + c_age + sex, data = ESMcovidDat, random = ~1| record_id,
 method = "REML", na.action = na.omit, control = lmeControl(opt='optim'))
summary(H2e_amount)

## Linear mixed-effects model fit by REML
## Data: ESMcovidDat
## AIC BIC logLik
## 1866.967 1896.926 -927.4837
##
## Random effects:
## Formula: ~1 | record_id
## (Intercept) Residual
## StdDev: 0.9135002 0.4902995
##
## Fixed effects: delta_want ~ stressors_amount + c_age + sex
## Value Std.Error DF t-value p-value
## (Intercept) -0.08990744 0.4096240 1005 -0.2194878 0.8263
## stressors_amount 0.04727478 0.0387216 84 1.2208902 0.2255
## c_age -0.11614066 0.0522860 84 -2.2212588 0.0290
## sex1 0.09730120 0.4146899 84 0.2346360 0.8151
## Correlation:
## (Intr) strss_ c_age
## stressors_amount -0.961
## c_age -0.077 -0.041
## sex1 0.059 -0.106 -0.168
##
## Standardized Within-Group Residuals:
## Min Q1 Med Q3 Max
## -8.33354139 -0.35018928 0.03362337 0.31917034 3.90387624
##
## Number of Observations: 1093
## Number of Groups: 88

H2f_amount <- lme(delta_excluded ~ stressors_amount + c_age + sex, data = ESMcovidDat, random = ~1| record_id,
 method = "REML", na.action = na.omit, control = lmeControl(opt='optim'))
summary(H2f_amount)

## Linear mixed-effects model fit by REML
## Data: ESMcovidDat
## AIC BIC logLik
## 2301.833 2331.786 -1144.917
##
## Random effects:
## Formula: ~1 | record_id
## (Intercept) Residual
## StdDev: 0.4777136 0.6376333
##
## Fixed effects: delta_excluded ~ stressors_amount + c_age + sex
## Value Std.Error DF t-value p-value
## (Intercept) -0.06458240 0.23355473 1004 -0.2765193 0.7822
## stressors_amount 0.02339966 0.02209759 84 1.0589240 0.2927
## c_age -0.03584004 0.03003520 84 -1.1932679 0.2361
## sex1 0.15908671 0.25222939 84 0.6307223 0.5299
## Correlation:
## (Intr) strss_ c_age
## stressors_amount -0.960
## c_age -0.100 -0.021
## sex1 0.084 -0.125 -0.183
##
## Standardized Within-Group Residuals:
## Min Q1 Med Q3 Max
## -3.43967942 -0.20221078 -0.05106434 -0.00907530 7.35656793
##
## Number of Observations: 1092
## Number of Groups: 88

H2g_amount <- lme(delta_loneliness ~ stressors_amount + c_age + sex, data = ESMcovidDat, random = ~1| record_id,
 method = "REML", na.action = na.omit, control = lmeControl(opt='optim'))
summary(H2g_amount)

## Linear mixed-effects model fit by REML
## Data: ESMcovidDat
## AIC BIC logLik
## 3065.172 3095.158 -1526.586
##
## Random effects:
## Formula: ~1 | record_id
## (Intercept) Residual
## StdDev: 0.8513368 0.8847749
##
## Fixed effects: delta_loneliness ~ stressors_amount + c_age + sex
## Value Std.Error DF t-value p-value
## (Intercept) -0.3448138 0.4015120 1010 -0.8587884 0.3907
## stressors_amount 0.0717017 0.0379752 84 1.8881183 0.0625
## c_age -0.0604506 0.0515131 84 -1.1734996 0.2439
## sex1 -0.4682414 0.4245613 84 -1.1028829 0.2732
## Correlation:
## (Intr) strss_ c_age
## stressors_amount -0.960
## c_age -0.094 -0.026
## sex1 0.076 -0.120 -0.179
##
## Standardized Within-Group Residuals:
## Min Q1 Med Q3 Max
## -4.05612860 -0.31351237 -0.10826317 0.05617883 6.44653598
##
## Number of Observations: 1098
## Number of Groups: 88

#Mean burdensomeness of stressors - this data cannot be imputed due to the conditional nature of the questionnaire
H2a_burden <- lm(delta_aloneprop ~ stressors_burden + c_age + sex, data = PLcovidDat, na.action = na.omit)
summary(H2a_burden)

##
## Call:
## lm(formula = delta_aloneprop ~ stressors_burden + c_age + sex,
## data = PLcovidDat, na.action = na.omit)
##
## Residuals:
## Min 1Q Median 3Q Max
## -4.6794 -2.9171 -1.8997 -0.3863 21.7299
##
## Coefficients:
## Estimate Std. Error t value Pr(>|t|)
## (Intercept) 0.9266 4.1371 0.224 0.823
## stressors_burden 0.8018 1.2929 0.620 0.537
## c_age -0.3434 0.3270 -1.050 0.297
## sex2 1.8975 2.4878 0.763 0.448
##
## Residual standard error: 5.798 on 84 degrees of freedom
## (12 observations deleted due to missingness)
## Multiple R-squared: 0.0194, Adjusted R-squared: -0.01562
## F-statistic: 0.5539 on 3 and 84 DF, p-value: 0.6469

H2b_burden <- lme(delta_PA ~ stressors_burden + c_age + sex, data = ESMcovidDat, random = ~1| record_id,
 method = "REML", na.action = na.omit, control = lmeControl(opt='optim'))
summary(H2b_burden)

## Linear mixed-effects model fit by REML
## Data: ESMcovidDat
## AIC BIC logLik
## 260.995 290.9806 -124.4975
##
## Random effects:
## Formula: ~1 | record_id
## (Intercept) Residual
## StdDev: 0.3453624 0.239262
##
## Fixed effects: delta_PA ~ stressors_burden + c_age + sex
## Value Std.Error DF t-value p-value
## (Intercept) 0.2813634 0.25728045 1010 1.0936057 0.2744
## stressors_burden -0.0910371 0.08053726 84 -1.1303722 0.2615
## c_age 0.0075788 0.02030288 84 0.3732883 0.7099
## sex1 0.4920101 0.16020037 84 3.0712169 0.0029
## Correlation:
## (Intr) strss_ c_age
## stressors_burden -0.985
## c_age 0.081 -0.154
## sex1 -0.043 0.018 -0.178
##
## Standardized Within-Group Residuals:
## Min Q1 Med Q3 Max
## -4.06668320 -0.38074208 0.07427849 0.45211754 5.22113803
##
## Number of Observations: 1098
## Number of Groups: 88

H2c_burden <- lme(delta_NA ~ stressors_burden + c_age + sex, data = ESMcovidDat, random = ~1| record_id,
 method = "REML", na.action = na.omit, control = lmeControl(opt='optim'))
summary(H2c_burden)

## Linear mixed-effects model fit by REML
## Data: ESMcovidDat
## AIC BIC logLik
## 1567.034 1597.019 -777.5168
##
## Random effects:
## Formula: ~1 | record_id
## (Intercept) Residual
## StdDev: 0.5444649 0.439275
##
## Fixed effects: delta_NA ~ stressors_burden + c_age + sex
## Value Std.Error DF t-value p-value
## (Intercept) -0.7575059 0.4110220 1010 -1.842981 0.0656
## stressors_burden 0.3114524 0.1286976 84 2.420033 0.0177
## c_age -0.0548839 0.0324018 84 -1.693853 0.0940
## sex1 -0.0762555 0.2579300 84 -0.295644 0.7682
## Correlation:
## (Intr) strss_ c_age
## stressors_burden -0.986
## c_age 0.078 -0.152
## sex1 -0.040 0.015 -0.180
##
## Standardized Within-Group Residuals:
## Min Q1 Med Q3 Max
## -3.73433103 -0.39811864 -0.08021781 0.30366672 5.68640245
##
## Number of Observations: 1098
## Number of Groups: 88

H2d_burden <- lme(delta_pleasant ~ stressors_burden + c_age + sex, data = ESMcovidDat, random = ~1| record_id,
 method = "REML", na.action = na.omit, control = lmeControl(opt='optim'))
summary(H2d_burden)

## Linear mixed-effects model fit by REML
## Data: ESMcovidDat
## AIC BIC logLik
## 795.9187 825.8933 -391.9593
##
## Random effects:
## Formula: ~1 | record_id
## (Intercept) Residual
## StdDev: 0.7009524 0.2947563
##
## Fixed effects: delta_pleasant ~ stressors_burden + c_age + sex
## Value Std.Error DF t-value p-value
## (Intercept) 0.7517247 0.5086406 1008 1.477909 0.1397
## stressors_burden -0.1850849 0.1591337 84 -1.163078 0.2481
## c_age -0.0577295 0.0402045 84 -1.435895 0.1547
## sex1 0.3285795 0.3108536 84 1.057023 0.2935
## Correlation:
## (Intr) strss_ c_age
## stressors_burden -0.985
## c_age 0.087 -0.160
## sex1 -0.050 0.023 -0.173
##
## Standardized Within-Group Residuals:
## Min Q1 Med Q3 Max
## -8.70705704 -0.30569453 0.03880189 0.40173734 5.10877876
##
## Number of Observations: 1096
## Number of Groups: 88

H2e_burden <- lme(delta_want ~ stressors_burden + c_age + sex, data = ESMcovidDat, random = ~1| record_id,
 method = "REML", na.action = na.omit, control = lmeControl(opt='optim'))
summary(H2e_burden)

## Linear mixed-effects model fit by REML
## Data: ESMcovidDat
## AIC BIC logLik
## 1864.18 1894.138 -926.0899
##
## Random effects:
## Formula: ~1 | record_id
## (Intercept) Residual
## StdDev: 0.9160783 0.4903335
##
## Fixed effects: delta_want ~ stressors_burden + c_age + sex
## Value Std.Error DF t-value p-value
## (Intercept) 1.0162862 0.6714186 1005 1.5136401 0.1304
## stressors_burden -0.1987270 0.2101049 84 -0.9458465 0.3469
## c_age -0.1056495 0.0530428 84 -1.9917794 0.0496
## sex1 0.1432991 0.4134896 84 0.3465603 0.7298
## Correlation:
## (Intr) strss_ c_age
## stressors_burden -0.985
## c_age 0.084 -0.157
## sex1 -0.047 0.021 -0.175
##
## Standardized Within-Group Residuals:
## Min Q1 Med Q3 Max
## -8.33302084 -0.34477608 0.02191377 0.32730389 3.90354849
##
## Number of Observations: 1093
## Number of Groups: 88

H2f_burden <- lme(delta_excluded ~ stressors_burden + c_age + sex, data = ESMcovidDat, random = ~1| record_id,
 method = "REML", na.action = na.omit, control = lmeControl(opt='optim'))
summary(H2f_burden)

## Linear mixed-effects model fit by REML
## Data: ESMcovidDat
## AIC BIC logLik
## 2299.212 2329.165 -1143.606
##
## Random effects:
## Formula: ~1 | record_id
## (Intercept) Residual
## StdDev: 0.4790933 0.6377592
##
## Fixed effects: delta_excluded ~ stressors_burden + c_age + sex
## Value Std.Error DF t-value p-value
## (Intercept) -0.04809631 0.3882390 1004 -0.1238832 0.9014
## stressors_burden 0.07028010 0.1217137 84 0.5774213 0.5652
## c_age -0.03768472 0.0304153 84 -1.2390042 0.2188
## sex1 0.19378147 0.2508034 84 0.7726428 0.4419
## Correlation:
## (Intr) strss_ c_age
## stressors_burden -0.986
## c_age 0.069 -0.143
## sex1 -0.030 0.008 -0.186
##
## Standardized Within-Group Residuals:
## Min Q1 Med Q3 Max
## -3.441878880 -0.175046800 -0.041953731 -0.007355592 7.352727544
##
## Number of Observations: 1092
## Number of Groups: 88

H2g_burden <- lme(delta_loneliness ~ stressors_burden + c_age + sex, data = ESMcovidDat, random = ~1| record_id,
 method = "REML", na.action = na.omit, control = lmeControl(opt='optim'))
summary(H2g_burden)

## Linear mixed-effects model fit by REML
## Data: ESMcovidDat
## AIC BIC logLik
## 3058.626 3088.612 -1523.313
##
## Random effects:
## Formula: ~1 | record_id
## (Intercept) Residual
## StdDev: 0.833148 0.8847464
##
## Fixed effects: delta_loneliness ~ stressors_burden + c_age + sex
## Value Std.Error DF t-value p-value
## (Intercept) -1.2986636 0.6499942 1010 -1.9979617 0.0460
## stressors_burden 0.5345753 0.2036474 84 2.6250046 0.0103
## c_age -0.0776097 0.0510932 84 -1.5189844 0.1325
## sex1 -0.3600583 0.4143300 84 -0.8690135 0.3873
## Correlation:
## (Intr) strss_ c_age
## stressors_burden -0.986
## c_age 0.073 -0.147
## sex1 -0.035 0.011 -0.184
##
## Standardized Within-Group Residuals:
## Min Q1 Med Q3 Max
## -4.06019772 -0.30097962 -0.10529372 0.04051485 6.43876107
##
## Number of Observations: 1098
## Number of Groups: 88

#Wave 1 cluster membership - this data cannot be imputed because it is categorical
H2a_cluster <- lm(PL_aloneprop ~ groupMean + c_age + sex, data = PLcovidDat, na.action = na.omit)
summary(H2a_cluster)

##
## Call:
## lm(formula = PL_aloneprop ~ groupMean + c_age + sex, data = PLcovidDat,
## na.action = na.omit)
##
## Residuals:
## Min 1Q Median 3Q Max
## -0.40847 -0.15872 -0.00571 0.13824 0.53824
##
## Coefficients:
## Estimate Std. Error t value Pr(>|t|)
## (Intercept) 0.430009 0.027148 15.839 <2e-16 ***
## groupMean1 0.001298 0.054441 0.024 0.981
## c_age 0.008095 0.012088 0.670 0.505
## sex2 -0.004662 0.090434 -0.052 0.959
## ---
## Signif. codes: 0 '***' 0.001 '**' 0.01 '*' 0.05 '.' 0.1 ' ' 1
##
## Residual standard error: 0.2108 on 84 degrees of freedom
## (12 observations deleted due to missingness)
## Multiple R-squared: 0.005796, Adjusted R-squared: -0.02971
## F-statistic: 0.1632 on 3 and 84 DF, p-value: 0.9208

H2b_cluster <- lme(momentPA ~ groupMean + c_age + sex, data = ESMcovidDat,
 random = ~1| record_id, method = "REML", na.action = na.omit, control = lmeControl(opt='optim'))
summary(H2b_cluster)

## Linear mixed-effects model fit by REML
## Data: ESMcovidDat
## AIC BIC logLik
## 3259.845 3289.831 -1623.923
##
## Random effects:
## Formula: ~1 | record_id
## (Intercept) Residual
## StdDev: 0.9225989 0.9694228
##
## Fixed effects: momentPA ~ groupMean + c_age + sex
## Value Std.Error DF t-value p-value
## (Intercept) 5.231784 0.1307673 1010 40.00836 0.0000
## groupMean1 -0.318793 0.2581318 84 -1.23500 0.2203
## c_age -0.179995 0.0570705 84 -3.15390 0.0022
## sex1 0.094013 0.4578152 84 0.20535 0.8378
## Correlation:
## (Intr) grpMn1 c_age
## groupMean1 -0.368
## c_age -0.314 -0.203
## sex1 -0.127 -0.007 -0.179
##
## Standardized Within-Group Residuals:
## Min Q1 Med Q3 Max
## -4.0139743 -0.4941842 0.1179468 0.6215867 3.0009070
##
## Number of Observations: 1098
## Number of Groups: 88

H2c_cluster <- lme(momentNA ~ groupMean + c_age + sex, data = ESMcovidDat,
 random = ~1| record_id, method = "REML", na.action = na.omit, control = lmeControl(opt='optim'))
summary(H2c_cluster)

## Linear mixed-effects model fit by REML
## Data: ESMcovidDat
## AIC BIC logLik
## 2683.056 2713.042 -1335.528
##
## Random effects:
## Formula: ~1 | record_id
## (Intercept) Residual
## StdDev: 1.072211 0.7224848
##
## Fixed effects: momentNA ~ groupMean + c_age + sex
## Value Std.Error DF t-value p-value
## (Intercept) 1.9459635 0.1454974 1010 13.374558 0.0000
## groupMean1 0.6887891 0.2874957 84 2.395824 0.0188
## c_age 0.0139565 0.0637294 84 0.218996 0.8272
## sex1 -0.0816863 0.4956113 84 -0.164819 0.8695
## Correlation:
## (Intr) grpMn1 c_age
## groupMean1 -0.366
## c_age -0.302 -0.221
## sex1 -0.136 -0.011 -0.170
##
## Standardized Within-Group Residuals:
## Min Q1 Med Q3 Max
## -3.2018795 -0.4703320 -0.1226829 0.3546438 4.6082639
##
## Number of Observations: 1098
## Number of Groups: 88

H2d_cluster <- lme(esm_soc_alone_pleasant ~ groupMean + c_age + sex, data = ESMcovidDat,
 random = ~1| record_id, method = "REML", na.action = na.omit, control = lmeControl(opt='optim'))
summary(H2d_cluster)

## Linear mixed-effects model fit by REML
## Data: ESMcovidDat
## AIC BIC logLik
## 3555.222 3585.197 -1771.611
##
## Random effects:
## Formula: ~1 | record_id
## (Intercept) Residual
## StdDev: 1.239045 1.100292
##
## Fixed effects: esm_soc_alone_pleasant ~ groupMean + c_age + sex
## Value Std.Error DF t-value p-value
## (Intercept) 5.331254 0.1721879 1008 30.961833 0.0000
## groupMean1 -0.200858 0.3399906 84 -0.590776 0.5563
## c_age -0.149970 0.0752662 84 -1.992524 0.0496
## sex1 -0.003351 0.5960173 84 -0.005623 0.9955
## Correlation:
## (Intr) grpMn1 c_age
## groupMean1 -0.367
## c_age -0.308 -0.210
## sex1 -0.131 -0.009 -0.175
##
## Standardized Within-Group Residuals:
## Min Q1 Med Q3 Max
## -4.5875226 -0.4624001 0.1096742 0.4642610 3.5612366
##
## Number of Observations: 1096
## Number of Groups: 88

H2e_cluster <- lme(esm_soc_alone_want ~ groupMean + c_age + sex, data = ESMcovidDat,
 random = ~1| record_id, method = "REML", na.action = na.omit, control = lmeControl(opt='optim'))
summary(H2e_cluster)

## Linear mixed-effects model fit by REML
## Data: ESMcovidDat
## AIC BIC logLik
## 3975.688 4005.646 -1981.844
##
## Random effects:
## Formula: ~1 | record_id
## (Intercept) Residual
## StdDev: 1.304635 1.354348
##
## Fixed effects: esm_soc_alone_want ~ groupMean + c_age + sex
## Value Std.Error DF t-value p-value
## (Intercept) 4.917459 0.1846351 1005 26.633383 0.0000
## groupMean1 -0.017073 0.3644911 84 -0.046840 0.9628
## c_age -0.202721 0.0805950 84 -2.515305 0.0138
## sex1 0.299024 0.6458373 84 0.463002 0.6446
## Correlation:
## (Intr) grpMn1 c_age
## groupMean1 -0.368
## c_age -0.313 -0.203
## sex1 -0.127 -0.007 -0.178
##
## Standardized Within-Group Residuals:
## Min Q1 Med Q3 Max
## -4.07525543 -0.48194696 0.09981628 0.53555813 3.19577045
##
## Number of Observations: 1093
## Number of Groups: 88

H2f_cluster <- lme(esm_soc_alone_excluded ~ groupMean + c_age + sex, data = ESMcovidDat,
 random = ~1| record_id, method = "REML", na.action = na.omit, control = lmeControl(opt='optim'))
summary(H2f_cluster)

## Linear mixed-effects model fit by REML
## Data: ESMcovidDat
## AIC BIC logLik
## 2525.002 2554.954 -1256.501
##
## Random effects:
## Formula: ~1 | record_id
## (Intercept) Residual
## StdDev: 0.6704971 0.6965208
##
## Fixed effects: esm_soc_alone_excluded ~ groupMean + c_age + sex
## Value Std.Error DF t-value p-value
## (Intercept) 1.3236439 0.0949235 1004 13.944316 0.0000
## groupMean1 0.3140692 0.1873470 84 1.676404 0.0974
## c_age 0.0082122 0.0414254 84 0.198239 0.8433
## sex1 0.2063137 0.3319644 84 0.621494 0.5360
## Correlation:
## (Intr) grpMn1 c_age
## groupMean1 -0.368
## c_age -0.313 -0.203
## sex1 -0.127 -0.007 -0.178
##
## Standardized Within-Group Residuals:
## Min Q1 Med Q3 Max
## -3.24047670 -0.17468693 -0.05209960 -0.02147846 6.70252051
##
## Number of Observations: 1092
## Number of Groups: 88

H2g_cluster <- lme(esm_mood_lonely ~ groupMean + c_age + sex, data = ESMcovidDat,
 random = ~1| record_id, method = "REML", na.action = na.omit, control = lmeControl(opt='optim'))
summary(H2g_cluster)

## Linear mixed-effects model fit by REML
## Data: ESMcovidDat
## AIC BIC logLik
## 3705.415 3735.401 -1846.708
##
## Random effects:
## Formula: ~1 | record_id
## (Intercept) Residual
## StdDev: 1.138585 1.18782
##
## Fixed effects: esm_mood_lonely ~ groupMean + c_age + sex
## Value Std.Error DF t-value p-value
## (Intercept) 1.8891463 0.1612295 1010 11.717127 0.0000
## groupMean1 0.9738216 0.3182662 84 3.059771 0.0030
## c_age -0.0505195 0.0703700 84 -0.717912 0.4748
## sex1 0.1341779 0.5641803 84 0.237828 0.8126
## Correlation:
## (Intr) grpMn1 c_age
## groupMean1 -0.368
## c_age -0.313 -0.203
## sex1 -0.127 -0.007 -0.178
##
## Standardized Within-Group Residuals:
## Min Q1 Med Q3 Max
## -3.2843985 -0.3985512 -0.1470607 0.1619249 4.7812215
##
## Number of Observations: 1098
## Number of Groups: 88

#Plot significant effects
effH2g_cluster <- as.data.frame(effect(term="groupMean",mod=H2g_cluster))[,c('groupMean','fit','se','lower','upper')]
effH2g_cluster$groupMean <- as.factor(effH2g_cluster$groupMean)
plot_H2g_cluster <- ggplot(data=effH2g_cluster, aes(x = groupMean, y= fit)) +
 geom_bar(stat="summary", fun="mean", position="dodge", width=.5)+
 #geom_signif(comparisons=list(c("0", "1")), map_signif_level=TRUE)+
 labs(title = "T1 Loneliness \n by T0 Cluster Membership", x= "T0 Social Withdrawal Cluster",
 y="T1 Loneliness") + theme_classic() + theme(axis.title=element_text(size=10)) + theme(plot.title=element_text(size=12)) +
 theme(plot.title=element_text(face="bold")) + theme(plot.title=element_text(hjust=0.5)) + scale_x_discrete(labels=c("Positive", "Negative"))
plot_H2g_cluster


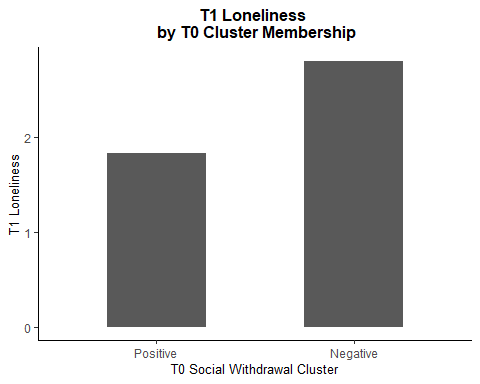


ggsave("H2g_cluster.png")

## Saving 5 x 4 in image

######################################################################################################
##MULTIPLE IMPUTATION OF SSL, VPV AND MSPSS DATA##

#Imputing the SSL and VPV
PLdat_Wave_I = PLdat[PLdat$wave=='wave1',]

data.ESM.aggregated = PLdat_Wave_I[,c("record_id", "ssl_invite", "ssl_visit", "ssl_affection", "ssl_comfort", "ssl_compliment", "ssl_interest",
 "ssl_offerhelp", "ssl_ease", "ssl_advice", "ssl_confidence", "ssl_askhelp", "ssl_positivecharacter",
 "vpv_meetfriends", "vpv_recogniseemotion", "vpv_class", "vpv_help", "vpv_easycontact", "vpv_hurt", "vpv_questions",
 "vpv_sensitivities", "vpv_standup", "vpv_said", "vpv_seekcontact", "vpv_wishes", "vpv_getalong", "vpv_interest",
 "vpv_talkconcerns", "vpv_adapt", "vpv_goodcontact", "vpv_learn", "age", "sex")]

init=mice(data.ESM.aggregated, maxit=0)
meth=init$method
predM=init$predictorMatrix
meth[c("ssl_invite", "ssl_visit","ssl_affection", "ssl_comfort", "ssl_compliment", "ssl_interest", "ssl_offerhelp", "ssl_ease", "ssl_advice",
 "ssl_confidence", "ssl_askhelp", "ssl_positivecharacter", "vpv_meetfriends", "vpv_recogniseemotion", "vpv_class", "vpv_help", "vpv_easycontact",
 "vpv_hurt", "vpv_questions", "vpv_sensitivities", "vpv_standup", "vpv_said", "vpv_seekcontact", "vpv_wishes", "vpv_getalong", "vpv_interest",
 "vpv_talkconcerns", "vpv_adapt", "vpv_goodcontact", "vpv_learn")]="pmm"
data.imp.Wave.I <- mice(data.ESM.aggregated, method=meth, predictorMatrix = predM, m = 20)

##
## iter imp variable
## 1 1 ssl_invite ssl_visit ssl_affection ssl_comfort ssl_compliment ssl_interest ssl_offerhelp ssl_ease ssl_advice ssl_confidence ssl_askhelp ssl_positivecharacter vpv_meetfriends vpv_recogniseemotion vpv_class vpv_help vpv_easycontact vpv_hurt vpv_questions vpv_sensitivities vpv_standup vpv_said vpv_seekcontact vpv_wishes vpv_getalong vpv_interest vpv_talkconcerns vpv_adapt vpv_goodcontact vpv_learn
## 1 2 ssl_invite ssl_visit ssl_affection ssl_comfort ssl_compliment ssl_interest ssl_offerhelp ssl_ease ssl_advice ssl_confidence ssl_askhelp ssl_positivecharacter vpv_meetfriends vpv_recogniseemotion vpv_class vpv_help vpv_easycontact vpv_hurt vpv_questions vpv_sensitivities vpv_standup vpv_said vpv_seekcontact vpv_wishes vpv_getalong vpv_interest vpv_talkconcerns vpv_adapt vpv_goodcontact vpv_learn
## 1 3 ssl_invite ssl_visit ssl_affection ssl_comfort ssl_compliment ssl_interest ssl_offerhelp ssl_ease ssl_advice ssl_confidence ssl_askhelp ssl_positivecharacter vpv_meetfriends vpv_recogniseemotion vpv_class vpv_help vpv_easycontact vpv_hurt vpv_questions vpv_sensitivities vpv_standup vpv_said vpv_seekcontact vpv_wishes vpv_getalong vpv_interest vpv_talkconcerns vpv_adapt vpv_goodcontact vpv_learn
## 1 4 ssl_invite ssl_visit ssl_affection ssl_comfort ssl_compliment ssl_interest ssl_offerhelp ssl_ease ssl_advice ssl_confidence ssl_askhelp ssl_positivecharacter vpv_meetfriends vpv_recogniseemotion vpv_class vpv_help vpv_easycontact vpv_hurt vpv_questions vpv_sensitivities vpv_standup vpv_said vpv_seekcontact vpv_wishes vpv_getalong vpv_interest vpv_talkconcerns vpv_adapt vpv_goodcontact vpv_learn
## 1 5 ssl_invite ssl_visit ssl_affection ssl_comfort ssl_compliment ssl_interest ssl_offerhelp ssl_ease ssl_advice ssl_confidence ssl_askhelp ssl_positivecharacter vpv_meetfriends vpv_recogniseemotion vpv_class vpv_help vpv_easycontact vpv_hurt vpv_questions vpv_sensitivities vpv_standup vpv_said vpv_seekcontact vpv_wishes vpv_getalong vpv_interest vpv_talkconcerns vpv_adapt vpv_goodcontact vpv_learn
## 1 6 ssl_invite ssl_visit ssl_affection ssl_comfort ssl_compliment ssl_interest ssl_offerhelp ssl_ease ssl_advice ssl_confidence ssl_askhelp ssl_positivecharacter vpv_meetfriends vpv_recogniseemotion vpv_class vpv_help vpv_easycontact vpv_hurt vpv_questions vpv_sensitivities vpv_standup vpv_said vpv_seekcontact vpv_wishes vpv_getalong vpv_interest vpv_talkconcerns vpv_adapt vpv_goodcontact vpv_learn
## 1 7 ssl_invite ssl_visit ssl_affection ssl_comfort ssl_compliment ssl_interest ssl_offerhelp ssl_ease ssl_advice ssl_confidence ssl_askhelp ssl_positivecharacter vpv_meetfriends vpv_recogniseemotion vpv_class vpv_help vpv_easycontact vpv_hurt vpv_questions vpv_sensitivities vpv_standup vpv_said vpv_seekcontact vpv_wishes vpv_getalong vpv_interest vpv_talkconcerns vpv_adapt vpv_goodcontact vpv_learn
## 1 8 ssl_invite ssl_visit ssl_affection ssl_comfort ssl_compliment ssl_interest ssl_offerhelp ssl_ease ssl_advice ssl_confidence ssl_askhelp ssl_positivecharacter vpv_meetfriends vpv_recogniseemotion vpv_class vpv_help vpv_easycontact vpv_hurt vpv_questions vpv_sensitivities vpv_standup vpv_said vpv_seekcontact vpv_wishes vpv_getalong vpv_interest vpv_talkconcerns vpv_adapt vpv_goodcontact vpv_learn
## 1 9 ssl_invite ssl_visit ssl_affection ssl_comfort ssl_compliment ssl_interest ssl_offerhelp ssl_ease ssl_advice ssl_confidence ssl_askhelp ssl_positivecharacter vpv_meetfriends vpv_recogniseemotion vpv_class vpv_help vpv_easycontact vpv_hurt vpv_questions vpv_sensitivities vpv_standup vpv_said vpv_seekcontact vpv_wishes vpv_getalong vpv_interest vpv_talkconcerns vpv_adapt vpv_goodcontact vpv_learn
## 1 10 ssl_invite ssl_visit ssl_affection ssl_comfort ssl_compliment ssl_interest ssl_offerhelp ssl_ease ssl_advice ssl_confidence ssl_askhelp ssl_positivecharacter vpv_meetfriends vpv_recogniseemotion vpv_class vpv_help vpv_easycontact vpv_hurt vpv_questions vpv_sensitivities vpv_standup vpv_said vpv_seekcontact vpv_wishes vpv_getalong vpv_interest vpv_talkconcerns vpv_adapt vpv_goodcontact vpv_learn
## 1 11 ssl_invite ssl_visit ssl_affection ssl_comfort ssl_compliment ssl_interest ssl_offerhelp ssl_ease ssl_advice ssl_confidence ssl_askhelp ssl_positivecharacter vpv_meetfriends vpv_recogniseemotion vpv_class vpv_help vpv_easycontact vpv_hurt vpv_questions vpv_sensitivities vpv_standup vpv_said vpv_seekcontact vpv_wishes vpv_getalong vpv_interest vpv_talkconcerns vpv_adapt vpv_goodcontact vpv_learn
## 1 12 ssl_invite ssl_visit ssl_affection ssl_comfort ssl_compliment ssl_interest ssl_offerhelp ssl_ease ssl_advice ssl_confidence ssl_askhelp ssl_positivecharacter vpv_meetfriends vpv_recogniseemotion vpv_class vpv_help vpv_easycontact vpv_hurt vpv_questions vpv_sensitivities vpv_standup vpv_said vpv_seekcontact vpv_wishes vpv_getalong vpv_interest vpv_talkconcerns vpv_adapt vpv_goodcontact vpv_learn
## 1 13 ssl_invite ssl_visit ssl_affection ssl_comfort ssl_compliment ssl_interest ssl_offerhelp ssl_ease ssl_advice ssl_confidence ssl_askhelp ssl_positivecharacter vpv_meetfriends vpv_recogniseemotion vpv_class vpv_help vpv_easycontact vpv_hurt vpv_questions vpv_sensitivities vpv_standup vpv_said vpv_seekcontact vpv_wishes vpv_getalong vpv_interest vpv_talkconcerns vpv_adapt vpv_goodcontact vpv_learn
## 1 14 ssl_invite ssl_visit ssl_affection ssl_comfort ssl_compliment ssl_interest ssl_offerhelp ssl_ease ssl_advice ssl_confidence ssl_askhelp ssl_positivecharacter vpv_meetfriends vpv_recogniseemotion vpv_class vpv_help vpv_easycontact vpv_hurt vpv_questions vpv_sensitivities vpv_standup vpv_said vpv_seekcontact vpv_wishes vpv_getalong vpv_interest vpv_talkconcerns vpv_adapt vpv_goodcontact vpv_learn
## 1 15 ssl_invite ssl_visit ssl_affection ssl_comfort ssl_compliment ssl_interest ssl_offerhelp ssl_ease ssl_advice ssl_confidence ssl_askhelp ssl_positivecharacter vpv_meetfriends vpv_recogniseemotion vpv_class vpv_help vpv_easycontact vpv_hurt vpv_questions vpv_sensitivities vpv_standup vpv_said vpv_seekcontact vpv_wishes vpv_getalong vpv_interest vpv_talkconcerns vpv_adapt vpv_goodcontact vpv_learn
## 1 16 ssl_invite ssl_visit ssl_affection ssl_comfort ssl_compliment ssl_interest ssl_offerhelp ssl_ease ssl_advice ssl_confidence ssl_askhelp ssl_positivecharacter vpv_meetfriends vpv_recogniseemotion vpv_class vpv_help vpv_easycontact vpv_hurt vpv_questions vpv_sensitivities vpv_standup vpv_said vpv_seekcontact vpv_wishes vpv_getalong vpv_interest vpv_talkconcerns vpv_adapt vpv_goodcontact vpv_learn
## 1 17 ssl_invite ssl_visit ssl_affection ssl_comfort ssl_compliment ssl_interest ssl_offerhelp ssl_ease ssl_advice ssl_confidence ssl_askhelp ssl_positivecharacter vpv_meetfriends vpv_recogniseemotion vpv_class vpv_help vpv_easycontact vpv_hurt vpv_questions vpv_sensitivities vpv_standup vpv_said vpv_seekcontact vpv_wishes vpv_getalong vpv_interest vpv_talkconcerns vpv_adapt vpv_goodcontact vpv_learn
## 1 18 ssl_invite ssl_visit ssl_affection ssl_comfort ssl_compliment ssl_interest ssl_offerhelp ssl_ease ssl_advice ssl_confidence ssl_askhelp ssl_positivecharacter vpv_meetfriends vpv_recogniseemotion vpv_class vpv_help vpv_easycontact vpv_hurt vpv_questions vpv_sensitivities vpv_standup vpv_said vpv_seekcontact vpv_wishes vpv_getalong vpv_interest vpv_talkconcerns vpv_adapt vpv_goodcontact vpv_learn
## 1 19 ssl_invite ssl_visit ssl_affection ssl_comfort ssl_compliment ssl_interest ssl_offerhelp ssl_ease ssl_advice ssl_confidence ssl_askhelp ssl_positivecharacter vpv_meetfriends vpv_recogniseemotion vpv_class vpv_help vpv_easycontact vpv_hurt vpv_questions vpv_sensitivities vpv_standup vpv_said vpv_seekcontact vpv_wishes vpv_getalong vpv_interest vpv_talkconcerns vpv_adapt vpv_goodcontact vpv_learn
## 1 20 ssl_invite ssl_visit ssl_affection ssl_comfort ssl_compliment ssl_interest ssl_offerhelp ssl_ease ssl_advice ssl_confidence ssl_askhelp ssl_positivecharacter vpv_meetfriends vpv_recogniseemotion vpv_class vpv_help vpv_easycontact vpv_hurt vpv_questions vpv_sensitivities vpv_standup vpv_said vpv_seekcontact vpv_wishes vpv_getalong vpv_interest vpv_talkconcerns vpv_adapt vpv_goodcontact vpv_learn
## 2 1 ssl_invite ssl_visit ssl_affection ssl_comfort ssl_compliment ssl_interest ssl_offerhelp ssl_ease ssl_advice ssl_confidence ssl_askhelp ssl_positivecharacter vpv_meetfriends vpv_recogniseemotion vpv_class vpv_help vpv_easycontact vpv_hurt vpv_questions vpv_sensitivities vpv_standup vpv_said vpv_seekcontact vpv_wishes vpv_getalong vpv_interest vpv_talkconcerns vpv_adapt vpv_goodcontact vpv_learn
## 2 2 ssl_invite ssl_visit ssl_affection ssl_comfort ssl_compliment ssl_interest ssl_offerhelp ssl_ease ssl_advice ssl_confidence ssl_askhelp ssl_positivecharacter vpv_meetfriends vpv_recogniseemotion vpv_class vpv_help vpv_easycontact vpv_hurt vpv_questions vpv_sensitivities vpv_standup vpv_said vpv_seekcontact vpv_wishes vpv_getalong vpv_interest vpv_talkconcerns vpv_adapt vpv_goodcontact vpv_learn
## 2 3 ssl_invite ssl_visit ssl_affection ssl_comfort ssl_compliment ssl_interest ssl_offerhelp ssl_ease ssl_advice ssl_confidence ssl_askhelp ssl_positivecharacter vpv_meetfriends vpv_recogniseemotion vpv_class vpv_help vpv_easycontact vpv_hurt vpv_questions vpv_sensitivities vpv_standup vpv_said vpv_seekcontact vpv_wishes vpv_getalong vpv_interest vpv_talkconcerns vpv_adapt vpv_goodcontact vpv_learn
## 2 4 ssl_invite ssl_visit ssl_affection ssl_comfort ssl_compliment ssl_interest ssl_offerhelp ssl_ease ssl_advice ssl_confidence ssl_askhelp ssl_positivecharacter vpv_meetfriends vpv_recogniseemotion vpv_class vpv_help vpv_easycontact vpv_hurt vpv_questions vpv_sensitivities vpv_standup vpv_said vpv_seekcontact vpv_wishes vpv_getalong vpv_interest vpv_talkconcerns vpv_adapt vpv_goodcontact vpv_learn
## 2 5 ssl_invite ssl_visit ssl_affection ssl_comfort ssl_compliment ssl_interest ssl_offerhelp ssl_ease ssl_advice ssl_confidence ssl_askhelp ssl_positivecharacter vpv_meetfriends vpv_recogniseemotion vpv_class vpv_help vpv_easycontact vpv_hurt vpv_questions vpv_sensitivities vpv_standup vpv_said vpv_seekcontact vpv_wishes vpv_getalong vpv_interest vpv_talkconcerns vpv_adapt vpv_goodcontact vpv_learn
## 2 6 ssl_invite ssl_visit ssl_affection ssl_comfort ssl_compliment ssl_interest ssl_offerhelp ssl_ease ssl_advice ssl_confidence ssl_askhelp ssl_positivecharacter vpv_meetfriends vpv_recogniseemotion vpv_class vpv_help vpv_easycontact vpv_hurt vpv_questions vpv_sensitivities vpv_standup vpv_said vpv_seekcontact vpv_wishes vpv_getalong vpv_interest vpv_talkconcerns vpv_adapt vpv_goodcontact vpv_learn
## 2 7 ssl_invite ssl_visit ssl_affection ssl_comfort ssl_compliment ssl_interest ssl_offerhelp ssl_ease ssl_advice ssl_confidence ssl_askhelp ssl_positivecharacter vpv_meetfriends vpv_recogniseemotion vpv_class vpv_help vpv_easycontact vpv_hurt vpv_questions vpv_sensitivities vpv_standup vpv_said vpv_seekcontact vpv_wishes vpv_getalong vpv_interest vpv_talkconcerns vpv_adapt vpv_goodcontact vpv_learn
## 2 8 ssl_invite ssl_visit ssl_affection ssl_comfort ssl_compliment ssl_interest ssl_offerhelp ssl_ease ssl_advice ssl_confidence ssl_askhelp ssl_positivecharacter vpv_meetfriends vpv_recogniseemotion vpv_class vpv_help vpv_easycontact vpv_hurt vpv_questions vpv_sensitivities vpv_standup vpv_said vpv_seekcontact vpv_wishes vpv_getalong vpv_interest vpv_talkconcerns vpv_adapt vpv_goodcontact vpv_learn
## 2 9 ssl_invite ssl_visit ssl_affection ssl_comfort ssl_compliment ssl_interest ssl_offerhelp ssl_ease ssl_advice ssl_confidence ssl_askhelp ssl_positivecharacter vpv_meetfriends vpv_recogniseemotion vpv_class vpv_help vpv_easycontact vpv_hurt vpv_questions vpv_sensitivities vpv_standup vpv_said vpv_seekcontact vpv_wishes vpv_getalong vpv_interest vpv_talkconcerns vpv_adapt vpv_goodcontact vpv_learn
## 2 10 ssl_invite ssl_visit ssl_affection ssl_comfort ssl_compliment ssl_interest ssl_offerhelp ssl_ease ssl_advice ssl_confidence ssl_askhelp ssl_positivecharacter vpv_meetfriends vpv_recogniseemotion vpv_class vpv_help vpv_easycontact vpv_hurt vpv_questions vpv_sensitivities vpv_standup vpv_said vpv_seekcontact vpv_wishes vpv_getalong vpv_interest vpv_talkconcerns vpv_adapt vpv_goodcontact vpv_learn
## 2 11 ssl_invite ssl_visit ssl_affection ssl_comfort ssl_compliment ssl_interest ssl_offerhelp ssl_ease ssl_advice ssl_confidence ssl_askhelp ssl_positivecharacter vpv_meetfriends vpv_recogniseemotion vpv_class vpv_help vpv_easycontact vpv_hurt vpv_questions vpv_sensitivities vpv_standup vpv_said vpv_seekcontact vpv_wishes vpv_getalong vpv_interest vpv_talkconcerns vpv_adapt vpv_goodcontact vpv_learn
## 2 12 ssl_invite ssl_visit ssl_affection ssl_comfort ssl_compliment ssl_interest ssl_offerhelp ssl_ease ssl_advice ssl_confidence ssl_askhelp ssl_positivecharacter vpv_meetfriends vpv_recogniseemotion vpv_class vpv_help vpv_easycontact vpv_hurt vpv_questions vpv_sensitivities vpv_standup vpv_said vpv_seekcontact vpv_wishes vpv_getalong vpv_interest vpv_talkconcerns vpv_adapt vpv_goodcontact vpv_learn
## 2 13 ssl_invite ssl_visit ssl_affection ssl_comfort ssl_compliment ssl_interest ssl_offerhelp ssl_ease ssl_advice ssl_confidence ssl_askhelp ssl_positivecharacter vpv_meetfriends vpv_recogniseemotion vpv_class vpv_help vpv_easycontact vpv_hurt vpv_questions vpv_sensitivities vpv_standup vpv_said vpv_seekcontact vpv_wishes vpv_getalong vpv_interest vpv_talkconcerns vpv_adapt vpv_goodcontact vpv_learn
## 2 14 ssl_invite ssl_visit ssl_affection ssl_comfort ssl_compliment ssl_interest ssl_offerhelp ssl_ease ssl_advice ssl_confidence ssl_askhelp ssl_positivecharacter vpv_meetfriends vpv_recogniseemotion vpv_class vpv_help vpv_easycontact vpv_hurt vpv_questions vpv_sensitivities vpv_standup vpv_said vpv_seekcontact vpv_wishes vpv_getalong vpv_interest vpv_talkconcerns vpv_adapt vpv_goodcontact vpv_learn
## 2 15 ssl_invite ssl_visit ssl_affection ssl_comfort ssl_compliment ssl_interest ssl_offerhelp ssl_ease ssl_advice ssl_confidence ssl_askhelp ssl_positivecharacter vpv_meetfriends vpv_recogniseemotion vpv_class vpv_help vpv_easycontact vpv_hurt vpv_questions vpv_sensitivities vpv_standup vpv_said vpv_seekcontact vpv_wishes vpv_getalong vpv_interest vpv_talkconcerns vpv_adapt vpv_goodcontact vpv_learn
## 2 16 ssl_invite ssl_visit ssl_affection ssl_comfort ssl_compliment ssl_interest ssl_offerhelp ssl_ease ssl_advice ssl_confidence ssl_askhelp ssl_positivecharacter vpv_meetfriends vpv_recogniseemotion vpv_class vpv_help vpv_easycontact vpv_hurt vpv_questions vpv_sensitivities vpv_standup vpv_said vpv_seekcontact vpv_wishes vpv_getalong vpv_interest vpv_talkconcerns vpv_adapt vpv_goodcontact vpv_learn
## 2 17 ssl_invite ssl_visit ssl_affection ssl_comfort ssl_compliment ssl_interest ssl_offerhelp ssl_ease ssl_advice ssl_confidence ssl_askhelp ssl_positivecharacter vpv_meetfriends vpv_recogniseemotion vpv_class vpv_help vpv_easycontact vpv_hurt vpv_questions vpv_sensitivities vpv_standup vpv_said vpv_seekcontact vpv_wishes vpv_getalong vpv_interest vpv_talkconcerns vpv_adapt vpv_goodcontact vpv_learn
## 2 18 ssl_invite ssl_visit ssl_affection ssl_comfort ssl_compliment ssl_interest ssl_offerhelp ssl_ease ssl_advice ssl_confidence ssl_askhelp ssl_positivecharacter vpv_meetfriends vpv_recogniseemotion vpv_class vpv_help vpv_easycontact vpv_hurt vpv_questions vpv_sensitivities vpv_standup vpv_said vpv_seekcontact vpv_wishes vpv_getalong vpv_interest vpv_talkconcerns vpv_adapt vpv_goodcontact vpv_learn
## 2 19 ssl_invite ssl_visit ssl_affection ssl_comfort ssl_compliment ssl_interest ssl_offerhelp ssl_ease ssl_advice ssl_confidence ssl_askhelp ssl_positivecharacter vpv_meetfriends vpv_recogniseemotion vpv_class vpv_help vpv_easycontact vpv_hurt vpv_questions vpv_sensitivities vpv_standup vpv_said vpv_seekcontact vpv_wishes vpv_getalong vpv_interest vpv_talkconcerns vpv_adapt vpv_goodcontact vpv_learn
## 2 20 ssl_invite ssl_visit ssl_affection ssl_comfort ssl_compliment ssl_interest ssl_offerhelp ssl_ease ssl_advice ssl_confidence ssl_askhelp ssl_positivecharacter vpv_meetfriends vpv_recogniseemotion vpv_class vpv_help vpv_easycontact vpv_hurt vpv_questions vpv_sensitivities vpv_standup vpv_said vpv_seekcontact vpv_wishes vpv_getalong vpv_interest vpv_talkconcerns vpv_adapt vpv_goodcontact vpv_learn
## 3 1 ssl_invite ssl_visit ssl_affection ssl_comfort ssl_compliment ssl_interest ssl_offerhelp ssl_ease ssl_advice ssl_confidence ssl_askhelp ssl_positivecharacter vpv_meetfriends vpv_recogniseemotion vpv_class vpv_help vpv_easycontact vpv_hurt vpv_questions vpv_sensitivities vpv_standup vpv_said vpv_seekcontact vpv_wishes vpv_getalong vpv_interest vpv_talkconcerns vpv_adapt vpv_goodcontact vpv_learn
## 3 2 ssl_invite ssl_visit ssl_affection ssl_comfort ssl_compliment ssl_interest ssl_offerhelp ssl_ease ssl_advice ssl_confidence ssl_askhelp ssl_positivecharacter vpv_meetfriends vpv_recogniseemotion vpv_class vpv_help vpv_easycontact vpv_hurt vpv_questions vpv_sensitivities vpv_standup vpv_said vpv_seekcontact vpv_wishes vpv_getalong vpv_interest vpv_talkconcerns vpv_adapt vpv_goodcontact vpv_learn
## 3 3 ssl_invite ssl_visit ssl_affection ssl_comfort ssl_compliment ssl_interest ssl_offerhelp ssl_ease ssl_advice ssl_confidence ssl_askhelp ssl_positivecharacter vpv_meetfriends vpv_recogniseemotion vpv_class vpv_help vpv_easycontact vpv_hurt vpv_questions vpv_sensitivities vpv_standup vpv_said vpv_seekcontact vpv_wishes vpv_getalong vpv_interest vpv_talkconcerns vpv_adapt vpv_goodcontact vpv_learn
## 3 4 ssl_invite ssl_visit ssl_affection ssl_comfort ssl_compliment ssl_interest ssl_offerhelp ssl_ease ssl_advice ssl_confidence ssl_askhelp ssl_positivecharacter vpv_meetfriends vpv_recogniseemotion vpv_class vpv_help vpv_easycontact vpv_hurt vpv_questions vpv_sensitivities vpv_standup vpv_said vpv_seekcontact vpv_wishes vpv_getalong vpv_interest vpv_talkconcerns vpv_adapt vpv_goodcontact vpv_learn
## 3 5 ssl_invite ssl_visit ssl_affection ssl_comfort ssl_compliment ssl_interest ssl_offerhelp ssl_ease ssl_advice ssl_confidence ssl_askhelp ssl_positivecharacter vpv_meetfriends vpv_recogniseemotion vpv_class vpv_help vpv_easycontact vpv_hurt vpv_questions vpv_sensitivities vpv_standup vpv_said vpv_seekcontact vpv_wishes vpv_getalong vpv_interest vpv_talkconcerns vpv_adapt vpv_goodcontact vpv_learn
## 3 6 ssl_invite ssl_visit ssl_affection ssl_comfort ssl_compliment ssl_interest ssl_offerhelp ssl_ease ssl_advice ssl_confidence ssl_askhelp ssl_positivecharacter vpv_meetfriends vpv_recogniseemotion vpv_class vpv_help vpv_easycontact vpv_hurt vpv_questions vpv_sensitivities vpv_standup vpv_said vpv_seekcontact vpv_wishes vpv_getalong vpv_interest vpv_talkconcerns vpv_adapt vpv_goodcontact vpv_learn
## 3 7 ssl_invite ssl_visit ssl_affection ssl_comfort ssl_compliment ssl_interest ssl_offerhelp ssl_ease ssl_advice ssl_confidence ssl_askhelp ssl_positivecharacter vpv_meetfriends vpv_recogniseemotion vpv_class vpv_help vpv_easycontact vpv_hurt vpv_questions vpv_sensitivities vpv_standup vpv_said vpv_seekcontact vpv_wishes vpv_getalong vpv_interest vpv_talkconcerns vpv_adapt vpv_goodcontact vpv_learn
## 3 8 ssl_invite ssl_visit ssl_affection ssl_comfort ssl_compliment ssl_interest ssl_offerhelp ssl_ease ssl_advice ssl_confidence ssl_askhelp ssl_positivecharacter vpv_meetfriends vpv_recogniseemotion vpv_class vpv_help vpv_easycontact vpv_hurt vpv_questions vpv_sensitivities vpv_standup vpv_said vpv_seekcontact vpv_wishes vpv_getalong vpv_interest vpv_talkconcerns vpv_adapt vpv_goodcontact vpv_learn
## 3 9 ssl_invite ssl_visit ssl_affection ssl_comfort ssl_compliment ssl_interest ssl_offerhelp ssl_ease ssl_advice ssl_confidence ssl_askhelp ssl_positivecharacter vpv_meetfriends vpv_recogniseemotion vpv_class vpv_help vpv_easycontact vpv_hurt vpv_questions vpv_sensitivities vpv_standup vpv_said vpv_seekcontact vpv_wishes vpv_getalong vpv_interest vpv_talkconcerns vpv_adapt vpv_goodcontact vpv_learn
## 3 10 ssl_invite ssl_visit ssl_affection ssl_comfort ssl_compliment ssl_interest ssl_offerhelp ssl_ease ssl_advice ssl_confidence ssl_askhelp ssl_positivecharacter vpv_meetfriends vpv_recogniseemotion vpv_class vpv_help vpv_easycontact vpv_hurt vpv_questions vpv_sensitivities vpv_standup vpv_said vpv_seekcontact vpv_wishes vpv_getalong vpv_interest vpv_talkconcerns vpv_adapt vpv_goodcontact vpv_learn
## 3 11 ssl_invite ssl_visit ssl_affection ssl_comfort ssl_compliment ssl_interest ssl_offerhelp ssl_ease ssl_advice ssl_confidence ssl_askhelp ssl_positivecharacter vpv_meetfriends vpv_recogniseemotion vpv_class vpv_help vpv_easycontact vpv_hurt vpv_questions vpv_sensitivities vpv_standup vpv_said vpv_seekcontact vpv_wishes vpv_getalong vpv_interest vpv_talkconcerns vpv_adapt vpv_goodcontact vpv_learn
## 3 12 ssl_invite ssl_visit ssl_affection ssl_comfort ssl_compliment ssl_interest ssl_offerhelp ssl_ease ssl_advice ssl_confidence ssl_askhelp ssl_positivecharacter vpv_meetfriends vpv_recogniseemotion vpv_class vpv_help vpv_easycontact vpv_hurt vpv_questions vpv_sensitivities vpv_standup vpv_said vpv_seekcontact vpv_wishes vpv_getalong vpv_interest vpv_talkconcerns vpv_adapt vpv_goodcontact vpv_learn
## 3 13 ssl_invite ssl_visit ssl_affection ssl_comfort ssl_compliment ssl_interest ssl_offerhelp ssl_ease ssl_advice ssl_confidence ssl_askhelp ssl_positivecharacter vpv_meetfriends vpv_recogniseemotion vpv_class vpv_help vpv_easycontact vpv_hurt vpv_questions vpv_sensitivities vpv_standup vpv_said vpv_seekcontact vpv_wishes vpv_getalong vpv_interest vpv_talkconcerns vpv_adapt vpv_goodcontact vpv_learn
## 3 14 ssl_invite ssl_visit ssl_affection ssl_comfort ssl_compliment ssl_interest ssl_offerhelp ssl_ease ssl_advice ssl_confidence ssl_askhelp ssl_positivecharacter vpv_meetfriends vpv_recogniseemotion vpv_class vpv_help vpv_easycontact vpv_hurt vpv_questions vpv_sensitivities vpv_standup vpv_said vpv_seekcontact vpv_wishes vpv_getalong vpv_interest vpv_talkconcerns vpv_adapt vpv_goodcontact vpv_learn
## 3 15 ssl_invite ssl_visit ssl_affection ssl_comfort ssl_compliment ssl_interest ssl_offerhelp ssl_ease ssl_advice ssl_confidence ssl_askhelp ssl_positivecharacter vpv_meetfriends vpv_recogniseemotion vpv_class vpv_help vpv_easycontact vpv_hurt vpv_questions vpv_sensitivities vpv_standup vpv_said vpv_seekcontact vpv_wishes vpv_getalong vpv_interest vpv_talkconcerns vpv_adapt vpv_goodcontact vpv_learn
## 3 16 ssl_invite ssl_visit ssl_affection ssl_comfort ssl_compliment ssl_interest ssl_offerhelp ssl_ease ssl_advice ssl_confidence ssl_askhelp ssl_positivecharacter vpv_meetfriends vpv_recogniseemotion vpv_class vpv_help vpv_easycontact vpv_hurt vpv_questions vpv_sensitivities vpv_standup vpv_said vpv_seekcontact vpv_wishes vpv_getalong vpv_interest vpv_talkconcerns vpv_adapt vpv_goodcontact vpv_learn
## 3 17 ssl_invite ssl_visit ssl_affection ssl_comfort ssl_compliment ssl_interest ssl_offerhelp ssl_ease ssl_advice ssl_confidence ssl_askhelp ssl_positivecharacter vpv_meetfriends vpv_recogniseemotion vpv_class vpv_help vpv_easycontact vpv_hurt vpv_questions vpv_sensitivities vpv_standup vpv_said vpv_seekcontact vpv_wishes vpv_getalong vpv_interest vpv_talkconcerns vpv_adapt vpv_goodcontact vpv_learn
## 3 18 ssl_invite ssl_visit ssl_affection ssl_comfort ssl_compliment ssl_interest ssl_offerhelp ssl_ease ssl_advice ssl_confidence ssl_askhelp ssl_positivecharacter vpv_meetfriends vpv_recogniseemotion vpv_class vpv_help vpv_easycontact vpv_hurt vpv_questions vpv_sensitivities vpv_standup vpv_said vpv_seekcontact vpv_wishes vpv_getalong vpv_interest vpv_talkconcerns vpv_adapt vpv_goodcontact vpv_learn
## 3 19 ssl_invite ssl_visit ssl_affection ssl_comfort ssl_compliment ssl_interest ssl_offerhelp ssl_ease ssl_advice ssl_confidence ssl_askhelp ssl_positivecharacter vpv_meetfriends vpv_recogniseemotion vpv_class vpv_help vpv_easycontact vpv_hurt vpv_questions vpv_sensitivities vpv_standup vpv_said vpv_seekcontact vpv_wishes vpv_getalong vpv_interest vpv_talkconcerns vpv_adapt vpv_goodcontact vpv_learn
## 3 20 ssl_invite ssl_visit ssl_affection ssl_comfort ssl_compliment ssl_interest ssl_offerhelp ssl_ease ssl_advice ssl_confidence ssl_askhelp ssl_positivecharacter vpv_meetfriends vpv_recogniseemotion vpv_class vpv_help vpv_easycontact vpv_hurt vpv_questions vpv_sensitivities vpv_standup vpv_said vpv_seekcontact vpv_wishes vpv_getalong vpv_interest vpv_talkconcerns vpv_adapt vpv_goodcontact vpv_learn
## 4 1 ssl_invite ssl_visit ssl_affection ssl_comfort ssl_compliment ssl_interest ssl_offerhelp ssl_ease ssl_advice ssl_confidence ssl_askhelp ssl_positivecharacter vpv_meetfriends vpv_recogniseemotion vpv_class vpv_help vpv_easycontact vpv_hurt vpv_questions vpv_sensitivities vpv_standup vpv_said vpv_seekcontact vpv_wishes vpv_getalong vpv_interest vpv_talkconcerns vpv_adapt vpv_goodcontact vpv_learn
## 4 2 ssl_invite ssl_visit ssl_affection ssl_comfort ssl_compliment ssl_interest ssl_offerhelp ssl_ease ssl_advice ssl_confidence ssl_askhelp ssl_positivecharacter vpv_meetfriends vpv_recogniseemotion vpv_class vpv_help vpv_easycontact vpv_hurt vpv_questions vpv_sensitivities vpv_standup vpv_said vpv_seekcontact vpv_wishes vpv_getalong vpv_interest vpv_talkconcerns vpv_adapt vpv_goodcontact vpv_learn
## 4 3 ssl_invite ssl_visit ssl_affection ssl_comfort ssl_compliment ssl_interest ssl_offerhelp ssl_ease ssl_advice ssl_confidence ssl_askhelp ssl_positivecharacter vpv_meetfriends vpv_recogniseemotion vpv_class vpv_help vpv_easycontact vpv_hurt vpv_questions vpv_sensitivities vpv_standup vpv_said vpv_seekcontact vpv_wishes vpv_getalong vpv_interest vpv_talkconcerns vpv_adapt vpv_goodcontact vpv_learn
## 4 4 ssl_invite ssl_visit ssl_affection ssl_comfort ssl_compliment ssl_interest ssl_offerhelp ssl_ease ssl_advice ssl_confidence ssl_askhelp ssl_positivecharacter vpv_meetfriends vpv_recogniseemotion vpv_class vpv_help vpv_easycontact vpv_hurt vpv_questions vpv_sensitivities vpv_standup vpv_said vpv_seekcontact vpv_wishes vpv_getalong vpv_interest vpv_talkconcerns vpv_adapt vpv_goodcontact vpv_learn
## 4 5 ssl_invite ssl_visit ssl_affection ssl_comfort ssl_compliment ssl_interest ssl_offerhelp ssl_ease ssl_advice ssl_confidence ssl_askhelp ssl_positivecharacter vpv_meetfriends vpv_recogniseemotion vpv_class vpv_help vpv_easycontact vpv_hurt vpv_questions vpv_sensitivities vpv_standup vpv_said vpv_seekcontact vpv_wishes vpv_getalong vpv_interest vpv_talkconcerns vpv_adapt vpv_goodcontact vpv_learn
## 4 6 ssl_invite ssl_visit ssl_affection ssl_comfort ssl_compliment ssl_interest ssl_offerhelp ssl_ease ssl_advice ssl_confidence ssl_askhelp ssl_positivecharacter vpv_meetfriends vpv_recogniseemotion vpv_class vpv_help vpv_easycontact vpv_hurt vpv_questions vpv_sensitivities vpv_standup vpv_said vpv_seekcontact vpv_wishes vpv_getalong vpv_interest vpv_talkconcerns vpv_adapt vpv_goodcontact vpv_learn
## 4 7 ssl_invite ssl_visit ssl_affection ssl_comfort ssl_compliment ssl_interest ssl_offerhelp ssl_ease ssl_advice ssl_confidence ssl_askhelp ssl_positivecharacter vpv_meetfriends vpv_recogniseemotion vpv_class vpv_help vpv_easycontact vpv_hurt vpv_questions vpv_sensitivities vpv_standup vpv_said vpv_seekcontact vpv_wishes vpv_getalong vpv_interest vpv_talkconcerns vpv_adapt vpv_goodcontact vpv_learn
## 4 8 ssl_invite ssl_visit ssl_affection ssl_comfort ssl_compliment ssl_interest ssl_offerhelp ssl_ease ssl_advice ssl_confidence ssl_askhelp ssl_positivecharacter vpv_meetfriends vpv_recogniseemotion vpv_class vpv_help vpv_easycontact vpv_hurt vpv_questions vpv_sensitivities vpv_standup vpv_said vpv_seekcontact vpv_wishes vpv_getalong vpv_interest vpv_talkconcerns vpv_adapt vpv_goodcontact vpv_learn
## 4 9 ssl_invite ssl_visit ssl_affection ssl_comfort ssl_compliment ssl_interest ssl_offerhelp ssl_ease ssl_advice ssl_confidence ssl_askhelp ssl_positivecharacter vpv_meetfriends vpv_recogniseemotion vpv_class vpv_help vpv_easycontact vpv_hurt vpv_questions vpv_sensitivities vpv_standup vpv_said vpv_seekcontact vpv_wishes vpv_getalong vpv_interest vpv_talkconcerns vpv_adapt vpv_goodcontact vpv_learn
## 4 10 ssl_invite ssl_visit ssl_affection ssl_comfort ssl_compliment ssl_interest ssl_offerhelp ssl_ease ssl_advice ssl_confidence ssl_askhelp ssl_positivecharacter vpv_meetfriends vpv_recogniseemotion vpv_class vpv_help vpv_easycontact vpv_hurt vpv_questions vpv_sensitivities vpv_standup vpv_said vpv_seekcontact vpv_wishes vpv_getalong vpv_interest vpv_talkconcerns vpv_adapt vpv_goodcontact vpv_learn
## 4 11 ssl_invite ssl_visit ssl_affection ssl_comfort ssl_compliment ssl_interest ssl_offerhelp ssl_ease ssl_advice ssl_confidence ssl_askhelp ssl_positivecharacter vpv_meetfriends vpv_recogniseemotion vpv_class vpv_help vpv_easycontact vpv_hurt vpv_questions vpv_sensitivities vpv_standup vpv_said vpv_seekcontact vpv_wishes vpv_getalong vpv_interest vpv_talkconcerns vpv_adapt vpv_goodcontact vpv_learn
## 4 12 ssl_invite ssl_visit ssl_affection ssl_comfort ssl_compliment ssl_interest ssl_offerhelp ssl_ease ssl_advice ssl_confidence ssl_askhelp ssl_positivecharacter vpv_meetfriends vpv_recogniseemotion vpv_class vpv_help vpv_easycontact vpv_hurt vpv_questions vpv_sensitivities vpv_standup vpv_said vpv_seekcontact vpv_wishes vpv_getalong vpv_interest vpv_talkconcerns vpv_adapt vpv_goodcontact vpv_learn
## 4 13 ssl_invite ssl_visit ssl_affection ssl_comfort ssl_compliment ssl_interest ssl_offerhelp ssl_ease ssl_advice ssl_confidence ssl_askhelp ssl_positivecharacter vpv_meetfriends vpv_recogniseemotion vpv_class vpv_help vpv_easycontact vpv_hurt vpv_questions vpv_sensitivities vpv_standup vpv_said vpv_seekcontact vpv_wishes vpv_getalong vpv_interest vpv_talkconcerns vpv_adapt vpv_goodcontact vpv_learn
## 4 14 ssl_invite ssl_visit ssl_affection ssl_comfort ssl_compliment ssl_interest ssl_offerhelp ssl_ease ssl_advice ssl_confidence ssl_askhelp ssl_positivecharacter vpv_meetfriends vpv_recogniseemotion vpv_class vpv_help vpv_easycontact vpv_hurt vpv_questions vpv_sensitivities vpv_standup vpv_said vpv_seekcontact vpv_wishes vpv_getalong vpv_interest vpv_talkconcerns vpv_adapt vpv_goodcontact vpv_learn
## 4 15 ssl_invite ssl_visit ssl_affection ssl_comfort ssl_compliment ssl_interest ssl_offerhelp ssl_ease ssl_advice ssl_confidence ssl_askhelp ssl_positivecharacter vpv_meetfriends vpv_recogniseemotion vpv_class vpv_help vpv_easycontact vpv_hurt vpv_questions vpv_sensitivities vpv_standup vpv_said vpv_seekcontact vpv_wishes vpv_getalong vpv_interest vpv_talkconcerns vpv_adapt vpv_goodcontact vpv_learn
## 4 16 ssl_invite ssl_visit ssl_affection ssl_comfort ssl_compliment ssl_interest ssl_offerhelp ssl_ease ssl_advice ssl_confidence ssl_askhelp ssl_positivecharacter vpv_meetfriends vpv_recogniseemotion vpv_class vpv_help vpv_easycontact vpv_hurt vpv_questions vpv_sensitivities vpv_standup vpv_said vpv_seekcontact vpv_wishes vpv_getalong vpv_interest vpv_talkconcerns vpv_adapt vpv_goodcontact vpv_learn
## 4 17 ssl_invite ssl_visit ssl_affection ssl_comfort ssl_compliment ssl_interest ssl_offerhelp ssl_ease ssl_advice ssl_confidence ssl_askhelp ssl_positivecharacter vpv_meetfriends vpv_recogniseemotion vpv_class vpv_help vpv_easycontact vpv_hurt vpv_questions vpv_sensitivities vpv_standup vpv_said vpv_seekcontact vpv_wishes vpv_getalong vpv_interest vpv_talkconcerns vpv_adapt vpv_goodcontact vpv_learn
## 4 18 ssl_invite ssl_visit ssl_affection ssl_comfort ssl_compliment ssl_interest ssl_offerhelp ssl_ease ssl_advice ssl_confidence ssl_askhelp ssl_positivecharacter vpv_meetfriends vpv_recogniseemotion vpv_class vpv_help vpv_easycontact vpv_hurt vpv_questions vpv_sensitivities vpv_standup vpv_said vpv_seekcontact vpv_wishes vpv_getalong vpv_interest vpv_talkconcerns vpv_adapt vpv_goodcontact vpv_learn
## 4 19 ssl_invite ssl_visit ssl_affection ssl_comfort ssl_compliment ssl_interest ssl_offerhelp ssl_ease ssl_advice ssl_confidence ssl_askhelp ssl_positivecharacter vpv_meetfriends vpv_recogniseemotion vpv_class vpv_help vpv_easycontact vpv_hurt vpv_questions vpv_sensitivities vpv_standup vpv_said vpv_seekcontact vpv_wishes vpv_getalong vpv_interest vpv_talkconcerns vpv_adapt vpv_goodcontact vpv_learn
## 4 20 ssl_invite ssl_visit ssl_affection ssl_comfort ssl_compliment ssl_interest ssl_offerhelp ssl_ease ssl_advice ssl_confidence ssl_askhelp ssl_positivecharacter vpv_meetfriends vpv_recogniseemotion vpv_class vpv_help vpv_easycontact vpv_hurt vpv_questions vpv_sensitivities vpv_standup vpv_said vpv_seekcontact vpv_wishes vpv_getalong vpv_interest vpv_talkconcerns vpv_adapt vpv_goodcontact vpv_learn
## 5 1 ssl_invite ssl_visit ssl_affection ssl_comfort ssl_compliment ssl_interest ssl_offerhelp ssl_ease ssl_advice ssl_confidence ssl_askhelp ssl_positivecharacter vpv_meetfriends vpv_recogniseemotion vpv_class vpv_help vpv_easycontact vpv_hurt vpv_questions vpv_sensitivities vpv_standup vpv_said vpv_seekcontact vpv_wishes vpv_getalong vpv_interest vpv_talkconcerns vpv_adapt vpv_goodcontact vpv_learn
## 5 2 ssl_invite ssl_visit ssl_affection ssl_comfort ssl_compliment ssl_interest ssl_offerhelp ssl_ease ssl_advice ssl_confidence ssl_askhelp ssl_positivecharacter vpv_meetfriends vpv_recogniseemotion vpv_class vpv_help vpv_easycontact vpv_hurt vpv_questions vpv_sensitivities vpv_standup vpv_said vpv_seekcontact vpv_wishes vpv_getalong vpv_interest vpv_talkconcerns vpv_adapt vpv_goodcontact vpv_learn
## 5 3 ssl_invite ssl_visit ssl_affection ssl_comfort ssl_compliment ssl_interest ssl_offerhelp ssl_ease ssl_advice ssl_confidence ssl_askhelp ssl_positivecharacter vpv_meetfriends vpv_recogniseemotion vpv_class vpv_help vpv_easycontact vpv_hurt vpv_questions vpv_sensitivities vpv_standup vpv_said vpv_seekcontact vpv_wishes vpv_getalong vpv_interest vpv_talkconcerns vpv_adapt vpv_goodcontact vpv_learn
## 5 4 ssl_invite ssl_visit ssl_affection ssl_comfort ssl_compliment ssl_interest ssl_offerhelp ssl_ease ssl_advice ssl_confidence ssl_askhelp ssl_positivecharacter vpv_meetfriends vpv_recogniseemotion vpv_class vpv_help vpv_easycontact vpv_hurt vpv_questions vpv_sensitivities vpv_standup vpv_said vpv_seekcontact vpv_wishes vpv_getalong vpv_interest vpv_talkconcerns vpv_adapt vpv_goodcontact vpv_learn
## 5 5 ssl_invite ssl_visit ssl_affection ssl_comfort ssl_compliment ssl_interest ssl_offerhelp ssl_ease ssl_advice ssl_confidence ssl_askhelp ssl_positivecharacter vpv_meetfriends vpv_recogniseemotion vpv_class vpv_help vpv_easycontact vpv_hurt vpv_questions vpv_sensitivities vpv_standup vpv_said vpv_seekcontact vpv_wishes vpv_getalong vpv_interest vpv_talkconcerns vpv_adapt vpv_goodcontact vpv_learn
## 5 6 ssl_invite ssl_visit ssl_affection ssl_comfort ssl_compliment ssl_interest ssl_offerhelp ssl_ease ssl_advice ssl_confidence ssl_askhelp ssl_positivecharacter vpv_meetfriends vpv_recogniseemotion vpv_class vpv_help vpv_easycontact vpv_hurt vpv_questions vpv_sensitivities vpv_standup vpv_said vpv_seekcontact vpv_wishes vpv_getalong vpv_interest vpv_talkconcerns vpv_adapt vpv_goodcontact vpv_learn
## 5 7 ssl_invite ssl_visit ssl_affection ssl_comfort ssl_compliment ssl_interest ssl_offerhelp ssl_ease ssl_advice ssl_confidence ssl_askhelp ssl_positivecharacter vpv_meetfriends vpv_recogniseemotion vpv_class vpv_help vpv_easycontact vpv_hurt vpv_questions vpv_sensitivities vpv_standup vpv_said vpv_seekcontact vpv_wishes vpv_getalong vpv_interest vpv_talkconcerns vpv_adapt vpv_goodcontact vpv_learn
## 5 8 ssl_invite ssl_visit ssl_affection ssl_comfort ssl_compliment ssl_interest ssl_offerhelp ssl_ease ssl_advice ssl_confidence ssl_askhelp ssl_positivecharacter vpv_meetfriends vpv_recogniseemotion vpv_class vpv_help vpv_easycontact vpv_hurt vpv_questions vpv_sensitivities vpv_standup vpv_said vpv_seekcontact vpv_wishes vpv_getalong vpv_interest vpv_talkconcerns vpv_adapt vpv_goodcontact vpv_learn
## 5 9 ssl_invite ssl_visit ssl_affection ssl_comfort ssl_compliment ssl_interest ssl_offerhelp ssl_ease ssl_advice ssl_confidence ssl_askhelp ssl_positivecharacter vpv_meetfriends vpv_recogniseemotion vpv_class vpv_help vpv_easycontact vpv_hurt vpv_questions vpv_sensitivities vpv_standup vpv_said vpv_seekcontact vpv_wishes vpv_getalong vpv_interest vpv_talkconcerns vpv_adapt vpv_goodcontact vpv_learn
## 5 10 ssl_invite ssl_visit ssl_affection ssl_comfort ssl_compliment ssl_interest ssl_offerhelp ssl_ease ssl_advice ssl_confidence ssl_askhelp ssl_positivecharacter vpv_meetfriends vpv_recogniseemotion vpv_class vpv_help vpv_easycontact vpv_hurt vpv_questions vpv_sensitivities vpv_standup vpv_said vpv_seekcontact vpv_wishes vpv_getalong vpv_interest vpv_talkconcerns vpv_adapt vpv_goodcontact vpv_learn
## 5 11 ssl_invite ssl_visit ssl_affection ssl_comfort ssl_compliment ssl_interest ssl_offerhelp ssl_ease ssl_advice ssl_confidence ssl_askhelp ssl_positivecharacter vpv_meetfriends vpv_recogniseemotion vpv_class vpv_help vpv_easycontact vpv_hurt vpv_questions vpv_sensitivities vpv_standup vpv_said vpv_seekcontact vpv_wishes vpv_getalong vpv_interest vpv_talkconcerns vpv_adapt vpv_goodcontact vpv_learn
## 5 12 ssl_invite ssl_visit ssl_affection ssl_comfort ssl_compliment ssl_interest ssl_offerhelp ssl_ease ssl_advice ssl_confidence ssl_askhelp ssl_positivecharacter vpv_meetfriends vpv_recogniseemotion vpv_class vpv_help vpv_easycontact vpv_hurt vpv_questions vpv_sensitivities vpv_standup vpv_said vpv_seekcontact vpv_wishes vpv_getalong vpv_interest vpv_talkconcerns vpv_adapt vpv_goodcontact vpv_learn
## 5 13 ssl_invite ssl_visit ssl_affection ssl_comfort ssl_compliment ssl_interest ssl_offerhelp ssl_ease ssl_advice ssl_confidence ssl_askhelp ssl_positivecharacter vpv_meetfriends vpv_recogniseemotion vpv_class vpv_help vpv_easycontact vpv_hurt vpv_questions vpv_sensitivities vpv_standup vpv_said vpv_seekcontact vpv_wishes vpv_getalong vpv_interest vpv_talkconcerns vpv_adapt vpv_goodcontact vpv_learn
## 5 14 ssl_invite ssl_visit ssl_affection ssl_comfort ssl_compliment ssl_interest ssl_offerhelp ssl_ease ssl_advice ssl_confidence ssl_askhelp ssl_positivecharacter vpv_meetfriends vpv_recogniseemotion vpv_class vpv_help vpv_easycontact vpv_hurt vpv_questions vpv_sensitivities vpv_standup vpv_said vpv_seekcontact vpv_wishes vpv_getalong vpv_interest vpv_talkconcerns vpv_adapt vpv_goodcontact vpv_learn
## 5 15 ssl_invite ssl_visit ssl_affection ssl_comfort ssl_compliment ssl_interest ssl_offerhelp ssl_ease ssl_advice ssl_confidence ssl_askhelp ssl_positivecharacter vpv_meetfriends vpv_recogniseemotion vpv_class vpv_help vpv_easycontact vpv_hurt vpv_questions vpv_sensitivities vpv_standup vpv_said vpv_seekcontact vpv_wishes vpv_getalong vpv_interest vpv_talkconcerns vpv_adapt vpv_goodcontact vpv_learn
## 5 16 ssl_invite ssl_visit ssl_affection ssl_comfort ssl_compliment ssl_interest ssl_offerhelp ssl_ease ssl_advice ssl_confidence ssl_askhelp ssl_positivecharacter vpv_meetfriends vpv_recogniseemotion vpv_class vpv_help vpv_easycontact vpv_hurt vpv_questions vpv_sensitivities vpv_standup vpv_said vpv_seekcontact vpv_wishes vpv_getalong vpv_interest vpv_talkconcerns vpv_adapt vpv_goodcontact vpv_learn
## 5 17 ssl_invite ssl_visit ssl_affection ssl_comfort ssl_compliment ssl_interest ssl_offerhelp ssl_ease ssl_advice ssl_confidence ssl_askhelp ssl_positivecharacter vpv_meetfriends vpv_recogniseemotion vpv_class vpv_help vpv_easycontact vpv_hurt vpv_questions vpv_sensitivities vpv_standup vpv_said vpv_seekcontact vpv_wishes vpv_getalong vpv_interest vpv_talkconcerns vpv_adapt vpv_goodcontact vpv_learn
## 5 18 ssl_invite ssl_visit ssl_affection ssl_comfort ssl_compliment ssl_interest ssl_offerhelp ssl_ease ssl_advice ssl_confidence ssl_askhelp ssl_positivecharacter vpv_meetfriends vpv_recogniseemotion vpv_class vpv_help vpv_easycontact vpv_hurt vpv_questions vpv_sensitivities vpv_standup vpv_said vpv_seekcontact vpv_wishes vpv_getalong vpv_interest vpv_talkconcerns vpv_adapt vpv_goodcontact vpv_learn
## 5 19 ssl_invite ssl_visit ssl_affection ssl_comfort ssl_compliment ssl_interest ssl_offerhelp ssl_ease ssl_advice ssl_confidence ssl_askhelp ssl_positivecharacter vpv_meetfriends vpv_recogniseemotion vpv_class vpv_help vpv_easycontact vpv_hurt vpv_questions vpv_sensitivities vpv_standup vpv_said vpv_seekcontact vpv_wishes vpv_getalong vpv_interest vpv_talkconcerns vpv_adapt vpv_goodcontact vpv_learn
## 5 20 ssl_invite ssl_visit ssl_affection ssl_comfort ssl_compliment ssl_interest ssl_offerhelp ssl_ease ssl_advice ssl_confidence ssl_askhelp ssl_positivecharacter vpv_meetfriends vpv_recogniseemotion vpv_class vpv_help vpv_easycontact vpv_hurt vpv_questions vpv_sensitivities vpv_standup vpv_said vpv_seekcontact vpv_wishes vpv_getalong vpv_interest vpv_talkconcerns vpv_adapt vpv_goodcontact vpv_learn

#Imputing the MSPSS
PLdat_Wave_Covid = PLdat[PLdat$wave=='covid',]

data.ESM.aggregated = PLdat_Wave_Covid[,c("record_id", "mspss_person_help", "mspss_person_joy", "mspss_family_help", "mspss_family_support",
 "mspss_person_comfort", "mspss_friends_help", "mspss_friends_count", "mspss_family_talk", "mspss_friends_joy",
 "mspss_person_feelings", "mspss_family_decide", "mspss_friends_talk", "age", "sex")]

init=mice(data.ESM.aggregated, maxit=0)
meth=init$method
predM=init$predictorMatrix
meth[c("mspss_person_help", "mspss_person_joy", "mspss_family_help", "mspss_family_support", "mspss_person_comfort", "mspss_friends_help",
 "mspss_friends_count", "mspss_family_talk", "mspss_friends_joy", "mspss_person_feelings", "mspss_family_decide", "mspss_friends_talk")]="pmm"
data.imp.Wave.Covid <- mice(data.ESM.aggregated, method=meth, predictorMatrix = predM, m = 20)

##
## iter imp variable
## 1 1 mspss_person_help mspss_person_joy mspss_family_help mspss_family_support mspss_person_comfort mspss_friends_help mspss_friends_count mspss_family_talk mspss_friends_joy mspss_person_feelings mspss_family_decide mspss_friends_talk
## 1 2 mspss_person_help mspss_person_joy mspss_family_help mspss_family_support mspss_person_comfort mspss_friends_help mspss_friends_count mspss_family_talk mspss_friends_joy mspss_person_feelings mspss_family_decide mspss_friends_talk
## 1 3 mspss_person_help mspss_person_joy mspss_family_help mspss_family_support mspss_person_comfort mspss_friends_help mspss_friends_count mspss_family_talk mspss_friends_joy mspss_person_feelings mspss_family_decide mspss_friends_talk
## 1 4 mspss_person_help mspss_person_joy mspss_family_help mspss_family_support mspss_person_comfort mspss_friends_help mspss_friends_count mspss_family_talk mspss_friends_joy mspss_person_feelings mspss_family_decide mspss_friends_talk
## 1 5 mspss_person_help mspss_person_joy mspss_family_help mspss_family_support mspss_person_comfort mspss_friends_help mspss_friends_count mspss_family_talk mspss_friends_joy mspss_person_feelings mspss_family_decide mspss_friends_talk
## 1 6 mspss_person_help mspss_person_joy mspss_family_help mspss_family_support mspss_person_comfort mspss_friends_help mspss_friends_count mspss_family_talk mspss_friends_joy mspss_person_feelings mspss_family_decide mspss_friends_talk
## 1 7 mspss_person_help mspss_person_joy mspss_family_help mspss_family_support mspss_person_comfort mspss_friends_help mspss_friends_count mspss_family_talk mspss_friends_joy mspss_person_feelings mspss_family_decide mspss_friends_talk
## 1 8 mspss_person_help mspss_person_joy mspss_family_help mspss_family_support mspss_person_comfort mspss_friends_help mspss_friends_count mspss_family_talk mspss_friends_joy mspss_person_feelings mspss_family_decide mspss_friends_talk
## 1 9 mspss_person_help mspss_person_joy mspss_family_help mspss_family_support mspss_person_comfort mspss_friends_help mspss_friends_count mspss_family_talk mspss_friends_joy mspss_person_feelings mspss_family_decide mspss_friends_talk
## 1 10 mspss_person_help mspss_person_joy mspss_family_help mspss_family_support mspss_person_comfort mspss_friends_help mspss_friends_count mspss_family_talk mspss_friends_joy mspss_person_feelings mspss_family_decide mspss_friends_talk
## 1 11 mspss_person_help mspss_person_joy mspss_family_help mspss_family_support mspss_person_comfort mspss_friends_help mspss_friends_count mspss_family_talk mspss_friends_joy mspss_person_feelings mspss_family_decide mspss_friends_talk
## 1 12 mspss_person_help mspss_person_joy mspss_family_help mspss_family_support mspss_person_comfort mspss_friends_help mspss_friends_count mspss_family_talk mspss_friends_joy mspss_person_feelings mspss_family_decide mspss_friends_talk
## 1 13 mspss_person_help mspss_person_joy mspss_family_help mspss_family_support mspss_person_comfort mspss_friends_help mspss_friends_count mspss_family_talk mspss_friends_joy mspss_person_feelings mspss_family_decide mspss_friends_talk
## 1 14 mspss_person_help mspss_person_joy mspss_family_help mspss_family_support mspss_person_comfort mspss_friends_help mspss_friends_count mspss_family_talk mspss_friends_joy mspss_person_feelings mspss_family_decide mspss_friends_talk
## 1 15 mspss_person_help mspss_person_joy mspss_family_help mspss_family_support mspss_person_comfort mspss_friends_help mspss_friends_count mspss_family_talk mspss_friends_joy mspss_person_feelings mspss_family_decide mspss_friends_talk
## 1 16 mspss_person_help mspss_person_joy mspss_family_help mspss_family_support mspss_person_comfort mspss_friends_help mspss_friends_count mspss_family_talk mspss_friends_joy mspss_person_feelings mspss_family_decide mspss_friends_talk
## 1 17 mspss_person_help mspss_person_joy mspss_family_help mspss_family_support mspss_person_comfort mspss_friends_help mspss_friends_count mspss_family_talk mspss_friends_joy mspss_person_feelings mspss_family_decide mspss_friends_talk
## 1 18 mspss_person_help mspss_person_joy mspss_family_help mspss_family_support mspss_person_comfort mspss_friends_help mspss_friends_count mspss_family_talk mspss_friends_joy mspss_person_feelings mspss_family_decide mspss_friends_talk
## 1 19 mspss_person_help mspss_person_joy mspss_family_help mspss_family_support mspss_person_comfort mspss_friends_help mspss_friends_count mspss_family_talk mspss_friends_joy mspss_person_feelings mspss_family_decide mspss_friends_talk
## 1 20 mspss_person_help mspss_person_joy mspss_family_help mspss_family_support mspss_person_comfort mspss_friends_help mspss_friends_count mspss_family_talk mspss_friends_joy mspss_person_feelings mspss_family_decide mspss_friends_talk
## 2 1 mspss_person_help mspss_person_joy mspss_family_help mspss_family_support mspss_person_comfort mspss_friends_help mspss_friends_count mspss_family_talk mspss_friends_joy mspss_person_feelings mspss_family_decide mspss_friends_talk
## 2 2 mspss_person_help mspss_person_joy mspss_family_help mspss_family_support mspss_person_comfort mspss_friends_help mspss_friends_count mspss_family_talk mspss_friends_joy mspss_person_feelings mspss_family_decide mspss_friends_talk
## 2 3 mspss_person_help mspss_person_joy mspss_family_help mspss_family_support mspss_person_comfort mspss_friends_help mspss_friends_count mspss_family_talk mspss_friends_joy mspss_person_feelings mspss_family_decide mspss_friends_talk
## 2 4 mspss_person_help mspss_person_joy mspss_family_help mspss_family_support mspss_person_comfort mspss_friends_help mspss_friends_count mspss_family_talk mspss_friends_joy mspss_person_feelings mspss_family_decide mspss_friends_talk
## 2 5 mspss_person_help mspss_person_joy mspss_family_help mspss_family_support mspss_person_comfort mspss_friends_help mspss_friends_count mspss_family_talk mspss_friends_joy mspss_person_feelings mspss_family_decide mspss_friends_talk
## 2 6 mspss_person_help mspss_person_joy mspss_family_help mspss_family_support mspss_person_comfort mspss_friends_help mspss_friends_count mspss_family_talk mspss_friends_joy mspss_person_feelings mspss_family_decide mspss_friends_talk
## 2 7 mspss_person_help mspss_person_joy mspss_family_help mspss_family_support mspss_person_comfort mspss_friends_help mspss_friends_count mspss_family_talk mspss_friends_joy mspss_person_feelings mspss_family_decide mspss_friends_talk
## 2 8 mspss_person_help mspss_person_joy mspss_family_help mspss_family_support mspss_person_comfort mspss_friends_help mspss_friends_count mspss_family_talk mspss_friends_joy mspss_person_feelings mspss_family_decide mspss_friends_talk
## 2 9 mspss_person_help mspss_person_joy mspss_family_help mspss_family_support mspss_person_comfort mspss_friends_help mspss_friends_count mspss_family_talk mspss_friends_joy mspss_person_feelings mspss_family_decide mspss_friends_talk
## 2 10 mspss_person_help mspss_person_joy mspss_family_help mspss_family_support mspss_person_comfort mspss_friends_help mspss_friends_count mspss_family_talk mspss_friends_joy mspss_person_feelings mspss_family_decide mspss_friends_talk
## 2 11 mspss_person_help mspss_person_joy mspss_family_help mspss_family_support mspss_person_comfort mspss_friends_help mspss_friends_count mspss_family_talk mspss_friends_joy mspss_person_feelings mspss_family_decide mspss_friends_talk
## 2 12 mspss_person_help mspss_person_joy mspss_family_help mspss_family_support mspss_person_comfort mspss_friends_help mspss_friends_count mspss_family_talk mspss_friends_joy mspss_person_feelings mspss_family_decide mspss_friends_talk
## 2 13 mspss_person_help mspss_person_joy mspss_family_help mspss_family_support mspss_person_comfort mspss_friends_help mspss_friends_count mspss_family_talk mspss_friends_joy mspss_person_feelings mspss_family_decide mspss_friends_talk
## 2 14 mspss_person_help mspss_person_joy mspss_family_help mspss_family_support mspss_person_comfort mspss_friends_help mspss_friends_count mspss_family_talk mspss_friends_joy mspss_person_feelings mspss_family_decide mspss_friends_talk
## 2 15 mspss_person_help mspss_person_joy mspss_family_help mspss_family_support mspss_person_comfort mspss_friends_help mspss_friends_count mspss_family_talk mspss_friends_joy mspss_person_feelings mspss_family_decide mspss_friends_talk
## 2 16 mspss_person_help mspss_person_joy mspss_family_help mspss_family_support mspss_person_comfort mspss_friends_help mspss_friends_count mspss_family_talk mspss_friends_joy mspss_person_feelings mspss_family_decide mspss_friends_talk
## 2 17 mspss_person_help mspss_person_joy mspss_family_help mspss_family_support mspss_person_comfort mspss_friends_help mspss_friends_count mspss_family_talk mspss_friends_joy mspss_person_feelings mspss_family_decide mspss_friends_talk
## 2 18 mspss_person_help mspss_person_joy mspss_family_help mspss_family_support mspss_person_comfort mspss_friends_help mspss_friends_count mspss_family_talk mspss_friends_joy mspss_person_feelings mspss_family_decide mspss_friends_talk
## 2 19 mspss_person_help mspss_person_joy mspss_family_help mspss_family_support mspss_person_comfort mspss_friends_help mspss_friends_count mspss_family_talk mspss_friends_joy mspss_person_feelings mspss_family_decide mspss_friends_talk
## 2 20 mspss_person_help mspss_person_joy mspss_family_help mspss_family_support mspss_person_comfort mspss_friends_help mspss_friends_count mspss_family_talk mspss_friends_joy mspss_person_feelings mspss_family_decide mspss_friends_talk
## 3 1 mspss_person_help mspss_person_joy mspss_family_help mspss_family_support mspss_person_comfort mspss_friends_help mspss_friends_count mspss_family_talk mspss_friends_joy mspss_person_feelings mspss_family_decide mspss_friends_talk
## 3 2 mspss_person_help mspss_person_joy mspss_family_help mspss_family_support mspss_person_comfort mspss_friends_help mspss_friends_count mspss_family_talk mspss_friends_joy mspss_person_feelings mspss_family_decide mspss_friends_talk
## 3 3 mspss_person_help mspss_person_joy mspss_family_help mspss_family_support mspss_person_comfort mspss_friends_help mspss_friends_count mspss_family_talk mspss_friends_joy mspss_person_feelings mspss_family_decide mspss_friends_talk
## 3 4 mspss_person_help mspss_person_joy mspss_family_help mspss_family_support mspss_person_comfort mspss_friends_help mspss_friends_count mspss_family_talk mspss_friends_joy mspss_person_feelings mspss_family_decide mspss_friends_talk
## 3 5 mspss_person_help mspss_person_joy mspss_family_help mspss_family_support mspss_person_comfort mspss_friends_help mspss_friends_count mspss_family_talk mspss_friends_joy mspss_person_feelings mspss_family_decide mspss_friends_talk
## 3 6 mspss_person_help mspss_person_joy mspss_family_help mspss_family_support mspss_person_comfort mspss_friends_help mspss_friends_count mspss_family_talk mspss_friends_joy mspss_person_feelings mspss_family_decide mspss_friends_talk
## 3 7 mspss_person_help mspss_person_joy mspss_family_help mspss_family_support mspss_person_comfort mspss_friends_help mspss_friends_count mspss_family_talk mspss_friends_joy mspss_person_feelings mspss_family_decide mspss_friends_talk
## 3 8 mspss_person_help mspss_person_joy mspss_family_help mspss_family_support mspss_person_comfort mspss_friends_help mspss_friends_count mspss_family_talk mspss_friends_joy mspss_person_feelings mspss_family_decide mspss_friends_talk
## 3 9 mspss_person_help mspss_person_joy mspss_family_help mspss_family_support mspss_person_comfort mspss_friends_help mspss_friends_count mspss_family_talk mspss_friends_joy mspss_person_feelings mspss_family_decide mspss_friends_talk
## 3 10 mspss_person_help mspss_person_joy mspss_family_help mspss_family_support mspss_person_comfort mspss_friends_help mspss_friends_count mspss_family_talk mspss_friends_joy mspss_person_feelings mspss_family_decide mspss_friends_talk
## 3 11 mspss_person_help mspss_person_joy mspss_family_help mspss_family_support mspss_person_comfort mspss_friends_help mspss_friends_count mspss_family_talk mspss_friends_joy mspss_person_feelings mspss_family_decide mspss_friends_talk
## 3 12 mspss_person_help mspss_person_joy mspss_family_help mspss_family_support mspss_person_comfort mspss_friends_help mspss_friends_count mspss_family_talk mspss_friends_joy mspss_person_feelings mspss_family_decide mspss_friends_talk
## 3 13 mspss_person_help mspss_person_joy mspss_family_help mspss_family_support mspss_person_comfort mspss_friends_help mspss_friends_count mspss_family_talk mspss_friends_joy mspss_person_feelings mspss_family_decide mspss_friends_talk
## 3 14 mspss_person_help mspss_person_joy mspss_family_help mspss_family_support mspss_person_comfort mspss_friends_help mspss_friends_count mspss_family_talk mspss_friends_joy mspss_person_feelings mspss_family_decide mspss_friends_talk
## 3 15 mspss_person_help mspss_person_joy mspss_family_help mspss_family_support mspss_person_comfort mspss_friends_help mspss_friends_count mspss_family_talk mspss_friends_joy mspss_person_feelings mspss_family_decide mspss_friends_talk
## 3 16 mspss_person_help mspss_person_joy mspss_family_help mspss_family_support mspss_person_comfort mspss_friends_help mspss_friends_count mspss_family_talk mspss_friends_joy mspss_person_feelings mspss_family_decide mspss_friends_talk
## 3 17 mspss_person_help mspss_person_joy mspss_family_help mspss_family_support mspss_person_comfort mspss_friends_help mspss_friends_count mspss_family_talk mspss_friends_joy mspss_person_feelings mspss_family_decide mspss_friends_talk
## 3 18 mspss_person_help mspss_person_joy mspss_family_help mspss_family_support mspss_person_comfort mspss_friends_help mspss_friends_count mspss_family_talk mspss_friends_joy mspss_person_feelings mspss_family_decide mspss_friends_talk
## 3 19 mspss_person_help mspss_person_joy mspss_family_help mspss_family_support mspss_person_comfort mspss_friends_help mspss_friends_count mspss_family_talk mspss_friends_joy mspss_person_feelings mspss_family_decide mspss_friends_talk
## 3 20 mspss_person_help mspss_person_joy mspss_family_help mspss_family_support mspss_person_comfort mspss_friends_help mspss_friends_count mspss_family_talk mspss_friends_joy mspss_person_feelings mspss_family_decide mspss_friends_talk
## 4 1 mspss_person_help mspss_person_joy mspss_family_help mspss_family_support mspss_person_comfort mspss_friends_help mspss_friends_count mspss_family_talk mspss_friends_joy mspss_person_feelings mspss_family_decide mspss_friends_talk
## 4 2 mspss_person_help mspss_person_joy mspss_family_help mspss_family_support mspss_person_comfort mspss_friends_help mspss_friends_count mspss_family_talk mspss_friends_joy mspss_person_feelings mspss_family_decide mspss_friends_talk
## 4 3 mspss_person_help mspss_person_joy mspss_family_help mspss_family_support mspss_person_comfort mspss_friends_help mspss_friends_count mspss_family_talk mspss_friends_joy mspss_person_feelings mspss_family_decide mspss_friends_talk
## 4 4 mspss_person_help mspss_person_joy mspss_family_help mspss_family_support mspss_person_comfort mspss_friends_help mspss_friends_count mspss_family_talk mspss_friends_joy mspss_person_feelings mspss_family_decide mspss_friends_talk
## 4 5 mspss_person_help mspss_person_joy mspss_family_help mspss_family_support mspss_person_comfort mspss_friends_help mspss_friends_count mspss_family_talk mspss_friends_joy mspss_person_feelings mspss_family_decide mspss_friends_talk
## 4 6 mspss_person_help mspss_person_joy mspss_family_help mspss_family_support mspss_person_comfort mspss_friends_help mspss_friends_count mspss_family_talk mspss_friends_joy mspss_person_feelings mspss_family_decide mspss_friends_talk
## 4 7 mspss_person_help mspss_person_joy mspss_family_help mspss_family_support mspss_person_comfort mspss_friends_help mspss_friends_count mspss_family_talk mspss_friends_joy mspss_person_feelings mspss_family_decide mspss_friends_talk
## 4 8 mspss_person_help mspss_person_joy mspss_family_help mspss_family_support mspss_person_comfort mspss_friends_help mspss_friends_count mspss_family_talk mspss_friends_joy mspss_person_feelings mspss_family_decide mspss_friends_talk
## 4 9 mspss_person_help mspss_person_joy mspss_family_help mspss_family_support mspss_person_comfort mspss_friends_help mspss_friends_count mspss_family_talk mspss_friends_joy mspss_person_feelings mspss_family_decide mspss_friends_talk
## 4 10 mspss_person_help mspss_person_joy mspss_family_help mspss_family_support mspss_person_comfort mspss_friends_help mspss_friends_count mspss_family_talk mspss_friends_joy mspss_person_feelings mspss_family_decide mspss_friends_talk
## 4 11 mspss_person_help mspss_person_joy mspss_family_help mspss_family_support mspss_person_comfort mspss_friends_help mspss_friends_count mspss_family_talk mspss_friends_joy mspss_person_feelings mspss_family_decide mspss_friends_talk
## 4 12 mspss_person_help mspss_person_joy mspss_family_help mspss_family_support mspss_person_comfort mspss_friends_help mspss_friends_count mspss_family_talk mspss_friends_joy mspss_person_feelings mspss_family_decide mspss_friends_talk
## 4 13 mspss_person_help mspss_person_joy mspss_family_help mspss_family_support mspss_person_comfort mspss_friends_help mspss_friends_count mspss_family_talk mspss_friends_joy mspss_person_feelings mspss_family_decide mspss_friends_talk
## 4 14 mspss_person_help mspss_person_joy mspss_family_help mspss_family_support mspss_person_comfort mspss_friends_help mspss_friends_count mspss_family_talk mspss_friends_joy mspss_person_feelings mspss_family_decide mspss_friends_talk
## 4 15 mspss_person_help mspss_person_joy mspss_family_help mspss_family_support mspss_person_comfort mspss_friends_help mspss_friends_count mspss_family_talk mspss_friends_joy mspss_person_feelings mspss_family_decide mspss_friends_talk
## 4 16 mspss_person_help mspss_person_joy mspss_family_help mspss_family_support mspss_person_comfort mspss_friends_help mspss_friends_count mspss_family_talk mspss_friends_joy mspss_person_feelings mspss_family_decide mspss_friends_talk
## 4 17 mspss_person_help mspss_person_joy mspss_family_help mspss_family_support mspss_person_comfort mspss_friends_help mspss_friends_count mspss_family_talk mspss_friends_joy mspss_person_feelings mspss_family_decide mspss_friends_talk
## 4 18 mspss_person_help mspss_person_joy mspss_family_help mspss_family_support mspss_person_comfort mspss_friends_help mspss_friends_count mspss_family_talk mspss_friends_joy mspss_person_feelings mspss_family_decide mspss_friends_talk
## 4 19 mspss_person_help mspss_person_joy mspss_family_help mspss_family_support mspss_person_comfort mspss_friends_help mspss_friends_count mspss_family_talk mspss_friends_joy mspss_person_feelings mspss_family_decide mspss_friends_talk
## 4 20 mspss_person_help mspss_person_joy mspss_family_help mspss_family_support mspss_person_comfort mspss_friends_help mspss_friends_count mspss_family_talk mspss_friends_joy mspss_person_feelings mspss_family_decide mspss_friends_talk
## 5 1 mspss_person_help mspss_person_joy mspss_family_help mspss_family_support mspss_person_comfort mspss_friends_help mspss_friends_count mspss_family_talk mspss_friends_joy mspss_person_feelings mspss_family_decide mspss_friends_talk
## 5 2 mspss_person_help mspss_person_joy mspss_family_help mspss_family_support mspss_person_comfort mspss_friends_help mspss_friends_count mspss_family_talk mspss_friends_joy mspss_person_feelings mspss_family_decide mspss_friends_talk
## 5 3 mspss_person_help mspss_person_joy mspss_family_help mspss_family_support mspss_person_comfort mspss_friends_help mspss_friends_count mspss_family_talk mspss_friends_joy mspss_person_feelings mspss_family_decide mspss_friends_talk
## 5 4 mspss_person_help mspss_person_joy mspss_family_help mspss_family_support mspss_person_comfort mspss_friends_help mspss_friends_count mspss_family_talk mspss_friends_joy mspss_person_feelings mspss_family_decide mspss_friends_talk
## 5 5 mspss_person_help mspss_person_joy mspss_family_help mspss_family_support mspss_person_comfort mspss_friends_help mspss_friends_count mspss_family_talk mspss_friends_joy mspss_person_feelings mspss_family_decide mspss_friends_talk
## 5 6 mspss_person_help mspss_person_joy mspss_family_help mspss_family_support mspss_person_comfort mspss_friends_help mspss_friends_count mspss_family_talk mspss_friends_joy mspss_person_feelings mspss_family_decide mspss_friends_talk
## 5 7 mspss_person_help mspss_person_joy mspss_family_help mspss_family_support mspss_person_comfort mspss_friends_help mspss_friends_count mspss_family_talk mspss_friends_joy mspss_person_feelings mspss_family_decide mspss_friends_talk
## 5 8 mspss_person_help mspss_person_joy mspss_family_help mspss_family_support mspss_person_comfort mspss_friends_help mspss_friends_count mspss_family_talk mspss_friends_joy mspss_person_feelings mspss_family_decide mspss_friends_talk
## 5 9 mspss_person_help mspss_person_joy mspss_family_help mspss_family_support mspss_person_comfort mspss_friends_help mspss_friends_count mspss_family_talk mspss_friends_joy mspss_person_feelings mspss_family_decide mspss_friends_talk
## 5 10 mspss_person_help mspss_person_joy mspss_family_help mspss_family_support mspss_person_comfort mspss_friends_help mspss_friends_count mspss_family_talk mspss_friends_joy mspss_person_feelings mspss_family_decide mspss_friends_talk
## 5 11 mspss_person_help mspss_person_joy mspss_family_help mspss_family_support mspss_person_comfort mspss_friends_help mspss_friends_count mspss_family_talk mspss_friends_joy mspss_person_feelings mspss_family_decide mspss_friends_talk
## 5 12 mspss_person_help mspss_person_joy mspss_family_help mspss_family_support mspss_person_comfort mspss_friends_help mspss_friends_count mspss_family_talk mspss_friends_joy mspss_person_feelings mspss_family_decide mspss_friends_talk
## 5 13 mspss_person_help mspss_person_joy mspss_family_help mspss_family_support mspss_person_comfort mspss_friends_help mspss_friends_count mspss_family_talk mspss_friends_joy mspss_person_feelings mspss_family_decide mspss_friends_talk
## 5 14 mspss_person_help mspss_person_joy mspss_family_help mspss_family_support mspss_person_comfort mspss_friends_help mspss_friends_count mspss_family_talk mspss_friends_joy mspss_person_feelings mspss_family_decide mspss_friends_talk
## 5 15 mspss_person_help mspss_person_joy mspss_family_help mspss_family_support mspss_person_comfort mspss_friends_help mspss_friends_count mspss_family_talk mspss_friends_joy mspss_person_feelings mspss_family_decide mspss_friends_talk
## 5 16 mspss_person_help mspss_person_joy mspss_family_help mspss_family_support mspss_person_comfort mspss_friends_help mspss_friends_count mspss_family_talk mspss_friends_joy mspss_person_feelings mspss_family_decide mspss_friends_talk
## 5 17 mspss_person_help mspss_person_joy mspss_family_help mspss_family_support mspss_person_comfort mspss_friends_help mspss_friends_count mspss_family_talk mspss_friends_joy mspss_person_feelings mspss_family_decide mspss_friends_talk
## 5 18 mspss_person_help mspss_person_joy mspss_family_help mspss_family_support mspss_person_comfort mspss_friends_help mspss_friends_count mspss_family_talk mspss_friends_joy mspss_person_feelings mspss_family_decide mspss_friends_talk
## 5 19 mspss_person_help mspss_person_joy mspss_family_help mspss_family_support mspss_person_comfort mspss_friends_help mspss_friends_count mspss_family_talk mspss_friends_joy mspss_person_feelings mspss_family_decide mspss_friends_talk
## 5 20 mspss_person_help mspss_person_joy mspss_family_help mspss_family_support mspss_person_comfort mspss_friends_help mspss_friends_count mspss_family_talk mspss_friends_joy mspss_person_feelings mspss_family_decide mspss_friends_talk

##INCLUDING THE IMPUTED VARIABLES IN THE PL AND ESM DATASETS##

dat_esm = list()
PLdat_complete = list()
imp.data.Wave.I = list()
imp.data.Wave.Covid = list()

for (i in 1:20) {
 #Computing the sum score of the SSL
 imp.data.Wave.I[[i]] = complete(data.imp.Wave.I,i)
 imp.data.Wave.I[[i]]$SSL = rowSums(imp.data.Wave.I[[i]][,c("ssl_invite", "ssl_visit","ssl_affection", "ssl_comfort", "ssl_compliment", "ssl_interest",
 "ssl_offerhelp", "ssl_ease", "ssl_advice", "ssl_confidence", "ssl_askhelp",
 "ssl_positivecharacter")])

 #Computing the sum score of the VPV
 imp.data.Wave.I[[i]]$VPV = rowSums(imp.data.Wave.I[[i]][,c("vpv_meetfriends", "vpv_recogniseemotion", "vpv_class", "vpv_help", "vpv_easycontact",
 "vpv_hurt", "vpv_questions", "vpv_sensitivities", "vpv_standup", "vpv_said", "vpv_seekcontact",
 "vpv_wishes", "vpv_getalong", "vpv_interest", "vpv_talkconcerns", "vpv_adapt",
 "vpv_goodcontact", "vpv_learn")])

 #Computing the sum score of the MSPSS
 imp.data.Wave.Covid[[i]] = complete(data.imp.Wave.Covid,i)
 imp.data.Wave.Covid[[i]]$MSPSS = rowSums(imp.data.Wave.Covid[[i]][,c("mspss_person_help", "mspss_person_joy", "mspss_family_help", "mspss_family_support",
 "mspss_person_comfort", "mspss_friends_help", "mspss_friends_count",
 "mspss_family_talk", "mspss_friends_joy", "mspss_person_feelings",
 "mspss_family_decide", "mspss_friends_talk")])


 n.ID = unique(dat$record_id)

 #Adding variables to the PL dataset
 PLdat_complete[[i]] = PLdat
 PLdat_complete[[i]]$SSL = rep(NA,nrow(PLdat))
 PLdat_complete[[i]]$VPV = rep(NA,nrow(PLdat))
 PLdat_complete[[i]]$MSPSS = rep(NA,nrow(PLdat))

 for (j in n.ID) {

 #SSL and VPV
 if (length(which(PLdat$record_id==j & PLdat$wave == 'wave1'))>0){

 T.i = which(PLdat$record_id==j)
 n.ID.Wave.I = which(imp.data.Wave.I[[i]][,"record_id"]==j)

 PLdat_complete[[i]]$SSL[T.i] = imp.data.Wave.I[[i]]$SSL[n.ID.Wave.I]
 PLdat_complete[[i]]$VPV[T.i] = imp.data.Wave.I[[i]]$VPV[n.ID.Wave.I]
 }

 #MSPSS
 T.i.covid = which(PLdat$record_id==j & PLdat$wave == 'covid')
 n.ID.Wave.Covid = which(imp.data.Wave.Covid[[i]][,"record_id"]==j)

 PLdat_complete[[i]]$MSPSS[T.i.covid] = imp.data.Wave.Covid[[i]]$MSPSS[n.ID.Wave.Covid]
 }

 #Adding variables to the ESM dataset
 dat_esm[[i]] = dat
 dat_esm[[i]]$SSL = rep(NA,nrow(dat))
 dat_esm[[i]]$VPV = rep(NA,nrow(dat))
 dat_esm[[i]]$MSPSS = rep(NA,nrow(dat))

 for (j in n.ID) {

 #SSL and VPV
 if (length(which(dat$record_id==j & dat$wave == 'wave1'))>0){

 T.i = which(dat$record_id==j)
 n.ID.Wave.I = which(imp.data.Wave.I[[i]][,"record_id"]==j)

 dat_esm[[i]]$SSL[T.i] = imp.data.Wave.I[[i]][n.ID.Wave.I,"SSL"]
 dat_esm[[i]]$VPV[T.i] = imp.data.Wave.I[[i]][n.ID.Wave.I,"VPV"]
 }

 #MSPSS
 T.i.covid = which(dat$record_id==j & dat$wave == 'covid')
 n.ID.Wave.Covid = which(imp.data.Wave.Covid[[i]][,"record_id"]==j)

 dat_esm[[i]]$MSPSS[T.i.covid] = imp.data.Wave.Covid[[i]][n.ID.Wave.Covid,"MSPSS"]
 }}

######################################################################################################
##RQ2 PART 2: MODERATORS IN CHANGES FROM T0 TO T1 (IMPUTED MODERATORS)##

#Alpha per moderator = .007 (Bonferroni corrected, initial alpha of .05)

PLcovidDat = list()
ESMcovidDat = list()

for (i in 1:20){
 PLcovidDat[[i]] = PLdat_complete[[i]][PLdat_complete[[i]]$wave == 'covid',]

 ESMcovidDat[[i]] = dat_esm[[i]][dat_esm[[i]]$wave == 'covid',]
}

PLcovidDat.list <- as.mitml.list(PLcovidDat)
ESMcovidDat.list <- as.mitml.list(ESMcovidDat)
PLdat.list <- as.mitml.list(PLdat_complete)
dat.list <- as.mitml.list(dat_esm)

for (i in 1:20){
 PLdat.list[[i]]$wave.f = as.factor(PLdat.list[[i]]$wave.f)
 PLdat.list[[i]]$sex = as.factor(PLdat.list[[i]]$sex)
 dat.list[[i]]$wave.f = as.factor(dat.list[[i]]$wave.f)
 dat.list[[i]]$sex = as.factor(dat.list[[i]]$sex_ismale)
 PLcovidDat.list[[i]]$wave.f = as.factor(PLcovidDat.list[[i]]$wave.f)
 PLcovidDat.list[[i]]$sex = as.factor(PLcovidDat.list[[i]]$sex)
 ESMcovidDat.list[[i]]$wave.f = as.factor(ESMcovidDat.list[[i]]$wave.f)
 ESMcovidDat.list[[i]]$sex = as.factor(ESMcovidDat.list[[i]]$sex_ismale)
}

#SSL
H2a_ssl <- with(PLdat.list, lme(PL_aloneprop ~ wave.f + SSL + wave.f*SSL + c_age + sex,
 random = ~1| record_id, na.action = na.omit))
testEstimates(H2a_ssl)

##
## Call:
##
## testEstimates(model = H2a_ssl)
##
## Final parameter estimates and inferences obtained from 20 imputed data sets.
##
## Estimate Std.Error t.value df P(>|t|) RIV FMI
## (Intercept) 0.209 0.065 3.208 3.001e+04 0.001 0.026 0.025
## wave.f2 0.417 0.082 5.065 6.147e+04 0.000 0.018 0.018
## SSL 0.000 0.003 0.090 2.342e+04 0.928 0.029 0.029
## c_age 0.012 0.008 1.619 5.398e+06 0.106 0.002 0.002
## sex2 -0.025 0.054 -0.458 3.671e+08 0.647 0.000 0.000
## wave.f2:SSL -0.009 0.003 -2.632 3.878e+04 0.008 0.023 0.022
##
## Unadjusted hypothesis test as appropriate in larger samples.

H2b_ssl <- with(dat.list, lme(momentPA ~ wave.f + SSL + wave.f*SSL + c_age + sex,
 random = ~1| record_id, method = "REML", na.action = na.omit, control = lmeControl(opt='optim')))
testEstimates(H2b_ssl)

##
## Call:
##
## testEstimates(model = H2b_ssl)
##
## Final parameter estimates and inferences obtained from 20 imputed data sets.
##
## Estimate Std.Error t.value df P(>|t|) RIV FMI
## (Intercept) 3.594 0.341 10.543 2.221e+05 0.000 0.009 0.009
## wave.fcovid 1.057 0.241 4.383 2.182e+04 0.000 0.030 0.030
## SSL 0.058 0.014 3.992 2.334e+05 0.000 0.009 0.009
## c_age -0.157 0.042 -3.721 3.296e+06 0.000 0.002 0.002
## sex1 0.045 0.330 0.136 4.159e+08 0.892 0.000 0.000
## wave.fcovid:SSL -0.037 0.010 -3.793 1.450e+04 0.000 0.038 0.036
##
## Unadjusted hypothesis test as appropriate in larger samples.

H2c_ssl <- with(dat.list, lme(momentNA ~ wave.f + SSL + wave.f*SSL + c_age + sex,
 random = ~1| record_id, method = "REML", na.action = na.omit, control = lmeControl(opt='optim')))
testEstimates(H2c_ssl)

##
## Call:
##
## testEstimates(model = H2c_ssl)
##
## Final parameter estimates and inferences obtained from 20 imputed data sets.
##
## Estimate Std.Error t.value df P(>|t|) RIV FMI
## (Intercept) 2.911 0.349 8.339 4.483e+04 0.000 0.021 0.021
## wave.fcovid 0.362 0.205 1.763 3.890e+03 0.078 0.075 0.070
## SSL -0.040 0.015 -2.670 4.669e+04 0.008 0.021 0.020
## c_age 0.015 0.042 0.367 1.783e+06 0.714 0.003 0.003
## sex1 -0.186 0.338 -0.549 1.015e+08 0.583 0.000 0.000
## wave.fcovid:SSL -0.009 0.008 -1.162 1.966e+03 0.245 0.109 0.099
##
## Unadjusted hypothesis test as appropriate in larger samples.

H2d_ssl <- with(dat.list, lme(esm_soc_alone_pleasant ~ wave.f + SSL + wave.f*SSL + c_age + sex,
 random = ~1| record_id, method = "REML", na.action = na.omit, control = lmeControl(opt='optim')))
testEstimates(H2d_ssl)

##
## Call:
##
## testEstimates(model = H2d_ssl)
##
## Final parameter estimates and inferences obtained from 20 imputed data sets.
##
## Estimate Std.Error t.value df P(>|t|) RIV FMI
## (Intercept) 5.738 0.512 11.214 1.995e+03 0.000 0.108 0.098
## wave.fcovid -0.574 0.389 -1.476 1.289e+02 0.142 0.623 0.393
## SSL -0.016 0.022 -0.730 1.723e+03 0.465 0.117 0.106
## c_age -0.144 0.060 -2.400 2.476e+06 0.016 0.003 0.003
## sex1 -0.235 0.475 -0.494 1.687e+07 0.621 0.001 0.001
## wave.fcovid:SSL 0.019 0.016 1.152 9.865e+01 0.252 0.782 0.450
##
## Unadjusted hypothesis test as appropriate in larger samples.

H2e_ssl <- with(dat.list, lme(esm_soc_alone_want ~ wave.f + SSL + wave.f*SSL + c_age + sex,
 random = ~1| record_id, method = "REML", na.action = na.omit, control = lmeControl(opt='optim')))
testEstimates(H2e_ssl)

##
## Call:
##
## testEstimates(model = H2e_ssl)
##
## Final parameter estimates and inferences obtained from 20 imputed data sets.
##
## Estimate Std.Error t.value df P(>|t|) RIV FMI
## (Intercept) 5.912 0.555 10.645 2.715e+03 0.000 0.091 0.084
## wave.fcovid -1.375 0.427 -3.221 1.939e+02 0.001 0.456 0.320
## SSL -0.053 0.024 -2.217 2.365e+03 0.027 0.098 0.090
## c_age -0.145 0.066 -2.190 1.432e+06 0.029 0.004 0.004
## sex1 0.140 0.518 0.269 1.603e+07 0.788 0.001 0.001
## wave.fcovid:SSL 0.066 0.018 3.711 1.432e+02 0.000 0.573 0.373
##
## Unadjusted hypothesis test as appropriate in larger samples.

H2f_ssl <- with(dat.list, lme(esm_soc_alone_excluded ~ wave.f + SSL + wave.f*SSL + c_age + sex,
 random = ~1| record_id, method = "REML", na.action = na.omit, control = lmeControl(opt='optim')))
testEstimates(H2f_ssl)

##
## Call:
##
## testEstimates(model = H2f_ssl)
##
## Final parameter estimates and inferences obtained from 20 imputed data sets.
##
## Estimate Std.Error t.value df P(>|t|) RIV FMI
## (Intercept) 1.793 0.242 7.411 1.018e+05 0.000 0.014 0.014
## wave.fcovid 0.855 0.183 4.661 2.609e+03 0.000 0.093 0.086
## SSL -0.020 0.010 -1.980 9.921e+04 0.048 0.014 0.014
## c_age -0.005 0.030 -0.151 1.520e+05 0.880 0.011 0.011
## sex1 -0.022 0.233 -0.096 1.596e+08 0.924 0.000 0.000
## wave.fcovid:SSL -0.033 0.007 -4.442 2.905e+03 0.000 0.088 0.082
##
## Unadjusted hypothesis test as appropriate in larger samples.

H2g_ssl <- with(dat.list, lme(esm_mood_lonely ~ wave.f + SSL + wave.f*SSL + c_age + sex,
 random = ~1| record_id, method = "REML", na.action = na.omit, control = lmeControl(opt='optim')))
testEstimates(H2g_ssl)

##
## Call:
##
## testEstimates(model = H2g_ssl)
##
## Final parameter estimates and inferences obtained from 20 imputed data sets.
##
## Estimate Std.Error t.value df P(>|t|) RIV FMI
## (Intercept) 2.873 0.410 7.005 7.490e+04 0.000 0.016 0.016
## wave.fcovid 0.377 0.325 1.159 7.196e+02 0.247 0.194 0.165
## SSL -0.043 0.017 -2.476 7.887e+04 0.013 0.016 0.016
## c_age -0.007 0.051 -0.132 1.712e+06 0.895 0.003 0.003
## sex1 0.316 0.394 0.802 1.958e+08 0.423 0.000 0.000
## wave.fcovid:SSL -0.007 0.014 -0.500 4.927e+02 0.617 0.244 0.200
##
## Unadjusted hypothesis test as appropriate in larger samples.

#Plot significant effects
#Proportion of Time Spent Socially Withdrawing
fitH2a_ssl = lapply(1:20, function(i) lme(PL_aloneprop ~ wave.f + SSL + wave.f*SSL + c_age + sex,
 random = ~1| record_id, na.action = na.omit,PLdat.list[[i]]))
effH2a_ssl <- lapply(1:20, function(i) as.data.frame(effect(term="wave.f*SSL", xlevels=list(SSL=c(15.79, 28.63)),
 mod=fitH2a_ssl[[i]]))[,c('SSL','fit','se','lower','upper')])
effH2a_ssl <- cbind(wave.f=c('1','2','1','2'), Reduce('+',effH2a_ssl)/20)
effH2a_ssl$SSL <- as.factor(effH2a_ssl$SSL)

plot_H2a_ssl <- ggplot(effH2a_ssl, aes(x=wave.f, y=fit, color=SSL,group=SSL)) +
 geom_point() +
 geom_line(size=1.2) +
 geom_ribbon(aes(ymin=lower, ymax=upper, fill=SSL),alpha=0.3) +
 labs(title = "Time Spent Socially \n Withdrawing by T0 Social Support", x= "Timepoint", y="Prop. Time Spent \n Socially Withdrawing", color="T0 Social \n Support", fill="T0 Social \n Support")+
 theme_classic() + theme(axis.title=element_text(size=10)) + theme(plot.title=element_text(size=12)) +
 scale_x_discrete(labels=c("T0", "T1")) + theme(plot.title=element_text(face="bold")) + theme(plot.title=element_text(hjust=0.5)) + theme(legend.title=element_text(size=8)) +
 theme(legend.text=element_text(size=6)) + theme(legend.position=c(0.9,0.8)) + scale_color_discrete(labels=c("Low (-1SD)", "High (+1SD)")) + scale_fill_discrete(labels=c("Low (-1SD)", "High (+1SD)"))
plot_H2a_ssl


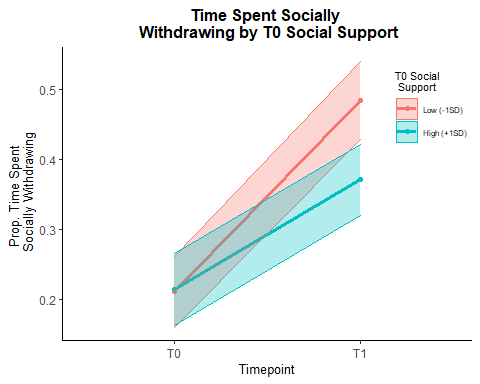


ggsave("H2a_ssl.png")

## Saving 5 x 4 in image

#Positive Affect
fitH2b_ssl = lapply(1:20, function(i) lme(momentPA ~ wave.f + SSL + wave.f*SSL + c_age + sex,
 random = ~1| record_id, method = "REML", na.action = na.omit, control = lmeControl(opt='optim'),dat.list[[i]]))
effH2b_ssl <- lapply(1:20, function(i) as.data.frame(effect(term="wave.f*SSL", xlevels=list(SSL=c(15.79, 28.63)),
 mod=fitH2b_ssl[[i]]))[,c('SSL','fit','se','lower','upper')])
effH2b_ssl <- cbind(wave.f=c('1','2','1','2'), Reduce('+',effH2b_ssl)/20)
effH2b_ssl$SSL <- as.factor(effH2b_ssl$SSL)

plot_H2b_ssl <- ggplot(effH2b_ssl, aes(x=wave.f, y=fit, color=SSL,group=SSL)) +
 geom_point() +
 geom_line(size=1.2) +
 geom_ribbon(aes(ymin=lower, ymax=upper, fill=SSL),alpha=0.3) +
 labs(title = "Positive Affect \n by T0 Social Support", x= "Timepoint", y="Positive Affect", color="T0 Social \n Support", fill="T0 Social \n Support")+
 theme_classic() + theme(axis.title=element_text(size=10)) + theme(plot.title=element_text(size=12)) +
 scale_x_discrete(labels=c("T0", "T1")) + theme(plot.title=element_text(face="bold")) + theme(plot.title=element_text(hjust=0.5)) + theme(legend.title=element_text(size=8)) +
 theme(legend.text=element_text(size=6)) + theme(legend.position=c(0.9,0.8)) + scale_color_discrete(labels=c("Low (-1SD)", "High (+1SD)")) + scale_fill_discrete(labels=c("Low (-1SD)", "High (+1SD)"))
plot_H2b_ssl


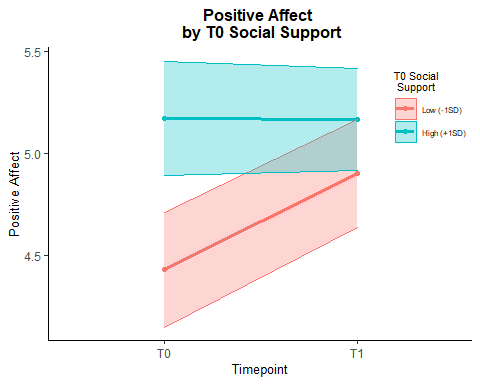


ggsave("H2b_ssl.png")

## Saving 5 x 4 in image

#Wanting to be Alone
fitH2e_ssl = lapply(1:20, function(i) lme(esm_soc_alone_want ~ wave.f + SSL + wave.f*SSL + c_age + sex,
 random = ~1| record_id, method = "REML", na.action = na.omit, control = lmeControl(opt='optim'),dat.list[[i]]))
effH2e_ssl <- lapply(1:20, function(i) as.data.frame(effect(term="wave.f*SSL", xlevels=list(SSL=c(15.79, 28.63)),
 mod=fitH2e_ssl[[i]]))[,c('SSL','fit','se','lower','upper')])
effH2e_ssl <- cbind(wave.f=c('1','2','1','2'), Reduce('+',effH2e_ssl)/20)
effH2e_ssl$SSL <- as.factor(effH2e_ssl$SSL)

plot_H2e_ssl <- ggplot(effH2e_ssl, aes(x=wave.f, y=fit, color=SSL,group=SSL)) +
 geom_point() +
 geom_line(size=1.2) +
 geom_ribbon(aes(ymin=lower, ymax=upper, fill=SSL),alpha=0.3) +
 labs(title = "Wanting to be Alone \n by T0 Social Support", x= "Timepoint", y="Wanting to be Alone", color="T0 Social \n Support", fill="T0 Social \n Support")+
 theme_classic() + theme(axis.title=element_text(size=10)) + theme(plot.title=element_text(size=12)) +
 scale_x_discrete(labels=c("T0", "T1")) + theme(plot.title=element_text(face="bold")) + theme(plot.title=element_text(hjust=0.5)) + theme(legend.title=element_text(size=8)) +
 theme(legend.text=element_text(size=6)) + theme(legend.position=c(0.9,0.8)) + scale_color_discrete(labels=c("Low (-1SD)", "High (+1SD)")) + scale_fill_discrete(labels=c("Low (-1SD)", "High (+1SD)"))
plot_H2e_ssl


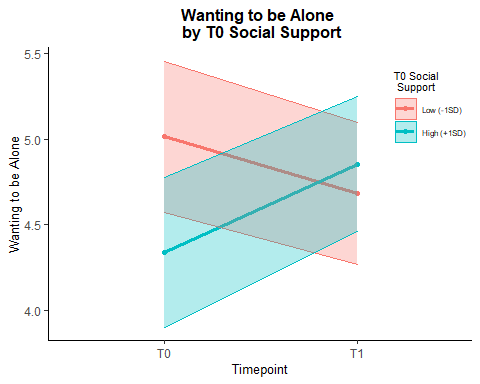


ggsave("H2e_ssl.png")

## Saving 5 x 4 in image

#Feeling Like an Outsider
fitH2f_ssl = lapply(1:20, function(i) lme(esm_soc_alone_excluded ~ wave.f + SSL + wave.f*SSL + c_age + sex,
 random = ~1| record_id, method = "REML", na.action = na.omit, control = lmeControl(opt='optim'),dat.list[[i]]))
effH2f_ssl <- lapply(1:20, function(i) as.data.frame(effect(term="wave.f*SSL", xlevels=list(SSL=c(15.79, 28.63)),
 mod=fitH2f_ssl[[i]]))[,c('SSL','fit','se','lower','upper')])
effH2f_ssl <- cbind(wave.f=c('1','2','1','2'), Reduce('+',effH2f_ssl)/20)
effH2f_ssl$SSL <- as.factor(effH2f_ssl$SSL)

plot_H2f_ssl <- ggplot(effH2f_ssl, aes(x=wave.f, y=fit, color=SSL,group=SSL)) +
 geom_point() +
 geom_line(size=1.2) +
 geom_ribbon(aes(ymin=lower, ymax=upper, fill=SSL),alpha=0.3) +
 labs(title = "Feeling Like an Outsider \n by T0 Social Support", x= "Timepoint", y="Feeling Like an Outsider", color="T0 Social \n Support", fill="T0 Social \n Support")+
 theme_classic() + theme(axis.title=element_text(size=10)) + theme(plot.title=element_text(size=12)) +
 scale_x_discrete(labels=c("T0", "T1")) + theme(plot.title=element_text(face="bold")) + theme(plot.title=element_text(hjust=0.5)) + theme(legend.title=element_text(size=8)) +
 theme(legend.text=element_text(size=6)) + theme(legend.position=c(0.9,0.8)) + scale_color_discrete(labels=c("Low (-1SD)", "High (+1SD)")) + scale_fill_discrete(labels=c("Low (-1SD)", "High (+1SD)"))
plot_H2f_ssl


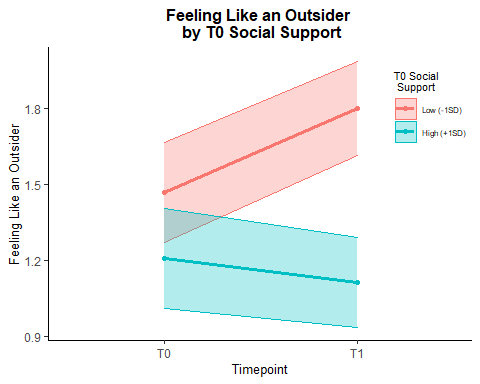


ggsave("H2f_ssl.png")

## Saving 5 x 4 in image

#VPV
H2a_vpv <- with(PLdat.list, lme(PL_aloneprop ~ wave.f + VPV + wave.f*VPV + c_age + sex,
 random = ~1| record_id, na.action = na.omit))
testEstimates(H2a_vpv)

##
## Call:
##
## testEstimates(model = H2a_vpv)
##
## Final parameter estimates and inferences obtained from 20 imputed data sets.
##
## Estimate Std.Error t.value df P(>|t|) RIV FMI
## (Intercept) 0.425 0.163 2.611 5.085e+04 0.009 0.020 0.019
## wave.f2 0.316 0.207 1.523 2.380e+04 0.128 0.029 0.028
## VPV -0.003 0.002 -1.301 5.023e+04 0.193 0.020 0.019
## c_age 0.012 0.008 1.503 6.008e+06 0.133 0.002 0.002
## sex2 -0.022 0.054 -0.400 9.817e+10 0.689 0.000 0.000
## wave.f2:VPV -0.001 0.003 -0.503 2.205e+04 0.615 0.030 0.029
##
## Unadjusted hypothesis test as appropriate in larger samples.

H2b_vpv <- with(dat.list, lme(momentPA ~ wave.f + VPV + wave.f*VPV + c_age + sex,
 random = ~1| record_id, method = "REML", na.action = na.omit, control = lmeControl(opt='optim')))
testEstimates(H2b_vpv)

##
## Call:
##
## testEstimates(model = H2b_vpv)
##
## Final parameter estimates and inferences obtained from 20 imputed data sets.
##
## Estimate Std.Error t.value df P(>|t|) RIV FMI
## (Intercept) 1.003 0.837 1.199 4.791e+04 0.231 0.020 0.020
## wave.fcovid 3.108 0.537 5.789 4.223e+04 0.000 0.022 0.021
## VPV 0.057 0.012 4.683 4.979e+04 0.000 0.020 0.020
## c_age -0.156 0.043 -3.653 2.755e+06 0.000 0.003 0.003
## sex1 -0.077 0.329 -0.234 1.068e+09 0.815 0.000 0.000
## wave.fcovid:VPV -0.042 0.008 -5.503 3.875e+04 0.000 0.023 0.022
##
## Unadjusted hypothesis test as appropriate in larger samples.

H2c_vpv <- with(dat.list, lme(momentNA ~ wave.f + VPV + wave.f*VPV + c_age + sex,
 random = ~1| record_id, method = "REML", na.action = na.omit, control = lmeControl(opt='optim')))
testEstimates(H2c_vpv)

##
## Call:
##
## testEstimates(model = H2c_vpv)
##
## Final parameter estimates and inferences obtained from 20 imputed data sets.
##
## Estimate Std.Error t.value df P(>|t|) RIV FMI
## (Intercept) 4.587 0.856 5.356 6.400e+04 0.000 0.018 0.017
## wave.fcovid -0.460 0.444 -1.036 6.303e+03 0.300 0.058 0.055
## VPV -0.037 0.012 -3.015 7.128e+04 0.003 0.017 0.016
## c_age 0.016 0.043 0.374 5.017e+06 0.709 0.002 0.002
## sex1 -0.088 0.339 -0.260 3.796e+09 0.795 0.000 0.000
## wave.fcovid:VPV 0.009 0.006 1.427 5.212e+03 0.154 0.064 0.061
##
## Unadjusted hypothesis test as appropriate in larger samples.

H2d_vpv <- with(dat.list, lme(esm_soc_alone_pleasant ~ wave.f + VPV + wave.f*VPV + c_age + sex,
 random = ~1| record_id, method = "REML", na.action = na.omit, control = lmeControl(opt='optim')))
testEstimates(H2d_vpv)

##
## Call:
##
## testEstimates(model = H2d_vpv)
##
## Final parameter estimates and inferences obtained from 20 imputed data sets.
##
## Estimate Std.Error t.value df P(>|t|) RIV FMI
## (Intercept) 5.413 1.258 4.304 1.537e+03 0.000 0.125 0.112
## wave.fcovid -0.972 0.784 -1.241 2.827e+02 0.216 0.350 0.264
## VPV -0.000 0.018 -0.026 1.487e+03 0.979 0.127 0.114
## c_age -0.139 0.060 -2.306 1.272e+06 0.021 0.004 0.004
## sex1 -0.203 0.473 -0.429 3.385e+09 0.668 0.000 0.000
## wave.fcovid:VPV 0.012 0.011 1.051 2.651e+02 0.294 0.366 0.273
##
## Unadjusted hypothesis test as appropriate in larger samples.

H2e_vpv <- with(dat.list, lme(esm_soc_alone_want ~ wave.f + VPV + wave.f*VPV + c_age + sex,
 random = ~1| record_id, method = "REML", na.action = na.omit, control = lmeControl(opt='optim')))
testEstimates(H2e_vpv)

##
## Call:
##
## testEstimates(model = H2e_vpv)
##
## Final parameter estimates and inferences obtained from 20 imputed data sets.
##
## Estimate Std.Error t.value df P(>|t|) RIV FMI
## (Intercept) 5.331 1.373 3.882 2.104e+03 0.000 0.105 0.096
## wave.fcovid -1.713 0.895 -1.915 4.134e+02 0.056 0.273 0.218
## VPV -0.009 0.020 -0.439 2.041e+03 0.661 0.107 0.097
## c_age -0.140 0.067 -2.088 2.009e+06 0.037 0.003 0.003
## sex1 0.234 0.520 0.451 3.259e+09 0.652 0.000 0.000
## wave.fcovid:VPV 0.026 0.013 2.056 3.837e+02 0.040 0.286 0.227
##
## Unadjusted hypothesis test as appropriate in larger samples.

H2f_vpv <- with(dat.list, lme(esm_soc_alone_excluded ~ wave.f + VPV + wave.f*VPV + c_age + sex,
 random = ~1| record_id, method = "REML", na.action = na.omit, control = lmeControl(opt='optim')))
testEstimates(H2f_vpv)

##
## Call:
##
## testEstimates(model = H2f_vpv)
##
## Final parameter estimates and inferences obtained from 20 imputed data sets.
##
## Estimate Std.Error t.value df P(>|t|) RIV FMI
## (Intercept) 2.129 0.602 3.535 1.516e+05 0.000 0.011 0.011
## wave.fcovid 1.544 0.405 3.813 1.698e+04 0.000 0.035 0.034
## VPV -0.012 0.009 -1.327 1.570e+05 0.184 0.011 0.011
## c_age -0.012 0.031 -0.393 3.685e+05 0.694 0.007 0.007
## sex1 0.015 0.238 0.061 1.831e+09 0.951 0.000 0.000
## wave.fcovid:VPV -0.021 0.006 -3.578 2.085e+04 0.000 0.031 0.030
##
## Unadjusted hypothesis test as appropriate in larger samples.

H2g_vpv <- with(dat.list, lme(esm_mood_lonely ~ wave.f + VPV + wave.f*VPV + c_age + sex,
 random = ~1| record_id, method = "REML", na.action = na.omit, control = lmeControl(opt='optim')))
testEstimates(H2g_vpv)

##
## Call:
##
## testEstimates(model = H2g_vpv)
##
## Final parameter estimates and inferences obtained from 20 imputed data sets.
##
## Estimate Std.Error t.value df P(>|t|) RIV FMI
## (Intercept) 3.397 1.016 3.344 4.394e+04 0.001 0.021 0.021
## wave.fcovid 0.508 0.712 0.714 1.672e+03 0.475 0.119 0.108
## VPV -0.022 0.015 -1.481 4.489e+04 0.139 0.021 0.021
## c_age -0.011 0.052 -0.207 5.719e+06 0.836 0.002 0.002
## sex1 0.396 0.399 0.993 1.666e+10 0.321 0.000 0.000
## wave.fcovid:VPV -0.004 0.010 -0.393 1.530e+03 0.694 0.125 0.113
##
## Unadjusted hypothesis test as appropriate in larger samples.

#Plot significant effects
#Positive Affect
fitH2b_vpv = lapply(1:20, function(i) lme(momentPA ~ wave.f + VPV + wave.f*VPV + c_age + sex,
 random = ~1| record_id, method = "REML", na.action = na.omit, control = lmeControl(opt='optim'),dat.list[[i]]))
effH2b_vpv <- lapply(1:20, function(i) as.data.frame(effect(term="wave.f*VPV", xlevels=list(VPV=c(61.53, 77.03)),
 mod=fitH2b_vpv[[i]]))[,c('VPV','fit','se','lower','upper')])
effH2b_vpv <- cbind(wave.f=c('1','2','1','2'), Reduce('+',effH2b_vpv)/20)
effH2b_vpv$VPV <- as.factor(effH2b_vpv$VPV)

plot_H2b_vpv <- ggplot(effH2b_vpv, aes(x=wave.f, y=fit, color=VPV,group=VPV)) +
 geom_point() +
 geom_line(size=1.2) +
 geom_ribbon(aes(ymin=lower, ymax=upper, fill=VPV),alpha=0.3) +
 labs(title = "Positive Affect by Social Skills", x= "Timepoint", y="Positive Affect", color="Social Skills", fill="Social Skills")+
 theme_classic() + theme(axis.title=element_text(size=10)) + theme(plot.title=element_text(size=12)) +
 scale_x_discrete(labels=c("T0", "T1")) + theme(plot.title=element_text(face="bold")) + theme(plot.title=element_text(hjust=0.5)) + theme(legend.title=element_text(size=8)) +
 theme(legend.text=element_text(size=6)) + theme(legend.position=c(0.9,0.8)) + scale_color_discrete(labels=c("Low (-1SD)", "High (+1SD)")) + scale_fill_discrete(labels=c("Low (-1SD)", "High (+1SD)"))
plot_H2b_vpv


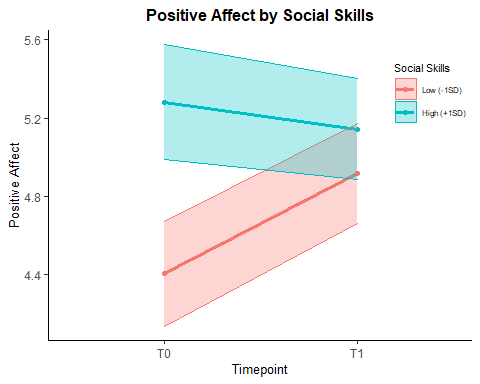


ggsave("H2b_vpv.png")

## Saving 5 x 4 in image

#Feeling Like an Outsider
fitH2f_vpv = lapply(1:20, function(i) lme(esm_soc_alone_excluded ~ wave.f + VPV + wave.f*VPV + c_age + sex,
 random = ~1| record_id, method = "REML", na.action = na.omit, control = lmeControl(opt='optim'),dat.list[[i]]))
effH2f_vpv <- lapply(1:20, function(i) as.data.frame(effect(term="wave.f*VPV", xlevels=list(VPV=c(61.53, 77.03)),
 mod=fitH2f_vpv[[i]]))[,c('VPV','fit','se','lower','upper')])
effH2f_vpv <- cbind(wave.f=c('1','2','1','2'), Reduce('+',effH2f_vpv)/20)
effH2f_vpv$VPV <- as.factor(effH2f_vpv$VPV)

plot_H2f_vpv <- ggplot(effH2f_vpv, aes(x=wave.f, y=fit, color=VPV,group=VPV)) +
 geom_point() +
 geom_line(size=1.2) +
 geom_ribbon(aes(ymin=lower, ymax=upper, fill=VPV),alpha=0.3) +
 labs(title = "Feeling Like an Outsider \n by Social Skills", x= "Timepoint", y="Feeling Like an Outsider", color="Social Skills", fill="Social Skills")+
 theme_classic() + theme(axis.title=element_text(size=10)) + theme(plot.title=element_text(size=12)) +
 scale_x_discrete(labels=c("T0", "T1")) + theme(plot.title=element_text(face="bold")) + theme(plot.title=element_text(hjust=0.5)) + theme(legend.title=element_text(size=8)) +
 theme(legend.text=element_text(size=6)) + theme(legend.position=c(0.9,0.8)) + scale_color_discrete(labels=c("Low (-1SD)", "High (+1SD)")) + scale_fill_discrete(labels=c("Low (-1SD)", "High (+1SD)"))
plot_H2f_vpv


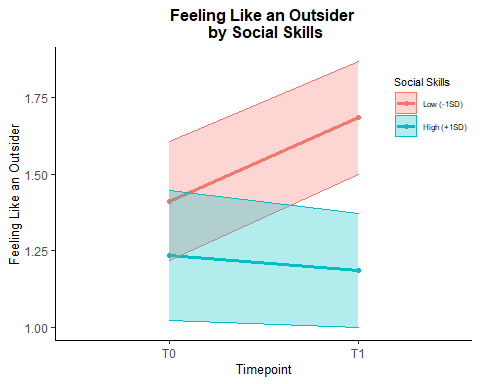


ggsave("H2f_vpv.png")

## Saving 5 x 4 in image

#MSPSS
H2a_mspss <- with(PLcovidDat.list, lm(delta_aloneprop ~ MSPSS + c_age + sex, na.action = na.omit))
testEstimates(H2a_mspss)

##
## Call:
##
## testEstimates(model = H2a_mspss)
##
## Final parameter estimates and inferences obtained from 20 imputed data sets.
##
## Estimate Std.Error t.value df P(>|t|) RIV FMI
## (Intercept) -0.663 2.779 -0.238 3.155e+04 0.812 0.025 0.025
## MSPSS 0.069 0.045 1.530 3.118e+04 0.126 0.025 0.025
## c_age -0.213 0.325 -0.655 8.134e+06 0.513 0.002 0.002
## sex2 1.351 2.479 0.545 1.746e+08 0.586 0.000 0.000
##
## Unadjusted hypothesis test as appropriate in larger samples.

H2b_mspss <- with(ESMcovidDat.list, lme(delta_PA ~ MSPSS + c_age + sex, random = ~1| record_id,
 method = "REML", na.action = na.omit, control = lmeControl(opt='optim')))
testEstimates(H2b_mspss)

##
## Call:
##
## testEstimates(model = H2b_mspss)
##
## Final parameter estimates and inferences obtained from 20 imputed data sets.
##
## Estimate Std.Error t.value df P(>|t|) RIV FMI
## (Intercept) -0.136 0.176 -0.768 1.066e+10 0.442 0.000 0.000
## MSPSS 0.002 0.003 0.763 5.371e+09 0.446 0.000 0.000
## c_age 0.007 0.020 0.340 4.156e+11 0.734 0.000 0.000
## sex1 0.477 0.162 2.938 1.627e+12 0.003 0.000 0.000
##
## Unadjusted hypothesis test as appropriate in larger samples.

H2c_mspss <- with(ESMcovidDat.list, lme(delta_NA ~ MSPSS + c_age + sex, random = ~1| record_id,
 method = "REML", na.action = na.omit, control = lmeControl(opt='optim')))
testEstimates(H2c_mspss)

##
## Call:
##
## testEstimates(model = H2c_mspss)
##
## Final parameter estimates and inferences obtained from 20 imputed data sets.
##
## Estimate Std.Error t.value df P(>|t|) RIV FMI
## (Intercept) 0.535 0.288 1.858 1.817e+08 0.063 0.000 0.000
## MSPSS -0.005 0.005 -1.120 2.627e+08 0.263 0.000 0.000
## c_age -0.050 0.033 -1.496 6.858e+09 0.135 0.000 0.000
## sex1 -0.042 0.266 -0.159 3.917e+12 0.874 0.000 0.000
##
## Unadjusted hypothesis test as appropriate in larger samples.

H2d_mspss <- with(ESMcovidDat.list, lme(delta_pleasant ~ MSPSS + c_age + sex, random = ~1| record_id,
 method = "REML", na.action = na.omit, control = lmeControl(opt='optim')))
testEstimates(H2d_mspss)

##
## Call:
##
## testEstimates(model = H2d_mspss)
##
## Final parameter estimates and inferences obtained from 20 imputed data sets.
##
## Estimate Std.Error t.value df P(>|t|) RIV FMI
## (Intercept) 0.309 0.348 0.888 9.847e+10 0.375 0.000 0.000
## MSPSS -0.002 0.006 -0.416 6.050e+10 0.678 0.000 0.000
## c_age -0.068 0.041 -1.679 1.467e+12 0.093 0.000 0.000
## sex1 0.356 0.316 1.125 1.643e+13 0.261 0.000 0.000
##
## Unadjusted hypothesis test as appropriate in larger samples.

H2e_mspss <- with(ESMcovidDat.list, lme(delta_want ~ MSPSS + c_age + sex, random = ~1| record_id,
 method = "REML", na.action = na.omit, control = lmeControl(opt='optim')))
testEstimates(H2e_mspss)

##
## Call:
##
## testEstimates(model = H2e_mspss)
##
## Final parameter estimates and inferences obtained from 20 imputed data sets.
##
## Estimate Std.Error t.value df P(>|t|) RIV FMI
## (Intercept) 0.532 0.460 1.156 3.288e+07 0.248 0.001 0.001
## MSPSS -0.002 0.007 -0.317 2.570e+07 0.751 0.001 0.001
## c_age -0.117 0.054 -2.176 7.177e+10 0.030 0.000 0.000
## sex1 0.171 0.420 0.407 4.342e+10 0.684 0.000 0.000
##
## Unadjusted hypothesis test as appropriate in larger samples.

H2f_mspss <- with(ESMcovidDat.list, lme(delta_excluded ~ MSPSS + c_age + sex, random = ~1| record_id,
 method = "REML", na.action = na.omit, control = lmeControl(opt='optim')))
testEstimates(H2f_mspss)

##
## Call:
##
## testEstimates(model = H2f_mspss)
##
## Final parameter estimates and inferences obtained from 20 imputed data sets.
##
## Estimate Std.Error t.value df P(>|t|) RIV FMI
## (Intercept) 0.645 0.264 2.447 8.494e+07 0.014 0.000 0.000
## MSPSS -0.008 0.004 -1.847 1.180e+08 0.065 0.000 0.000
## c_age -0.045 0.030 -1.498 3.973e+09 0.134 0.000 0.000
## sex1 0.265 0.250 1.059 9.175e+11 0.290 0.000 0.000
##
## Unadjusted hypothesis test as appropriate in larger samples.

H2g_mspss <- with(ESMcovidDat.list, lme(delta_loneliness ~ MSPSS + c_age + sex, random = ~1| record_id,
 method = "REML", na.action = na.omit, control = lmeControl(opt='optim')))
testEstimates(H2g_mspss)

##
## Call:
##
## testEstimates(model = H2g_mspss)
##
## Final parameter estimates and inferences obtained from 20 imputed data sets.
##
## Estimate Std.Error t.value df P(>|t|) RIV FMI
## (Intercept) 0.671 0.462 1.452 4.172e+07 0.147 0.001 0.001
## MSPSS -0.005 0.007 -0.642 4.387e+07 0.521 0.001 0.001
## c_age -0.064 0.053 -1.207 8.930e+09 0.228 0.000 0.000
## sex1 -0.330 0.432 -0.764 1.498e+11 0.445 0.000 0.000
##
## Unadjusted hypothesis test as appropriate in larger samples.

######################################################################################################
##COMPILE ALL PLOTS INTO ONE IMAGE##

t1_grid <- plot_grid(plot_H1a, plot_H2a_ssl, plot_H2b_ssl, plot_H2f_ssl, plot_H2e_ssl, plot_H2b_vpv, plot_H2f_vpv, plot_H2g_cluster,
 labels = c("A", "B", "C", "D", "E", "F", "G", "H"), align = "h", axis = "bt")
t1_grid


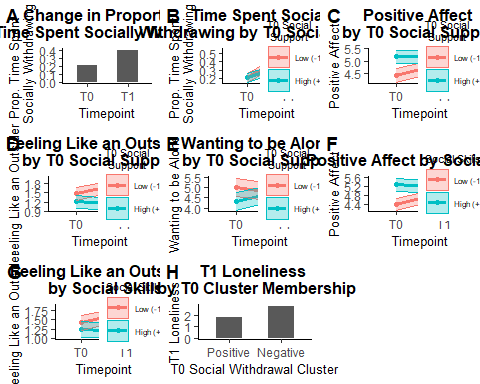


save_plot("Fig1_T1_gridplot.pdf", plot = t1_grid, ncol = 3, nrow = 3)

Pre-Pandemic (T0) to Mid-Pandemic (T2) Analyses

rm(list = ls(all.names = TRUE))

#######################################################################################################
##SETUP##

#Set seed
set.seed(1996)

#Set WD
setwd("C:/Users/u0131909/OneDrive - KU Leuven/Papers/COVID SIGMA/Analyses/Main analyses")

#Install and load packages
if (!suppressWarnings(require(remotes, quietly=TRUE)))
 install.packages("remotes")
if (!suppressWarnings(require(esmpack, quietly=TRUE)))
 remotes::install_github("wviechtb/esmpack")

pckgs <- c("esmpack", "dplyr", "tidyr", "psych", "nlme", "multilevelTools", "naniar", "apaTables", "GPArotation", "mitml", "lmerTest", "mice",
 "ggplot2", "ggsignif", "effects", "r2glmm", "cowplot")

lapply(pckgs, library, character.only = TRUE)

## [[1]]
## [1] "cowplot" "r2glmm" "effects" "carData" "ggsignif" "ggplot2"
## [7] "mice" "lmerTest" "lme4" "Matrix" "mitml" "GPArotation"
## [13] "apaTables" "naniar" "multilevelTools" "nlme" "psych" "tidyr"
## [19] "dplyr" "esmpack" "remotes" "rmarkdown" "stats" "graphics"
## [25] "grDevices" "utils" "datasets" "methods" "base"
##
## [[2]]
## [1] "cowplot" "r2glmm" "effects" "carData" "ggsignif" "ggplot2"
## [7] "mice" "lmerTest" "lme4" "Matrix" "mitml" "GPArotation"
## [13] "apaTables" "naniar" "multilevelTools" "nlme" "psych" "tidyr"
## [19] "dplyr" "esmpack" "remotes" "rmarkdown" "stats" "graphics"
## [25] "grDevices" "utils" "datasets" "methods" "base"
##
## [[3]]
## [1] "cowplot" "r2glmm" "effects" "carData" "ggsignif" "ggplot2"
## [7] "mice" "lmerTest" "lme4" "Matrix" "mitml" "GPArotation"
## [13] "apaTables" "naniar" "multilevelTools" "nlme" "psych" "tidyr"
## [19] "dplyr" "esmpack" "remotes" "rmarkdown" "stats" "graphics"
## [25] "grDevices" "utils" "datasets" "methods" "base"
##
## [[4]]
## [1] "cowplot" "r2glmm" "effects" "carData" "ggsignif" "ggplot2"
## [7] "mice" "lmerTest" "lme4" "Matrix" "mitml" "GPArotation"
## [13] "apaTables" "naniar" "multilevelTools" "nlme" "psych" "tidyr"
## [19] "dplyr" "esmpack" "remotes" "rmarkdown" "stats" "graphics"
## [25] "grDevices" "utils" "datasets" "methods" "base"
##
## [[5]]
## [1] "cowplot" "r2glmm" "effects" "carData" "ggsignif" "ggplot2"
## [7] "mice" "lmerTest" "lme4" "Matrix" "mitml" "GPArotation"
## [13] "apaTables" "naniar" "multilevelTools" "nlme" "psych" "tidyr"
## [19] "dplyr" "esmpack" "remotes" "rmarkdown" "stats" "graphics"
## [25] "grDevices" "utils" "datasets" "methods" "base"
##
## [[6]]
## [1] "cowplot" "r2glmm" "effects" "carData" "ggsignif" "ggplot2"
## [7] "mice" "lmerTest" "lme4" "Matrix" "mitml" "GPArotation"
## [13] "apaTables" "naniar" "multilevelTools" "nlme" "psych" "tidyr"
## [19] "dplyr" "esmpack" "remotes" "rmarkdown" "stats" "graphics"
## [25] "grDevices" "utils" "datasets" "methods" "base"
##
## [[7]]
## [1] "cowplot" "r2glmm" "effects" "carData" "ggsignif" "ggplot2"
## [7] "mice" "lmerTest" "lme4" "Matrix" "mitml" "GPArotation"
## [13] "apaTables" "naniar" "multilevelTools" "nlme" "psych" "tidyr"
## [19] "dplyr" "esmpack" "remotes" "rmarkdown" "stats" "graphics"
## [25] "grDevices" "utils" "datasets" "methods" "base"
##
## [[8]]
## [1] "cowplot" "r2glmm" "effects" "carData" "ggsignif" "ggplot2"
## [7] "mice" "lmerTest" "lme4" "Matrix" "mitml" "GPArotation"
## [13] "apaTables" "naniar" "multilevelTools" "nlme" "psych" "tidyr"
## [19] "dplyr" "esmpack" "remotes" "rmarkdown" "stats" "graphics"
## [25] "grDevices" "utils" "datasets" "methods" "base"
##
## [[9]]
## [1] "cowplot" "r2glmm" "effects" "carData" "ggsignif" "ggplot2"
## [7] "mice" "lmerTest" "lme4" "Matrix" "mitml" "GPArotation"
## [13] "apaTables" "naniar" "multilevelTools" "nlme" "psych" "tidyr"
## [19] "dplyr" "esmpack" "remotes" "rmarkdown" "stats" "graphics"
## [25] "grDevices" "utils" "datasets" "methods" "base"
##
## [[10]]
## [1] "cowplot" "r2glmm" "effects" "carData" "ggsignif" "ggplot2"
## [7] "mice" "lmerTest" "lme4" "Matrix" "mitml" "GPArotation"
## [13] "apaTables" "naniar" "multilevelTools" "nlme" "psych" "tidyr"
## [19] "dplyr" "esmpack" "remotes" "rmarkdown" "stats" "graphics"
## [25] "grDevices" "utils" "datasets" "methods" "base"
##
## [[11]]
## [1] "cowplot" "r2glmm" "effects" "carData" "ggsignif" "ggplot2"
## [7] "mice" "lmerTest" "lme4" "Matrix" "mitml" "GPArotation"
## [13] "apaTables" "naniar" "multilevelTools" "nlme" "psych" "tidyr"
## [19] "dplyr" "esmpack" "remotes" "rmarkdown" "stats" "graphics"
## [25] "grDevices" "utils" "datasets" "methods" "base"
##
## [[12]]
## [1] "cowplot" "r2glmm" "effects" "carData" "ggsignif" "ggplot2"
## [7] "mice" "lmerTest" "lme4" "Matrix" "mitml" "GPArotation"
## [13] "apaTables" "naniar" "multilevelTools" "nlme" "psych" "tidyr"
## [19] "dplyr" "esmpack" "remotes" "rmarkdown" "stats" "graphics"
## [25] "grDevices" "utils" "datasets" "methods" "base"
##
## [[13]]
## [1] "cowplot" "r2glmm" "effects" "carData" "ggsignif" "ggplot2"
## [7] "mice" "lmerTest" "lme4" "Matrix" "mitml" "GPArotation"
## [13] "apaTables" "naniar" "multilevelTools" "nlme" "psych" "tidyr"
## [19] "dplyr" "esmpack" "remotes" "rmarkdown" "stats" "graphics"
## [25] "grDevices" "utils" "datasets" "methods" "base"
##
## [[14]]
## [1] "cowplot" "r2glmm" "effects" "carData" "ggsignif" "ggplot2"
## [7] "mice" "lmerTest" "lme4" "Matrix" "mitml" "GPArotation"
## [13] "apaTables" "naniar" "multilevelTools" "nlme" "psych" "tidyr"
## [19] "dplyr" "esmpack" "remotes" "rmarkdown" "stats" "graphics"
## [25] "grDevices" "utils" "datasets" "methods" "base"
##
## [[15]]
## [1] "cowplot" "r2glmm" "effects" "carData" "ggsignif" "ggplot2"
## [7] "mice" "lmerTest" "lme4" "Matrix" "mitml" "GPArotation"
## [13] "apaTables" "naniar" "multilevelTools" "nlme" "psych" "tidyr"
## [19] "dplyr" "esmpack" "remotes" "rmarkdown" "stats" "graphics"
## [25] "grDevices" "utils" "datasets" "methods" "base"
##
## [[16]]
## [1] "cowplot" "r2glmm" "effects" "carData" "ggsignif" "ggplot2"
## [7] "mice" "lmerTest" "lme4" "Matrix" "mitml" "GPArotation"
## [13] "apaTables" "naniar" "multilevelTools" "nlme" "psych" "tidyr"
## [19] "dplyr" "esmpack" "remotes" "rmarkdown" "stats" "graphics"
## [25] "grDevices" "utils" "datasets" "methods" "base"
##
## [[17]]
## [1] "cowplot" "r2glmm" "effects" "carData" "ggsignif" "ggplot2"
## [7] "mice" "lmerTest" "lme4" "Matrix" "mitml" "GPArotation"
## [13] "apaTables" "naniar" "multilevelTools" "nlme" "psych" "tidyr"
## [19] "dplyr" "esmpack" "remotes" "rmarkdown" "stats" "graphics"
## [25] "grDevices" "utils" "datasets" "methods" "base"

#Read in data
dat <- read.csv("wave1wave2_sample.csv", header = TRUE, sep = ",", na.strings = "")

#######################################################################################################
##DATA CLEANING##

#Select only the participants who were tested after March 2020
wave2b <- read.csv("participants2021.csv", header = TRUE, sep = ",")
dat <- dat[which(dat$record_id %in% wave2b$record_id),]

#Remove (empty) RedCap variables + all esm_soc_who___ items except esm_soc_who___10 (= alone)
dat <- subset(dat, select = -c(redcap_event_name, redcap_repeat_instance, redcap_repeat_instrument, esm_soc_who___1, esm_soc_who___2,
 esm_soc_who___3, esm_soc_who___4, esm_soc_who___5, esm_soc_who___6, esm_soc_who___7, esm_soc_who___8, esm_soc_who___9, esm_soc_who___11))

#Add "obs" variable
dat <- dat %>%
 group_by(wave, record_id) %>%
 mutate(obs = row_number()-1) %>%
 ungroup()

#Spread person-level rows to beep level
dat <- dat %>%
 dplyr::group_by(wave, record_id) %>%
 tidyr::fill(c(agl_ado_age, agl_ado_sex, ssl_invite, ssl_visit, ssl_affection, ssl_comfort, ssl_compliment, ssl_interest, ssl_offerhelp, ssl_ease,
 ssl_advice, ssl_confidence, ssl_askhelp, ssl_positivecharacter, vpv_meetfriends, vpv_recogniseemotion, vpv_homework, vpv_disagreement,
 vpv_class, vpv_help, vpv_best, vpv_calm, vpv_easycontact, vpv_hurt, vpv_workleisure, vpv_polite, vpv_questions, vpv_sensitivities,
 vpv_attention, vpv_criticism, vpv_standup, vpv_said, vpv_money, vpv_think, vpv_seekcontact, vpv_wishes, vpv_order, vpv_lead, vpv_getalong,
 vpv_interest, vpv_dobest, vpv_calmcriticism, vpv_talkconcerns, vpv_adapt, vpv_usefulleisure, vpv_relax, vpv_goodcontact, vpv_learn,
 vpv_constant, vpv_adults,covid_symptoms_self, covid_symptoms_self_burden, covid_symptoms_family, covid_symptoms_family_burden,
 covid_risk_self, covid_risk_self_burden, covid_risk_family, covid_risk_family_burden, covid_job_self, covid_job_self_burden,
 covid_job_family, covid_job_family_burden, covid_healthcare, covid_healthcare_burden, covid_restriction, covid_restriction_burden,
 covid_lesspeople, covid_lesspeople_burden, covid_socialevents, covid_socialevents_burden, covid_hospital, covid_hospital_burden,
 covid_funeral, covid_funeral_burden, covid_hobby, covid_hobby_burden, covid_move, covid_move_burden, covid_tension_parents,
 covid_tension_parents_burden, covid_tension_self, covid_tension_self_burden, covid_siblings, covid_siblings_burden, covid_travel,
 covid_travel_burden, covid_basics, covid_basics_burden, covid_media, covid_media_burden, covid_school, covid_school_burden, covid_money,
 covid_money_burden, mspss_person_help, mspss_person_joy, mspss_family_help, mspss_family_support, mspss_person_comfort, mspss_friends_help,
 mspss_friends_count, mspss_family_talk, mspss_friends_joy, mspss_person_feelings, mspss_family_decide, mspss_friends_talk)) %>%
 ungroup()

#Remove rows with missingness (based on missingness in first ESM item)
dat <- subset(dat, is.na(esm_mood_cheerful)==0)

#Set values "88" as NA
dat <- dat %>%
 naniar::replace_with_na_all(condition = ~.x == 88)

#Calculate valid number of beeps per person - separately for T0 and T2
dat <- dat %>%
 group_by(wave, record_id) %>%
 mutate(bcount = length(obs)) %>%
 ungroup()

##VARIABLE CREATION##

#Momentary PA and NA
dat$momentPA <- combitems(c("esm_mood_cheerful", "esm_mood_relaxed", "esm_mood_satisfied", "esm_mood_generally_well"), dat)

## Computed 'mean' for these items: esm_mood_cheerful, esm_mood_relaxed, esm_mood_satisfied, esm_mood_generally_well.

dat$momentNA <- combitems(c("esm_mood_irritated", "esm_mood_anxious", "esm_mood_insecure", "esm_mood_paranoid", "esm_mood_sad", "esm_mood_stressed",
 "esm_mood_restless"), dat)

## Computed 'mean' for these items: esm_mood_irritated, esm_mood_anxious, esm_mood_insecure, esm_mood_paranoid, esm_mood_sad, esm_mood_stressed, esm_mood_restless.

# #Alternative if esmpack malfunctions
# dat$momentPA <- rowMeans(subset(dat,
# select=c("esm_mood_cheerful", "esm_mood_relaxed", "esm_mood_satisfied", "esm_mood_generally_well")), na.rm=T)
#
# dat$momentNA <- rowMeans(subset(dat,
# select=c("esm_mood_irritated", "esm_mood_anxious", "esm_mood_insecure", "esm_mood_paranoid", "esm_mood_sad", "esm_mood_stressed","esm_mood_restless")), na.rm=T)

#Within- and between-person reliability for PA and NA - separate for T0 and T1
omegaSEM(items = c("esm_mood_cheerful", "esm_mood_relaxed", "esm_mood_satisfied", "esm_mood_generally_well"), id = "record_id",
 data = dat[which(dat$wave == 'wave1'),])

## Warning in lav_data_full(data = data, group = group, cluster = cluster, : lavaan WARNING:
## Level-1 variable "esm_mood_cheerful" has no variance within some
## clusters. The cluster ids with zero within variance are: 2457

## Warning in lav_data_full(data = data, group = group, cluster = cluster, : lavaan WARNING:
## Level-1 variable "esm_mood_relaxed" has no variance within some
## clusters. The cluster ids with zero within variance are: 2230 2546

## Warning in lav_data_full(data = data, group = group, cluster = cluster, : lavaan WARNING:
## Level-1 variable "esm_mood_satisfied" has no variance within some
## clusters. The cluster ids with zero within variance are: 2274

## Warning in lav_data_full(data = data, group = group, cluster = cluster, : lavaan WARNING:
## Level-1 variable "esm_mood_generally_well" has no variance within
## some clusters. The cluster ids with zero within variance are: 1052
## 2230 2274

## $Results
## label est ci.lower ci.upper
## 35 omega_within 0.743 0.733 0.753
## 42 omega_between 0.956 0.945 0.966

omegaSEM(items = c("esm_mood_irritated", "esm_mood_anxious", "esm_mood_insecure", "esm_mood_paranoid", "esm_mood_sad", "esm_mood_stressed",
 "esm_mood_restless"), id = "record_id", data = dat[which(dat$wave == "wave1"),])

## Warning in lav_data_full(data = data, group = group, cluster = cluster, : lavaan WARNING:
## Level-1 variable "esm_mood_irritated" has no variance within some
## clusters. The cluster ids with zero within variance are: 2230 2274
## 2294 2383 2416 2496 2591 2751

## Warning in lav_data_full(data = data, group = group, cluster = cluster, : lavaan WARNING:
## Level-1 variable "esm_mood_anxious" has no variance within some
## clusters. The cluster ids with zero within variance are: 1011 1053
## 1102 1103 1111 1122 1126 1131 1217 1272 1273 1289 1757 1765 1812
## 1815 1846 1872 1982 1998 2010 2011 2140 2178 2186 2202 2203 2228
## 2232 2234 2274 2294 2383 2393 2416 2428 2457 2462 2511 2523 2524
## 2591 2751 2873 2887 2951 2997 3093 3187

## Warning in lav_data_full(data = data, group = group, cluster = cluster, : lavaan WARNING:
## Level-1 variable "esm_mood_insecure" has no variance within some
## clusters. The cluster ids with zero within variance are: 1215 1289
## 1770 1800 1834 1998 2230 2274 2292 2294 2383 2393 2457 2462 2523
## 2591 2751 2873 2951 2955

## Warning in lav_data_full(data = data, group = group, cluster = cluster, : lavaan WARNING:
## Level-1 variable "esm_mood_paranoid" has no variance within some
## clusters. The cluster ids with zero within variance are: 1053 1056
## 1104 1110 1111 1122 1131 1215 1289 1812 1815 1982 1998 2023 2141
## 2151 2186 2187 2202 2203 2230 2269 2274 2292 2294 2334 2383 2393
## 2411 2447 2457 2523 2546 2873 2887 2951 3064 3187 3245

## Warning in lav_data_full(data = data, group = group, cluster = cluster, : lavaan WARNING:
## Level-1 variable "esm_mood_sad" has no variance within some
## clusters. The cluster ids with zero within variance are: 1008 1053
## 1126 1289 1693 1765 1822 1982 2140 2151 2186 2187 2202 2230 2239
## 2274 2294 2383 2410 2428 2457 2496 2511 2546 2591 2887 2951 2963
## 2997 3064 3184 3187 3255

## Warning in lav_data_full(data = data, group = group, cluster = cluster, : lavaan WARNING:
## Level-1 variable "esm_mood_stressed" has no variance within some
## clusters. The cluster ids with zero within variance are: 1126 1693
## 1770 1800 2097 2457 2591 2751 2951

## Warning in lav_data_full(data = data, group = group, cluster = cluster, : lavaan WARNING:
## Level-1 variable "esm_mood_restless" has no variance within some
## clusters. The cluster ids with zero within variance are: 1053 1110
## 1126 1131 1215 1770 1785 1998 2151 2178 2186 2187 2202 2230 2232
## 2274 2383 2428 2523 2524 2951 3187

## $Results
## label est ci.lower ci.upper
## 56 omega_within 0.728 0.718 0.738
## 66 omega_between 0.894 0.872 0.917

omegaSEM(items = c("esm_mood_cheerful", "esm_mood_relaxed", "esm_mood_satisfied", "esm_mood_generally_well"), id = "record_id",
 data = dat[which(dat$wave == "wave2"),])

## Warning in lav_data_full(data = data, group = group, cluster = cluster, : lavaan WARNING:
## Level-1 variable "esm_mood_cheerful" has no variance within some
## clusters. The cluster ids with zero within variance are: 2279 2397
## 2416 2457 2564 2591 2958

## Warning in lav_data_full(data = data, group = group, cluster = cluster, : lavaan WARNING:
## Level-1 variable "esm_mood_relaxed" has no variance within some
## clusters. The cluster ids with zero within variance are: 2397 2457
## 2566 2591 2958

## Warning in lav_data_full(data = data, group = group, cluster = cluster, : lavaan WARNING:
## Level-1 variable "esm_mood_satisfied" has no variance within some
## clusters. The cluster ids with zero within variance are: 1052 2182
## 2279 2397 2416 2457 2566 2591

## Warning in lav_data_full(data = data, group = group, cluster = cluster, : lavaan WARNING:
## Level-1 variable "esm_mood_generally_well" has no variance within
## some clusters. The cluster ids with zero within variance are: 2279
## 2397 2457 2591 2958

## $Results
## label est ci.lower ci.upper
## 35 omega_within 0.801 0.792 0.810
## 42 omega_between 0.950 0.937 0.963

omegaSEM(items = c("esm_mood_irritated", "esm_mood_anxious", "esm_mood_insecure", "esm_mood_paranoid", "esm_mood_sad", "esm_mood_stressed",
 "esm_mood_restless"), id = "record_id", data = dat[which(dat$wave == "wave2"),])

## Warning in lav_data_full(data = data, group = group, cluster = cluster, : lavaan WARNING:
## Level-1 variable "esm_mood_irritated" has no variance within some
## clusters. The cluster ids with zero within variance are: 1215 2182
## 2202 2279 2383 2457 2496 2579 2591

## Warning in lav_data_full(data = data, group = group, cluster = cluster, : lavaan WARNING:
## Level-1 variable "esm_mood_anxious" has no variance within some
## clusters. The cluster ids with zero within variance are: 1102 1105
## 1126 1131 1215 1313 1693 1770 1872 1998 2012 2102 2121 2128 2178
## 2181 2182 2203 2232 2264 2294 2296 2383 2393 2397 2416 2428 2457
## 2462 2496 2564 2566 2571 2579 2751 2865 2873 2878 2887 2936 2955
## 2958 2960 2962 3093 3170 3184 3215

## Warning in lav_data_full(data = data, group = group, cluster = cluster, : lavaan WARNING:
## Level-1 variable "esm_mood_insecure" has no variance within some
## clusters. The cluster ids with zero within variance are: 1215 1693
## 1770 2232 2383 2393 2397 2446 2457 2462 2496 2564 2566 2591 2857
## 2873 3187

## Warning in lav_data_full(data = data, group = group, cluster = cluster, : lavaan WARNING:
## Level-1 variable "esm_mood_paranoid" has no variance within some
## clusters. The cluster ids with zero within variance are: 1014 1053
## 1056 1102 1105 1111 1126 1131 1174 1770 1811 1812 1998 2121 2181
## 2189 2222 2232 2238 2264 2269 2296 2383 2393 2397 2411 2416 2428
## 2446 2447 2457 2462 2496 2538 2546 2564 2566 2571 2591 2793 2857
## 2873 2878 2887 2936 2958 2962 3170 3184 3187 3215 3255

## Warning in lav_data_full(data = data, group = group, cluster = cluster, : lavaan WARNING:
## Level-1 variable "esm_mood_sad" has no variance within some
## clusters. The cluster ids with zero within variance are: 1102 1131
## 1174 1215 1313 1423 1693 1812 2128 2181 2202 2230 2232 2262 2264
## 2292 2296 2383 2393 2397 2411 2416 2428 2447 2457 2462 2496 2564
## 2566 2571 2591 2873 2887 3170 3187

## Warning in lav_data_full(data = data, group = group, cluster = cluster, : lavaan WARNING:
## Level-1 variable "esm_mood_stressed" has no variance within some
## clusters. The cluster ids with zero within variance are: 1423 1693
## 1834 1998 2128 2178 2232 2262 2393 2397 2457 2496 2564 2566 2887
## 2958 3262

## Warning in lav_data_full(data = data, group = group, cluster = cluster, : lavaan WARNING:
## Level-1 variable "esm_mood_restless" has no variance within some
## clusters. The cluster ids with zero within variance are: 1102 1105
## 1215 1423 1811 1998 2128 2181 2230 2232 2397 2446 2457 2462 2496
## 2525 2546 2564 2566 2571 2873 2887 2936 2958 3150 3170 3184 3255

## $Results
## label est ci.lower ci.upper
## 56 omega_within 0.774 0.765 0.783
## 66 omega_between 0.932 0.918 0.947

#Proportion of time spent alone
#Make alone count variable
dat <- dat %>%
 group_by(wave, record_id) %>%
 mutate(alone_count = sum(esm_soc_who___10))%>%
 ungroup()

#Make proportion alone variable
dat$alone_prop <- dat$alone_count/dat$bcount

#Turn sex into a binary variable (by omitting participants who indicated gender = other)
dat <- subset(dat, agl_ado_sex != 2)
dat$sex_ismale <- dummy.code(dat$agl_ado_sex, group = 0)

#Grand-mean center age - separate for each wave
agedf <- dat %>%
 group_by(wave, record_id) %>%
 summarise(age=mean(dat$agl_ado_age, na.rm = TRUE))

## `summarise()` has grouped output by 'wave'. You can override using the `.groups` argument.

agemean <- mean(agedf$age)

dat$c_age <- dat$agl_ado_age - agemean

#Select beeps when participants are alone - only these beeps will be taken into account for the analyses
dat <- subset(dat, esm_soc_who___10==1)

#Create new obs variable - original obs variable has gaps because we selected only the rows for which esm_soc_who___10==1
n.i = unique(dat$record_id)
dat$obs = rep(0,nrow(dat))
for (i in n.i){
 dat$obs[which(dat$record_id==i)] = seq(1:length(which(dat$record_id==i)))
}

#Create bcount_alone variable
dat <- dat %>%
 group_by(wave, record_id) %>%
 mutate(bcount_alone = length(obs)) %>%
 ungroup()

######################################################################################################
##PERSON-LEVEL DATASET##

#Make a person-level dataset
PLdat <- dat %>%
 group_by(record_id, wave) %>%
 summarize(sex = first(as.factor(sex_ismale)),
 age = mean(agl_ado_age, na.rm=T),
 bcount = mean(bcount, na.rm=T),
 bcount_alone = mean(bcount_alone, na.rm=T),
 ssl_invite = mean(ssl_invite, na.rm=T), # SSL items
 ssl_visit = mean(ssl_visit, na.rm=T),
 ssl_affection = mean(ssl_affection, na.rm=T),
 ssl_comfort = mean(ssl_comfort, na.rm=T),
 ssl_compliment = mean(ssl_compliment, na.rm=T),
 ssl_interest = mean(ssl_interest, na.rm=T),
 ssl_offerhelp = mean(ssl_offerhelp, na.rm=T),
 ssl_ease = mean(ssl_ease, na.rm=T),
 ssl_advice = mean(ssl_advice, na.rm=T),
 ssl_confidence = mean(ssl_confidence, na.rm=T),
 ssl_askhelp = mean(ssl_askhelp, na.rm=T),
 ssl_positivecharacter = mean(ssl_positivecharacter, na.rm=T),
 vpv_meetfriends = mean(vpv_meetfriends, na.rm=T), # For the VPV, we only select items here from the interpersonal subscale
 vpv_recogniseemotion = mean(vpv_recogniseemotion, na.rm=T),
 vpv_class = mean(vpv_class, na.rm=T),
 vpv_help = mean(vpv_help, na.rm=T),
 vpv_easycontact = mean(vpv_easycontact, na.rm=T),
 vpv_hurt = mean(vpv_hurt, na.rm=T),
 vpv_questions = mean(vpv_questions, na.rm=T),
 vpv_sensitivities = mean(vpv_sensitivities, na.rm=T),
 vpv_standup = mean(vpv_standup, na.rm=T),
 vpv_said = mean(vpv_said, na.rm=T),
 vpv_seekcontact = mean(vpv_seekcontact, na.rm=T),
 vpv_wishes = mean(vpv_wishes, na.rm=T),
 vpv_getalong = mean(vpv_getalong, na.rm=T),
 vpv_interest = mean(vpv_interest, na.rm=T),
 vpv_talkconcerns = mean(vpv_talkconcerns, na.rm=T),
 vpv_adapt = mean(vpv_adapt, na.rm=T),
 vpv_goodcontact = mean(vpv_goodcontact, na.rm=T),
 vpv_learn = mean(vpv_learn, na.rm=T),
 covid_symptoms_self = mean(covid_symptoms_self, na.rm=T), #COVID stressors
 covid_symptoms_self_burden = mean(covid_symptoms_self_burden, na.rm=T),
 covid_symptoms_family = mean(covid_symptoms_family, na.rm=T),
 covid_symptoms_family_burden = mean(covid_symptoms_family_burden, na.rm=T),
 covid_risk_self = mean(covid_risk_self, na.rm=T),
 covid_risk_self_burden = mean(covid_risk_self_burden, na.rm=T),
 covid_risk_family = mean(covid_risk_family, na.rm=T),
 covid_risk_family_burden = mean(covid_risk_family_burden, na.rm=T),
 covid_job_family = mean(covid_job_family, na.rm=T),
 covid_job_family_burden = mean(covid_job_family_burden, na.rm=T),
 covid_job_self = mean(covid_job_self, na.rm=T),
 covid_job_self_burden = mean(covid_job_self_burden, na.rm=T),
 covid_healthcare = mean(covid_healthcare, na.rm=T),
 covid_healthcare_burden = mean(covid_healthcare_burden, na.rm=T),
 covid_restriction = mean(covid_restriction, na.rm=T),
 covid_restriction_burden = mean(covid_restriction_burden, na.rm=T),
 covid_lesspeople = mean(covid_lesspeople, na.rm=T),
 covid_lesspeople_burden = mean(covid_lesspeople_burden, na.rm=T),
 covid_socialevents = mean(covid_socialevents, na.rm=T),
 covid_socialevents_burden = mean(covid_socialevents_burden, na.rm=T),
 covid_hospital = mean(covid_hospital, na.rm=T),
 covid_hospital_burden = mean(covid_hospital_burden, na.rm=T),
 covid_funeral = mean(covid_funeral, na.rm=T),
 covid_funeral_burden = mean(covid_funeral_burden, na.rm=T),
 covid_hobby = mean(covid_hobby, na.rm=T),
 covid_hobby_burden = mean(covid_hobby_burden, na.rm=T),
 covid_move = mean(covid_move, na.rm=T),
 covid_move_burden = mean(covid_move_burden, na.rm=T),
 covid_tension_parents = mean(covid_tension_parents, na.rm=T),
 covid_tension_parents_burden = mean(covid_tension_parents_burden, na.rm=T),
 covid_tension_self = mean(covid_tension_self, na.rm=T),
 covid_tension_self_burden = mean(covid_tension_self_burden, na.rm=T),
 covid_siblings = mean(covid_siblings, na.rm=T),
 covid_siblings_burden = mean(covid_siblings_burden, na.rm=T),
 covid_travel = mean(covid_travel, na.rm=T),
 covid_travel_burden = mean(covid_travel_burden, na.rm=T),
 covid_basics = mean(covid_basics, na.rm=T),
 covid_basics_burden = mean(covid_basics_burden, na.rm=T),
 covid_media = mean(covid_media, na.rm=T),
 covid_media_burden = mean(covid_media_burden, na.rm=T),
 covid_school = mean(covid_school, na.rm=T),
 covid_school_burden = mean(covid_school_burden, na.rm=T),
 covid_money = mean(covid_money, na.rm=T),
 covid_money_burden = mean(covid_money_burden, na.rm=T),
 mspss_person_help = mean(mspss_person_help, na.rm=T), #T1 social support
 mspss_person_joy = mean(mspss_person_joy, na.rm=T),
 mspss_family_help = mean(mspss_family_help, na.rm=T),
 mspss_family_support = mean(mspss_family_support, na.rm=T),
 mspss_person_comfort = mean(mspss_person_comfort, na.rm=T),
 mspss_friends_help = mean(mspss_friends_help, na.rm=T),
 mspss_friends_count = mean(mspss_friends_count, na.rm=T),
 mspss_family_talk = mean(mspss_family_talk, na.rm=T),
 mspss_friends_joy = mean(mspss_friends_joy, na.rm=T),
 mspss_person_feelings = mean(mspss_person_feelings, na.rm=T),
 mspss_family_decide = mean(mspss_family_decide, na.rm=T),
 mspss_friends_talk = mean(mspss_friends_talk, na.rm=T),
 PL_momentPA = mean(momentPA, na.rm=T),
 PL_momentNA = mean(momentNA, na.rm=T),
 PL_loneliness = mean(esm_mood_lonely, na.rm=T),
 PL_pleasant = mean(esm_soc_alone_pleasant, na.rm=T),
 PL_excluded = mean(esm_soc_alone_excluded, na.rm=T),
 PL_want = mean(esm_soc_alone_want, na.rm=T),
 PL_aloneprop = mean(alone_prop, na.rm=T)) %>%
 ungroup()

## `summarise()` has grouped output by 'record_id'. You can override using the `.groups` argument.

#Set values "NaN" as NA
PLdat <- PLdat %>%
 mutate_all(~ifelse(is.nan(.), NA, .))

#Resetting the reference for study
dat$wave.f <- as.factor(dat$wave)
dat$wave.f <- relevel(dat$wave.f, ref="wave1")
PLdat$wave.f <- as.factor(PLdat$wave)
PLdat$wave.f <- relevel(PLdat$wave.f, ref="wave1")

T0 = which(PLdat$wave == "wave1")
T2 = which(PLdat$wave == "wave2")

#Create total scores for questionnaires
#SSL - social support at T0
ssldata <- select(PLdat, record_id, starts_with("ssl"))
colnames(ssldata)

## [1] "record_id" "ssl_invite" "ssl_visit" "ssl_affection"
## [5] "ssl_comfort" "ssl_compliment" "ssl_interest" "ssl_offerhelp"
## [9] "ssl_ease" "ssl_advice" "ssl_confidence" "ssl_askhelp"
## [13] "ssl_positivecharacter"

total <- c("ssl_compliment", "ssl_confidence", "ssl_askhelp", "ssl_positivecharacter", "ssl_comfort", "ssl_offerhelp", "ssl_ease", "ssl_advice", "ssl_invite", "ssl_visit", "ssl_affection", "ssl_interest")

ssldata$ssl_total_miss <- apply(ssldata[,total], 1, function(x) sum(is.na(x)))
ssldata$ssl_total_mean_temp <- ifelse(ssldata$ssl_total_miss <= 4, rowMeans(ssldata[,total], na.rm = TRUE), NA)

ssldata$ssl_total_sum <- ifelse(ssldata$ssl_total_miss<=4, rowSums(ssldata[, total], na.rm = TRUE), NA)
ssldata$ssl_total_total <- ifelse(ssldata$ssl_total_miss<=4, ssldata$ssl_total_sum+(ssldata$ssl_total_miss*ssldata$ssl_total_mean_temp), ssldata$ssl_total_sum)
PLdat$ssl_total <- ssldata$ssl_total_total

#VPV - social skills at T0 and T2
vpvdata <- select(PLdat, record_id, wave, starts_with("vpv"))
colnames(vpvdata)

## [1] "record_id" "wave" "vpv_meetfriends" "vpv_recogniseemotion" "vpv_class"
## [6] "vpv_help" "vpv_easycontact" "vpv_hurt" "vpv_questions" "vpv_sensitivities"
## [11] "vpv_standup" "vpv_said" "vpv_seekcontact" "vpv_wishes" "vpv_getalong"
## [16] "vpv_interest" "vpv_talkconcerns" "vpv_adapt" "vpv_goodcontact" "vpv_learn"

vpvdata$vpv_miss <- apply(vpvdata[,2:19], 1, function(x) sum(is.na(x)))
table(vpvdata$vpv_miss)

##
## 0 1 2 3 4 5 7 17
## 360 6 2 1 1 1 2 15

vpv_r <- c("vpv_meetfriends", "vpv_class", "vpv_easycontact", "vpv_questions", "vpv_standup", "vpv_seekcontact", "vpv_getalong", "vpv_talkconcerns", "vpv_goodcontact")
PLdat$vpv_r <- ifelse(vpvdata$vpv_miss==0, rowSums(vpvdata[, vpv_r]), NA)
vpv_a <- c("vpv_recogniseemotion", "vpv_help", "vpv_hurt", "vpv_sensitivities", "vpv_said", "vpv_wishes", "vpv_learn", "vpv_interest", "vpv_adapt")
PLdat$vpv_a <- ifelse(vpvdata$vpv_miss==0, rowSums(vpvdata[, vpv_a]), NA)
PLdat$vpv_interpers <- PLdat$vpv_a+PLdat$vpv_r

#Amount of COVID-related events
PLdat$stressors_amount <- PLdat$covid_symptoms_self + PLdat$covid_symptoms_family + PLdat$covid_risk_self + PLdat$covid_risk_family +
 PLdat$covid_healthcare + PLdat$covid_job_family + PLdat$covid_job_self + PLdat$covid_restriction + PLdat$covid_lesspeople +
 PLdat$covid_socialevents + PLdat$covid_hospital + PLdat$covid_funeral + PLdat$covid_hobby + PLdat$covid_move + PLdat$covid_tension_parents +
 PLdat$covid_tension_self + PLdat$covid_siblings + PLdat$covid_travel + PLdat$covid_basics + PLdat$covid_media + PLdat$covid_school + PLdat$covid_money

#Mean burdensomeness of COVID-related events
PLdat <- PLdat %>%
 group_by(wave, record_id) %>%
 mutate(stressors_burden = mean(c(covid_symptoms_self_burden, covid_symptoms_family_burden, covid_risk_self_burden,
 covid_risk_family_burden, covid_healthcare_burden, covid_job_family_burden, covid_job_self_burden,
 covid_restriction_burden, covid_lesspeople_burden, covid_socialevents_burden, covid_hospital_burden,
 covid_funeral_burden, covid_hobby_burden, covid_move_burden, covid_tension_parents_burden,
 covid_tension_self_burden, covid_siblings_burden, covid_travel_burden, covid_basics_burden,
 covid_media_burden, covid_school_burden, covid_money_burden), na.rm = TRUE)) %>%
 mutate_all(~ifelse(is.nan(.), NA, .)) %>%
 ungroup()

## `mutate_all()` ignored the following grouping variables:
## • Columns `wave`, `record_id`
## ℹ Use `mutate_at(df, vars(-group_cols()), myoperation)` to silence the message.

#MSPSS - social support at T1
PLdat$mspss_total <- PLdat$mspss_person_help + PLdat$mspss_person_joy + PLdat$mspss_family_help + PLdat$mspss_family_support +
 PLdat$mspss_person_comfort + PLdat$mspss_friends_help + PLdat$mspss_friends_count + PLdat$mspss_family_talk +
 PLdat$mspss_friends_joy + PLdat$mspss_person_feelings + PLdat$mspss_family_decide + PLdat$mspss_friends_talk

#Add T0 cluster membership to this dataset
#The cluster membership is calculated with code from the file "analyses_code_DLsocialwithdrawal" available on the OSF project page of the
#study "Identifying clusters of adolescents based on their daily-life social withdrawal experience" (https://osf.io/7htwv/?view_only=7fa5907478364f369766f51aa19041c4)
wave1clst <- read.csv("Wave1_Clustermeans.csv", header = TRUE, sep = ",")
rcrdid <- unique(PLdat$record_id)
clstdat <- wave1clst[which(wave1clst$record_id %in% rcrdid == TRUE),]
PLdat <- left_join(x = PLdat, y = wave1clst)

## Joining, by = "record_id"

#Grand-mean center age
PLdat$c_age <- PLdat$age - mean(PLdat$age, na.rm=T)

##DESCRIPTIVES##

#Calculate ESM compliance - not separate for T0 and T1, as both had a maximum of 60 beeps
PLdat$compliance_prop <- PLdat$bcount / 60

#Dependent variable descriptives
psych::describe(PLdat[T0, c("PL_momentPA", "PL_momentNA", "PL_pleasant", "PL_excluded", "PL_want", "PL_aloneprop", "PL_loneliness")])

## vars n mean sd median trimmed mad min max range skew kurtosis se
## PL_momentPA 1 184 5.25 1.12 5.40 5.31 1.04 1.50 7.00 5.50 -0.59 0.37 0.08
## PL_momentNA 2 184 1.93 0.98 1.60 1.79 0.74 1.00 7.00 6.00 1.64 4.16 0.07
## PL_pleasant 3 184 5.19 1.60 5.50 5.38 1.73 1.00 7.00 6.00 -0.82 -0.09 0.12
## PL_excluded 4 184 1.36 0.82 1.00 1.16 0.00 1.00 7.00 6.00 3.78 17.97 0.06
## PL_want 5 184 4.58 1.68 4.71 4.69 1.72 1.00 7.00 6.00 -0.46 -0.55 0.12
## PL_aloneprop 6 184 0.18 0.12 0.14 0.16 0.13 0.02 0.71 0.69 1.01 1.21 0.01
## PL_loneliness 7 184 2.00 1.35 1.23 1.75 0.34 1.00 7.00 6.00 1.54 2.13 0.10

psych::describe(PLdat[T2, c("PL_momentPA", "PL_momentNA", "PL_pleasant", "PL_excluded", "PL_want", "PL_aloneprop", "PL_loneliness")])

## vars n mean sd median trimmed mad min max range skew kurtosis se
## PL_momentPA 1 204 4.63 1.09 4.63 4.65 1.00 1.25 7.00 5.75 -0.28 0.13 0.08
## PL_momentNA 2 204 2.30 1.02 2.03 2.19 0.97 1.00 5.86 4.86 0.96 0.48 0.07
## PL_pleasant 3 204 4.89 1.46 5.00 4.98 1.48 1.00 7.00 6.00 -0.46 -0.45 0.10
## PL_excluded 4 204 1.47 0.91 1.07 1.26 0.11 1.00 7.00 6.00 3.62 16.11 0.06
## PL_want 5 204 4.44 1.54 4.48 4.49 1.56 1.00 7.00 6.00 -0.24 -0.63 0.11
## PL_aloneprop 6 204 0.44 0.22 0.44 0.44 0.24 0.03 1.00 0.97 0.32 -0.53 0.02
## PL_loneliness 7 204 2.29 1.43 1.85 2.07 1.26 1.00 7.00 6.00 1.20 0.78 0.10

boxplot(PL_momentPA ~ wave, dat=PLdat)


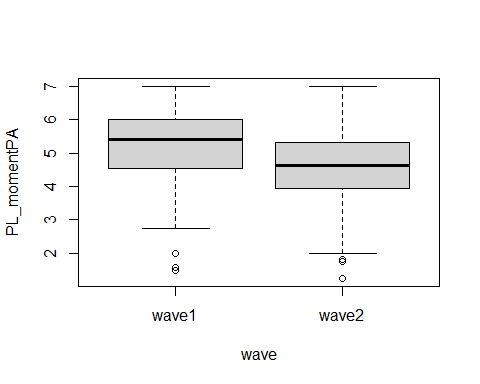


boxplot(PL_momentNA ~ wave, dat=PLdat)


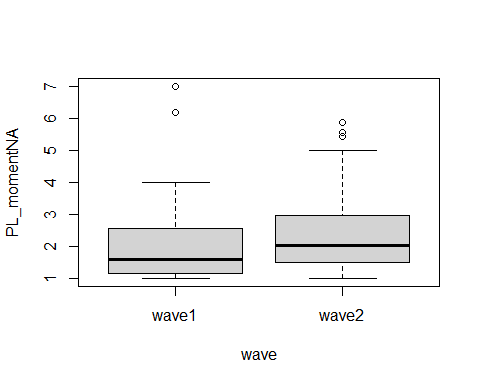


boxplot(PL_pleasant ~ wave, dat=PLdat)


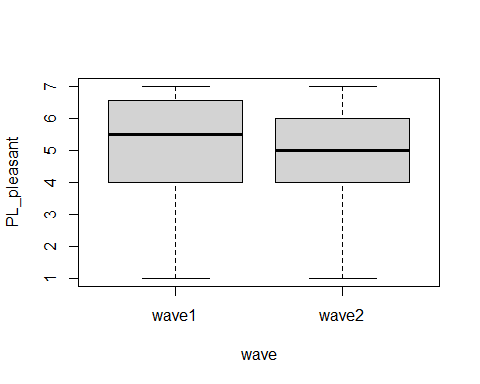


boxplot(PL_excluded ~ wave, dat=PLdat)


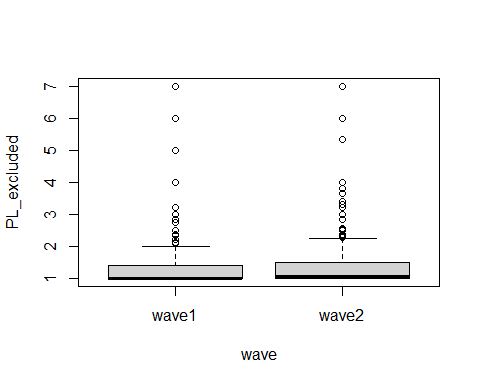


boxplot(PL_want ~ wave, dat=PLdat)


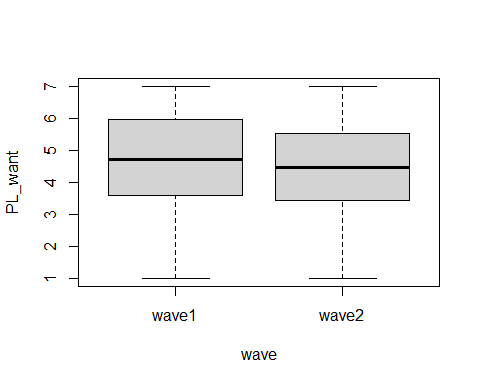


boxplot(PL_aloneprop ~ wave, dat=PLdat)


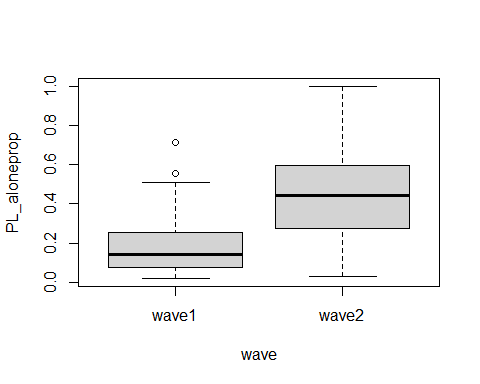


boxplot(PL_loneliness ~ wave, dat=PLdat)


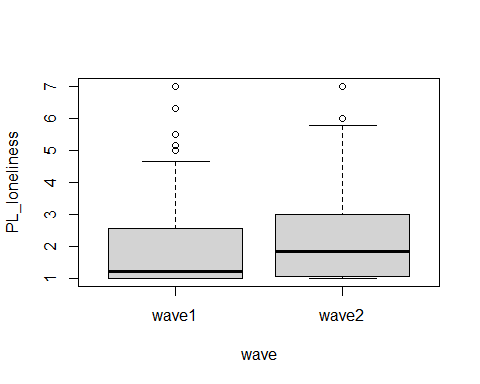


#Moderator descriptives
psych::describe(PLdat[T0, c("vpv_interpers", "ssl_total")])

## vars n mean sd median trimmed mad min max range skew kurtosis se
## vpv_interpers 1 169 69.96 6.99 70 69.89 5.93 54 90 36 0.14 -0.27 0.54
## ssl_total 2 178 23.27 5.63 23 23.35 5.93 5 35 30 -0.17 -0.35 0.42

table(PLdat$groupMean[T0], useNA = "always")

##
## 0 1 <NA>
## 140 44 0

psych::describe(PLdat[T2, c("vpv_interpers", "stressors_amount", "stressors_burden", "mspss_total")])

## vars n mean sd median trimmed mad min max range skew kurtosis se
## vpv_interpers 1 191 68.64 8.10 68.00 68.94 7.41 43 89 46 -0.31 0.40 0.59
## stressors_amount 2 195 10.68 3.19 11.00 10.81 2.97 1 17 16 -0.49 0.32 0.23
## stressors_burden 3 194 3.24 0.59 3.28 3.25 0.53 1 5 4 -0.36 1.06 0.04
## mspss_total 4 191 65.72 14.08 70.00 67.20 13.34 22 84 62 -0.88 0.20 1.02

#Covariates + compliance descriptives
psych::describe(PLdat[T0, c("age", "compliance_prop")])

## vars n mean sd median trimmed mad min max range skew kurtosis se
## age 1 183 13.92 1.84 14.00 13.76 2.97 11.00 19.00 8.00 0.50 -0.90 0.14
## compliance_prop 2 184 0.55 0.19 0.55 0.56 0.22 0.13 0.98 0.85 -0.13 -0.77 0.01

table(PLdat$sex[T0], useNA = "always")

##
## 1 2 <NA>
## 139 45 0

psych::describe(PLdat[T2, c("age", "compliance_prop")])

## vars n mean sd median trimmed mad min max range skew kurtosis se
## age 1 204 16.47 1.95 16.00 16.35 1.48 14.00 21 7.00 0.45 -1.05 0.14
## compliance_prop 2 204 0.42 0.24 0.39 0.41 0.28 0.02 1 0.98 0.32 -0.98 0.02

table(PLdat$sex[T2], useNA = "always")

##
## 1 2 <NA>
## 161 43 0

#Correlation table
CorDatT0 <- subset(PLdat[T0, c("PL_momentPA", "PL_momentNA", "PL_loneliness", "PL_pleasant", "PL_excluded", "PL_want", "PL_aloneprop",
 "vpv_interpers", "ssl_total", "age")])
apa.cor.table(CorDatT0, filename = "COVID_cortable_T0T2.doc", table.number = 4)

##
##
## Table 4
##
## Means, standard deviations, and correlations with confidence intervals
##
##
## Variable M SD 1 2 3 4 5 6 7
## 1. PL_momentPA 5.25 1.12
##
## 2. PL_momentNA 1.93 0.98 -.54**
## [-.63, -.43]
##
## 3. PL_loneliness 2.00 1.35 -.23** .47**
## [-.36, -.08] [.35, .58]
##
## 4. PL_pleasant 5.19 1.60 .05 -.01 -.18*
## [-.10, .19] [-.15, .14] [-.32, -.04]
##
## 5. PL_excluded 1.36 0.82 -.23** .30** .24** -.15*
## [-.36, -.09] [.16, .43] [.10, .38] [-.29, -.01]
##
## 6. PL_want 4.58 1.68 -.07 .02 -.18* .70** .02
## [-.22, .07] [-.12, .17] [-.32, -.04] [.62, .77] [-.12, .16]
##
## 7. PL_aloneprop 0.18 0.12 -.31** .20** .06 .15* .02 .18*
## [-.43, -.17] [.06, .33] [-.08, .21] [.00, .29] [-.13, .16] [.04, .32]
##
## 8. vpv_interpers 69.96 6.99 .25** -.14 -.07 .05 -.18* .05 -.23**
## [.10, .39] [-.29, .01] [-.22, .08] [-.10, .20] [-.33, -.03] [-.11, .20] [-.36, -.08]
##
## 9. ssl_total 23.27 5.63 .23** -.13 -.03 -.00 -.22** -.06 -.14
## [.08, .36] [-.27, .02] [-.17, .12] [-.15, .14] [-.35, -.07] [-.21, .09] [-.28, .01]
##
## 10. age 13.92 1.84 -.39** .27** .10 .17* .11 .20** .39**
## [-.51, -.26] [.13, .40] [-.05, .24] [.02, .31] [-.04, .25] [.05, .33] [.26, .51]
##
## 8 9
##
##
##
##
##
##
##
##
##
##
##
##
##
##
##
##
##
##
##
##
##
##
##
## .51**
## [.39, .61]
##
## -.13 -.12
## [-.27, .02] [-.26, .03]
##
##
## Note. M and SD are used to represent mean and standard deviation, respectively.
## Values in square brackets indicate the 95% confidence interval.
## The confidence interval is a plausible range of population correlations
## that could have caused the sample correlation (Cumming, 2014).
## * indicates p < .05. ** indicates p < .01.
##

CorDatT2 <- subset(PLdat[T2, c("PL_momentPA", "PL_momentNA", "PL_loneliness", "PL_pleasant", "PL_excluded", "PL_want", "PL_aloneprop",
 "vpv_interpers","stressors_amount", "stressors_burden", "mspss_total", "age")])
apa.cor.table(CorDatT2, filename = "COVID_cortableT2.doc", table.number = 5)

##
##
## Table 5
##
## Means, standard deviations, and correlations with confidence intervals
##
##
## Variable M SD 1 2 3 4 5 6
## 1. PL_momentPA 4.63 1.09
##
## 2. PL_momentNA 2.30 1.02 -.74**
## [-.80, -.68]
##
## 3. PL_loneliness 2.29 1.43 -.52** .67**
## [-.61, -.41] [.58, .74]
##
## 4. PL_pleasant 4.89 1.46 .27** -.25** -.43**
## [.14, .39] [-.37, -.11] [-.53, -.31]
##
## 5. PL_excluded 1.47 0.91 -.43** .48** .44** -.19**
## [-.54, -.31] [.37, .58] [.32, .54] [-.32, -.05]
##
## 6. PL_want 4.44 1.54 .08 -.04 -.29** .80** .03
## [-.05, .22] [-.18, .10] [-.41, -.16] [.75, .85] [-.10, .17]
##
## 7. PL_aloneprop 0.44 0.22 -.10 .13 .12 .05 .02 .02
## [-.24, .04] [-.00, .27] [-.02, .25] [-.09, .18] [-.12, .16] [-.12, .15]
##
## 8. vpv_interpers 68.64 8.10 .26** -.18* -.21** .03 -.33** -.05
## [.12, .39] [-.31, -.04] [-.34, -.07] [-.11, .17] [-.45, -.20] [-.19, .09]
##
## 9. stressors_amount 10.68 3.19 -.16* .16* .14 -.04 -.01 -.05
## [-.29, -.02] [.02, .30] [-.00, .28] [-.18, .10] [-.15, .13] [-.19, .09]
##
## 10. stressors_burden 3.24 0.59 -.24** .20** .15* -.11 .04 -.15*
## [-.36, -.10] [.06, .33] [.01, .29] [-.25, .03] [-.10, .18] [-.28, -.01]
##
## 11. mspss_total 65.72 14.08 .34** -.27** -.22** .02 -.35** -.07
## [.21, .46] [-.40, -.14] [-.36, -.09] [-.12, .17] [-.47, -.22] [-.21, .07]
##
## 12. age 16.47 1.95 -.12 .05 .07 -.08 .07 -.10
## [-.26, .01] [-.08, .19] [-.06, .21] [-.22, .05] [-.07, .20] [-.24, .03]
##
## 7 8 9 10 11
##
##
##
##
##
##
##
##
##
##
##
##
##
##
##
##
##
##
##
##
## -.09
## [-.23, .05]
##
## .03 -.09
## [-.11, .17] [-.23, .06]
##
## .11 -.12 .21**
## [-.03, .25] [-.26, .02] [.07, .34]
##
## -.22** .47** -.07 -.24**
## [-.35, -.09] [.35, .57] [-.21, .07] [-.37, -.10]
##
## .15* -.08 -.01 .29** -.18*
## [.02, .29] [-.22, .06] [-.15, .13] [.15, .41] [-.31, -.04]
##
##
## Note. M and SD are used to represent mean and standard deviation, respectively.
## Values in square brackets indicate the 95% confidence interval.
## The confidence interval is a plausible range of population correlations
## that could have caused the sample correlation (Cumming, 2014).
## * indicates p < .05. ** indicates p < .01.
##

#Reliability of person-level questionnaires
ssldat <- PLdat[T0, c("ssl_invite", "ssl_visit", "ssl_affection", "ssl_comfort", "ssl_compliment", "ssl_interest", "ssl_offerhelp",
 "ssl_ease", "ssl_advice", "ssl_confidence", "ssl_askhelp")]
psych::alpha(ssldat, na.rm=T)

##
## Reliability analysis
## Call: psych::alpha(x = ssldat, na.rm = T)
##
## raw_alpha std.alpha G6(smc) average_r S/N ase mean sd median_r
## 0.82 0.82 0.83 0.29 4.6 0.02 2 0.47 0.3
##
## 95% confidence boundaries
## lower alpha upper
## Feldt 0.78 0.82 0.86
## Duhachek 0.78 0.82 0.86
##
## Reliability if an item is dropped:
## raw_alpha std.alpha G6(smc) average_r S/N alpha se var.r med.r
## ssl_invite 0.81 0.81 0.82 0.30 4.4 0.021 0.0082 0.31
## ssl_visit 0.81 0.81 0.81 0.29 4.2 0.021 0.0095 0.30
## ssl_affection 0.81 0.81 0.82 0.29 4.2 0.021 0.0102 0.30
## ssl_comfort 0.81 0.81 0.81 0.30 4.3 0.021 0.0069 0.30
## ssl_compliment 0.81 0.81 0.81 0.29 4.2 0.021 0.0090 0.29
## ssl_interest 0.80 0.80 0.81 0.29 4.1 0.022 0.0098 0.29
## ssl_offerhelp 0.80 0.80 0.81 0.28 4.0 0.022 0.0087 0.29
## ssl_ease 0.79 0.79 0.80 0.28 3.9 0.023 0.0073 0.29
## ssl_advice 0.80 0.80 0.81 0.29 4.0 0.022 0.0097 0.28
## ssl_confidence 0.81 0.81 0.81 0.30 4.2 0.021 0.0088 0.30
## ssl_askhelp 0.82 0.81 0.82 0.30 4.4 0.020 0.0079 0.31
##
## Item statistics
## n raw.r std.r r.cor r.drop mean sd
## ssl_invite 178 0.52 0.52 0.45 0.40 2.0 0.73
## ssl_visit 178 0.60 0.59 0.54 0.48 1.7 0.84
## ssl_affection 178 0.58 0.59 0.52 0.48 2.1 0.71
## ssl_comfort 178 0.55 0.55 0.49 0.43 1.9 0.81
## ssl_compliment 179 0.59 0.59 0.54 0.48 1.8 0.75
## ssl_interest 177 0.62 0.63 0.58 0.52 1.9 0.75
## ssl_offerhelp 175 0.67 0.65 0.62 0.55 1.9 0.87
## ssl_ease 178 0.71 0.70 0.68 0.61 2.0 0.83
## ssl_advice 175 0.65 0.65 0.60 0.55 2.1 0.76
## ssl_confidence 178 0.58 0.58 0.53 0.47 2.3 0.77
## ssl_askhelp 177 0.51 0.52 0.45 0.39 1.9 0.83
##
## Non missing response frequency for each item
## 0 1 2 3 miss
## ssl_invite 0.02 0.20 0.52 0.26 0.03
## ssl_visit 0.09 0.31 0.46 0.15 0.03
## ssl_affection 0.01 0.17 0.50 0.33 0.03
## ssl_comfort 0.03 0.29 0.43 0.26 0.03
## ssl_compliment 0.03 0.32 0.49 0.17 0.03
## ssl_interest 0.03 0.28 0.50 0.19 0.04
## ssl_offerhelp 0.07 0.22 0.45 0.25 0.05
## ssl_ease 0.03 0.25 0.39 0.33 0.03
## ssl_advice 0.02 0.19 0.46 0.33 0.05
## ssl_confidence 0.02 0.13 0.40 0.44 0.03
## ssl_askhelp 0.03 0.27 0.42 0.28 0.04

psych::omega(ssldat, na.rm=T)


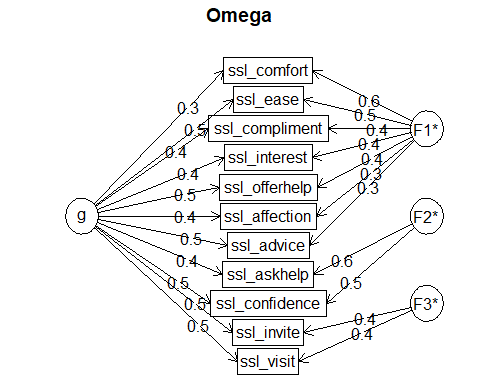


## Omega
## Call: omegah(m = m, nfactors = nfactors, fm = fm, key = key, flip = flip,
## digits = digits, title = title, sl = sl, labels = labels,
## plot = plot, n.obs = n.obs, rotate = rotate, Phi = Phi, option = option,
## covar = covar, na.rm = ..1)
## Alpha: 0.82
## G.6: 0.83
## Omega Hierarchical: 0.53
## Omega H asymptotic: 0.62
## Omega Total 0.85
##
## Schmid Leiman Factor loadings greater than 0.2
## g F1* F2* F3* h2 u2 p2
## ssl_invite 0.52 0.44 0.49 0.51 0.55
## ssl_visit 0.50 0.40 0.45 0.55 0.55
## ssl_affection 0.38 0.35 0.28 0.72 0.51
## ssl_comfort 0.29 0.64 0.50 0.50 0.17
## ssl_compliment 0.38 0.39 0.32 0.68 0.47
## ssl_interest 0.42 0.36 0.33 0.67 0.52
## ssl_offerhelp 0.48 0.36 0.39 0.61 0.59
## ssl_ease 0.48 0.52 0.52 0.48 0.44
## ssl_advice 0.48 0.28 0.36 0.64 0.64
## ssl_confidence 0.47 0.46 0.43 0.57 0.50
## ssl_askhelp 0.41 0.57 0.49 0.51 0.34
##
## With eigenvalues of:
## g F1* F2* F3*
## 2.13 1.34 0.65 0.44
##
## general/max 1.59 max/min = 3.04
## mean percent general = 0.48 with sd = 0.13 and cv of 0.27
## Explained Common Variance of the general factor = 0.47
##
## The degrees of freedom are 25 and the fit is 0.26
## The number of observations was 184 with Chi Square = 45.26 with prob < 0.0078
## The root mean square of the residuals is 0.04
## The df corrected root mean square of the residuals is 0.06
## RMSEA index = 0.066 and the 10 % confidence intervals are 0.034 0.097
## BIC = -85.11
##
## Compare this with the adequacy of just a general factor and no group factors
## The degrees of freedom for just the general factor are 44 and the fit is 0.87
## The number of observations was 184 with Chi Square = 155.01 with prob < 2.8e-14
## The root mean square of the residuals is 0.14
## The df corrected root mean square of the residuals is 0.16
##
## RMSEA index = 0.117 and the 10 % confidence intervals are 0.098 0.138
## BIC = -74.44
##
## Measures of factor score adequacy
## g F1* F2* F3*
## Correlation of scores with factors 0.76 0.76 0.67 0.56
## Multiple R square of scores with factors 0.57 0.57 0.45 0.31
## Minimum correlation of factor score estimates 0.14 0.14 -0.11 -0.38
##
## Total, General and Subset omega for each subset
## g F1* F2* F3*
## Omega total for total scores and subscales 0.85 0.78 0.63 0.62
## Omega general for total scores and subscales 0.53 0.39 0.26 0.37
## Omega group for total scores and subscales 0.23 0.39 0.37 0.26

vpvdat.T0 <- PLdat[T0, c("vpv_meetfriends", "vpv_recogniseemotion", "vpv_class", "vpv_help", "vpv_easycontact", "vpv_hurt", "vpv_questions",
 "vpv_sensitivities", "vpv_standup", "vpv_said", "vpv_seekcontact", "vpv_wishes", "vpv_getalong","vpv_interest",
 "vpv_talkconcerns","vpv_adapt", "vpv_goodcontact", "vpv_learn")]
psych::alpha(vpvdat.T0, na.rm=T)

##
## Reliability analysis
## Call: psych::alpha(x = vpvdat.T0, na.rm = T)
##
## raw_alpha std.alpha G6(smc) average_r S/N ase mean sd median_r
## 0.8 0.81 0.85 0.2 4.4 0.021 3.9 0.39 0.18
##
## 95% confidence boundaries
## lower alpha upper
## Feldt 0.76 0.8 0.84
## Duhachek 0.76 0.8 0.85
##
## Reliability if an item is dropped:
## raw_alpha std.alpha G6(smc) average_r S/N alpha se var.r med.r
## vpv_meetfriends 0.80 0.81 0.84 0.20 4.2 0.022 0.013 0.18
## vpv_recogniseemotion 0.80 0.81 0.84 0.20 4.2 0.022 0.015 0.18
## vpv_class 0.79 0.80 0.83 0.19 4.1 0.023 0.014 0.17
## vpv_help 0.79 0.80 0.83 0.19 4.0 0.022 0.015 0.17
## vpv_easycontact 0.80 0.81 0.84 0.20 4.3 0.021 0.014 0.18
## vpv_hurt 0.80 0.81 0.84 0.20 4.2 0.022 0.014 0.18
## vpv_questions 0.80 0.81 0.84 0.21 4.4 0.021 0.014 0.19
## vpv_sensitivities 0.79 0.80 0.83 0.19 4.0 0.023 0.013 0.17
## vpv_standup 0.79 0.80 0.83 0.19 4.1 0.022 0.015 0.17
## vpv_said 0.79 0.80 0.83 0.19 4.1 0.022 0.014 0.18
## vpv_seekcontact 0.79 0.81 0.84 0.20 4.1 0.022 0.014 0.18
## vpv_wishes 0.79 0.80 0.83 0.19 4.0 0.022 0.015 0.17
## vpv_getalong 0.79 0.80 0.83 0.19 4.0 0.023 0.015 0.17
## vpv_interest 0.79 0.80 0.83 0.19 4.0 0.022 0.014 0.17
## vpv_talkconcerns 0.79 0.80 0.83 0.20 4.1 0.022 0.014 0.17
## vpv_adapt 0.81 0.82 0.85 0.21 4.5 0.021 0.013 0.20
## vpv_goodcontact 0.79 0.80 0.83 0.19 3.9 0.023 0.013 0.17
## vpv_learn 0.80 0.81 0.84 0.20 4.3 0.021 0.015 0.19
##
## Item statistics
## n raw.r std.r r.cor r.drop mean sd
## vpv_meetfriends 177 0.47 0.46 0.42 0.37 4.2 0.80
## vpv_recogniseemotion 178 0.44 0.45 0.40 0.34 3.8 0.78
## vpv_class 177 0.56 0.53 0.50 0.45 3.8 1.03
## vpv_help 178 0.53 0.56 0.53 0.46 4.0 0.72
## vpv_easycontact 175 0.43 0.38 0.32 0.29 3.3 1.09
## vpv_hurt 178 0.43 0.45 0.41 0.33 3.9 0.79
## vpv_questions 178 0.37 0.35 0.28 0.26 3.4 0.87
## vpv_sensitivities 176 0.58 0.61 0.60 0.51 4.2 0.69
## vpv_standup 176 0.54 0.52 0.48 0.44 3.4 0.89
## vpv_said 177 0.51 0.52 0.49 0.42 4.0 0.75
## vpv_seekcontact 177 0.49 0.49 0.45 0.40 4.1 0.79
## vpv_wishes 177 0.55 0.58 0.54 0.48 4.0 0.67
## vpv_getalong 177 0.58 0.57 0.54 0.50 4.0 0.74
## vpv_interest 175 0.55 0.58 0.55 0.47 4.1 0.60
## vpv_talkconcerns 176 0.53 0.51 0.48 0.42 3.9 0.97
## vpv_adapt 177 0.26 0.27 0.18 0.14 3.9 0.81
## vpv_goodcontact 175 0.60 0.61 0.60 0.53 4.0 0.75
## vpv_learn 175 0.41 0.40 0.34 0.31 3.7 0.86
##
## Non missing response frequency for each item
## 1 2 3 4 5 miss
## vpv_meetfriends 0.00 0.03 0.14 0.40 0.43 0.04
## vpv_recogniseemotion 0.01 0.04 0.26 0.52 0.17 0.03
## vpv_class 0.03 0.07 0.23 0.37 0.31 0.04
## vpv_help 0.00 0.03 0.16 0.57 0.24 0.03
## vpv_easycontact 0.06 0.16 0.35 0.27 0.15 0.05
## vpv_hurt 0.00 0.04 0.24 0.49 0.23 0.03
## vpv_questions 0.02 0.12 0.40 0.38 0.07 0.03
## vpv_sensitivities 0.00 0.01 0.13 0.53 0.33 0.04
## vpv_standup 0.02 0.12 0.38 0.39 0.10 0.04
## vpv_said 0.00 0.03 0.19 0.53 0.25 0.04
## vpv_seekcontact 0.00 0.03 0.16 0.47 0.33 0.04
## vpv_wishes 0.00 0.02 0.17 0.60 0.21 0.04
## vpv_getalong 0.00 0.03 0.18 0.55 0.23 0.04
## vpv_interest 0.00 0.01 0.13 0.65 0.21 0.05
## vpv_talkconcerns 0.02 0.07 0.20 0.39 0.32 0.04
## vpv_adapt 0.02 0.02 0.24 0.52 0.20 0.04
## vpv_goodcontact 0.01 0.02 0.16 0.56 0.25 0.05
## vpv_learn 0.02 0.05 0.29 0.48 0.17 0.05

psych::omega(vpvdat.T0, na.rm=T)


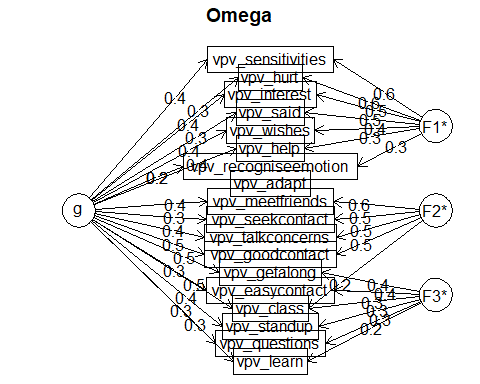


## Omega
## Call: omegah(m = m, nfactors = nfactors, fm = fm, key = key, flip = flip,
## digits = digits, title = title, sl = sl, labels = labels,
## plot = plot, n.obs = n.obs, rotate = rotate, Phi = Phi, option = option,
## covar = covar, na.rm = ..1)
## Alpha: 0.81
## G.6: 0.85
## Omega Hierarchical: 0.51
## Omega H asymptotic: 0.6
## Omega Total 0.85
##
## Schmid Leiman Factor loadings greater than 0.2
## g F1* F2* F3* h2 u2 p2
## vpv_meetfriends 0.37 0.57 0.48 0.52 0.29
## vpv_recogniseemotion 0.25 0.33 0.18 0.82 0.35
## vpv_class 0.46 0.21 0.34 0.37 0.63 0.57
## vpv_help 0.37 0.33 0.27 0.73 0.51
## vpv_easycontact 0.33 0.41 0.29 0.71 0.39
## vpv_hurt 0.26 0.57 0.42 0.58 0.16
## vpv_questions 0.26 0.27 0.14 0.86 0.47
## vpv_sensitivities 0.41 0.60 0.54 0.46 0.31
## vpv_standup 0.40 0.31 0.28 0.72 0.58
## vpv_said 0.35 0.45 0.33 0.67 0.36
## vpv_seekcontact 0.34 0.53 0.41 0.59 0.28
## vpv_wishes 0.40 0.39 0.33 0.67 0.48
## vpv_getalong 0.51 0.43 0.45 0.55 0.57
## vpv_interest 0.39 0.49 0.41 0.59 0.37
## vpv_talkconcerns 0.37 0.53 0.42 0.58 0.33
## vpv_adapt 0.06 0.94 0.17
## vpv_goodcontact 0.47 0.48 0.48 0.52 0.46
## vpv_learn 0.28 0.23 0.15 0.85 0.52
##
## With eigenvalues of:
## g F1* F2* F3*
## 2.37 1.61 1.27 0.75
##
## general/max 1.47 max/min = 2.15
## mean percent general = 0.4 with sd = 0.13 and cv of 0.33
## Explained Common Variance of the general factor = 0.4
##
## The degrees of freedom are 102 and the fit is 0.91
## The number of observations was 184 with Chi Square = 159.3 with prob < 0.00025
## The root mean square of the residuals is 0.05
## The df corrected root mean square of the residuals is 0.07
## RMSEA index = 0.055 and the 10 % confidence intervals are 0.038 0.072
## BIC = -372.62
##
## Compare this with the adequacy of just a general factor and no group factors
## The degrees of freedom for just the general factor are 135 and the fit is 2.2
## The number of observations was 184 with Chi Square = 386.28 with prob < 4.6e-26
## The root mean square of the residuals is 0.13
## The df corrected root mean square of the residuals is 0.13
##
## RMSEA index = 0.1 and the 10 % confidence intervals are 0.089 0.113
## BIC = -317.74
##
## Measures of factor score adequacy
## g F1* F2* F3*
## Correlation of scores with factors 0.74 0.78 0.76 0.62
## Multiple R square of scores with factors 0.54 0.62 0.58 0.38
## Minimum correlation of factor score estimates 0.08 0.23 0.17 -0.24
##
## Total, General and Subset omega for each subset
## g F1* F2* F3*
## Omega total for total scores and subscales 0.85 0.75 0.75 0.67
## Omega general for total scores and subscales 0.51 0.27 0.26 0.38
## Omega group for total scores and subscales 0.25 0.48 0.49 0.30

vpvdat.T2 <- PLdat[T2, c("vpv_meetfriends", "vpv_recogniseemotion", "vpv_class", "vpv_help", "vpv_easycontact", "vpv_hurt", "vpv_questions",
 "vpv_sensitivities", "vpv_standup", "vpv_said", "vpv_seekcontact", "vpv_wishes", "vpv_getalong","vpv_interest",
 "vpv_talkconcerns","vpv_adapt", "vpv_goodcontact", "vpv_learn")]
psych::alpha(vpvdat.T2, na.rm=T)

##
## Reliability analysis
## Call: psych::alpha(x = vpvdat.T2, na.rm = T)
##
## raw_alpha std.alpha G6(smc) average_r S/N ase mean sd median_r
## 0.82 0.83 0.86 0.21 4.9 0.018 3.8 0.45 0.2
##
## 95% confidence boundaries
## lower alpha upper
## Feldt 0.79 0.82 0.86
## Duhachek 0.79 0.82 0.86
##
## Reliability if an item is dropped:
## raw_alpha std.alpha G6(smc) average_r S/N alpha se var.r med.r
## vpv_meetfriends 0.82 0.83 0.86 0.22 4.7 0.019 0.015 0.21
## vpv_recogniseemotion 0.82 0.83 0.86 0.22 4.8 0.018 0.016 0.20
## vpv_class 0.81 0.82 0.85 0.21 4.5 0.020 0.016 0.20
## vpv_help 0.82 0.82 0.85 0.21 4.6 0.019 0.017 0.20
## vpv_easycontact 0.82 0.83 0.86 0.22 4.8 0.018 0.017 0.21
## vpv_hurt 0.82 0.82 0.85 0.21 4.6 0.019 0.015 0.20
## vpv_questions 0.82 0.82 0.86 0.21 4.6 0.019 0.018 0.20
## vpv_sensitivities 0.82 0.83 0.86 0.22 4.8 0.018 0.016 0.20
## vpv_standup 0.82 0.82 0.86 0.22 4.7 0.019 0.017 0.20
## vpv_said 0.81 0.82 0.85 0.21 4.6 0.019 0.015 0.20
## vpv_seekcontact 0.81 0.81 0.85 0.21 4.4 0.020 0.015 0.20
## vpv_wishes 0.81 0.82 0.86 0.21 4.6 0.019 0.017 0.20
## vpv_getalong 0.81 0.82 0.85 0.21 4.5 0.019 0.017 0.20
## vpv_interest 0.81 0.82 0.85 0.21 4.4 0.019 0.016 0.20
## vpv_talkconcerns 0.82 0.82 0.86 0.22 4.7 0.019 0.015 0.20
## vpv_adapt 0.82 0.83 0.86 0.22 4.9 0.018 0.017 0.21
## vpv_goodcontact 0.81 0.82 0.85 0.21 4.5 0.019 0.015 0.20
## vpv_learn 0.82 0.83 0.86 0.22 4.8 0.018 0.017 0.20
##
## Item statistics
## n raw.r std.r r.cor r.drop mean sd
## vpv_meetfriends 195 0.49 0.46 0.43 0.38 3.7 1.08
## vpv_recogniseemotion 195 0.41 0.42 0.37 0.32 4.0 0.84
## vpv_class 195 0.61 0.58 0.55 0.51 3.6 1.04
## vpv_help 195 0.50 0.52 0.48 0.42 4.0 0.83
## vpv_easycontact 194 0.47 0.42 0.37 0.34 3.2 1.22
## vpv_hurt 193 0.50 0.51 0.48 0.40 3.7 0.97
## vpv_questions 195 0.51 0.50 0.45 0.41 3.4 1.01
## vpv_sensitivities 194 0.42 0.45 0.40 0.33 4.1 0.82
## vpv_standup 195 0.48 0.47 0.43 0.38 3.4 0.93
## vpv_said 194 0.53 0.55 0.53 0.45 3.9 0.83
## vpv_seekcontact 194 0.65 0.64 0.63 0.58 4.0 0.91
## vpv_wishes 194 0.51 0.54 0.50 0.44 4.0 0.71
## vpv_getalong 193 0.57 0.58 0.55 0.50 4.0 0.79
## vpv_interest 194 0.60 0.63 0.62 0.54 4.0 0.69
## vpv_talkconcerns 194 0.50 0.48 0.45 0.40 4.0 0.95
## vpv_adapt 194 0.37 0.37 0.30 0.28 4.0 0.81
## vpv_goodcontact 193 0.59 0.59 0.57 0.52 3.8 0.79
## vpv_learn 194 0.41 0.43 0.37 0.33 3.7 0.80
##
## Non missing response frequency for each item
## 1 2 3 4 5 miss
## vpv_meetfriends 0.04 0.08 0.25 0.35 0.28 0.04
## vpv_recogniseemotion 0.01 0.05 0.17 0.48 0.30 0.04
## vpv_class 0.02 0.17 0.21 0.42 0.19 0.04
## vpv_help 0.00 0.05 0.19 0.47 0.29 0.04
## vpv_easycontact 0.07 0.27 0.23 0.25 0.18 0.05
## vpv_hurt 0.03 0.10 0.19 0.49 0.20 0.05
## vpv_questions 0.03 0.17 0.33 0.33 0.14 0.04
## vpv_sensitivities 0.00 0.05 0.15 0.46 0.34 0.05
## vpv_standup 0.03 0.14 0.37 0.36 0.09 0.04
## vpv_said 0.02 0.03 0.19 0.53 0.23 0.05
## vpv_seekcontact 0.01 0.07 0.16 0.43 0.33 0.05
## vpv_wishes 0.00 0.03 0.17 0.58 0.23 0.05
## vpv_getalong 0.00 0.05 0.17 0.52 0.27 0.05
## vpv_interest 0.00 0.01 0.18 0.56 0.25 0.05
## vpv_talkconcerns 0.02 0.06 0.15 0.46 0.31 0.05
## vpv_adapt 0.01 0.03 0.19 0.52 0.25 0.05
## vpv_goodcontact 0.01 0.04 0.23 0.56 0.16 0.05
## vpv_learn 0.01 0.04 0.29 0.50 0.15 0.05

psych::omega(vpvdat.T2, na.rm=T)


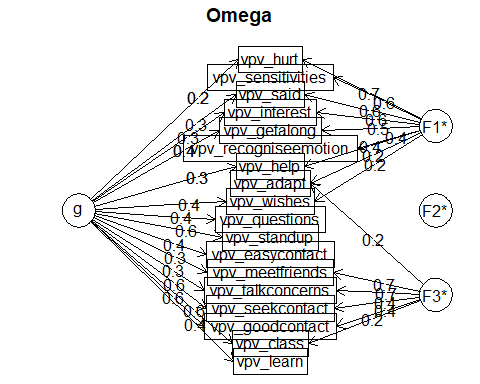


## Omega
## Call: omegah(m = m, nfactors = nfactors, fm = fm, key = key, flip = flip,
## digits = digits, title = title, sl = sl, labels = labels,
## plot = plot, n.obs = n.obs, rotate = rotate, Phi = Phi, option = option,
## covar = covar, na.rm = ..1)
## Alpha: 0.83
## G.6: 0.86
## Omega Hierarchical: 0.5
## Omega H asymptotic: 0.58
## Omega Total 0.86
##
## Schmid Leiman Factor loadings greater than 0.2
## g F1* F2* F3* h2 u2 p2
## vpv_meetfriends 0.28 0.73 0.61 0.39 0.12
## vpv_recogniseemotion 0.40 0.18 0.82 0.14
## vpv_class 0.57 0.23 0.39 0.61 0.84
## vpv_help 0.33 0.35 0.23 0.77 0.46
## vpv_easycontact 0.42 0.19 0.81 0.93
## vpv_hurt 0.23 0.66 0.52 0.48 0.10
## vpv_questions 0.43 0.22 0.78 0.84
## vpv_sensitivities 0.65 0.44 0.56 0.01
## vpv_standup 0.56 0.33 0.67 0.95
## vpv_said 0.27 0.64 0.48 0.52 0.15
## vpv_seekcontact 0.60 0.42 0.55 0.45 0.66
## vpv_wishes 0.45 0.23 0.26 0.74 0.75
## vpv_getalong 0.36 0.46 0.34 0.66 0.37
## vpv_interest 0.34 0.59 0.48 0.52 0.25
## vpv_talkconcerns 0.32 0.65 0.53 0.47 0.19
## vpv_adapt 0.25 0.22 0.12 0.88 0.10
## vpv_goodcontact 0.57 0.39 0.49 0.51 0.67
## vpv_learn 0.43 0.26 0.74 0.70
##
## With eigenvalues of:
## g F1* F2* F3*
## 2.77 2.27 0.08 1.50
##
## general/max 1.22 max/min = 29.1
## mean percent general = 0.46 with sd = 0.33 and cv of 0.73
## Explained Common Variance of the general factor = 0.42
##
## The degrees of freedom are 102 and the fit is 1.01
## The number of observations was 204 with Chi Square = 195.19 with prob < 8.1e-08
## The root mean square of the residuals is 0.05
## The df corrected root mean square of the residuals is 0.07
## RMSEA index = 0.067 and the 10 % confidence intervals are 0.053 0.081
## BIC = -347.26
##
## Compare this with the adequacy of just a general factor and no group factors
## The degrees of freedom for just the general factor are 135 and the fit is 2.89
## The number of observations was 204 with Chi Square = 565.71 with prob < 4.5e-54
## The root mean square of the residuals is 0.15
## The df corrected root mean square of the residuals is 0.16
##
## RMSEA index = 0.125 and the 10 % confidence intervals are 0.115 0.136
## BIC = -152.24
##
## Measures of factor score adequacy
## g F1* F2* F3*
## Correlation of scores with factors 0.85 0.87 0.19 0.83
## Multiple R square of scores with factors 0.71 0.76 0.04 0.70
## Minimum correlation of factor score estimates 0.43 0.51 -0.93 0.39
##
## Total, General and Subset omega for each subset
## g F1* F2* F3*
## Omega total for total scores and subscales 0.86 0.79 0.41 0.80
## Omega general for total scores and subscales 0.50 0.22 0.40 0.48
## Omega group for total scores and subscales 0.29 0.57 0.02 0.31

amountdat <- PLdat[T2, c("covid_symptoms_self", "covid_symptoms_family", "covid_risk_self", "covid_risk_family", "covid_job_family",
 "covid_job_self", "covid_healthcare", "covid_restriction", "covid_lesspeople", "covid_socialevents",
 "covid_hospital", "covid_funeral", "covid_hobby", "covid_move", "covid_tension_parents", "covid_tension_self",
 "covid_siblings", "covid_travel", "covid_basics", "covid_media", "covid_school", "covid_money")]
psych::alpha(amountdat, na.rm=T)

##
## Reliability analysis
## Call: psych::alpha(x = amountdat, na.rm = T)
##
## raw_alpha std.alpha G6(smc) average_r S/N ase mean sd median_r
## 0.66 0.65 0.7 0.078 1.9 0.033 0.49 0.15 0.074
##
## 95% confidence boundaries
## lower alpha upper
## Feldt 0.59 0.66 0.73
## Duhachek 0.60 0.66 0.73
##
## Reliability if an item is dropped:
## raw_alpha std.alpha G6(smc) average_r S/N alpha se var.r med.r
## covid_symptoms_self 0.66 0.64 0.69 0.078 1.8 0.034 0.0092 0.074
## covid_symptoms_family 0.65 0.64 0.68 0.077 1.7 0.035 0.0087 0.073
## covid_risk_self 0.67 0.66 0.70 0.084 1.9 0.033 0.0090 0.077
## covid_risk_family 0.65 0.64 0.69 0.077 1.8 0.034 0.0091 0.072
## covid_job_family 0.66 0.64 0.69 0.078 1.8 0.034 0.0092 0.074
## covid_job_self 0.66 0.65 0.69 0.081 1.8 0.033 0.0092 0.074
## covid_healthcare 0.67 0.66 0.70 0.083 1.9 0.033 0.0089 0.077
## covid_restriction 0.64 0.63 0.67 0.074 1.7 0.036 0.0085 0.070
## covid_lesspeople 0.65 0.62 0.67 0.073 1.6 0.035 0.0083 0.071
## covid_socialevents 0.65 0.63 0.67 0.075 1.7 0.035 0.0080 0.071
## covid_hospital 0.67 0.65 0.69 0.081 1.8 0.033 0.0091 0.075
## covid_funeral 0.66 0.65 0.69 0.080 1.8 0.033 0.0093 0.076
## covid_hobby 0.66 0.64 0.69 0.079 1.8 0.034 0.0086 0.076
## covid_move 0.65 0.64 0.69 0.078 1.8 0.034 0.0091 0.075
## covid_tension_parents 0.64 0.63 0.67 0.074 1.7 0.036 0.0086 0.071
## covid_tension_self 0.62 0.61 0.66 0.070 1.6 0.037 0.0082 0.070
## covid_siblings 0.66 0.64 0.69 0.079 1.8 0.034 0.0088 0.075
## covid_travel 0.65 0.64 0.68 0.077 1.7 0.034 0.0089 0.073
## covid_basics 0.67 0.66 0.70 0.084 1.9 0.033 0.0088 0.077
## covid_media 0.65 0.64 0.68 0.077 1.7 0.034 0.0091 0.074
## covid_school 0.65 0.63 0.67 0.074 1.7 0.035 0.0087 0.071
## covid_money 0.67 0.66 0.70 0.085 1.9 0.032 0.0083 0.076
##
## Item statistics
## n raw.r std.r r.cor r.drop mean sd
## covid_symptoms_self 195 0.360 0.35 0.292 0.223 0.318 0.47
## covid_symptoms_family 195 0.402 0.38 0.340 0.273 0.708 0.46
## covid_risk_self 195 0.140 0.19 0.078 0.065 0.062 0.24
## covid_risk_family 195 0.395 0.37 0.304 0.256 0.621 0.49
## covid_job_family 195 0.356 0.34 0.272 0.208 0.528 0.50
## covid_job_self 195 0.272 0.27 0.186 0.151 0.200 0.40
## covid_healthcare 195 0.091 0.20 0.104 0.053 0.015 0.12
## covid_restriction 195 0.482 0.45 0.426 0.349 0.554 0.50
## covid_lesspeople 195 0.448 0.49 0.488 0.378 0.923 0.27
## covid_socialevents 195 0.427 0.43 0.410 0.323 0.831 0.38
## covid_hospital 195 0.266 0.27 0.186 0.131 0.262 0.44
## covid_funeral 195 0.283 0.30 0.218 0.165 0.190 0.39
## covid_hobby 195 0.333 0.31 0.259 0.196 0.697 0.46
## covid_move 195 0.384 0.35 0.287 0.247 0.656 0.48
## covid_tension_parents 195 0.504 0.46 0.440 0.375 0.426 0.50
## covid_tension_self 195 0.590 0.56 0.567 0.473 0.564 0.50
## covid_siblings 195 0.354 0.31 0.241 0.206 0.508 0.50
## covid_travel 195 0.383 0.38 0.336 0.259 0.754 0.43
## covid_basics 195 0.102 0.19 0.100 0.048 0.031 0.17
## covid_media 195 0.374 0.39 0.340 0.265 0.826 0.38
## covid_school 195 0.433 0.45 0.419 0.343 0.877 0.33
## covid_money 195 0.126 0.16 0.071 0.021 0.128 0.34
##
## Non missing response frequency for each item
## 0 1 miss
## covid_symptoms_self 0.68 0.32 0.04
## covid_symptoms_family 0.29 0.71 0.04
## covid_risk_self 0.94 0.06 0.04
## covid_risk_family 0.38 0.62 0.04
## covid_job_family 0.47 0.53 0.04
## covid_job_self 0.80 0.20 0.04
## covid_healthcare 0.98 0.02 0.04
## covid_restriction 0.45 0.55 0.04
## covid_lesspeople 0.08 0.92 0.04
## covid_socialevents 0.17 0.83 0.04
## covid_hospital 0.74 0.26 0.04
## covid_funeral 0.81 0.19 0.04
## covid_hobby 0.30 0.70 0.04
## covid_move 0.34 0.66 0.04
## covid_tension_parents 0.57 0.43 0.04
## covid_tension_self 0.44 0.56 0.04
## covid_siblings 0.49 0.51 0.04
## covid_travel 0.25 0.75 0.04
## covid_basics 0.97 0.03 0.04
## covid_media 0.17 0.83 0.04
## covid_school 0.12 0.88 0.04
## covid_money 0.87 0.13 0.04

psych::omega(amountdat, na.rm=T)


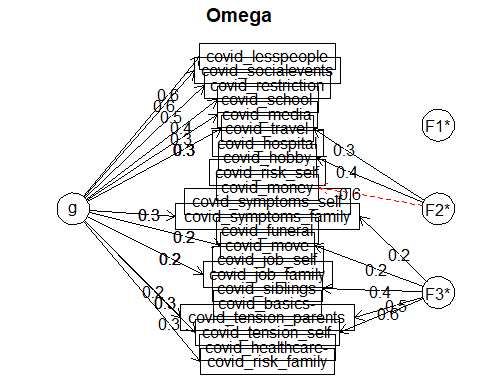


## Omega
## Call: omegah(m = m, nfactors = nfactors, fm = fm, key = key, flip = flip,
## digits = digits, title = title, sl = sl, labels = labels,
## plot = plot, n.obs = n.obs, rotate = rotate, Phi = Phi, option = option,
## covar = covar, na.rm = ..1)
## Alpha: 0.63
## G.6: 0.68
## Omega Hierarchical: 0.49
## Omega H asymptotic: 0.73
## Omega Total 0.67
##
## Schmid Leiman Factor loadings greater than 0.2
## g F1* F2* F3* h2 u2 p2
## covid_symptoms_self 0.20 0.09 0.91 0.33
## covid_symptoms_family 0.27 0.20 0.12 0.88 0.60
## covid_risk_self 0.02 0.98 0.47
## covid_risk_family 0.33 0.12 0.88 0.92
## covid_job_family 0.22 0.06 0.94 0.78
## covid_job_self 0.03 0.97 0.57
## covid_healthcare- 0.01 0.99 0.01
## covid_restriction 0.49 0.28 0.72 0.88
## covid_lesspeople 0.62 0.39 0.61 0.99
## covid_socialevents 0.59 0.38 0.62 0.92
## covid_hospital 0.03 0.97 0.63
## covid_funeral 0.06 0.94 0.20
## covid_hobby 0.38 0.20 0.80 0.16
## covid_move 0.21 0.22 0.10 0.90 0.46
## covid_tension_parents 0.22 0.51 0.32 0.68 0.16
## covid_tension_self 0.31 0.62 0.48 0.52 0.20
## covid_siblings 0.42 0.18 0.82 0.02
## covid_travel 0.28 0.30 0.20 0.80 0.41
## covid_basics- 0.03 0.97 0.13
## covid_media 0.28 0.11 0.89 0.68
## covid_school 0.43 0.21 0.79 0.90
## covid_money -0.62 0.39 0.61 0.00
##
## With eigenvalues of:
## g F1* F2* F3*
## 1.86 0.00 0.79 1.14
##
## general/max 1.64 max/min = 261.25
## mean percent general = 0.47 with sd = 0.33 and cv of 0.7
## Explained Common Variance of the general factor = 0.49
##
## The degrees of freedom are 168 and the fit is 1.13
## The number of observations was 204 with Chi Square = 217.79 with prob < 0.0058
## The root mean square of the residuals is 0.06
## The df corrected root mean square of the residuals is 0.07
## RMSEA index = 0.038 and the 10 % confidence intervals are 0.021 0.052
## BIC = -675.65
##
## Compare this with the adequacy of just a general factor and no group factors
## The degrees of freedom for just the general factor are 209 and the fit is 1.69
## The number of observations was 204 with Chi Square = 327.49 with prob < 3e-07
## The root mean square of the residuals is 0.08
## The df corrected root mean square of the residuals is 0.08
##
## RMSEA index = 0.052 and the 10 % confidence intervals are 0.042 0.064
## BIC = -784
##
## Measures of factor score adequacy
## g F1* F2* F3*
## Correlation of scores with factors 0.83 0.05 0.73 0.77
## Multiple R square of scores with factors 0.69 0.00 0.53 0.59
## Minimum correlation of factor score estimates 0.39 -1.00 0.07 0.18
##
## Total, General and Subset omega for each subset
## g F1* F2* F3*
## Omega total for total scores and subscales 0.67 NA 0.53 0.47
## Omega general for total scores and subscales 0.49 NA 0.52 0.20
## Omega group for total scores and subscales 0.10 NA 0.00 0.27

# burdendat <- PLdat[T1, c("covid_symptoms_self_burden", "covid_symptoms_family_burden", "covid_risk_self_burden", "covid_risk_family_burden",
# "covid_healthcare_burden", "covid_job_family_burden", "covid_job_self_burden", "covid_restriction_burden",
# "covid_lesspeople_burden", "covid_socialevents_burden","covid_hospital_burden", "covid_funeral_burden",
# "covid_hobby_burden", "covid_move_burden", "covid_tension_parents_burden", "covid_tension_self_burden",
# "covid_siblings_burden", "covid_travel_burden", "covid_basics_burden", "covid_media_burden",
# "covid_school_burden", "covid_money_burden")]
# psych::alpha(burdendat, na.rm=T)
# psych::omega(burdendat, na.rm=T)

mspssdat <- PLdat[T2, c("mspss_person_help", "mspss_person_joy", "mspss_family_help", "mspss_family_support", "mspss_person_comfort",
 "mspss_friends_help", "mspss_friends_count", "mspss_family_talk", "mspss_friends_joy", "mspss_person_feelings",
 "mspss_family_decide", "mspss_friends_talk")]
psych::alpha(mspssdat, na.rm=T)

##
## Reliability analysis
## Call: psych::alpha(x = mspssdat, na.rm = T)
##
## raw_alpha std.alpha G6(smc) average_r S/N ase mean sd median_r
## 0.9 0.9 0.96 0.43 9.1 0.011 5.5 1.2 0.34
##
## 95% confidence boundaries
## lower alpha upper
## Feldt 0.88 0.9 0.92
## Duhachek 0.88 0.9 0.92
##
## Reliability if an item is dropped:
## raw_alpha std.alpha G6(smc) average_r S/N alpha se var.r med.r
## mspss_person_help 0.89 0.89 0.96 0.43 8.5 0.012 0.060 0.34
## mspss_person_joy 0.89 0.89 0.96 0.43 8.2 0.012 0.061 0.33
## mspss_family_help 0.90 0.90 0.96 0.44 8.8 0.012 0.058 0.34
## mspss_family_support 0.89 0.90 0.96 0.44 8.6 0.012 0.060 0.34
## mspss_person_comfort 0.89 0.89 0.96 0.43 8.2 0.012 0.061 0.33
## mspss_friends_help 0.89 0.89 0.96 0.43 8.3 0.012 0.061 0.34
## mspss_friends_count 0.89 0.89 0.96 0.43 8.3 0.012 0.061 0.33
## mspss_family_talk 0.89 0.90 0.96 0.44 8.6 0.012 0.061 0.34
## mspss_friends_joy 0.89 0.89 0.96 0.43 8.2 0.012 0.064 0.33
## mspss_person_feelings 0.89 0.89 0.96 0.43 8.2 0.013 0.065 0.33
## mspss_family_decide 0.89 0.90 0.96 0.44 8.6 0.012 0.061 0.34
## mspss_friends_talk 0.89 0.89 0.96 0.42 8.0 0.012 0.063 0.30
##
## Item statistics
## n raw.r std.r r.cor r.drop mean sd
## mspss_person_help 195 0.69 0.67 0.66 0.61 5.3 1.9
## mspss_person_joy 195 0.74 0.72 0.72 0.67 5.6 1.8
## mspss_family_help 195 0.61 0.61 0.59 0.53 5.5 1.6
## mspss_family_support 194 0.65 0.65 0.64 0.57 5.0 1.8
## mspss_person_comfort 195 0.73 0.72 0.71 0.66 5.5 1.8
## mspss_friends_help 195 0.69 0.71 0.70 0.63 5.6 1.6
## mspss_friends_count 194 0.69 0.71 0.71 0.63 5.7 1.6
## mspss_family_talk 195 0.65 0.65 0.63 0.57 4.8 1.8
## mspss_friends_joy 194 0.71 0.73 0.71 0.65 5.8 1.6
## mspss_person_feelings 195 0.74 0.73 0.71 0.68 5.6 1.7
## mspss_family_decide 195 0.64 0.65 0.63 0.57 5.6 1.6
## mspss_friends_talk 194 0.74 0.76 0.75 0.68 5.6 1.6
##
## Non missing response frequency for each item
## 1 2 3 4 5 6 7 miss
## mspss_person_help 0.06 0.09 0.07 0.08 0.11 0.23 0.37 0.04
## mspss_person_joy 0.04 0.07 0.05 0.08 0.09 0.28 0.40 0.04
## mspss_family_help 0.03 0.05 0.04 0.13 0.11 0.30 0.34 0.04
## mspss_family_support 0.06 0.07 0.06 0.13 0.17 0.26 0.24 0.05
## mspss_person_comfort 0.04 0.09 0.03 0.09 0.12 0.24 0.41 0.04
## mspss_friends_help 0.03 0.04 0.04 0.13 0.14 0.27 0.36 0.04
## mspss_friends_count 0.03 0.03 0.05 0.09 0.13 0.25 0.43 0.05
## mspss_family_talk 0.08 0.08 0.05 0.16 0.17 0.28 0.17 0.04
## mspss_friends_joy 0.03 0.04 0.04 0.03 0.15 0.25 0.46 0.05
## mspss_person_feelings 0.04 0.05 0.03 0.11 0.10 0.23 0.44 0.04
## mspss_family_decide 0.04 0.04 0.03 0.06 0.19 0.29 0.35 0.04
## mspss_friends_talk 0.04 0.03 0.06 0.09 0.14 0.27 0.37 0.05

psych::omega(mspssdat, na.rm=T)


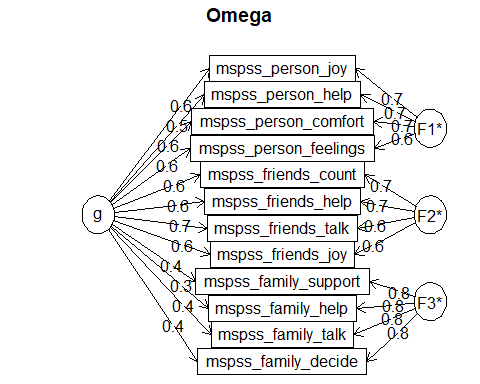


## Omega
## Call: omegah(m = m, nfactors = nfactors, fm = fm, key = key, flip = flip,
## digits = digits, title = title, sl = sl, labels = labels,
## plot = plot, n.obs = n.obs, rotate = rotate, Phi = Phi, option = option,
## covar = covar, na.rm = ..1)
## Alpha: 0.9
## G.6: 0.96
## Omega Hierarchical: 0.59
## Omega H asymptotic: 0.61
## Omega Total 0.97
##
## Schmid Leiman Factor loadings greater than 0.2
## g F1* F2* F3* h2 u2 p2
## mspss_person_help 0.54 0.72 0.81 0.19 0.36
## mspss_person_joy 0.58 0.74 0.88 0.12 0.39
## mspss_family_help 0.35 0.82 0.80 0.20 0.15
## mspss_family_support 0.39 0.84 0.86 0.14 0.17
## mspss_person_comfort 0.59 0.71 0.85 0.15 0.41
## mspss_friends_help 0.64 0.66 0.85 0.15 0.49
## mspss_friends_count 0.65 0.69 0.90 0.10 0.47
## mspss_family_talk 0.39 0.79 0.78 0.22 0.19
## mspss_friends_joy 0.64 0.56 0.74 0.26 0.55
## mspss_person_feelings 0.57 0.64 0.74 0.26 0.44
## mspss_family_decide 0.40 0.78 0.79 0.21 0.20
## mspss_friends_talk 0.67 0.61 0.83 0.17 0.54
##
## With eigenvalues of:
## g F1* F2* F3*
## 3.6 2.0 1.6 2.6
##
## general/max 1.36 max/min = 1.63
## mean percent general = 0.36 with sd = 0.15 and cv of 0.41
## Explained Common Variance of the general factor = 0.36
##
## The degrees of freedom are 33 and the fit is 0.56
## The number of observations was 204 with Chi Square = 109.23 with prob < 4.3e-10
## The root mean square of the residuals is 0.02
## The df corrected root mean square of the residuals is 0.02
## RMSEA index = 0.106 and the 10 % confidence intervals are 0.085 0.129
## BIC = -66.27
##
## Compare this with the adequacy of just a general factor and no group factors
## The degrees of freedom for just the general factor are 54 and the fit is 8.26
## The number of observations was 204 with Chi Square = 1631.79 with prob < 5.9e-306
## The root mean square of the residuals is 0.28
## The df corrected root mean square of the residuals is 0.31
##
## RMSEA index = 0.378 and the 10 % confidence intervals are 0.364 0.395
## BIC = 1344.61
##
## Measures of factor score adequacy
## g F1* F2* F3*
## Correlation of scores with factors 0.79 0.85 0.78 0.93
## Multiple R square of scores with factors 0.63 0.73 0.61 0.87
## Minimum correlation of factor score estimates 0.26 0.45 0.21 0.73
##
## Total, General and Subset omega for each subset
## g F1* F2* F3*
## Omega total for total scores and subscales 0.97 0.95 0.95 0.94
## Omega general for total scores and subscales 0.59 0.38 0.49 0.17
## Omega group for total scores and subscales 0.36 0.57 0.46 0.77

# ##GRAND MEAN CENTER MODERATORS & COVARIATES##
# PLdat$c_ssl_total <- PLdat$ssl_total - mean(PLdat$ssl_total, na.rm=T)
# PLdat$c_vpv_interpers <- PLdat$vpv_interpers - mean(PLdat$vpv_interpers, na.rm=T)
# PLdat$c_stressors_amount <- PLdat$stressors_amount - mean(PLdat$stressors_amount, na.rm=T)
# PLdat$c_stressors_burden <- PLdat$stressors_burden - mean(PLdat$stressors_burden, na.rm=T)
# PLdat$c_mspsss_total <- PLdat$mspss_total - mean(PLdat$mspss_total, na.rm=T)

######################################################################################################
##RQ3: CHANGES FROM T0 TO T2##

PLdat$wave.f = as.factor(PLdat$wave.f)
PLdat$sex = as.factor(PLdat$sex)
dat$wave.f = as.factor(dat$wave.f)
dat$sex = as.factor(dat$sex_ismale)

PLdat = data.frame(PLdat)
dat = data.frame(dat)

#Alpha = .007 (Bonferroni corrected, initial alpha of .05)

H3a <- lme(PL_aloneprop ~ wave.f + c_age + sex, data = PLdat, random = ~1| record_id, na.action = na.omit)
summary(H3a)

## Linear mixed-effects model fit by REML
## Data: PLdat
## AIC BIC logLik
## -213.5545 -189.8663 112.7772
##
## Random effects:
## Formula: ~1 | record_id
## (Intercept) Residual
## StdDev: 0.07484205 0.1598333
##
## Fixed effects: PL_aloneprop ~ wave.f + c_age + sex
## Value Std.Error DF t-value p-value
## (Intercept) 0.20330229 0.015484912 217 13.129057 0.0000
## wave.f2 0.21884912 0.020991593 166 10.425560 0.0000
## c_age 0.02029801 0.005110782 166 3.971606 0.0001
## sex2 -0.01346016 0.023133336 166 -0.581851 0.5615
## Correlation:
## (Intr) wav.f2 c_age
## wave.f2 -0.706
## c_age 0.408 -0.620
## sex2 -0.305 -0.046 0.122
##
## Standardized Within-Group Residuals:
## Min Q1 Med Q3 Max
## -2.30911715 -0.59445538 -0.08986826 0.58883175 2.93216580
##
## Number of Observations: 387
## Number of Groups: 218

H3b <- lme(momentPA ~ wave.f + c_age + sex, data = dat, random = ~1| record_id,
 method = "REML", na.action = na.omit, control = lmeControl(opt='optim'))
summary(H3b)

## Linear mixed-effects model fit by REML
## Data: dat
## AIC BIC logLik
## 10890.09 10926.9 -5439.047
##
## Random effects:
## Formula: ~1 | record_id
## (Intercept) Residual
## StdDev: 0.7861162 1.116981
##
## Fixed effects: momentPA ~ wave.f + c_age + sex
## Value Std.Error DF t-value p-value
## (Intercept) 4.980811 0.07877089 3194 63.23162 0.0000
## wave.fwave2 -0.243612 0.08888669 3194 -2.74070 0.0062
## c_age -0.091922 0.02981013 3194 -3.08359 0.0021
## sex1 0.491425 0.13700053 3194 3.58703 0.0003
## Correlation:
## (Intr) wv.fw2 c_age
## wave.fwave2 -0.516
## c_age 0.363 -0.859
## sex1 -0.350 -0.075 0.112
##
## Standardized Within-Group Residuals:
## Min Q1 Med Q3 Max
## -4.3480533 -0.5328157 0.1054975 0.6249931 2.8843267
##
## Number of Observations: 3415
## Number of Groups: 218

H3c <- lme(momentNA ~ wave.f + c_age + sex, data = dat, random = ~1| record_id,
 method = "REML", na.action = na.omit, control = lmeControl(opt='optim'))
summary(H3c)

## Linear mixed-effects model fit by REML
## Data: dat
## AIC BIC logLik
## 9083.88 9120.689 -4535.94
##
## Random effects:
## Formula: ~1 | record_id
## (Intercept) Residual
## StdDev: 0.7810894 0.8448839
##
## Fixed effects: momentNA ~ wave.f + c_age + sex
## Value Std.Error DF t-value p-value
## (Intercept) 2.1189588 0.07314535 3194 28.969154 0.0000
## wave.fwave2 0.0987620 0.07904276 3194 1.249476 0.2116
## c_age 0.0621871 0.02767275 3194 2.247234 0.0247
## sex1 -0.3921928 0.12623640 3194 -3.106813 0.0019
## Correlation:
## (Intr) wv.fw2 c_age
## wave.fwave2 -0.485
## c_age 0.377 -0.899
## sex1 -0.353 -0.075 0.108
##
## Standardized Within-Group Residuals:
## Min Q1 Med Q3 Max
## -3.3342361 -0.5350955 -0.1405411 0.4204834 5.1435681
##
## Number of Observations: 3415
## Number of Groups: 218

H3d <- lme(esm_soc_alone_pleasant ~ wave.f + c_age + sex, data = dat, random = ~1| record_id,
 method = "REML", na.action = na.omit, control = lmeControl(opt='optim'))
summary(H3d)

## Linear mixed-effects model fit by REML
## Data: dat
## AIC BIC logLik
## 12638.36 12675.17 -6313.182
##
## Random effects:
## Formula: ~1 | record_id
## (Intercept) Residual
## StdDev: 1.226569 1.428293
##
## Fixed effects: esm_soc_alone_pleasant ~ wave.f + c_age + sex
## Value Std.Error DF t-value p-value
## (Intercept) 5.341011 0.11679713 3194 45.72896 0.0000
## wave.fwave2 -0.282295 0.12777738 3194 -2.20927 0.0272
## c_age -0.064389 0.04427585 3194 -1.45427 0.1460
## sex1 -0.355615 0.20233709 3194 -1.75754 0.0789
## Correlation:
## (Intr) wv.fw2 c_age
## wave.fwave2 -0.493
## c_age 0.374 -0.889
## sex1 -0.353 -0.075 0.109
##
## Standardized Within-Group Residuals:
## Min Q1 Med Q3 Max
## -4.0204265 -0.5665574 0.1403708 0.6165215 2.7596090
##
## Number of Observations: 3415
## Number of Groups: 218

H3e <- lme(esm_soc_alone_want ~ wave.f + c_age + sex, data = dat, random = ~1| record_id,
 method = "REML", na.action = na.omit, control = lmeControl(opt='optim'))
summary(H3e)

## Linear mixed-effects model fit by REML
## Data: dat
## AIC BIC logLik
## 13517.08 13553.89 -6752.542
##
## Random effects:
## Formula: ~1 | record_id
## (Intercept) Residual
## StdDev: 1.219633 1.636862
##
## Fixed effects: esm_soc_alone_want ~ wave.f + c_age + sex
## Value Std.Error DF t-value p-value
## (Intercept) 4.832079 0.12031925 3194 40.16048 0.0000
## wave.fwave2 -0.227725 0.13460983 3194 -1.69174 0.0908
## c_age -0.066735 0.04560253 3194 -1.46340 0.1435
## sex1 -0.517472 0.20921634 3194 -2.47338 0.0134
## Correlation:
## (Intr) wv.fw2 c_age
## wave.fwave2 -0.509
## c_age 0.367 -0.868
## sex1 -0.351 -0.075 0.111
##
## Standardized Within-Group Residuals:
## Min Q1 Med Q3 Max
## -3.4509051 -0.5613503 0.1424000 0.6584089 3.1643478
##
## Number of Observations: 3415
## Number of Groups: 218

H3f <- lme(esm_soc_alone_excluded ~ wave.f + c_age + sex, data = dat, random = ~1| record_id,
 method = "REML", na.action = na.omit, control = lmeControl(opt='optim'))
summary(H3f)

## Linear mixed-effects model fit by REML
## Data: dat
## AIC BIC logLik
## 9157.499 9194.303 -4572.75
##
## Random effects:
## Formula: ~1 | record_id
## (Intercept) Residual
## StdDev: 0.4737845 0.878719
##
## Fixed effects: esm_soc_alone_excluded ~ wave.f + c_age + sex
## Value Std.Error DF t-value p-value
## (Intercept) 1.3724234 0.05172069 3191 26.535286 0.0000
## wave.fwave2 0.0403683 0.06062048 3191 0.665919 0.5055
## c_age 0.0256623 0.01924266 3191 1.333617 0.1824
## sex1 -0.1276088 0.08922846 3191 -1.430136 0.1528
## Correlation:
## (Intr) wv.fw2 c_age
## wave.fwave2 -0.551
## c_age 0.342 -0.811
## sex1 -0.341 -0.073 0.115
##
## Standardized Within-Group Residuals:
## Min Q1 Med Q3 Max
## -2.46111393 -0.34106051 -0.13709777 -0.04208912 6.60067546
##
## Number of Observations: 3412
## Number of Groups: 218

H3g <- lme(esm_mood_lonely ~ wave.f + c_age + sex, data = dat, random = ~1| record_id,
 method = "REML", na.action = na.omit, control = lmeControl(opt='optim'))
summary(H3g)

## Linear mixed-effects model fit by REML
## Data: dat
## AIC BIC logLik
## 12349.73 12386.54 -6168.866
##
## Random effects:
## Formula: ~1 | record_id
## (Intercept) Residual
## StdDev: 1.012659 1.380538
##
## Fixed effects: esm_mood_lonely ~ wave.f + c_age + sex
## Value Std.Error DF t-value p-value
## (Intercept) 2.1250232 0.10032103 3194 21.182231 0.0000
## wave.fwave2 0.1119388 0.11250365 3194 0.994980 0.3198
## c_age 0.0318962 0.03801023 3194 0.839147 0.4014
## sex1 -0.2792680 0.17446560 3194 -1.600705 0.1095
## Correlation:
## (Intr) wv.fw2 c_age
## wave.fwave2 -0.511
## c_age 0.366 -0.866
## sex1 -0.351 -0.075 0.111
##
## Standardized Within-Group Residuals:
## Min Q1 Med Q3 Max
## -2.9164678 -0.5167455 -0.2022720 0.2943299 4.1733046
##
## Number of Observations: 3415
## Number of Groups: 218

#Plot significant effects
#Proportion of time spent socially withdrawing
effH3a <- as.data.frame(effect(term="wave.f",mod=H3a))[,c('wave.f','fit','se','lower','upper')]
effH3a$wave.f <- as.factor(effH3a$wave.f)
plot_H3a <- ggplot(effH3a, aes(x = wave.f, y= fit)) +
 geom_bar(stat="summary", fun="mean", position="dodge", width=.5)+
 #geom_signif(comparisons=list(c("1", "2")), map_signif_level = TRUE)+
 labs(title = "Change in Proportion of \n Time Spent \n Socially Withdrawing", x= "Timepoint",
 y="Prop. Time Spent \n Socially Withdrawing") + theme_classic() + theme(axis.title=element_text(size=10)) + theme(plot.title=element_text(size=12)) +
 theme(plot.title=element_text(face="bold")) + theme(plot.title=element_text(hjust=0.5)) + scale_x_discrete(labels=c("T0", "T2"))
plot_H3a


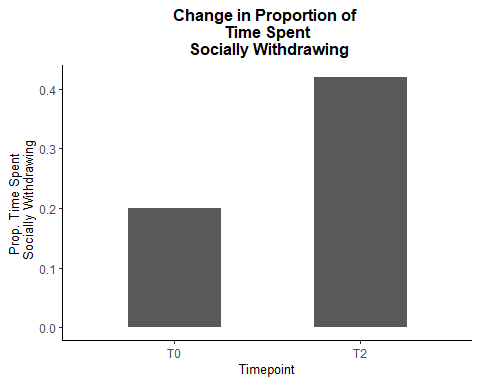


ggsave("H3a.png")

## Saving 5 x 4 in image

#PA
effH3b <- as.data.frame(effect(term="wave.f",mod=H3b))[,c('wave.f','fit','se','lower','upper')]
effH3b$wave.f <- as.factor(effH3b$wave.f)
plot_H3b <- ggplot(effH3b, aes(x = wave.f, y= fit)) +
 geom_bar(stat="summary", fun="mean", position="dodge", width=.5)+
 #geom_signif(comparisons=list(c("1", "2")), map_signif_level=TRUE)+
 labs(title = "Change in Positive Affect", x= "Timepoint",
 y="Positive Affect") + theme_classic() + theme(axis.title=element_text(size=10)) + theme(plot.title=element_text(size=12)) +
 theme(plot.title=element_text(face="bold")) + theme(plot.title=element_text(hjust=0.5)) + scale_x_discrete(labels=c("T0", "T2"))
plot_H3b


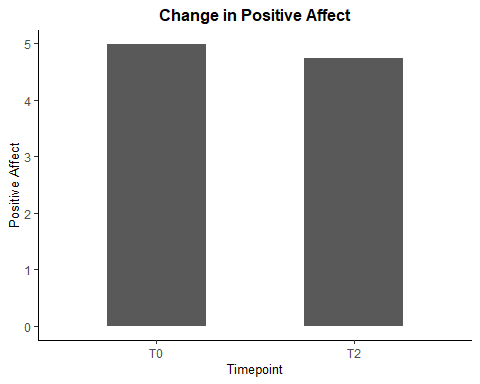


ggsave("H3b.png")

## Saving 5 x 4 in image

######################################################################################################
##PREPARE DATASETS FOR MODERATOR ANALYSES##
#For the moderators that were only measured at T2, we need to use difference scores as outcome variables in order to make inferences
#about the change from T0 to T2

#Create a difference score variable for aloneprop (delta_aloneprop = aloneprop[T2] - aloneprop[T0])
PLdat <- PLdat %>%
 group_by(record_id) %>%
 mutate(delta_aloneprop = ifelse(length(which(wave == 'wave1'))>0, (diff(PL_aloneprop)*-1)/PL_aloneprop[T0], NA)) %>%
 ungroup()

#Create "difference scores" for the beep-level outcomes
#We calculate the difference at T1 compared to the person-level mean at T0
#Create columns with T0 means
PLdat <- PLdat %>%
 group_by(record_id) %>%
 mutate(momentPA_wave1 = ifelse(length(which(wave == 'wave1'))>0, PL_momentPA[T0], NA),
 momentNA_wave1 = ifelse(length(which(wave == 'wave1'))>0, PL_momentNA[T0], NA),
 pleasant_wave1 = ifelse(length(which(wave == 'wave1'))>0, PL_pleasant[T0], NA),
 excluded_wave1 = ifelse(length(which(wave == 'wave1'))>0, PL_excluded[T0], NA),
 want_wave1 = ifelse(length(which(wave == 'wave1'))>0, PL_want[T0], NA),
 loneliness_wave1 = ifelse(length(which(wave == 'wave1'))>0, PL_loneliness[T0], NA)) %>%
 ungroup()

#Append columns from PLdat to dat
# dat$groupMean = as.integer(dat$groupMean)
# PLdat$groupMean = as.integer(PLdat$groupMean)
mergedat = subset(PLdat, select = c("record_id", "wave", "stressors_amount", "stressors_burden", "groupMean", "delta_aloneprop", "momentPA_wave1",
 "momentNA_wave1", "pleasant_wave1", "excluded_wave1", "want_wave1", "loneliness_wave1"))
dat = left_join(x = dat, y = mergedat)

## Joining, by = c("record_id", "wave")

#Create momentary "difference scores"
dat <- dat %>%
 group_by(record_id) %>%
 mutate(delta_PA = ifelse(wave == 'wave2', (momentPA - momentPA_wave1)/momentPA_wave1, NA),
 delta_NA = ifelse(wave == 'wave2', (momentNA - momentNA_wave1)/momentNA_wave1, NA),
 delta_pleasant = ifelse(wave == 'wave2', (esm_soc_alone_pleasant - pleasant_wave1)/pleasant_wave1, NA),
 delta_excluded = ifelse(wave == 'wave2', (esm_soc_alone_excluded - excluded_wave1)/excluded_wave1, NA),
 delta_want = ifelse(wave == 'wave2', (esm_soc_alone_want - want_wave1)/want_wave1, NA),
 delta_loneliness = ifelse(wave == 'wave2', (esm_mood_lonely - loneliness_wave1)/loneliness_wave1, NA)) %>%
 ungroup()

######################################################################################################
##RQ4 PART 1: MODERATORS IN CHANGES FROM T0 TO T2 (NON-IMPUTED MODERATORS)##

#Alpha per moderator = .007 (Bonferroni corrected, initial alpha of .05)

PLdat$groupMean = as.factor(PLdat$groupMean)
PLdat$wave.f = as.factor(PLdat$wave.f)
PLdat$sex = as.factor(PLdat$sex)
dat$groupMean = as.factor(dat$groupMean)
dat$wave.f = as.factor(dat$wave.f)
dat$sex = as.factor(dat$sex_ismale)

PLcovidDat <- subset(PLdat[PLdat$wave == 'wave2',], select = c("record_id", "stressors_amount", "stressors_burden", "groupMean", "delta_aloneprop",
 "PL_aloneprop", "c_age", "sex"))
ESMcovidDat <- subset(dat[dat$wave == 'wave2',], select = c("record_id", "stressors_amount", "stressors_burden", "groupMean", "delta_PA", "delta_NA",
 "delta_pleasant", "delta_excluded", "delta_want", "delta_loneliness", "momentPA", "momentNA",
 "esm_soc_alone_pleasant", "esm_soc_alone_excluded", "esm_soc_alone_want", "esm_mood_lonely", "c_age", "sex"))

PLcovidDat = as.data.frame(PLcovidDat)
ESMcovidDat = as.data.frame(ESMcovidDat)

PLcovidDat$groupMean = as.factor(PLcovidDat$groupMean)
PLcovidDat$sex = as.factor(PLcovidDat$sex)
ESMcovidDat$groupMean = as.factor(ESMcovidDat$groupMean)
ESMcovidDat$sex = as.factor(ESMcovidDat$sex)

#Amount of stressors - this data cannot be imputed due to the conditional nature of the questionnaire
H4a_amount <- lm(delta_aloneprop ~ stressors_amount + c_age + sex, data = PLcovidDat, na.action = na.omit)
summary(H4a_amount)

##
## Call:
## lm(formula = delta_aloneprop ~ stressors_amount + c_age + sex,
## data = PLcovidDat, na.action = na.omit)
##
## Residuals:
## Min 1Q Median 3Q Max
## -23.6158 -0.9719 1.1436 2.7416 5.5857
##
## Coefficients:
## Estimate Std. Error t value Pr(>|t|)
## (Intercept) -5.54758 1.36137 -4.075 7.27e-05 ***
## stressors_amount 0.09727 0.11502 0.846 0.399003
## c_age 0.66235 0.18775 3.528 0.000549 ***
## sex2 0.45205 0.91029 0.497 0.620163
## ---
## Signif. codes: 0 '***' 0.001 '**' 0.01 '*' 0.05 '.' 0.1 ' ' 1
##
## Residual standard error: 4.592 on 158 degrees of freedom
## (42 observations deleted due to missingness)
## Multiple R-squared: 0.07716, Adjusted R-squared: 0.05964
## F-statistic: 4.404 on 3 and 158 DF, p-value: 0.005266

H4b_amount <- lme(delta_PA ~ stressors_amount + c_age + sex, data = ESMcovidDat, random = ~1| record_id,
 method = "REML", na.action = na.omit, control = lmeControl(opt='optim'))
summary(H4b_amount)

## Linear mixed-effects model fit by REML
## Data: ESMcovidDat
## AIC BIC logLik
## 3.356813 36.77019 4.321593
##
## Random effects:
## Formula: ~1 | record_id
## (Intercept) Residual
## StdDev: 0.2831183 0.213481
##
## Fixed effects: delta_PA ~ stressors_amount + c_age + sex
## Value Std.Error DF t-value p-value
## (Intercept) -0.09892293 0.08870929 1779 -1.1151360 0.2649
## stressors_amount -0.00271608 0.00745230 158 -0.3644617 0.7160
## c_age 0.01690601 0.01208375 158 1.3990696 0.1638
## sex1 0.09634335 0.05880086 158 1.6384685 0.1033
## Correlation:
## (Intr) strss_ c_age
## stressors_amount -0.926
## c_age -0.214 -0.026
## sex1 -0.285 0.145 0.085
##
## Standardized Within-Group Residuals:
## Min Q1 Med Q3 Max
## -4.30360659 -0.48719447 0.05861801 0.53266568 7.36169738
##
## Number of Observations: 1941
## Number of Groups: 162

H4c_amount <- lme(delta_NA ~ stressors_amount + c_age + sex, data = ESMcovidDat, random = ~1| record_id,
 method = "REML", na.action = na.omit, control = lmeControl(opt='optim'))
summary(H4c_amount)

## Linear mixed-effects model fit by REML
## Data: ESMcovidDat
## AIC BIC logLik
## 2965.834 2999.247 -1476.917
##
## Random effects:
## Formula: ~1 | record_id
## (Intercept) Residual
## StdDev: 0.5179547 0.4645369
##
## Fixed effects: delta_NA ~ stressors_amount + c_age + sex
## Value Std.Error DF t-value p-value
## (Intercept) 0.21058932 0.16499584 1779 1.276331 0.2020
## stressors_amount 0.02140288 0.01386627 158 1.543521 0.1247
## c_age -0.05676129 0.02244000 158 -2.529469 0.0124
## sex1 -0.17152608 0.10926118 158 -1.569872 0.1184
## Correlation:
## (Intr) strss_ c_age
## stressors_amount -0.926
## c_age -0.210 -0.031
## sex1 -0.282 0.142 0.086
##
## Standardized Within-Group Residuals:
## Min Q1 Med Q3 Max
## -3.7953019 -0.5407338 -0.1344720 0.3631308 6.4962713
##
## Number of Observations: 1941
## Number of Groups: 162

H4d_amount <- lme(delta_pleasant ~ stressors_amount + c_age + sex, data = ESMcovidDat, random = ~1| record_id,
 method = "REML", na.action = na.omit, control = lmeControl(opt='optim'))
summary(H4d_amount)

## Linear mixed-effects model fit by REML
## Data: ESMcovidDat
## AIC BIC logLik
## 2116.76 2150.174 -1052.38
##
## Random effects:
## Formula: ~1 | record_id
## (Intercept) Residual
## StdDev: 0.6473815 0.3599011
##
## Fixed effects: delta_pleasant ~ stressors_amount + c_age + sex
## Value Std.Error DF t-value p-value
## (Intercept) 0.24151497 0.19880405 1779 1.2148393 0.2246
## stressors_amount -0.00397795 0.01669101 158 -0.2383289 0.8119
## c_age -0.06807967 0.02713428 158 -2.5089907 0.0131
## sex1 0.05566967 0.13189269 158 0.4220831 0.6735
## Correlation:
## (Intr) strss_ c_age
## stressors_amount -0.926
## c_age -0.220 -0.019
## sex1 -0.289 0.149 0.084
##
## Standardized Within-Group Residuals:
## Min Q1 Med Q3 Max
## -8.26231569 -0.30207091 0.04088288 0.37603400 6.69452105
##
## Number of Observations: 1941
## Number of Groups: 162

H4e_amount <- lme(delta_want ~ stressors_amount + c_age + sex, data = ESMcovidDat, random = ~1| record_id,
 method = "REML", na.action = na.omit, control = lmeControl(opt='optim'))
summary(H4e_amount)

## Linear mixed-effects model fit by REML
## Data: ESMcovidDat
## AIC BIC logLik
## 3094.155 3127.569 -1541.078
##
## Random effects:
## Formula: ~1 | record_id
## (Intercept) Residual
## StdDev: 0.8863079 0.460774
##
## Fixed effects: delta_want ~ stressors_amount + c_age + sex
## Value Std.Error DF t-value p-value
## (Intercept) 0.12812668 0.27129921 1779 0.4722708 0.6368
## stressors_amount 0.01861977 0.02277490 158 0.8175567 0.4148
## c_age -0.11160317 0.03704107 158 -3.0129570 0.0030
## sex1 0.23945640 0.18000653 158 1.3302650 0.1853
## Correlation:
## (Intr) strss_ c_age
## stressors_amount -0.926
## c_age -0.221 -0.018
## sex1 -0.289 0.150 0.084
##
## Standardized Within-Group Residuals:
## Min Q1 Med Q3 Max
## -8.41306448 -0.32142110 0.02343293 0.36052495 10.10599008
##
## Number of Observations: 1941
## Number of Groups: 162

H4f_amount <- lme(delta_excluded ~ stressors_amount + c_age + sex, data = ESMcovidDat, random = ~1| record_id,
 method = "REML", na.action = na.omit, control = lmeControl(opt='optim'))
summary(H4f_amount)

## Linear mixed-effects model fit by REML
## Data: ESMcovidDat
## AIC BIC logLik
## 4223.422 4256.829 -2105.711
##
## Random effects:
## Formula: ~1 | record_id
## (Intercept) Residual
## StdDev: 0.5085895 0.6600999
##
## Fixed effects: delta_excluded ~ stressors_amount + c_age + sex
## Value Std.Error DF t-value p-value
## (Intercept) 0.07313330 0.17077239 1777 0.4282501 0.6685
## stressors_amount 0.01276553 0.01435995 158 0.8889676 0.3754
## c_age -0.00593165 0.02311433 158 -0.2566221 0.7978
## sex1 -0.16663456 0.11262285 158 -1.4795804 0.1410
## Correlation:
## (Intr) strss_ c_age
## stressors_amount -0.926
## c_age -0.202 -0.043
## sex1 -0.276 0.135 0.087
##
## Standardized Within-Group Residuals:
## Min Q1 Med Q3 Max
## -3.66068548 -0.33234190 -0.06974750 -0.01334476 8.69364735
##
## Number of Observations: 1939
## Number of Groups: 162

H4g_amount <- lme(delta_loneliness ~ stressors_amount + c_age + sex, data = ESMcovidDat, random = ~1| record_id,
 method = "REML", na.action = na.omit, control = lmeControl(opt='optim'))
summary(H4g_amount)

## Linear mixed-effects model fit by REML
## Data: ESMcovidDat
## AIC BIC logLik
## 5608.532 5641.946 -2798.266
##
## Random effects:
## Formula: ~1 | record_id
## (Intercept) Residual
## StdDev: 1.102008 0.9136615
##
## Fixed effects: delta_loneliness ~ stressors_amount + c_age + sex
## Value Std.Error DF t-value p-value
## (Intercept) -0.3421475 0.3482415 1779 -0.9825005 0.3260
## stressors_amount 0.0908965 0.0292612 158 3.1063837 0.0022
## c_age -0.0386525 0.0473980 158 -0.8154879 0.4160
## sex1 -0.4108244 0.2307217 158 -1.7806058 0.0769
## Correlation:
## (Intr) strss_ c_age
## stressors_amount -0.926
## c_age -0.212 -0.028
## sex1 -0.283 0.143 0.085
##
## Standardized Within-Group Residuals:
## Min Q1 Med Q3 Max
## -3.92428822 -0.41666926 -0.09162255 0.17741544 6.12607106
##
## Number of Observations: 1941
## Number of Groups: 162

#Plot significant effects
#Loneliness
effH4g_amount <- as.data.frame(effect(term="stressors_amount",mod=H4g_amount))[,c('stressors_amount','fit','se','lower','upper')]
plot_H4g_amount <- ggplot(effH4g_amount, aes(x=stressors_amount, y=fit)) +
 geom_point() +
 geom_line(size=1.2) +
 geom_ribbon(aes(ymin=lower, ymax=upper),alpha=0.3) +
 labs(title = "Relative Change in Loneliness \n by Amount of Stressors", x= "Amount of COVID-related stressors", y="Change in Loneliness (%)")+
 theme_classic() + theme(axis.title=element_text(size=10)) + theme(plot.title=element_text(size=12)) +
 theme(plot.title=element_text(face="bold")) + theme(plot.title=element_text(hjust=0.5))
plot_H4g_amount


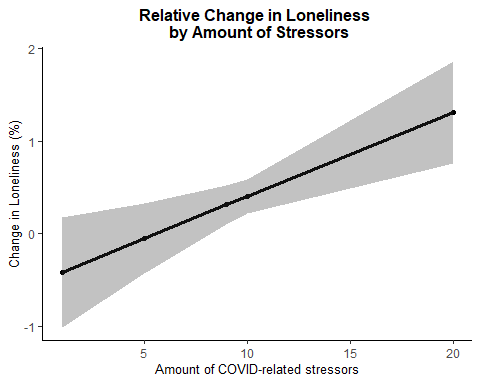


ggsave("H4g_amount.png")

## Saving 5 x 4 in image

#Mean burdensomeness of stressors - this data cannot be imputed due to the conditional nature of the questionnaire
H4a_burden <- lm(delta_aloneprop ~ stressors_burden + c_age + sex, data = PLcovidDat, na.action = na.omit)
summary(H4a_burden)

##
## Call:
## lm(formula = delta_aloneprop ~ stressors_burden + c_age + sex,
## data = PLcovidDat, na.action = na.omit)
##
## Residuals:
## Min 1Q Median 3Q Max
## -23.550 -1.095 1.274 2.795 5.367
##
## Coefficients:
## Estimate Std. Error t value Pr(>|t|)
## (Intercept) -5.1385 2.2810 -2.253 0.02566 *
## stressors_burden 0.2063 0.6910 0.299 0.76564
## c_age 0.6457 0.1973 3.272 0.00131 **
## sex2 0.3840 0.9178 0.418 0.67622
## ---
## Signif. codes: 0 '***' 0.001 '**' 0.01 '*' 0.05 '.' 0.1 ' ' 1
##
## Residual standard error: 4.602 on 158 degrees of freedom
## (42 observations deleted due to missingness)
## Multiple R-squared: 0.07351, Adjusted R-squared: 0.05591
## F-statistic: 4.178 on 3 and 158 DF, p-value: 0.007046

H4b_burden <- lme(delta_PA ~ stressors_burden + c_age + sex, data = ESMcovidDat, random = ~1| record_id,
 method = "REML", na.action = na.omit, control = lmeControl(opt='optim'))
summary(H4b_burden)

## Linear mixed-effects model fit by REML
## Data: ESMcovidDat
## AIC BIC logLik
## -0.3878285 33.02555 6.193914
##
## Random effects:
## Formula: ~1 | record_id
## (Intercept) Residual
## StdDev: 0.2830291 0.2134771
##
## Fixed effects: delta_PA ~ stressors_burden + c_age + sex
## Value Std.Error DF t-value p-value
## (Intercept) -0.05192169 0.14694962 1779 -0.3533299 0.7239
## stressors_burden -0.02409562 0.04481090 158 -0.5377177 0.5915
## c_age 0.01888752 0.01268855 158 1.4885484 0.1386
## sex1 0.09341499 0.05923383 158 1.5770546 0.1168
## Correlation:
## (Intr) strss_ c_age
## stressors_burden -0.974
## c_age 0.162 -0.307
## sex1 -0.274 0.189 0.026
##
## Standardized Within-Group Residuals:
## Min Q1 Med Q3 Max
## -4.31004241 -0.48791224 0.06043407 0.53474506 7.37136686
##
## Number of Observations: 1941
## Number of Groups: 162

H4c_burden <- lme(delta_NA ~ stressors_burden + c_age + sex, data = ESMcovidDat, random = ~1| record_id,
 method = "REML", na.action = na.omit, control = lmeControl(opt='optim'))
summary(H4c_burden)

## Linear mixed-effects model fit by REML
## Data: ESMcovidDat
## AIC BIC logLik
## 2961.203 2994.617 -1474.602
##
## Random effects:
## Formula: ~1 | record_id
## (Intercept) Residual
## StdDev: 0.5170066 0.4644689
##
## Fixed effects: delta_NA ~ stressors_burden + c_age + sex
## Value Std.Error DF t-value p-value
## (Intercept) -0.04604268 0.27294996 1779 -0.1686854 0.8661
## stressors_burden 0.15434602 0.08330323 158 1.8528216 0.0658
## c_age -0.06917178 0.02354186 158 -2.9382467 0.0038
## sex1 -0.15698490 0.10995426 158 -1.4277291 0.1553
## Correlation:
## (Intr) strss_ c_age
## stressors_burden -0.974
## c_age 0.163 -0.309
## sex1 -0.274 0.189 0.027
##
## Standardized Within-Group Residuals:
## Min Q1 Med Q3 Max
## -3.7894182 -0.5431063 -0.1353560 0.3646756 6.4934716
##
## Number of Observations: 1941
## Number of Groups: 162

H4d_burden <- lme(delta_pleasant ~ stressors_burden + c_age + sex, data = ESMcovidDat, random = ~1| record_id,
 method = "REML", na.action = na.omit, control = lmeControl(opt='optim'))
summary(H4d_burden)

## Linear mixed-effects model fit by REML
## Data: ESMcovidDat
## AIC BIC logLik
## 2113.209 2146.622 -1050.604
##
## Random effects:
## Formula: ~1 | record_id
## (Intercept) Residual
## StdDev: 0.6474704 0.3599005
##
## Fixed effects: delta_pleasant ~ stressors_burden + c_age + sex
## Value Std.Error DF t-value p-value
## (Intercept) 0.24475201 0.3293250 1779 0.7431929 0.4575
## stressors_burden -0.01473828 0.1003123 158 -0.1469239 0.8834
## c_age -0.06692584 0.0284880 158 -2.3492628 0.0200
## sex1 0.05665062 0.1328540 158 0.4264125 0.6704
## Correlation:
## (Intr) strss_ c_age
## stressors_burden -0.974
## c_age 0.160 -0.305
## sex1 -0.275 0.190 0.024
##
## Standardized Within-Group Residuals:
## Min Q1 Med Q3 Max
## -8.26286116 -0.30345361 0.04045965 0.37552663 6.69217907
##
## Number of Observations: 1941
## Number of Groups: 162

H4e_burden <- lme(delta_want ~ stressors_burden + c_age + sex, data = ESMcovidDat, random = ~1| record_id,
 method = "REML", na.action = na.omit, control = lmeControl(opt='optim'))
summary(H4e_burden)

## Linear mixed-effects model fit by REML
## Data: ESMcovidDat
## AIC BIC logLik
## 3090.672 3124.085 -1539.336
##
## Random effects:
## Formula: ~1 | record_id
## (Intercept) Residual
## StdDev: 0.8867465 0.4607679
##
## Fixed effects: delta_want ~ stressors_burden + c_age + sex
## Value Std.Error DF t-value p-value
## (Intercept) 0.6625387 0.4495158 1779 1.4738944 0.1407
## stressors_burden -0.1029080 0.1368963 158 -0.7517225 0.4533
## c_age -0.1021697 0.0388988 158 -2.6265505 0.0095
## sex1 0.1914440 0.1813646 158 1.0555752 0.2928
## Correlation:
## (Intr) strss_ c_age
## stressors_burden -0.974
## c_age 0.160 -0.304
## sex1 -0.275 0.190 0.024
##
## Standardized Within-Group Residuals:
## Min Q1 Med Q3 Max
## -8.41229041 -0.31807772 0.02372243 0.36148983 10.11277830
##
## Number of Observations: 1941
## Number of Groups: 162

H4f_burden <- lme(delta_excluded ~ stressors_burden + c_age + sex, data = ESMcovidDat, random = ~1| record_id,
 method = "REML", na.action = na.omit, control = lmeControl(opt='optim'))
summary(H4f_burden)

## Linear mixed-effects model fit by REML
## Data: ESMcovidDat
## AIC BIC logLik
## 4220.598 4254.005 -2104.299
##
## Random effects:
## Formula: ~1 | record_id
## (Intercept) Residual
## StdDev: 0.5109352 0.6600255
##
## Fixed effects: delta_excluded ~ stressors_burden + c_age + sex
## Value Std.Error DF t-value p-value
## (Intercept) 0.25252493 0.28360930 1777 0.8903972 0.3734
## stressors_burden -0.01224431 0.08674273 158 -0.1411567 0.8879
## c_age -0.00388235 0.02441666 158 -0.1590041 0.8739
## sex1 -0.18306464 0.11405980 158 -1.6049883 0.1105
## Correlation:
## (Intr) strss_ c_age
## stressors_burden -0.973
## c_age 0.167 -0.314
## sex1 -0.274 0.188 0.029
##
## Standardized Within-Group Residuals:
## Min Q1 Med Q3 Max
## -3.66726972 -0.33417269 -0.07324701 -0.01293558 8.68621841
##
## Number of Observations: 1939
## Number of Groups: 162

H4g_burden <- lme(delta_loneliness ~ stressors_burden + c_age + sex, data = ESMcovidDat, random = ~1| record_id,
 method = "REML", na.action = na.omit, control = lmeControl(opt='optim'))
summary(H4g_burden)

## Linear mixed-effects model fit by REML
## Data: ESMcovidDat
## AIC BIC logLik
## 5613.211 5646.624 -2800.606
##
## Random effects:
## Formula: ~1 | record_id
## (Intercept) Residual
## StdDev: 1.136815 0.9135022
##
## Fixed effects: delta_loneliness ~ stressors_burden + c_age + sex
## Value Std.Error DF t-value p-value
## (Intercept) 0.0644127 0.5935370 1779 0.1085235 0.9136
## stressors_burden 0.1862719 0.1810454 158 1.0288684 0.3051
## c_age -0.0501692 0.0512289 158 -0.9793147 0.3289
## sex1 -0.4672044 0.2391976 158 -1.9532147 0.0526
## Correlation:
## (Intr) strss_ c_age
## stressors_burden -0.974
## c_age 0.163 -0.307
## sex1 -0.274 0.189 0.026
##
## Standardized Within-Group Residuals:
## Min Q1 Med Q3 Max
## -3.92305206 -0.40584809 -0.08741509 0.16593181 6.12917890
##
## Number of Observations: 1941
## Number of Groups: 162

#Wave 1 cluster membership - this data cannot be imputed because it is categorical
H4a_cluster <- lm(PL_aloneprop ~ groupMean + c_age + sex, data = PLcovidDat, na.action = na.omit)
summary(H4a_cluster)

##
## Call:
## lm(formula = PL_aloneprop ~ groupMean + c_age + sex, data = PLcovidDat,
## na.action = na.omit)
##
## Residuals:
## Min 1Q Median 3Q Max
## -0.43697 -0.14916 -0.01202 0.13155 0.50410
##
## Coefficients:
## Estimate Std. Error t value Pr(>|t|)
## (Intercept) 0.445406 0.023901 18.635 <2e-16 ***
## groupMean1 -0.028623 0.039042 -0.733 0.4645
## c_age 0.017823 0.008542 2.087 0.0385 *
## sex2 0.019590 0.040658 0.482 0.6306
## ---
## Signif. codes: 0 '***' 0.001 '**' 0.01 '*' 0.05 '.' 0.1 ' ' 1
##
## Residual standard error: 0.2139 on 166 degrees of freedom
## (34 observations deleted due to missingness)
## Multiple R-squared: 0.02735, Adjusted R-squared: 0.009771
## F-statistic: 1.556 on 3 and 166 DF, p-value: 0.2021

H4b_cluster <- lme(momentPA ~ groupMean + c_age + sex, data = ESMcovidDat,
 random = ~1| record_id, method = "REML", na.action = na.omit, control = lmeControl(opt='optim'))
summary(H4b_cluster)

## Linear mixed-effects model fit by REML
## Data: ESMcovidDat
## AIC BIC logLik
## 6143.221 6176.854 -3065.611
##
## Random effects:
## Formula: ~1 | record_id
## (Intercept) Residual
## StdDev: 0.8387457 1.018859
##
## Fixed effects: momentPA ~ groupMean + c_age + sex
## Value Std.Error DF t-value p-value
## (Intercept) 4.717026 0.10886401 1843 43.32952 0.0000
## groupMean1 -0.539261 0.16972298 166 -3.17730 0.0018
## c_age -0.048918 0.03679034 166 -1.32965 0.1855
## sex1 0.529092 0.17719229 166 2.98598 0.0033
## Correlation:
## (Intr) grpMn1 c_age
## groupMean1 -0.331
## c_age -0.540 -0.147
## sex1 -0.441 0.120 0.102
##
## Standardized Within-Group Residuals:
## Min Q1 Med Q3 Max
## -4.7023350 -0.5347095 0.1104040 0.6011491 2.9859526
##
## Number of Observations: 2013
## Number of Groups: 170

H4c_cluster <- lme(momentNA ~ groupMean + c_age + sex, data = ESMcovidDat,
 random = ~1| record_id, method = "REML", na.action = na.omit, control = lmeControl(opt='optim'))
summary(H4c_cluster)

## Linear mixed-effects model fit by REML
## Data: ESMcovidDat
## AIC BIC logLik
## 5048.156 5081.789 -2518.078
##
## Random effects:
## Formula: ~1 | record_id
## (Intercept) Residual
## StdDev: 0.8322321 0.7603375
##
## Fixed effects: momentNA ~ groupMean + c_age + sex
## Value Std.Error DF t-value p-value
## (Intercept) 2.2465888 0.10376512 1843 21.650712 0.0000
## groupMean1 0.4462970 0.16203330 166 2.754354 0.0065
## c_age 0.0039363 0.03524996 166 0.111667 0.9112
## sex1 -0.4476532 0.16923543 166 -2.645150 0.0089
## Correlation:
## (Intr) grpMn1 c_age
## groupMean1 -0.334
## c_age -0.536 -0.144
## sex1 -0.440 0.116 0.101
##
## Standardized Within-Group Residuals:
## Min Q1 Med Q3 Max
## -3.5346250 -0.5454911 -0.1413831 0.4158867 5.9560952
##
## Number of Observations: 2013
## Number of Groups: 170

H4d_cluster <- lme(esm_soc_alone_pleasant ~ groupMean + c_age + sex, data = ESMcovidDat,
 random = ~1| record_id, method = "REML", na.action = na.omit, control = lmeControl(opt='optim'))
summary(H4d_cluster)

## Linear mixed-effects model fit by REML
## Data: ESMcovidDat
## AIC BIC logLik
## 7167.174 7200.806 -3577.587
##
## Random effects:
## Formula: ~1 | record_id
## (Intercept) Residual
## StdDev: 1.280257 1.298127
##
## Fixed effects: esm_soc_alone_pleasant ~ groupMean + c_age + sex
## Value Std.Error DF t-value p-value
## (Intercept) 5.129881 0.16168307 1843 31.72800 0.0000
## groupMean1 -0.452880 0.25235755 166 -1.79460 0.0745
## c_age -0.025257 0.05483678 166 -0.46058 0.6457
## sex1 -0.159767 0.26356416 166 -0.60618 0.5452
## Correlation:
## (Intr) grpMn1 c_age
## groupMean1 -0.333
## c_age -0.537 -0.145
## sex1 -0.441 0.118 0.101
##
## Standardized Within-Group Residuals:
## Min Q1 Med Q3 Max
## -4.1389150 -0.4640887 0.1362408 0.5742874 2.9248137
##
## Number of Observations: 2013
## Number of Groups: 170

H4e_cluster <- lme(esm_soc_alone_want ~ groupMean + c_age + sex, data = ESMcovidDat,
 random = ~1| record_id, method = "REML", na.action = na.omit, control = lmeControl(opt='optim'))
summary(H4e_cluster)

## Linear mixed-effects model fit by REML
## Data: ESMcovidDat
## AIC BIC logLik
## 7575.907 7609.54 -3781.954
##
## Random effects:
## Formula: ~1 | record_id
## (Intercept) Residual
## StdDev: 1.339647 1.443326
##
## Fixed effects: esm_soc_alone_want ~ groupMean + c_age + sex
## Value Std.Error DF t-value p-value
## (Intercept) 4.699230 0.17061354 1843 27.543126 0.0000
## groupMean1 -0.212507 0.26620803 166 -0.798273 0.4259
## c_age -0.073570 0.05780281 166 -1.272773 0.2049
## sex1 -0.259790 0.27800727 166 -0.934472 0.3514
## Correlation:
## (Intr) grpMn1 c_age
## groupMean1 -0.332
## c_age -0.538 -0.146
## sex1 -0.441 0.118 0.102
##
## Standardized Within-Group Residuals:
## Min Q1 Med Q3 Max
## -3.74702564 -0.54977023 0.07269534 0.55532949 3.00027907
##
## Number of Observations: 2013
## Number of Groups: 170

H4f_cluster <- lme(esm_soc_alone_excluded ~ groupMean + c_age + sex, data = ESMcovidDat,
 random = ~1| record_id, method = "REML", na.action = na.omit, control = lmeControl(opt='optim'))
summary(H4f_cluster)

## Linear mixed-effects model fit by REML
## Data: ESMcovidDat
## AIC BIC logLik
## 5163.777 5197.404 -2575.889
##
## Random effects:
## Formula: ~1 | record_id
## (Intercept) Residual
## StdDev: 0.6245427 0.8024653
##
## Fixed effects: esm_soc_alone_excluded ~ groupMean + c_age + sex
## Value Std.Error DF t-value p-value
## (Intercept) 1.4025704 0.08189980 1841 17.125444 0.0000
## groupMean1 0.3269081 0.12759360 166 2.562104 0.0113
## c_age -0.0121020 0.02763709 166 -0.437891 0.6620
## sex1 -0.1475208 0.13318473 166 -1.107641 0.2696
## Correlation:
## (Intr) grpMn1 c_age
## groupMean1 -0.331
## c_age -0.541 -0.147
## sex1 -0.442 0.122 0.103
##
## Standardized Within-Group Residuals:
## Min Q1 Med Q3 Max
## -3.08375993 -0.34906362 -0.09864984 -0.02046777 7.12789564
##
## Number of Observations: 2011
## Number of Groups: 170

H4g_cluster <- lme(esm_mood_lonely ~ groupMean + c_age + sex, data = ESMcovidDat,
 random = ~1| record_id, method = "REML", na.action = na.omit, control = lmeControl(opt='optim'))
summary(H4g_cluster)

## Linear mixed-effects model fit by REML
## Data: ESMcovidDat
## AIC BIC logLik
## 6952.045 6985.678 -3470.023
##
## Random effects:
## Formula: ~1 | record_id
## (Intercept) Residual
## StdDev: 1.188208 1.232442
##
## Fixed effects: esm_mood_lonely ~ groupMean + c_age + sex
## Value Std.Error DF t-value p-value
## (Intercept) 2.1556114 0.15051818 1843 14.321270 0.0000
## groupMean1 0.4199996 0.23490340 166 1.787968 0.0756
## c_age 0.0304335 0.05103002 166 0.596384 0.5517
## sex1 -0.4069931 0.24532881 166 -1.658970 0.0990
## Correlation:
## (Intr) grpMn1 c_age
## groupMean1 -0.333
## c_age -0.538 -0.145
## sex1 -0.441 0.118 0.101
##
## Standardized Within-Group Residuals:
## Min Q1 Med Q3 Max
## -3.1001611 -0.4932524 -0.1382761 0.3297309 4.4945495
##
## Number of Observations: 2013
## Number of Groups: 170

#Plot significant effects
#PA
effH4b_cluster <- as.data.frame(effect(term="groupMean",mod=H4b_cluster))[,c('groupMean','fit','se','lower','upper')]
effH4b_cluster$groupMean <- as.factor(effH4b_cluster$groupMean)
plot_H4b_cluster <- ggplot(data=effH4b_cluster, aes(x = groupMean, y= fit)) +
 geom_bar(stat="summary", fun="mean", position="dodge", width=.5)+
 #geom_signif(comparisons=list(c("0", "1")), map_signif_level=TRUE)+
 labs(title = "T2 Positive Affect \n by T0 Cluster Membership", x= "T0 Social Withdrawal Cluster",
 y="T2 Positive Affect") + theme_classic() + theme(axis.title=element_text(size=10)) + theme(plot.title=element_text(size=12)) +
 theme(plot.title=element_text(face="bold")) + theme(plot.title=element_text(hjust=0.5)) + scale_x_discrete(labels=c("Positive", "Negative"))
plot_H4b_cluster


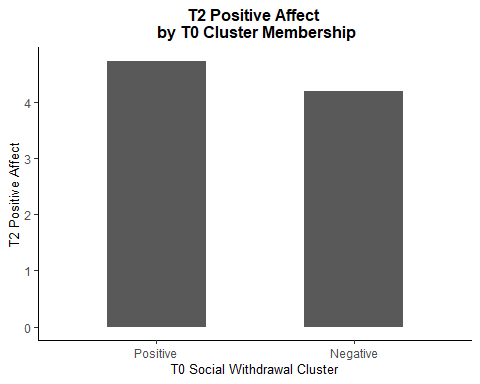


ggsave("H4b_cluster.png")

## Saving 5 x 4 in image

#NA
effH4c_cluster <- as.data.frame(effect(term="groupMean",mod=H4c_cluster))[,c('groupMean','fit','se','lower','upper')]
effH4c_cluster$groupMean <- as.factor(effH4c_cluster$groupMean)
plot_H4c_cluster <- ggplot(data=effH4c_cluster, aes(x = groupMean, y= fit)) +
 geom_bar(stat="summary", fun="mean", position="dodge", width=.5)+
 #geom_signif(comparisons=list(c("0", "1")), map_signif_level=TRUE)+
 labs(title = "T2 Negative Affect \n by T0 Cluster Membership", x= "T0 Social Withdrawal Cluster",
 y="T2 Negative Affect") + theme_classic() + theme(axis.title=element_text(size=10)) + theme(plot.title=element_text(size=12)) +
 theme(plot.title=element_text(face="bold")) + theme(plot.title=element_text(hjust=0.5)) + scale_x_discrete(labels=c("Positive", "Negative"))
plot_H4c_cluster


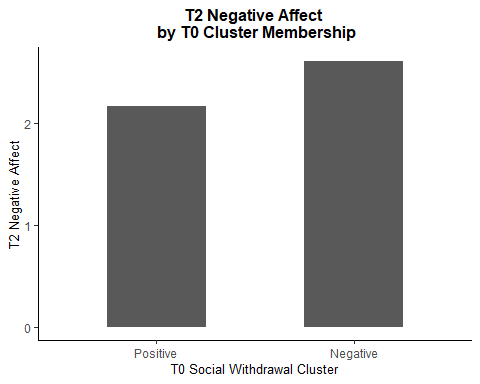


ggsave("H4c_cluster.png")

## Saving 5 x 4 in image

######################################################################################################
##MULTIPLE IMPUTATION OF SSL, VPV AND MSPSS DATA##

#Imputing the SSL and VPV
PLdat_Wave_I = PLdat[PLdat$wave=='wave1',]

data.ESM.aggregated = PLdat_Wave_I[,c("record_id", "ssl_invite", "ssl_visit", "ssl_affection", "ssl_comfort", "ssl_compliment", "ssl_interest",
 "ssl_offerhelp", "ssl_ease", "ssl_advice", "ssl_confidence", "ssl_askhelp", "ssl_positivecharacter",
 "vpv_meetfriends", "vpv_recogniseemotion", "vpv_class", "vpv_help", "vpv_easycontact", "vpv_hurt", "vpv_questions",
 "vpv_sensitivities", "vpv_standup", "vpv_said", "vpv_seekcontact", "vpv_wishes", "vpv_getalong", "vpv_interest",
 "vpv_talkconcerns", "vpv_adapt", "vpv_goodcontact", "vpv_learn")]

init=mice(data.ESM.aggregated, maxit=0)
meth=init$method
predM=init$predictorMatrix
meth[c("ssl_invite", "ssl_visit","ssl_affection", "ssl_comfort", "ssl_compliment", "ssl_interest", "ssl_offerhelp", "ssl_ease", "ssl_advice",
 "ssl_confidence", "ssl_askhelp", "ssl_positivecharacter", "vpv_meetfriends", "vpv_recogniseemotion", "vpv_class", "vpv_help", "vpv_easycontact",
 "vpv_hurt", "vpv_questions", "vpv_sensitivities", "vpv_standup", "vpv_said", "vpv_seekcontact", "vpv_wishes", "vpv_getalong", "vpv_interest",
 "vpv_talkconcerns", "vpv_adapt", "vpv_goodcontact", "vpv_learn")]="pmm"
data.imp.Wave.I <- mice(data.ESM.aggregated, method=meth, predictorMatrix = predM, m = 20)

##
## iter imp variable
## 1 1 ssl_invite ssl_visit ssl_affection ssl_comfort ssl_compliment ssl_interest ssl_offerhelp ssl_ease ssl_advice ssl_confidence ssl_askhelp ssl_positivecharacter vpv_meetfriends vpv_recogniseemotion vpv_class vpv_help vpv_easycontact vpv_hurt vpv_questions vpv_sensitivities vpv_standup vpv_said vpv_seekcontact vpv_wishes vpv_getalong vpv_interest vpv_talkconcerns vpv_adapt vpv_goodcontact vpv_learn
## 1 2 ssl_invite ssl_visit ssl_affection ssl_comfort ssl_compliment ssl_interest ssl_offerhelp ssl_ease ssl_advice ssl_confidence ssl_askhelp ssl_positivecharacter vpv_meetfriends vpv_recogniseemotion vpv_class vpv_help vpv_easycontact vpv_hurt vpv_questions vpv_sensitivities vpv_standup vpv_said vpv_seekcontact vpv_wishes vpv_getalong vpv_interest vpv_talkconcerns vpv_adapt vpv_goodcontact vpv_learn
## 1 3 ssl_invite ssl_visit ssl_affection ssl_comfort ssl_compliment ssl_interest ssl_offerhelp ssl_ease ssl_advice ssl_confidence ssl_askhelp ssl_positivecharacter vpv_meetfriends vpv_recogniseemotion vpv_class vpv_help vpv_easycontact vpv_hurt vpv_questions vpv_sensitivities vpv_standup vpv_said vpv_seekcontact vpv_wishes vpv_getalong vpv_interest vpv_talkconcerns vpv_adapt vpv_goodcontact vpv_learn
## 1 4 ssl_invite ssl_visit ssl_affection ssl_comfort ssl_compliment ssl_interest ssl_offerhelp ssl_ease ssl_advice ssl_confidence ssl_askhelp ssl_positivecharacter vpv_meetfriends vpv_recogniseemotion vpv_class vpv_help vpv_easycontact vpv_hurt vpv_questions vpv_sensitivities vpv_standup vpv_said vpv_seekcontact vpv_wishes vpv_getalong vpv_interest vpv_talkconcerns vpv_adapt vpv_goodcontact vpv_learn
## 1 5 ssl_invite ssl_visit ssl_affection ssl_comfort ssl_compliment ssl_interest ssl_offerhelp ssl_ease ssl_advice ssl_confidence ssl_askhelp ssl_positivecharacter vpv_meetfriends vpv_recogniseemotion vpv_class vpv_help vpv_easycontact vpv_hurt vpv_questions vpv_sensitivities vpv_standup vpv_said vpv_seekcontact vpv_wishes vpv_getalong vpv_interest vpv_talkconcerns vpv_adapt vpv_goodcontact vpv_learn
## 1 6 ssl_invite ssl_visit ssl_affection ssl_comfort ssl_compliment ssl_interest ssl_offerhelp ssl_ease ssl_advice ssl_confidence ssl_askhelp ssl_positivecharacter vpv_meetfriends vpv_recogniseemotion vpv_class vpv_help vpv_easycontact vpv_hurt vpv_questions vpv_sensitivities vpv_standup vpv_said vpv_seekcontact vpv_wishes vpv_getalong vpv_interest vpv_talkconcerns vpv_adapt vpv_goodcontact vpv_learn
## 1 7 ssl_invite ssl_visit ssl_affection ssl_comfort ssl_compliment ssl_interest ssl_offerhelp ssl_ease ssl_advice ssl_confidence ssl_askhelp ssl_positivecharacter vpv_meetfriends vpv_recogniseemotion vpv_class vpv_help vpv_easycontact vpv_hurt vpv_questions vpv_sensitivities vpv_standup vpv_said vpv_seekcontact vpv_wishes vpv_getalong vpv_interest vpv_talkconcerns vpv_adapt vpv_goodcontact vpv_learn
## 1 8 ssl_invite ssl_visit ssl_affection ssl_comfort ssl_compliment ssl_interest ssl_offerhelp ssl_ease ssl_advice ssl_confidence ssl_askhelp ssl_positivecharacter vpv_meetfriends vpv_recogniseemotion vpv_class vpv_help vpv_easycontact vpv_hurt vpv_questions vpv_sensitivities vpv_standup vpv_said vpv_seekcontact vpv_wishes vpv_getalong vpv_interest vpv_talkconcerns vpv_adapt vpv_goodcontact vpv_learn
## 1 9 ssl_invite ssl_visit ssl_affection ssl_comfort ssl_compliment ssl_interest ssl_offerhelp ssl_ease ssl_advice ssl_confidence ssl_askhelp ssl_positivecharacter vpv_meetfriends vpv_recogniseemotion vpv_class vpv_help vpv_easycontact vpv_hurt vpv_questions vpv_sensitivities vpv_standup vpv_said vpv_seekcontact vpv_wishes vpv_getalong vpv_interest vpv_talkconcerns vpv_adapt vpv_goodcontact vpv_learn
## 1 10 ssl_invite ssl_visit ssl_affection ssl_comfort ssl_compliment ssl_interest ssl_offerhelp ssl_ease ssl_advice ssl_confidence ssl_askhelp ssl_positivecharacter vpv_meetfriends vpv_recogniseemotion vpv_class vpv_help vpv_easycontact vpv_hurt vpv_questions vpv_sensitivities vpv_standup vpv_said vpv_seekcontact vpv_wishes vpv_getalong vpv_interest vpv_talkconcerns vpv_adapt vpv_goodcontact vpv_learn
## 1 11 ssl_invite ssl_visit ssl_affection ssl_comfort ssl_compliment ssl_interest ssl_offerhelp ssl_ease ssl_advice ssl_confidence ssl_askhelp ssl_positivecharacter vpv_meetfriends vpv_recogniseemotion vpv_class vpv_help vpv_easycontact vpv_hurt vpv_questions vpv_sensitivities vpv_standup vpv_said vpv_seekcontact vpv_wishes vpv_getalong vpv_interest vpv_talkconcerns vpv_adapt vpv_goodcontact vpv_learn
## 1 12 ssl_invite ssl_visit ssl_affection ssl_comfort ssl_compliment ssl_interest ssl_offerhelp ssl_ease ssl_advice ssl_confidence ssl_askhelp ssl_positivecharacter vpv_meetfriends vpv_recogniseemotion vpv_class vpv_help vpv_easycontact vpv_hurt vpv_questions vpv_sensitivities vpv_standup vpv_said vpv_seekcontact vpv_wishes vpv_getalong vpv_interest vpv_talkconcerns vpv_adapt vpv_goodcontact vpv_learn
## 1 13 ssl_invite ssl_visit ssl_affection ssl_comfort ssl_compliment ssl_interest ssl_offerhelp ssl_ease ssl_advice ssl_confidence ssl_askhelp ssl_positivecharacter vpv_meetfriends vpv_recogniseemotion vpv_class vpv_help vpv_easycontact vpv_hurt vpv_questions vpv_sensitivities vpv_standup vpv_said vpv_seekcontact vpv_wishes vpv_getalong vpv_interest vpv_talkconcerns vpv_adapt vpv_goodcontact vpv_learn
## 1 14 ssl_invite ssl_visit ssl_affection ssl_comfort ssl_compliment ssl_interest ssl_offerhelp ssl_ease ssl_advice ssl_confidence ssl_askhelp ssl_positivecharacter vpv_meetfriends vpv_recogniseemotion vpv_class vpv_help vpv_easycontact vpv_hurt vpv_questions vpv_sensitivities vpv_standup vpv_said vpv_seekcontact vpv_wishes vpv_getalong vpv_interest vpv_talkconcerns vpv_adapt vpv_goodcontact vpv_learn
## 1 15 ssl_invite ssl_visit ssl_affection ssl_comfort ssl_compliment ssl_interest ssl_offerhelp ssl_ease ssl_advice ssl_confidence ssl_askhelp ssl_positivecharacter vpv_meetfriends vpv_recogniseemotion vpv_class vpv_help vpv_easycontact vpv_hurt vpv_questions vpv_sensitivities vpv_standup vpv_said vpv_seekcontact vpv_wishes vpv_getalong vpv_interest vpv_talkconcerns vpv_adapt vpv_goodcontact vpv_learn
## 1 16 ssl_invite ssl_visit ssl_affection ssl_comfort ssl_compliment ssl_interest ssl_offerhelp ssl_ease ssl_advice ssl_confidence ssl_askhelp ssl_positivecharacter vpv_meetfriends vpv_recogniseemotion vpv_class vpv_help vpv_easycontact vpv_hurt vpv_questions vpv_sensitivities vpv_standup vpv_said vpv_seekcontact vpv_wishes vpv_getalong vpv_interest vpv_talkconcerns vpv_adapt vpv_goodcontact vpv_learn
## 1 17 ssl_invite ssl_visit ssl_affection ssl_comfort ssl_compliment ssl_interest ssl_offerhelp ssl_ease ssl_advice ssl_confidence ssl_askhelp ssl_positivecharacter vpv_meetfriends vpv_recogniseemotion vpv_class vpv_help vpv_easycontact vpv_hurt vpv_questions vpv_sensitivities vpv_standup vpv_said vpv_seekcontact vpv_wishes vpv_getalong vpv_interest vpv_talkconcerns vpv_adapt vpv_goodcontact vpv_learn
## 1 18 ssl_invite ssl_visit ssl_affection ssl_comfort ssl_compliment ssl_interest ssl_offerhelp ssl_ease ssl_advice ssl_confidence ssl_askhelp ssl_positivecharacter vpv_meetfriends vpv_recogniseemotion vpv_class vpv_help vpv_easycontact vpv_hurt vpv_questions vpv_sensitivities vpv_standup vpv_said vpv_seekcontact vpv_wishes vpv_getalong vpv_interest vpv_talkconcerns vpv_adapt vpv_goodcontact vpv_learn
## 1 19 ssl_invite ssl_visit ssl_affection ssl_comfort ssl_compliment ssl_interest ssl_offerhelp ssl_ease ssl_advice ssl_confidence ssl_askhelp ssl_positivecharacter vpv_meetfriends vpv_recogniseemotion vpv_class vpv_help vpv_easycontact vpv_hurt vpv_questions vpv_sensitivities vpv_standup vpv_said vpv_seekcontact vpv_wishes vpv_getalong vpv_interest vpv_talkconcerns vpv_adapt vpv_goodcontact vpv_learn
## 1 20 ssl_invite ssl_visit ssl_affection ssl_comfort ssl_compliment ssl_interest ssl_offerhelp ssl_ease ssl_advice ssl_confidence ssl_askhelp ssl_positivecharacter vpv_meetfriends vpv_recogniseemotion vpv_class vpv_help vpv_easycontact vpv_hurt vpv_questions vpv_sensitivities vpv_standup vpv_said vpv_seekcontact vpv_wishes vpv_getalong vpv_interest vpv_talkconcerns vpv_adapt vpv_goodcontact vpv_learn
## 2 1 ssl_invite ssl_visit ssl_affection ssl_comfort ssl_compliment ssl_interest ssl_offerhelp ssl_ease ssl_advice ssl_confidence ssl_askhelp ssl_positivecharacter vpv_meetfriends vpv_recogniseemotion vpv_class vpv_help vpv_easycontact vpv_hurt vpv_questions vpv_sensitivities vpv_standup vpv_said vpv_seekcontact vpv_wishes vpv_getalong vpv_interest vpv_talkconcerns vpv_adapt vpv_goodcontact vpv_learn
## 2 2 ssl_invite ssl_visit ssl_affection ssl_comfort ssl_compliment ssl_interest ssl_offerhelp ssl_ease ssl_advice ssl_confidence ssl_askhelp ssl_positivecharacter vpv_meetfriends vpv_recogniseemotion vpv_class vpv_help vpv_easycontact vpv_hurt vpv_questions vpv_sensitivities vpv_standup vpv_said vpv_seekcontact vpv_wishes vpv_getalong vpv_interest vpv_talkconcerns vpv_adapt vpv_goodcontact vpv_learn
## 2 3 ssl_invite ssl_visit ssl_affection ssl_comfort ssl_compliment ssl_interest ssl_offerhelp ssl_ease ssl_advice ssl_confidence ssl_askhelp ssl_positivecharacter vpv_meetfriends vpv_recogniseemotion vpv_class vpv_help vpv_easycontact vpv_hurt vpv_questions vpv_sensitivities vpv_standup vpv_said vpv_seekcontact vpv_wishes vpv_getalong vpv_interest vpv_talkconcerns vpv_adapt vpv_goodcontact vpv_learn
## 2 4 ssl_invite ssl_visit ssl_affection ssl_comfort ssl_compliment ssl_interest ssl_offerhelp ssl_ease ssl_advice ssl_confidence ssl_askhelp ssl_positivecharacter vpv_meetfriends vpv_recogniseemotion vpv_class vpv_help vpv_easycontact vpv_hurt vpv_questions vpv_sensitivities vpv_standup vpv_said vpv_seekcontact vpv_wishes vpv_getalong vpv_interest vpv_talkconcerns vpv_adapt vpv_goodcontact vpv_learn
## 2 5 ssl_invite ssl_visit ssl_affection ssl_comfort ssl_compliment ssl_interest ssl_offerhelp ssl_ease ssl_advice ssl_confidence ssl_askhelp ssl_positivecharacter vpv_meetfriends vpv_recogniseemotion vpv_class vpv_help vpv_easycontact vpv_hurt vpv_questions vpv_sensitivities vpv_standup vpv_said vpv_seekcontact vpv_wishes vpv_getalong vpv_interest vpv_talkconcerns vpv_adapt vpv_goodcontact vpv_learn
## 2 6 ssl_invite ssl_visit ssl_affection ssl_comfort ssl_compliment ssl_interest ssl_offerhelp ssl_ease ssl_advice ssl_confidence ssl_askhelp ssl_positivecharacter vpv_meetfriends vpv_recogniseemotion vpv_class vpv_help vpv_easycontact vpv_hurt vpv_questions vpv_sensitivities vpv_standup vpv_said vpv_seekcontact vpv_wishes vpv_getalong vpv_interest vpv_talkconcerns vpv_adapt vpv_goodcontact vpv_learn
## 2 7 ssl_invite ssl_visit ssl_affection ssl_comfort ssl_compliment ssl_interest ssl_offerhelp ssl_ease ssl_advice ssl_confidence ssl_askhelp ssl_positivecharacter vpv_meetfriends vpv_recogniseemotion vpv_class vpv_help vpv_easycontact vpv_hurt vpv_questions vpv_sensitivities vpv_standup vpv_said vpv_seekcontact vpv_wishes vpv_getalong vpv_interest vpv_talkconcerns vpv_adapt vpv_goodcontact vpv_learn
## 2 8 ssl_invite ssl_visit ssl_affection ssl_comfort ssl_compliment ssl_interest ssl_offerhelp ssl_ease ssl_advice ssl_confidence ssl_askhelp ssl_positivecharacter vpv_meetfriends vpv_recogniseemotion vpv_class vpv_help vpv_easycontact vpv_hurt vpv_questions vpv_sensitivities vpv_standup vpv_said vpv_seekcontact vpv_wishes vpv_getalong vpv_interest vpv_talkconcerns vpv_adapt vpv_goodcontact vpv_learn
## 2 9 ssl_invite ssl_visit ssl_affection ssl_comfort ssl_compliment ssl_interest ssl_offerhelp ssl_ease ssl_advice ssl_confidence ssl_askhelp ssl_positivecharacter vpv_meetfriends vpv_recogniseemotion vpv_class vpv_help vpv_easycontact vpv_hurt vpv_questions vpv_sensitivities vpv_standup vpv_said vpv_seekcontact vpv_wishes vpv_getalong vpv_interest vpv_talkconcerns vpv_adapt vpv_goodcontact vpv_learn
## 2 10 ssl_invite ssl_visit ssl_affection ssl_comfort ssl_compliment ssl_interest ssl_offerhelp ssl_ease ssl_advice ssl_confidence ssl_askhelp ssl_positivecharacter vpv_meetfriends vpv_recogniseemotion vpv_class vpv_help vpv_easycontact vpv_hurt vpv_questions vpv_sensitivities vpv_standup vpv_said vpv_seekcontact vpv_wishes vpv_getalong vpv_interest vpv_talkconcerns vpv_adapt vpv_goodcontact vpv_learn
## 2 11 ssl_invite ssl_visit ssl_affection ssl_comfort ssl_compliment ssl_interest ssl_offerhelp ssl_ease ssl_advice ssl_confidence ssl_askhelp ssl_positivecharacter vpv_meetfriends vpv_recogniseemotion vpv_class vpv_help vpv_easycontact vpv_hurt vpv_questions vpv_sensitivities vpv_standup vpv_said vpv_seekcontact vpv_wishes vpv_getalong vpv_interest vpv_talkconcerns vpv_adapt vpv_goodcontact vpv_learn
## 2 12 ssl_invite ssl_visit ssl_affection ssl_comfort ssl_compliment ssl_interest ssl_offerhelp ssl_ease ssl_advice ssl_confidence ssl_askhelp ssl_positivecharacter vpv_meetfriends vpv_recogniseemotion vpv_class vpv_help vpv_easycontact vpv_hurt vpv_questions vpv_sensitivities vpv_standup vpv_said vpv_seekcontact vpv_wishes vpv_getalong vpv_interest vpv_talkconcerns vpv_adapt vpv_goodcontact vpv_learn
## 2 13 ssl_invite ssl_visit ssl_affection ssl_comfort ssl_compliment ssl_interest ssl_offerhelp ssl_ease ssl_advice ssl_confidence ssl_askhelp ssl_positivecharacter vpv_meetfriends vpv_recogniseemotion vpv_class vpv_help vpv_easycontact vpv_hurt vpv_questions vpv_sensitivities vpv_standup vpv_said vpv_seekcontact vpv_wishes vpv_getalong vpv_interest vpv_talkconcerns vpv_adapt vpv_goodcontact vpv_learn
## 2 14 ssl_invite ssl_visit ssl_affection ssl_comfort ssl_compliment ssl_interest ssl_offerhelp ssl_ease ssl_advice ssl_confidence ssl_askhelp ssl_positivecharacter vpv_meetfriends vpv_recogniseemotion vpv_class vpv_help vpv_easycontact vpv_hurt vpv_questions vpv_sensitivities vpv_standup vpv_said vpv_seekcontact vpv_wishes vpv_getalong vpv_interest vpv_talkconcerns vpv_adapt vpv_goodcontact vpv_learn
## 2 15 ssl_invite ssl_visit ssl_affection ssl_comfort ssl_compliment ssl_interest ssl_offerhelp ssl_ease ssl_advice ssl_confidence ssl_askhelp ssl_positivecharacter vpv_meetfriends vpv_recogniseemotion vpv_class vpv_help vpv_easycontact vpv_hurt vpv_questions vpv_sensitivities vpv_standup vpv_said vpv_seekcontact vpv_wishes vpv_getalong vpv_interest vpv_talkconcerns vpv_adapt vpv_goodcontact vpv_learn
## 2 16 ssl_invite ssl_visit ssl_affection ssl_comfort ssl_compliment ssl_interest ssl_offerhelp ssl_ease ssl_advice ssl_confidence ssl_askhelp ssl_positivecharacter vpv_meetfriends vpv_recogniseemotion vpv_class vpv_help vpv_easycontact vpv_hurt vpv_questions vpv_sensitivities vpv_standup vpv_said vpv_seekcontact vpv_wishes vpv_getalong vpv_interest vpv_talkconcerns vpv_adapt vpv_goodcontact vpv_learn
## 2 17 ssl_invite ssl_visit ssl_affection ssl_comfort ssl_compliment ssl_interest ssl_offerhelp ssl_ease ssl_advice ssl_confidence ssl_askhelp ssl_positivecharacter vpv_meetfriends vpv_recogniseemotion vpv_class vpv_help vpv_easycontact vpv_hurt vpv_questions vpv_sensitivities vpv_standup vpv_said vpv_seekcontact vpv_wishes vpv_getalong vpv_interest vpv_talkconcerns vpv_adapt vpv_goodcontact vpv_learn
## 2 18 ssl_invite ssl_visit ssl_affection ssl_comfort ssl_compliment ssl_interest ssl_offerhelp ssl_ease ssl_advice ssl_confidence ssl_askhelp ssl_positivecharacter vpv_meetfriends vpv_recogniseemotion vpv_class vpv_help vpv_easycontact vpv_hurt vpv_questions vpv_sensitivities vpv_standup vpv_said vpv_seekcontact vpv_wishes vpv_getalong vpv_interest vpv_talkconcerns vpv_adapt vpv_goodcontact vpv_learn
## 2 19 ssl_invite ssl_visit ssl_affection ssl_comfort ssl_compliment ssl_interest ssl_offerhelp ssl_ease ssl_advice ssl_confidence ssl_askhelp ssl_positivecharacter vpv_meetfriends vpv_recogniseemotion vpv_class vpv_help vpv_easycontact vpv_hurt vpv_questions vpv_sensitivities vpv_standup vpv_said vpv_seekcontact vpv_wishes vpv_getalong vpv_interest vpv_talkconcerns vpv_adapt vpv_goodcontact vpv_learn
## 2 20 ssl_invite ssl_visit ssl_affection ssl_comfort ssl_compliment ssl_interest ssl_offerhelp ssl_ease ssl_advice ssl_confidence ssl_askhelp ssl_positivecharacter vpv_meetfriends vpv_recogniseemotion vpv_class vpv_help vpv_easycontact vpv_hurt vpv_questions vpv_sensitivities vpv_standup vpv_said vpv_seekcontact vpv_wishes vpv_getalong vpv_interest vpv_talkconcerns vpv_adapt vpv_goodcontact vpv_learn
## 3 1 ssl_invite ssl_visit ssl_affection ssl_comfort ssl_compliment ssl_interest ssl_offerhelp ssl_ease ssl_advice ssl_confidence ssl_askhelp ssl_positivecharacter vpv_meetfriends vpv_recogniseemotion vpv_class vpv_help vpv_easycontact vpv_hurt vpv_questions vpv_sensitivities vpv_standup vpv_said vpv_seekcontact vpv_wishes vpv_getalong vpv_interest vpv_talkconcerns vpv_adapt vpv_goodcontact vpv_learn
## 3 2 ssl_invite ssl_visit ssl_affection ssl_comfort ssl_compliment ssl_interest ssl_offerhelp ssl_ease ssl_advice ssl_confidence ssl_askhelp ssl_positivecharacter vpv_meetfriends vpv_recogniseemotion vpv_class vpv_help vpv_easycontact vpv_hurt vpv_questions vpv_sensitivities vpv_standup vpv_said vpv_seekcontact vpv_wishes vpv_getalong vpv_interest vpv_talkconcerns vpv_adapt vpv_goodcontact vpv_learn
## 3 3 ssl_invite ssl_visit ssl_affection ssl_comfort ssl_compliment ssl_interest ssl_offerhelp ssl_ease ssl_advice ssl_confidence ssl_askhelp ssl_positivecharacter vpv_meetfriends vpv_recogniseemotion vpv_class vpv_help vpv_easycontact vpv_hurt vpv_questions vpv_sensitivities vpv_standup vpv_said vpv_seekcontact vpv_wishes vpv_getalong vpv_interest vpv_talkconcerns vpv_adapt vpv_goodcontact vpv_learn
## 3 4 ssl_invite ssl_visit ssl_affection ssl_comfort ssl_compliment ssl_interest ssl_offerhelp ssl_ease ssl_advice ssl_confidence ssl_askhelp ssl_positivecharacter vpv_meetfriends vpv_recogniseemotion vpv_class vpv_help vpv_easycontact vpv_hurt vpv_questions vpv_sensitivities vpv_standup vpv_said vpv_seekcontact vpv_wishes vpv_getalong vpv_interest vpv_talkconcerns vpv_adapt vpv_goodcontact vpv_learn
## 3 5 ssl_invite ssl_visit ssl_affection ssl_comfort ssl_compliment ssl_interest ssl_offerhelp ssl_ease ssl_advice ssl_confidence ssl_askhelp ssl_positivecharacter vpv_meetfriends vpv_recogniseemotion vpv_class vpv_help vpv_easycontact vpv_hurt vpv_questions vpv_sensitivities vpv_standup vpv_said vpv_seekcontact vpv_wishes vpv_getalong vpv_interest vpv_talkconcerns vpv_adapt vpv_goodcontact vpv_learn
## 3 6 ssl_invite ssl_visit ssl_affection ssl_comfort ssl_compliment ssl_interest ssl_offerhelp ssl_ease ssl_advice ssl_confidence ssl_askhelp ssl_positivecharacter vpv_meetfriends vpv_recogniseemotion vpv_class vpv_help vpv_easycontact vpv_hurt vpv_questions vpv_sensitivities vpv_standup vpv_said vpv_seekcontact vpv_wishes vpv_getalong vpv_interest vpv_talkconcerns vpv_adapt vpv_goodcontact vpv_learn
## 3 7 ssl_invite ssl_visit ssl_affection ssl_comfort ssl_compliment ssl_interest ssl_offerhelp ssl_ease ssl_advice ssl_confidence ssl_askhelp ssl_positivecharacter vpv_meetfriends vpv_recogniseemotion vpv_class vpv_help vpv_easycontact vpv_hurt vpv_questions vpv_sensitivities vpv_standup vpv_said vpv_seekcontact vpv_wishes vpv_getalong vpv_interest vpv_talkconcerns vpv_adapt vpv_goodcontact vpv_learn
## 3 8 ssl_invite ssl_visit ssl_affection ssl_comfort ssl_compliment ssl_interest ssl_offerhelp ssl_ease ssl_advice ssl_confidence ssl_askhelp ssl_positivecharacter vpv_meetfriends vpv_recogniseemotion vpv_class vpv_help vpv_easycontact vpv_hurt vpv_questions vpv_sensitivities vpv_standup vpv_said vpv_seekcontact vpv_wishes vpv_getalong vpv_interest vpv_talkconcerns vpv_adapt vpv_goodcontact vpv_learn
## 3 9 ssl_invite ssl_visit ssl_affection ssl_comfort ssl_compliment ssl_interest ssl_offerhelp ssl_ease ssl_advice ssl_confidence ssl_askhelp ssl_positivecharacter vpv_meetfriends vpv_recogniseemotion vpv_class vpv_help vpv_easycontact vpv_hurt vpv_questions vpv_sensitivities vpv_standup vpv_said vpv_seekcontact vpv_wishes vpv_getalong vpv_interest vpv_talkconcerns vpv_adapt vpv_goodcontact vpv_learn
## 3 10 ssl_invite ssl_visit ssl_affection ssl_comfort ssl_compliment ssl_interest ssl_offerhelp ssl_ease ssl_advice ssl_confidence ssl_askhelp ssl_positivecharacter vpv_meetfriends vpv_recogniseemotion vpv_class vpv_help vpv_easycontact vpv_hurt vpv_questions vpv_sensitivities vpv_standup vpv_said vpv_seekcontact vpv_wishes vpv_getalong vpv_interest vpv_talkconcerns vpv_adapt vpv_goodcontact vpv_learn
## 3 11 ssl_invite ssl_visit ssl_affection ssl_comfort ssl_compliment ssl_interest ssl_offerhelp ssl_ease ssl_advice ssl_confidence ssl_askhelp ssl_positivecharacter vpv_meetfriends vpv_recogniseemotion vpv_class vpv_help vpv_easycontact vpv_hurt vpv_questions vpv_sensitivities vpv_standup vpv_said vpv_seekcontact vpv_wishes vpv_getalong vpv_interest vpv_talkconcerns vpv_adapt vpv_goodcontact vpv_learn
## 3 12 ssl_invite ssl_visit ssl_affection ssl_comfort ssl_compliment ssl_interest ssl_offerhelp ssl_ease ssl_advice ssl_confidence ssl_askhelp ssl_positivecharacter vpv_meetfriends vpv_recogniseemotion vpv_class vpv_help vpv_easycontact vpv_hurt vpv_questions vpv_sensitivities vpv_standup vpv_said vpv_seekcontact vpv_wishes vpv_getalong vpv_interest vpv_talkconcerns vpv_adapt vpv_goodcontact vpv_learn
## 3 13 ssl_invite ssl_visit ssl_affection ssl_comfort ssl_compliment ssl_interest ssl_offerhelp ssl_ease ssl_advice ssl_confidence ssl_askhelp ssl_positivecharacter vpv_meetfriends vpv_recogniseemotion vpv_class vpv_help vpv_easycontact vpv_hurt vpv_questions vpv_sensitivities vpv_standup vpv_said vpv_seekcontact vpv_wishes vpv_getalong vpv_interest vpv_talkconcerns vpv_adapt vpv_goodcontact vpv_learn
## 3 14 ssl_invite ssl_visit ssl_affection ssl_comfort ssl_compliment ssl_interest ssl_offerhelp ssl_ease ssl_advice ssl_confidence ssl_askhelp ssl_positivecharacter vpv_meetfriends vpv_recogniseemotion vpv_class vpv_help vpv_easycontact vpv_hurt vpv_questions vpv_sensitivities vpv_standup vpv_said vpv_seekcontact vpv_wishes vpv_getalong vpv_interest vpv_talkconcerns vpv_adapt vpv_goodcontact vpv_learn
## 3 15 ssl_invite ssl_visit ssl_affection ssl_comfort ssl_compliment ssl_interest ssl_offerhelp ssl_ease ssl_advice ssl_confidence ssl_askhelp ssl_positivecharacter vpv_meetfriends vpv_recogniseemotion vpv_class vpv_help vpv_easycontact vpv_hurt vpv_questions vpv_sensitivities vpv_standup vpv_said vpv_seekcontact vpv_wishes vpv_getalong vpv_interest vpv_talkconcerns vpv_adapt vpv_goodcontact vpv_learn
## 3 16 ssl_invite ssl_visit ssl_affection ssl_comfort ssl_compliment ssl_interest ssl_offerhelp ssl_ease ssl_advice ssl_confidence ssl_askhelp ssl_positivecharacter vpv_meetfriends vpv_recogniseemotion vpv_class vpv_help vpv_easycontact vpv_hurt vpv_questions vpv_sensitivities vpv_standup vpv_said vpv_seekcontact vpv_wishes vpv_getalong vpv_interest vpv_talkconcerns vpv_adapt vpv_goodcontact vpv_learn
## 3 17 ssl_invite ssl_visit ssl_affection ssl_comfort ssl_compliment ssl_interest ssl_offerhelp ssl_ease ssl_advice ssl_confidence ssl_askhelp ssl_positivecharacter vpv_meetfriends vpv_recogniseemotion vpv_class vpv_help vpv_easycontact vpv_hurt vpv_questions vpv_sensitivities vpv_standup vpv_said vpv_seekcontact vpv_wishes vpv_getalong vpv_interest vpv_talkconcerns vpv_adapt vpv_goodcontact vpv_learn
## 3 18 ssl_invite ssl_visit ssl_affection ssl_comfort ssl_compliment ssl_interest ssl_offerhelp ssl_ease ssl_advice ssl_confidence ssl_askhelp ssl_positivecharacter vpv_meetfriends vpv_recogniseemotion vpv_class vpv_help vpv_easycontact vpv_hurt vpv_questions vpv_sensitivities vpv_standup vpv_said vpv_seekcontact vpv_wishes vpv_getalong vpv_interest vpv_talkconcerns vpv_adapt vpv_goodcontact vpv_learn
## 3 19 ssl_invite ssl_visit ssl_affection ssl_comfort ssl_compliment ssl_interest ssl_offerhelp ssl_ease ssl_advice ssl_confidence ssl_askhelp ssl_positivecharacter vpv_meetfriends vpv_recogniseemotion vpv_class vpv_help vpv_easycontact vpv_hurt vpv_questions vpv_sensitivities vpv_standup vpv_said vpv_seekcontact vpv_wishes vpv_getalong vpv_interest vpv_talkconcerns vpv_adapt vpv_goodcontact vpv_learn
## 3 20 ssl_invite ssl_visit ssl_affection ssl_comfort ssl_compliment ssl_interest ssl_offerhelp ssl_ease ssl_advice ssl_confidence ssl_askhelp ssl_positivecharacter vpv_meetfriends vpv_recogniseemotion vpv_class vpv_help vpv_easycontact vpv_hurt vpv_questions vpv_sensitivities vpv_standup vpv_said vpv_seekcontact vpv_wishes vpv_getalong vpv_interest vpv_talkconcerns vpv_adapt vpv_goodcontact vpv_learn
## 4 1 ssl_invite ssl_visit ssl_affection ssl_comfort ssl_compliment ssl_interest ssl_offerhelp ssl_ease ssl_advice ssl_confidence ssl_askhelp ssl_positivecharacter vpv_meetfriends vpv_recogniseemotion vpv_class vpv_help vpv_easycontact vpv_hurt vpv_questions vpv_sensitivities vpv_standup vpv_said vpv_seekcontact vpv_wishes vpv_getalong vpv_interest vpv_talkconcerns vpv_adapt vpv_goodcontact vpv_learn
## 4 2 ssl_invite ssl_visit ssl_affection ssl_comfort ssl_compliment ssl_interest ssl_offerhelp ssl_ease ssl_advice ssl_confidence ssl_askhelp ssl_positivecharacter vpv_meetfriends vpv_recogniseemotion vpv_class vpv_help vpv_easycontact vpv_hurt vpv_questions vpv_sensitivities vpv_standup vpv_said vpv_seekcontact vpv_wishes vpv_getalong vpv_interest vpv_talkconcerns vpv_adapt vpv_goodcontact vpv_learn
## 4 3 ssl_invite ssl_visit ssl_affection ssl_comfort ssl_compliment ssl_interest ssl_offerhelp ssl_ease ssl_advice ssl_confidence ssl_askhelp ssl_positivecharacter vpv_meetfriends vpv_recogniseemotion vpv_class vpv_help vpv_easycontact vpv_hurt vpv_questions vpv_sensitivities vpv_standup vpv_said vpv_seekcontact vpv_wishes vpv_getalong vpv_interest vpv_talkconcerns vpv_adapt vpv_goodcontact vpv_learn
## 4 4 ssl_invite ssl_visit ssl_affection ssl_comfort ssl_compliment ssl_interest ssl_offerhelp ssl_ease ssl_advice ssl_confidence ssl_askhelp ssl_positivecharacter vpv_meetfriends vpv_recogniseemotion vpv_class vpv_help vpv_easycontact vpv_hurt vpv_questions vpv_sensitivities vpv_standup vpv_said vpv_seekcontact vpv_wishes vpv_getalong vpv_interest vpv_talkconcerns vpv_adapt vpv_goodcontact vpv_learn
## 4 5 ssl_invite ssl_visit ssl_affection ssl_comfort ssl_compliment ssl_interest ssl_offerhelp ssl_ease ssl_advice ssl_confidence ssl_askhelp ssl_positivecharacter vpv_meetfriends vpv_recogniseemotion vpv_class vpv_help vpv_easycontact vpv_hurt vpv_questions vpv_sensitivities vpv_standup vpv_said vpv_seekcontact vpv_wishes vpv_getalong vpv_interest vpv_talkconcerns vpv_adapt vpv_goodcontact vpv_learn
## 4 6 ssl_invite ssl_visit ssl_affection ssl_comfort ssl_compliment ssl_interest ssl_offerhelp ssl_ease ssl_advice ssl_confidence ssl_askhelp ssl_positivecharacter vpv_meetfriends vpv_recogniseemotion vpv_class vpv_help vpv_easycontact vpv_hurt vpv_questions vpv_sensitivities vpv_standup vpv_said vpv_seekcontact vpv_wishes vpv_getalong vpv_interest vpv_talkconcerns vpv_adapt vpv_goodcontact vpv_learn
## 4 7 ssl_invite ssl_visit ssl_affection ssl_comfort ssl_compliment ssl_interest ssl_offerhelp ssl_ease ssl_advice ssl_confidence ssl_askhelp ssl_positivecharacter vpv_meetfriends vpv_recogniseemotion vpv_class vpv_help vpv_easycontact vpv_hurt vpv_questions vpv_sensitivities vpv_standup vpv_said vpv_seekcontact vpv_wishes vpv_getalong vpv_interest vpv_talkconcerns vpv_adapt vpv_goodcontact vpv_learn
## 4 8 ssl_invite ssl_visit ssl_affection ssl_comfort ssl_compliment ssl_interest ssl_offerhelp ssl_ease ssl_advice ssl_confidence ssl_askhelp ssl_positivecharacter vpv_meetfriends vpv_recogniseemotion vpv_class vpv_help vpv_easycontact vpv_hurt vpv_questions vpv_sensitivities vpv_standup vpv_said vpv_seekcontact vpv_wishes vpv_getalong vpv_interest vpv_talkconcerns vpv_adapt vpv_goodcontact vpv_learn
## 4 9 ssl_invite ssl_visit ssl_affection ssl_comfort ssl_compliment ssl_interest ssl_offerhelp ssl_ease ssl_advice ssl_confidence ssl_askhelp ssl_positivecharacter vpv_meetfriends vpv_recogniseemotion vpv_class vpv_help vpv_easycontact vpv_hurt vpv_questions vpv_sensitivities vpv_standup vpv_said vpv_seekcontact vpv_wishes vpv_getalong vpv_interest vpv_talkconcerns vpv_adapt vpv_goodcontact vpv_learn
## 4 10 ssl_invite ssl_visit ssl_affection ssl_comfort ssl_compliment ssl_interest ssl_offerhelp ssl_ease ssl_advice ssl_confidence ssl_askhelp ssl_positivecharacter vpv_meetfriends vpv_recogniseemotion vpv_class vpv_help vpv_easycontact vpv_hurt vpv_questions vpv_sensitivities vpv_standup vpv_said vpv_seekcontact vpv_wishes vpv_getalong vpv_interest vpv_talkconcerns vpv_adapt vpv_goodcontact vpv_learn
## 4 11 ssl_invite ssl_visit ssl_affection ssl_comfort ssl_compliment ssl_interest ssl_offerhelp ssl_ease ssl_advice ssl_confidence ssl_askhelp ssl_positivecharacter vpv_meetfriends vpv_recogniseemotion vpv_class vpv_help vpv_easycontact vpv_hurt vpv_questions vpv_sensitivities vpv_standup vpv_said vpv_seekcontact vpv_wishes vpv_getalong vpv_interest vpv_talkconcerns vpv_adapt vpv_goodcontact vpv_learn
## 4 12 ssl_invite ssl_visit ssl_affection ssl_comfort ssl_compliment ssl_interest ssl_offerhelp ssl_ease ssl_advice ssl_confidence ssl_askhelp ssl_positivecharacter vpv_meetfriends vpv_recogniseemotion vpv_class vpv_help vpv_easycontact vpv_hurt vpv_questions vpv_sensitivities vpv_standup vpv_said vpv_seekcontact vpv_wishes vpv_getalong vpv_interest vpv_talkconcerns vpv_adapt vpv_goodcontact vpv_learn
## 4 13 ssl_invite ssl_visit ssl_affection ssl_comfort ssl_compliment ssl_interest ssl_offerhelp ssl_ease ssl_advice ssl_confidence ssl_askhelp ssl_positivecharacter vpv_meetfriends vpv_recogniseemotion vpv_class vpv_help vpv_easycontact vpv_hurt vpv_questions vpv_sensitivities vpv_standup vpv_said vpv_seekcontact vpv_wishes vpv_getalong vpv_interest vpv_talkconcerns vpv_adapt vpv_goodcontact vpv_learn
## 4 14 ssl_invite ssl_visit ssl_affection ssl_comfort ssl_compliment ssl_interest ssl_offerhelp ssl_ease ssl_advice ssl_confidence ssl_askhelp ssl_positivecharacter vpv_meetfriends vpv_recogniseemotion vpv_class vpv_help vpv_easycontact vpv_hurt vpv_questions vpv_sensitivities vpv_standup vpv_said vpv_seekcontact vpv_wishes vpv_getalong vpv_interest vpv_talkconcerns vpv_adapt vpv_goodcontact vpv_learn
## 4 15 ssl_invite ssl_visit ssl_affection ssl_comfort ssl_compliment ssl_interest ssl_offerhelp ssl_ease ssl_advice ssl_confidence ssl_askhelp ssl_positivecharacter vpv_meetfriends vpv_recogniseemotion vpv_class vpv_help vpv_easycontact vpv_hurt vpv_questions vpv_sensitivities vpv_standup vpv_said vpv_seekcontact vpv_wishes vpv_getalong vpv_interest vpv_talkconcerns vpv_adapt vpv_goodcontact vpv_learn
## 4 16 ssl_invite ssl_visit ssl_affection ssl_comfort ssl_compliment ssl_interest ssl_offerhelp ssl_ease ssl_advice ssl_confidence ssl_askhelp ssl_positivecharacter vpv_meetfriends vpv_recogniseemotion vpv_class vpv_help vpv_easycontact vpv_hurt vpv_questions vpv_sensitivities vpv_standup vpv_said vpv_seekcontact vpv_wishes vpv_getalong vpv_interest vpv_talkconcerns vpv_adapt vpv_goodcontact vpv_learn
## 4 17 ssl_invite ssl_visit ssl_affection ssl_comfort ssl_compliment ssl_interest ssl_offerhelp ssl_ease ssl_advice ssl_confidence ssl_askhelp ssl_positivecharacter vpv_meetfriends vpv_recogniseemotion vpv_class vpv_help vpv_easycontact vpv_hurt vpv_questions vpv_sensitivities vpv_standup vpv_said vpv_seekcontact vpv_wishes vpv_getalong vpv_interest vpv_talkconcerns vpv_adapt vpv_goodcontact vpv_learn
## 4 18 ssl_invite ssl_visit ssl_affection ssl_comfort ssl_compliment ssl_interest ssl_offerhelp ssl_ease ssl_advice ssl_confidence ssl_askhelp ssl_positivecharacter vpv_meetfriends vpv_recogniseemotion vpv_class vpv_help vpv_easycontact vpv_hurt vpv_questions vpv_sensitivities vpv_standup vpv_said vpv_seekcontact vpv_wishes vpv_getalong vpv_interest vpv_talkconcerns vpv_adapt vpv_goodcontact vpv_learn
## 4 19 ssl_invite ssl_visit ssl_affection ssl_comfort ssl_compliment ssl_interest ssl_offerhelp ssl_ease ssl_advice ssl_confidence ssl_askhelp ssl_positivecharacter vpv_meetfriends vpv_recogniseemotion vpv_class vpv_help vpv_easycontact vpv_hurt vpv_questions vpv_sensitivities vpv_standup vpv_said vpv_seekcontact vpv_wishes vpv_getalong vpv_interest vpv_talkconcerns vpv_adapt vpv_goodcontact vpv_learn
## 4 20 ssl_invite ssl_visit ssl_affection ssl_comfort ssl_compliment ssl_interest ssl_offerhelp ssl_ease ssl_advice ssl_confidence ssl_askhelp ssl_positivecharacter vpv_meetfriends vpv_recogniseemotion vpv_class vpv_help vpv_easycontact vpv_hurt vpv_questions vpv_sensitivities vpv_standup vpv_said vpv_seekcontact vpv_wishes vpv_getalong vpv_interest vpv_talkconcerns vpv_adapt vpv_goodcontact vpv_learn
## 5 1 ssl_invite ssl_visit ssl_affection ssl_comfort ssl_compliment ssl_interest ssl_offerhelp ssl_ease ssl_advice ssl_confidence ssl_askhelp ssl_positivecharacter vpv_meetfriends vpv_recogniseemotion vpv_class vpv_help vpv_easycontact vpv_hurt vpv_questions vpv_sensitivities vpv_standup vpv_said vpv_seekcontact vpv_wishes vpv_getalong vpv_interest vpv_talkconcerns vpv_adapt vpv_goodcontact vpv_learn
## 5 2 ssl_invite ssl_visit ssl_affection ssl_comfort ssl_compliment ssl_interest ssl_offerhelp ssl_ease ssl_advice ssl_confidence ssl_askhelp ssl_positivecharacter vpv_meetfriends vpv_recogniseemotion vpv_class vpv_help vpv_easycontact vpv_hurt vpv_questions vpv_sensitivities vpv_standup vpv_said vpv_seekcontact vpv_wishes vpv_getalong vpv_interest vpv_talkconcerns vpv_adapt vpv_goodcontact vpv_learn
## 5 3 ssl_invite ssl_visit ssl_affection ssl_comfort ssl_compliment ssl_interest ssl_offerhelp ssl_ease ssl_advice ssl_confidence ssl_askhelp ssl_positivecharacter vpv_meetfriends vpv_recogniseemotion vpv_class vpv_help vpv_easycontact vpv_hurt vpv_questions vpv_sensitivities vpv_standup vpv_said vpv_seekcontact vpv_wishes vpv_getalong vpv_interest vpv_talkconcerns vpv_adapt vpv_goodcontact vpv_learn
## 5 4 ssl_invite ssl_visit ssl_affection ssl_comfort ssl_compliment ssl_interest ssl_offerhelp ssl_ease ssl_advice ssl_confidence ssl_askhelp ssl_positivecharacter vpv_meetfriends vpv_recogniseemotion vpv_class vpv_help vpv_easycontact vpv_hurt vpv_questions vpv_sensitivities vpv_standup vpv_said vpv_seekcontact vpv_wishes vpv_getalong vpv_interest vpv_talkconcerns vpv_adapt vpv_goodcontact vpv_learn
## 5 5 ssl_invite ssl_visit ssl_affection ssl_comfort ssl_compliment ssl_interest ssl_offerhelp ssl_ease ssl_advice ssl_confidence ssl_askhelp ssl_positivecharacter vpv_meetfriends vpv_recogniseemotion vpv_class vpv_help vpv_easycontact vpv_hurt vpv_questions vpv_sensitivities vpv_standup vpv_said vpv_seekcontact vpv_wishes vpv_getalong vpv_interest vpv_talkconcerns vpv_adapt vpv_goodcontact vpv_learn
## 5 6 ssl_invite ssl_visit ssl_affection ssl_comfort ssl_compliment ssl_interest ssl_offerhelp ssl_ease ssl_advice ssl_confidence ssl_askhelp ssl_positivecharacter vpv_meetfriends vpv_recogniseemotion vpv_class vpv_help vpv_easycontact vpv_hurt vpv_questions vpv_sensitivities vpv_standup vpv_said vpv_seekcontact vpv_wishes vpv_getalong vpv_interest vpv_talkconcerns vpv_adapt vpv_goodcontact vpv_learn
## 5 7 ssl_invite ssl_visit ssl_affection ssl_comfort ssl_compliment ssl_interest ssl_offerhelp ssl_ease ssl_advice ssl_confidence ssl_askhelp ssl_positivecharacter vpv_meetfriends vpv_recogniseemotion vpv_class vpv_help vpv_easycontact vpv_hurt vpv_questions vpv_sensitivities vpv_standup vpv_said vpv_seekcontact vpv_wishes vpv_getalong vpv_interest vpv_talkconcerns vpv_adapt vpv_goodcontact vpv_learn
## 5 8 ssl_invite ssl_visit ssl_affection ssl_comfort ssl_compliment ssl_interest ssl_offerhelp ssl_ease ssl_advice ssl_confidence ssl_askhelp ssl_positivecharacter vpv_meetfriends vpv_recogniseemotion vpv_class vpv_help vpv_easycontact vpv_hurt vpv_questions vpv_sensitivities vpv_standup vpv_said vpv_seekcontact vpv_wishes vpv_getalong vpv_interest vpv_talkconcerns vpv_adapt vpv_goodcontact vpv_learn
## 5 9 ssl_invite ssl_visit ssl_affection ssl_comfort ssl_compliment ssl_interest ssl_offerhelp ssl_ease ssl_advice ssl_confidence ssl_askhelp ssl_positivecharacter vpv_meetfriends vpv_recogniseemotion vpv_class vpv_help vpv_easycontact vpv_hurt vpv_questions vpv_sensitivities vpv_standup vpv_said vpv_seekcontact vpv_wishes vpv_getalong vpv_interest vpv_talkconcerns vpv_adapt vpv_goodcontact vpv_learn
## 5 10 ssl_invite ssl_visit ssl_affection ssl_comfort ssl_compliment ssl_interest ssl_offerhelp ssl_ease ssl_advice ssl_confidence ssl_askhelp ssl_positivecharacter vpv_meetfriends vpv_recogniseemotion vpv_class vpv_help vpv_easycontact vpv_hurt vpv_questions vpv_sensitivities vpv_standup vpv_said vpv_seekcontact vpv_wishes vpv_getalong vpv_interest vpv_talkconcerns vpv_adapt vpv_goodcontact vpv_learn
## 5 11 ssl_invite ssl_visit ssl_affection ssl_comfort ssl_compliment ssl_interest ssl_offerhelp ssl_ease ssl_advice ssl_confidence ssl_askhelp ssl_positivecharacter vpv_meetfriends vpv_recogniseemotion vpv_class vpv_help vpv_easycontact vpv_hurt vpv_questions vpv_sensitivities vpv_standup vpv_said vpv_seekcontact vpv_wishes vpv_getalong vpv_interest vpv_talkconcerns vpv_adapt vpv_goodcontact vpv_learn
## 5 12 ssl_invite ssl_visit ssl_affection ssl_comfort ssl_compliment ssl_interest ssl_offerhelp ssl_ease ssl_advice ssl_confidence ssl_askhelp ssl_positivecharacter vpv_meetfriends vpv_recogniseemotion vpv_class vpv_help vpv_easycontact vpv_hurt vpv_questions vpv_sensitivities vpv_standup vpv_said vpv_seekcontact vpv_wishes vpv_getalong vpv_interest vpv_talkconcerns vpv_adapt vpv_goodcontact vpv_learn
## 5 13 ssl_invite ssl_visit ssl_affection ssl_comfort ssl_compliment ssl_interest ssl_offerhelp ssl_ease ssl_advice ssl_confidence ssl_askhelp ssl_positivecharacter vpv_meetfriends vpv_recogniseemotion vpv_class vpv_help vpv_easycontact vpv_hurt vpv_questions vpv_sensitivities vpv_standup vpv_said vpv_seekcontact vpv_wishes vpv_getalong vpv_interest vpv_talkconcerns vpv_adapt vpv_goodcontact vpv_learn
## 5 14 ssl_invite ssl_visit ssl_affection ssl_comfort ssl_compliment ssl_interest ssl_offerhelp ssl_ease ssl_advice ssl_confidence ssl_askhelp ssl_positivecharacter vpv_meetfriends vpv_recogniseemotion vpv_class vpv_help vpv_easycontact vpv_hurt vpv_questions vpv_sensitivities vpv_standup vpv_said vpv_seekcontact vpv_wishes vpv_getalong vpv_interest vpv_talkconcerns vpv_adapt vpv_goodcontact vpv_learn
## 5 15 ssl_invite ssl_visit ssl_affection ssl_comfort ssl_compliment ssl_interest ssl_offerhelp ssl_ease ssl_advice ssl_confidence ssl_askhelp ssl_positivecharacter vpv_meetfriends vpv_recogniseemotion vpv_class vpv_help vpv_easycontact vpv_hurt vpv_questions vpv_sensitivities vpv_standup vpv_said vpv_seekcontact vpv_wishes vpv_getalong vpv_interest vpv_talkconcerns vpv_adapt vpv_goodcontact vpv_learn
## 5 16 ssl_invite ssl_visit ssl_affection ssl_comfort ssl_compliment ssl_interest ssl_offerhelp ssl_ease ssl_advice ssl_confidence ssl_askhelp ssl_positivecharacter vpv_meetfriends vpv_recogniseemotion vpv_class vpv_help vpv_easycontact vpv_hurt vpv_questions vpv_sensitivities vpv_standup vpv_said vpv_seekcontact vpv_wishes vpv_getalong vpv_interest vpv_talkconcerns vpv_adapt vpv_goodcontact vpv_learn
## 5 17 ssl_invite ssl_visit ssl_affection ssl_comfort ssl_compliment ssl_interest ssl_offerhelp ssl_ease ssl_advice ssl_confidence ssl_askhelp ssl_positivecharacter vpv_meetfriends vpv_recogniseemotion vpv_class vpv_help vpv_easycontact vpv_hurt vpv_questions vpv_sensitivities vpv_standup vpv_said vpv_seekcontact vpv_wishes vpv_getalong vpv_interest vpv_talkconcerns vpv_adapt vpv_goodcontact vpv_learn
## 5 18 ssl_invite ssl_visit ssl_affection ssl_comfort ssl_compliment ssl_interest ssl_offerhelp ssl_ease ssl_advice ssl_confidence ssl_askhelp ssl_positivecharacter vpv_meetfriends vpv_recogniseemotion vpv_class vpv_help vpv_easycontact vpv_hurt vpv_questions vpv_sensitivities vpv_standup vpv_said vpv_seekcontact vpv_wishes vpv_getalong vpv_interest vpv_talkconcerns vpv_adapt vpv_goodcontact vpv_learn
## 5 19 ssl_invite ssl_visit ssl_affection ssl_comfort ssl_compliment ssl_interest ssl_offerhelp ssl_ease ssl_advice ssl_confidence ssl_askhelp ssl_positivecharacter vpv_meetfriends vpv_recogniseemotion vpv_class vpv_help vpv_easycontact vpv_hurt vpv_questions vpv_sensitivities vpv_standup vpv_said vpv_seekcontact vpv_wishes vpv_getalong vpv_interest vpv_talkconcerns vpv_adapt vpv_goodcontact vpv_learn
## 5 20 ssl_invite ssl_visit ssl_affection ssl_comfort ssl_compliment ssl_interest ssl_offerhelp ssl_ease ssl_advice ssl_confidence ssl_askhelp ssl_positivecharacter vpv_meetfriends vpv_recogniseemotion vpv_class vpv_help vpv_easycontact vpv_hurt vpv_questions vpv_sensitivities vpv_standup vpv_said vpv_seekcontact vpv_wishes vpv_getalong vpv_interest vpv_talkconcerns vpv_adapt vpv_goodcontact vpv_learn

#Imputing the MSPSS
PLdat_Wave2 = PLdat[PLdat$wave=='wave2',]

data.ESM.aggregated = PLdat_Wave2[,c("record_id", "mspss_person_help", "mspss_person_joy", "mspss_family_help", "mspss_family_support",
 "mspss_person_comfort", "mspss_friends_help", "mspss_friends_count", "mspss_family_talk", "mspss_friends_joy",
 "mspss_person_feelings", "mspss_family_decide", "mspss_friends_talk","vpv_meetfriends", "vpv_recogniseemotion",
 "vpv_class", "vpv_help", "vpv_easycontact", "vpv_hurt", "vpv_questions", "vpv_sensitivities", "vpv_standup", "vpv_said",
 "vpv_seekcontact", "vpv_wishes", "vpv_getalong", "vpv_interest", "vpv_talkconcerns", "vpv_adapt", "vpv_goodcontact",
 "vpv_learn")]

init=mice(data.ESM.aggregated, maxit=0)
meth=init$method
predM=init$predictorMatrix
meth[c("mspss_person_help", "mspss_person_joy", "mspss_family_help", "mspss_family_support", "mspss_person_comfort", "mspss_friends_help",
 "mspss_friends_count", "mspss_family_talk", "mspss_friends_joy", "mspss_person_feelings", "mspss_family_decide", "mspss_friends_talk", "vpv_meetfriends",
 "vpv_recogniseemotion", "vpv_class", "vpv_help", "vpv_easycontact", "vpv_hurt", "vpv_questions", "vpv_sensitivities", "vpv_standup", "vpv_said",
 "vpv_seekcontact", "vpv_wishes", "vpv_getalong", "vpv_interest", "vpv_talkconcerns", "vpv_adapt", "vpv_goodcontact", "vpv_learn")]="pmm"
data.imp.Wave.II <- mice(data.ESM.aggregated, method=meth, predictorMatrix = predM, m = 20)

##
## iter imp variable
## 1 1 mspss_person_help mspss_person_joy mspss_family_help mspss_family_support mspss_person_comfort mspss_friends_help mspss_friends_count mspss_family_talk mspss_friends_joy mspss_person_feelings mspss_family_decide mspss_friends_talk vpv_meetfriends vpv_recogniseemotion vpv_class vpv_help vpv_easycontact vpv_hurt vpv_questions vpv_sensitivities vpv_standup vpv_said vpv_seekcontact vpv_wishes vpv_getalong vpv_interest vpv_talkconcerns vpv_adapt vpv_goodcontact vpv_learn
## 1 2 mspss_person_help mspss_person_joy mspss_family_help mspss_family_support mspss_person_comfort mspss_friends_help mspss_friends_count mspss_family_talk mspss_friends_joy mspss_person_feelings mspss_family_decide mspss_friends_talk vpv_meetfriends vpv_recogniseemotion vpv_class vpv_help vpv_easycontact vpv_hurt vpv_questions vpv_sensitivities vpv_standup vpv_said vpv_seekcontact vpv_wishes vpv_getalong vpv_interest vpv_talkconcerns vpv_adapt vpv_goodcontact vpv_learn
## 1 3 mspss_person_help mspss_person_joy mspss_family_help mspss_family_support mspss_person_comfort mspss_friends_help mspss_friends_count mspss_family_talk mspss_friends_joy mspss_person_feelings mspss_family_decide mspss_friends_talk vpv_meetfriends vpv_recogniseemotion vpv_class vpv_help vpv_easycontact vpv_hurt vpv_questions vpv_sensitivities vpv_standup vpv_said vpv_seekcontact vpv_wishes vpv_getalong vpv_interest vpv_talkconcerns vpv_adapt vpv_goodcontact vpv_learn
## 1 4 mspss_person_help mspss_person_joy mspss_family_help mspss_family_support mspss_person_comfort mspss_friends_help mspss_friends_count mspss_family_talk mspss_friends_joy mspss_person_feelings mspss_family_decide mspss_friends_talk vpv_meetfriends vpv_recogniseemotion vpv_class vpv_help vpv_easycontact vpv_hurt vpv_questions vpv_sensitivities vpv_standup vpv_said vpv_seekcontact vpv_wishes vpv_getalong vpv_interest vpv_talkconcerns vpv_adapt vpv_goodcontact vpv_learn
## 1 5 mspss_person_help mspss_person_joy mspss_family_help mspss_family_support mspss_person_comfort mspss_friends_help mspss_friends_count mspss_family_talk mspss_friends_joy mspss_person_feelings mspss_family_decide mspss_friends_talk vpv_meetfriends vpv_recogniseemotion vpv_class vpv_help vpv_easycontact vpv_hurt vpv_questions vpv_sensitivities vpv_standup vpv_said vpv_seekcontact vpv_wishes vpv_getalong vpv_interest vpv_talkconcerns vpv_adapt vpv_goodcontact vpv_learn
## 1 6 mspss_person_help mspss_person_joy mspss_family_help mspss_family_support mspss_person_comfort mspss_friends_help mspss_friends_count mspss_family_talk mspss_friends_joy mspss_person_feelings mspss_family_decide mspss_friends_talk vpv_meetfriends vpv_recogniseemotion vpv_class vpv_help vpv_easycontact vpv_hurt vpv_questions vpv_sensitivities vpv_standup vpv_said vpv_seekcontact vpv_wishes vpv_getalong vpv_interest vpv_talkconcerns vpv_adapt vpv_goodcontact vpv_learn
## 1 7 mspss_person_help mspss_person_joy mspss_family_help mspss_family_support mspss_person_comfort mspss_friends_help mspss_friends_count mspss_family_talk mspss_friends_joy mspss_person_feelings mspss_family_decide mspss_friends_talk vpv_meetfriends vpv_recogniseemotion vpv_class vpv_help vpv_easycontact vpv_hurt vpv_questions vpv_sensitivities vpv_standup vpv_said vpv_seekcontact vpv_wishes vpv_getalong vpv_interest vpv_talkconcerns vpv_adapt vpv_goodcontact vpv_learn
## 1 8 mspss_person_help mspss_person_joy mspss_family_help mspss_family_support mspss_person_comfort mspss_friends_help mspss_friends_count mspss_family_talk mspss_friends_joy mspss_person_feelings mspss_family_decide mspss_friends_talk vpv_meetfriends vpv_recogniseemotion vpv_class vpv_help vpv_easycontact vpv_hurt vpv_questions vpv_sensitivities vpv_standup vpv_said vpv_seekcontact vpv_wishes vpv_getalong vpv_interest vpv_talkconcerns vpv_adapt vpv_goodcontact vpv_learn
## 1 9 mspss_person_help mspss_person_joy mspss_family_help mspss_family_support mspss_person_comfort mspss_friends_help mspss_friends_count mspss_family_talk mspss_friends_joy mspss_person_feelings mspss_family_decide mspss_friends_talk vpv_meetfriends vpv_recogniseemotion vpv_class vpv_help vpv_easycontact vpv_hurt vpv_questions vpv_sensitivities vpv_standup vpv_said vpv_seekcontact vpv_wishes vpv_getalong vpv_interest vpv_talkconcerns vpv_adapt vpv_goodcontact vpv_learn
## 1 10 mspss_person_help mspss_person_joy mspss_family_help mspss_family_support mspss_person_comfort mspss_friends_help mspss_friends_count mspss_family_talk mspss_friends_joy mspss_person_feelings mspss_family_decide mspss_friends_talk vpv_meetfriends vpv_recogniseemotion vpv_class vpv_help vpv_easycontact vpv_hurt vpv_questions vpv_sensitivities vpv_standup vpv_said vpv_seekcontact vpv_wishes vpv_getalong vpv_interest vpv_talkconcerns vpv_adapt vpv_goodcontact vpv_learn
## 1 11 mspss_person_help mspss_person_joy mspss_family_help mspss_family_support mspss_person_comfort mspss_friends_help mspss_friends_count mspss_family_talk mspss_friends_joy mspss_person_feelings mspss_family_decide mspss_friends_talk vpv_meetfriends vpv_recogniseemotion vpv_class vpv_help vpv_easycontact vpv_hurt vpv_questions vpv_sensitivities vpv_standup vpv_said vpv_seekcontact vpv_wishes vpv_getalong vpv_interest vpv_talkconcerns vpv_adapt vpv_goodcontact vpv_learn
## 1 12 mspss_person_help mspss_person_joy mspss_family_help mspss_family_support mspss_person_comfort mspss_friends_help mspss_friends_count mspss_family_talk mspss_friends_joy mspss_person_feelings mspss_family_decide mspss_friends_talk vpv_meetfriends vpv_recogniseemotion vpv_class vpv_help vpv_easycontact vpv_hurt vpv_questions vpv_sensitivities vpv_standup vpv_said vpv_seekcontact vpv_wishes vpv_getalong vpv_interest vpv_talkconcerns vpv_adapt vpv_goodcontact vpv_learn
## 1 13 mspss_person_help mspss_person_joy mspss_family_help mspss_family_support mspss_person_comfort mspss_friends_help mspss_friends_count mspss_family_talk mspss_friends_joy mspss_person_feelings mspss_family_decide mspss_friends_talk vpv_meetfriends vpv_recogniseemotion vpv_class vpv_help vpv_easycontact vpv_hurt vpv_questions vpv_sensitivities vpv_standup vpv_said vpv_seekcontact vpv_wishes vpv_getalong vpv_interest vpv_talkconcerns vpv_adapt vpv_goodcontact vpv_learn
## 1 14 mspss_person_help mspss_person_joy mspss_family_help mspss_family_support mspss_person_comfort mspss_friends_help mspss_friends_count mspss_family_talk mspss_friends_joy mspss_person_feelings mspss_family_decide mspss_friends_talk vpv_meetfriends vpv_recogniseemotion vpv_class vpv_help vpv_easycontact vpv_hurt vpv_questions vpv_sensitivities vpv_standup vpv_said vpv_seekcontact vpv_wishes vpv_getalong vpv_interest vpv_talkconcerns vpv_adapt vpv_goodcontact vpv_learn
## 1 15 mspss_person_help mspss_person_joy mspss_family_help mspss_family_support mspss_person_comfort mspss_friends_help mspss_friends_count mspss_family_talk mspss_friends_joy mspss_person_feelings mspss_family_decide mspss_friends_talk vpv_meetfriends vpv_recogniseemotion vpv_class vpv_help vpv_easycontact vpv_hurt vpv_questions vpv_sensitivities vpv_standup vpv_said vpv_seekcontact vpv_wishes vpv_getalong vpv_interest vpv_talkconcerns vpv_adapt vpv_goodcontact vpv_learn
## 1 16 mspss_person_help mspss_person_joy mspss_family_help mspss_family_support mspss_person_comfort mspss_friends_help mspss_friends_count mspss_family_talk mspss_friends_joy mspss_person_feelings mspss_family_decide mspss_friends_talk vpv_meetfriends vpv_recogniseemotion vpv_class vpv_help vpv_easycontact vpv_hurt vpv_questions vpv_sensitivities vpv_standup vpv_said vpv_seekcontact vpv_wishes vpv_getalong vpv_interest vpv_talkconcerns vpv_adapt vpv_goodcontact vpv_learn
## 1 17 mspss_person_help mspss_person_joy mspss_family_help mspss_family_support mspss_person_comfort mspss_friends_help mspss_friends_count mspss_family_talk mspss_friends_joy mspss_person_feelings mspss_family_decide mspss_friends_talk vpv_meetfriends vpv_recogniseemotion vpv_class vpv_help vpv_easycontact vpv_hurt vpv_questions vpv_sensitivities vpv_standup vpv_said vpv_seekcontact vpv_wishes vpv_getalong vpv_interest vpv_talkconcerns vpv_adapt vpv_goodcontact vpv_learn
## 1 18 mspss_person_help mspss_person_joy mspss_family_help mspss_family_support mspss_person_comfort mspss_friends_help mspss_friends_count mspss_family_talk mspss_friends_joy mspss_person_feelings mspss_family_decide mspss_friends_talk vpv_meetfriends vpv_recogniseemotion vpv_class vpv_help vpv_easycontact vpv_hurt vpv_questions vpv_sensitivities vpv_standup vpv_said vpv_seekcontact vpv_wishes vpv_getalong vpv_interest vpv_talkconcerns vpv_adapt vpv_goodcontact vpv_learn
## 1 19 mspss_person_help mspss_person_joy mspss_family_help mspss_family_support mspss_person_comfort mspss_friends_help mspss_friends_count mspss_family_talk mspss_friends_joy mspss_person_feelings mspss_family_decide mspss_friends_talk vpv_meetfriends vpv_recogniseemotion vpv_class vpv_help vpv_easycontact vpv_hurt vpv_questions vpv_sensitivities vpv_standup vpv_said vpv_seekcontact vpv_wishes vpv_getalong vpv_interest vpv_talkconcerns vpv_adapt vpv_goodcontact vpv_learn
## 1 20 mspss_person_help mspss_person_joy mspss_family_help mspss_family_support mspss_person_comfort mspss_friends_help mspss_friends_count mspss_family_talk mspss_friends_joy mspss_person_feelings mspss_family_decide mspss_friends_talk vpv_meetfriends vpv_recogniseemotion vpv_class vpv_help vpv_easycontact vpv_hurt vpv_questions vpv_sensitivities vpv_standup vpv_said vpv_seekcontact vpv_wishes vpv_getalong vpv_interest vpv_talkconcerns vpv_adapt vpv_goodcontact vpv_learn
## 2 1 mspss_person_help mspss_person_joy mspss_family_help mspss_family_support mspss_person_comfort mspss_friends_help mspss_friends_count mspss_family_talk mspss_friends_joy mspss_person_feelings mspss_family_decide mspss_friends_talk vpv_meetfriends vpv_recogniseemotion vpv_class vpv_help vpv_easycontact vpv_hurt vpv_questions vpv_sensitivities vpv_standup vpv_said vpv_seekcontact vpv_wishes vpv_getalong vpv_interest vpv_talkconcerns vpv_adapt vpv_goodcontact vpv_learn
## 2 2 mspss_person_help mspss_person_joy mspss_family_help mspss_family_support mspss_person_comfort mspss_friends_help mspss_friends_count mspss_family_talk mspss_friends_joy mspss_person_feelings mspss_family_decide mspss_friends_talk vpv_meetfriends vpv_recogniseemotion vpv_class vpv_help vpv_easycontact vpv_hurt vpv_questions vpv_sensitivities vpv_standup vpv_said vpv_seekcontact vpv_wishes vpv_getalong vpv_interest vpv_talkconcerns vpv_adapt vpv_goodcontact vpv_learn
## 2 3 mspss_person_help mspss_person_joy mspss_family_help mspss_family_support mspss_person_comfort mspss_friends_help mspss_friends_count mspss_family_talk mspss_friends_joy mspss_person_feelings mspss_family_decide mspss_friends_talk vpv_meetfriends vpv_recogniseemotion vpv_class vpv_help vpv_easycontact vpv_hurt vpv_questions vpv_sensitivities vpv_standup vpv_said vpv_seekcontact vpv_wishes vpv_getalong vpv_interest vpv_talkconcerns vpv_adapt vpv_goodcontact vpv_learn
## 2 4 mspss_person_help mspss_person_joy mspss_family_help mspss_family_support mspss_person_comfort mspss_friends_help mspss_friends_count mspss_family_talk mspss_friends_joy mspss_person_feelings mspss_family_decide mspss_friends_talk vpv_meetfriends vpv_recogniseemotion vpv_class vpv_help vpv_easycontact vpv_hurt vpv_questions vpv_sensitivities vpv_standup vpv_said vpv_seekcontact vpv_wishes vpv_getalong vpv_interest vpv_talkconcerns vpv_adapt vpv_goodcontact vpv_learn
## 2 5 mspss_person_help mspss_person_joy mspss_family_help mspss_family_support mspss_person_comfort mspss_friends_help mspss_friends_count mspss_family_talk mspss_friends_joy mspss_person_feelings mspss_family_decide mspss_friends_talk vpv_meetfriends vpv_recogniseemotion vpv_class vpv_help vpv_easycontact vpv_hurt vpv_questions vpv_sensitivities vpv_standup vpv_said vpv_seekcontact vpv_wishes vpv_getalong vpv_interest vpv_talkconcerns vpv_adapt vpv_goodcontact vpv_learn
## 2 6 mspss_person_help mspss_person_joy mspss_family_help mspss_family_support mspss_person_comfort mspss_friends_help mspss_friends_count mspss_family_talk mspss_friends_joy mspss_person_feelings mspss_family_decide mspss_friends_talk vpv_meetfriends vpv_recogniseemotion vpv_class vpv_help vpv_easycontact vpv_hurt vpv_questions vpv_sensitivities vpv_standup vpv_said vpv_seekcontact vpv_wishes vpv_getalong vpv_interest vpv_talkconcerns vpv_adapt vpv_goodcontact vpv_learn
## 2 7 mspss_person_help mspss_person_joy mspss_family_help mspss_family_support mspss_person_comfort mspss_friends_help mspss_friends_count mspss_family_talk mspss_friends_joy mspss_person_feelings mspss_family_decide mspss_friends_talk vpv_meetfriends vpv_recogniseemotion vpv_class vpv_help vpv_easycontact vpv_hurt vpv_questions vpv_sensitivities vpv_standup vpv_said vpv_seekcontact vpv_wishes vpv_getalong vpv_interest vpv_talkconcerns vpv_adapt vpv_goodcontact vpv_learn
## 2 8 mspss_person_help mspss_person_joy mspss_family_help mspss_family_support mspss_person_comfort mspss_friends_help mspss_friends_count mspss_family_talk mspss_friends_joy mspss_person_feelings mspss_family_decide mspss_friends_talk vpv_meetfriends vpv_recogniseemotion vpv_class vpv_help vpv_easycontact vpv_hurt vpv_questions vpv_sensitivities vpv_standup vpv_said vpv_seekcontact vpv_wishes vpv_getalong vpv_interest vpv_talkconcerns vpv_adapt vpv_goodcontact vpv_learn
## 2 9 mspss_person_help mspss_person_joy mspss_family_help mspss_family_support mspss_person_comfort mspss_friends_help mspss_friends_count mspss_family_talk mspss_friends_joy mspss_person_feelings mspss_family_decide mspss_friends_talk vpv_meetfriends vpv_recogniseemotion vpv_class vpv_help vpv_easycontact vpv_hurt vpv_questions vpv_sensitivities vpv_standup vpv_said vpv_seekcontact vpv_wishes vpv_getalong vpv_interest vpv_talkconcerns vpv_adapt vpv_goodcontact vpv_learn
## 2 10 mspss_person_help mspss_person_joy mspss_family_help mspss_family_support mspss_person_comfort mspss_friends_help mspss_friends_count mspss_family_talk mspss_friends_joy mspss_person_feelings mspss_family_decide mspss_friends_talk vpv_meetfriends vpv_recogniseemotion vpv_class vpv_help vpv_easycontact vpv_hurt vpv_questions vpv_sensitivities vpv_standup vpv_said vpv_seekcontact vpv_wishes vpv_getalong vpv_interest vpv_talkconcerns vpv_adapt vpv_goodcontact vpv_learn
## 2 11 mspss_person_help mspss_person_joy mspss_family_help mspss_family_support mspss_person_comfort mspss_friends_help mspss_friends_count mspss_family_talk mspss_friends_joy mspss_person_feelings mspss_family_decide mspss_friends_talk vpv_meetfriends vpv_recogniseemotion vpv_class vpv_help vpv_easycontact vpv_hurt vpv_questions vpv_sensitivities vpv_standup vpv_said vpv_seekcontact vpv_wishes vpv_getalong vpv_interest vpv_talkconcerns vpv_adapt vpv_goodcontact vpv_learn
## 2 12 mspss_person_help mspss_person_joy mspss_family_help mspss_family_support mspss_person_comfort mspss_friends_help mspss_friends_count mspss_family_talk mspss_friends_joy mspss_person_feelings mspss_family_decide mspss_friends_talk vpv_meetfriends vpv_recogniseemotion vpv_class vpv_help vpv_easycontact vpv_hurt vpv_questions vpv_sensitivities vpv_standup vpv_said vpv_seekcontact vpv_wishes vpv_getalong vpv_interest vpv_talkconcerns vpv_adapt vpv_goodcontact vpv_learn
## 2 13 mspss_person_help mspss_person_joy mspss_family_help mspss_family_support mspss_person_comfort mspss_friends_help mspss_friends_count mspss_family_talk mspss_friends_joy mspss_person_feelings mspss_family_decide mspss_friends_talk vpv_meetfriends vpv_recogniseemotion vpv_class vpv_help vpv_easycontact vpv_hurt vpv_questions vpv_sensitivities vpv_standup vpv_said vpv_seekcontact vpv_wishes vpv_getalong vpv_interest vpv_talkconcerns vpv_adapt vpv_goodcontact vpv_learn
## 2 14 mspss_person_help mspss_person_joy mspss_family_help mspss_family_support mspss_person_comfort mspss_friends_help mspss_friends_count mspss_family_talk mspss_friends_joy mspss_person_feelings mspss_family_decide mspss_friends_talk vpv_meetfriends vpv_recogniseemotion vpv_class vpv_help vpv_easycontact vpv_hurt vpv_questions vpv_sensitivities vpv_standup vpv_said vpv_seekcontact vpv_wishes vpv_getalong vpv_interest vpv_talkconcerns vpv_adapt vpv_goodcontact vpv_learn
## 2 15 mspss_person_help mspss_person_joy mspss_family_help mspss_family_support mspss_person_comfort mspss_friends_help mspss_friends_count mspss_family_talk mspss_friends_joy mspss_person_feelings mspss_family_decide mspss_friends_talk vpv_meetfriends vpv_recogniseemotion vpv_class vpv_help vpv_easycontact vpv_hurt vpv_questions vpv_sensitivities vpv_standup vpv_said vpv_seekcontact vpv_wishes vpv_getalong vpv_interest vpv_talkconcerns vpv_adapt vpv_goodcontact vpv_learn
## 2 16 mspss_person_help mspss_person_joy mspss_family_help mspss_family_support mspss_person_comfort mspss_friends_help mspss_friends_count mspss_family_talk mspss_friends_joy mspss_person_feelings mspss_family_decide mspss_friends_talk vpv_meetfriends vpv_recogniseemotion vpv_class vpv_help vpv_easycontact vpv_hurt vpv_questions vpv_sensitivities vpv_standup vpv_said vpv_seekcontact vpv_wishes vpv_getalong vpv_interest vpv_talkconcerns vpv_adapt vpv_goodcontact vpv_learn
## 2 17 mspss_person_help mspss_person_joy mspss_family_help mspss_family_support mspss_person_comfort mspss_friends_help mspss_friends_count mspss_family_talk mspss_friends_joy mspss_person_feelings mspss_family_decide mspss_friends_talk vpv_meetfriends vpv_recogniseemotion vpv_class vpv_help vpv_easycontact vpv_hurt vpv_questions vpv_sensitivities vpv_standup vpv_said vpv_seekcontact vpv_wishes vpv_getalong vpv_interest vpv_talkconcerns vpv_adapt vpv_goodcontact vpv_learn
## 2 18 mspss_person_help mspss_person_joy mspss_family_help mspss_family_support mspss_person_comfort mspss_friends_help mspss_friends_count mspss_family_talk mspss_friends_joy mspss_person_feelings mspss_family_decide mspss_friends_talk vpv_meetfriends vpv_recogniseemotion vpv_class vpv_help vpv_easycontact vpv_hurt vpv_questions vpv_sensitivities vpv_standup vpv_said vpv_seekcontact vpv_wishes vpv_getalong vpv_interest vpv_talkconcerns vpv_adapt vpv_goodcontact vpv_learn
## 2 19 mspss_person_help mspss_person_joy mspss_family_help mspss_family_support mspss_person_comfort mspss_friends_help mspss_friends_count mspss_family_talk mspss_friends_joy mspss_person_feelings mspss_family_decide mspss_friends_talk vpv_meetfriends vpv_recogniseemotion vpv_class vpv_help vpv_easycontact vpv_hurt vpv_questions vpv_sensitivities vpv_standup vpv_said vpv_seekcontact vpv_wishes vpv_getalong vpv_interest vpv_talkconcerns vpv_adapt vpv_goodcontact vpv_learn
## 2 20 mspss_person_help mspss_person_joy mspss_family_help mspss_family_support mspss_person_comfort mspss_friends_help mspss_friends_count mspss_family_talk mspss_friends_joy mspss_person_feelings mspss_family_decide mspss_friends_talk vpv_meetfriends vpv_recogniseemotion vpv_class vpv_help vpv_easycontact vpv_hurt vpv_questions vpv_sensitivities vpv_standup vpv_said vpv_seekcontact vpv_wishes vpv_getalong vpv_interest vpv_talkconcerns vpv_adapt vpv_goodcontact vpv_learn
## 3 1 mspss_person_help mspss_person_joy mspss_family_help mspss_family_support mspss_person_comfort mspss_friends_help mspss_friends_count mspss_family_talk mspss_friends_joy mspss_person_feelings mspss_family_decide mspss_friends_talk vpv_meetfriends vpv_recogniseemotion vpv_class vpv_help vpv_easycontact vpv_hurt vpv_questions vpv_sensitivities vpv_standup vpv_said vpv_seekcontact vpv_wishes vpv_getalong vpv_interest vpv_talkconcerns vpv_adapt vpv_goodcontact vpv_learn
## 3 2 mspss_person_help mspss_person_joy mspss_family_help mspss_family_support mspss_person_comfort mspss_friends_help mspss_friends_count mspss_family_talk mspss_friends_joy mspss_person_feelings mspss_family_decide mspss_friends_talk vpv_meetfriends vpv_recogniseemotion vpv_class vpv_help vpv_easycontact vpv_hurt vpv_questions vpv_sensitivities vpv_standup vpv_said vpv_seekcontact vpv_wishes vpv_getalong vpv_interest vpv_talkconcerns vpv_adapt vpv_goodcontact vpv_learn
## 3 3 mspss_person_help mspss_person_joy mspss_family_help mspss_family_support mspss_person_comfort mspss_friends_help mspss_friends_count mspss_family_talk mspss_friends_joy mspss_person_feelings mspss_family_decide mspss_friends_talk vpv_meetfriends vpv_recogniseemotion vpv_class vpv_help vpv_easycontact vpv_hurt vpv_questions vpv_sensitivities vpv_standup vpv_said vpv_seekcontact vpv_wishes vpv_getalong vpv_interest vpv_talkconcerns vpv_adapt vpv_goodcontact vpv_learn
## 3 4 mspss_person_help mspss_person_joy mspss_family_help mspss_family_support mspss_person_comfort mspss_friends_help mspss_friends_count mspss_family_talk mspss_friends_joy mspss_person_feelings mspss_family_decide mspss_friends_talk vpv_meetfriends vpv_recogniseemotion vpv_class vpv_help vpv_easycontact vpv_hurt vpv_questions vpv_sensitivities vpv_standup vpv_said vpv_seekcontact vpv_wishes vpv_getalong vpv_interest vpv_talkconcerns vpv_adapt vpv_goodcontact vpv_learn
## 3 5 mspss_person_help mspss_person_joy mspss_family_help mspss_family_support mspss_person_comfort mspss_friends_help mspss_friends_count mspss_family_talk mspss_friends_joy mspss_person_feelings mspss_family_decide mspss_friends_talk vpv_meetfriends vpv_recogniseemotion vpv_class vpv_help vpv_easycontact vpv_hurt vpv_questions vpv_sensitivities vpv_standup vpv_said vpv_seekcontact vpv_wishes vpv_getalong vpv_interest vpv_talkconcerns vpv_adapt vpv_goodcontact vpv_learn
## 3 6 mspss_person_help mspss_person_joy mspss_family_help mspss_family_support mspss_person_comfort mspss_friends_help mspss_friends_count mspss_family_talk mspss_friends_joy mspss_person_feelings mspss_family_decide mspss_friends_talk vpv_meetfriends vpv_recogniseemotion vpv_class vpv_help vpv_easycontact vpv_hurt vpv_questions vpv_sensitivities vpv_standup vpv_said vpv_seekcontact vpv_wishes vpv_getalong vpv_interest vpv_talkconcerns vpv_adapt vpv_goodcontact vpv_learn
## 3 7 mspss_person_help mspss_person_joy mspss_family_help mspss_family_support mspss_person_comfort mspss_friends_help mspss_friends_count mspss_family_talk mspss_friends_joy mspss_person_feelings mspss_family_decide mspss_friends_talk vpv_meetfriends vpv_recogniseemotion vpv_class vpv_help vpv_easycontact vpv_hurt vpv_questions vpv_sensitivities vpv_standup vpv_said vpv_seekcontact vpv_wishes vpv_getalong vpv_interest vpv_talkconcerns vpv_adapt vpv_goodcontact vpv_learn
## 3 8 mspss_person_help mspss_person_joy mspss_family_help mspss_family_support mspss_person_comfort mspss_friends_help mspss_friends_count mspss_family_talk mspss_friends_joy mspss_person_feelings mspss_family_decide mspss_friends_talk vpv_meetfriends vpv_recogniseemotion vpv_class vpv_help vpv_easycontact vpv_hurt vpv_questions vpv_sensitivities vpv_standup vpv_said vpv_seekcontact vpv_wishes vpv_getalong vpv_interest vpv_talkconcerns vpv_adapt vpv_goodcontact vpv_learn
## 3 9 mspss_person_help mspss_person_joy mspss_family_help mspss_family_support mspss_person_comfort mspss_friends_help mspss_friends_count mspss_family_talk mspss_friends_joy mspss_person_feelings mspss_family_decide mspss_friends_talk vpv_meetfriends vpv_recogniseemotion vpv_class vpv_help vpv_easycontact vpv_hurt vpv_questions vpv_sensitivities vpv_standup vpv_said vpv_seekcontact vpv_wishes vpv_getalong vpv_interest vpv_talkconcerns vpv_adapt vpv_goodcontact vpv_learn
## 3 10 mspss_person_help mspss_person_joy mspss_family_help mspss_family_support mspss_person_comfort mspss_friends_help mspss_friends_count mspss_family_talk mspss_friends_joy mspss_person_feelings mspss_family_decide mspss_friends_talk vpv_meetfriends vpv_recogniseemotion vpv_class vpv_help vpv_easycontact vpv_hurt vpv_questions vpv_sensitivities vpv_standup vpv_said vpv_seekcontact vpv_wishes vpv_getalong vpv_interest vpv_talkconcerns vpv_adapt vpv_goodcontact vpv_learn
## 3 11 mspss_person_help mspss_person_joy mspss_family_help mspss_family_support mspss_person_comfort mspss_friends_help mspss_friends_count mspss_family_talk mspss_friends_joy mspss_person_feelings mspss_family_decide mspss_friends_talk vpv_meetfriends vpv_recogniseemotion vpv_class vpv_help vpv_easycontact vpv_hurt vpv_questions vpv_sensitivities vpv_standup vpv_said vpv_seekcontact vpv_wishes vpv_getalong vpv_interest vpv_talkconcerns vpv_adapt vpv_goodcontact vpv_learn
## 3 12 mspss_person_help mspss_person_joy mspss_family_help mspss_family_support mspss_person_comfort mspss_friends_help mspss_friends_count mspss_family_talk mspss_friends_joy mspss_person_feelings mspss_family_decide mspss_friends_talk vpv_meetfriends vpv_recogniseemotion vpv_class vpv_help vpv_easycontact vpv_hurt vpv_questions vpv_sensitivities vpv_standup vpv_said vpv_seekcontact vpv_wishes vpv_getalong vpv_interest vpv_talkconcerns vpv_adapt vpv_goodcontact vpv_learn
## 3 13 mspss_person_help mspss_person_joy mspss_family_help mspss_family_support mspss_person_comfort mspss_friends_help mspss_friends_count mspss_family_talk mspss_friends_joy mspss_person_feelings mspss_family_decide mspss_friends_talk vpv_meetfriends vpv_recogniseemotion vpv_class vpv_help vpv_easycontact vpv_hurt vpv_questions vpv_sensitivities vpv_standup vpv_said vpv_seekcontact vpv_wishes vpv_getalong vpv_interest vpv_talkconcerns vpv_adapt vpv_goodcontact vpv_learn
## 3 14 mspss_person_help mspss_person_joy mspss_family_help mspss_family_support mspss_person_comfort mspss_friends_help mspss_friends_count mspss_family_talk mspss_friends_joy mspss_person_feelings mspss_family_decide mspss_friends_talk vpv_meetfriends vpv_recogniseemotion vpv_class vpv_help vpv_easycontact vpv_hurt vpv_questions vpv_sensitivities vpv_standup vpv_said vpv_seekcontact vpv_wishes vpv_getalong vpv_interest vpv_talkconcerns vpv_adapt vpv_goodcontact vpv_learn
## 3 15 mspss_person_help mspss_person_joy mspss_family_help mspss_family_support mspss_person_comfort mspss_friends_help mspss_friends_count mspss_family_talk mspss_friends_joy mspss_person_feelings mspss_family_decide mspss_friends_talk vpv_meetfriends vpv_recogniseemotion vpv_class vpv_help vpv_easycontact vpv_hurt vpv_questions vpv_sensitivities vpv_standup vpv_said vpv_seekcontact vpv_wishes vpv_getalong vpv_interest vpv_talkconcerns vpv_adapt vpv_goodcontact vpv_learn
## 3 16 mspss_person_help mspss_person_joy mspss_family_help mspss_family_support mspss_person_comfort mspss_friends_help mspss_friends_count mspss_family_talk mspss_friends_joy mspss_person_feelings mspss_family_decide mspss_friends_talk vpv_meetfriends vpv_recogniseemotion vpv_class vpv_help vpv_easycontact vpv_hurt vpv_questions vpv_sensitivities vpv_standup vpv_said vpv_seekcontact vpv_wishes vpv_getalong vpv_interest vpv_talkconcerns vpv_adapt vpv_goodcontact vpv_learn
## 3 17 mspss_person_help mspss_person_joy mspss_family_help mspss_family_support mspss_person_comfort mspss_friends_help mspss_friends_count mspss_family_talk mspss_friends_joy mspss_person_feelings mspss_family_decide mspss_friends_talk vpv_meetfriends vpv_recogniseemotion vpv_class vpv_help vpv_easycontact vpv_hurt vpv_questions vpv_sensitivities vpv_standup vpv_said vpv_seekcontact vpv_wishes vpv_getalong vpv_interest vpv_talkconcerns vpv_adapt vpv_goodcontact vpv_learn
## 3 18 mspss_person_help mspss_person_joy mspss_family_help mspss_family_support mspss_person_comfort mspss_friends_help mspss_friends_count mspss_family_talk mspss_friends_joy mspss_person_feelings mspss_family_decide mspss_friends_talk vpv_meetfriends vpv_recogniseemotion vpv_class vpv_help vpv_easycontact vpv_hurt vpv_questions vpv_sensitivities vpv_standup vpv_said vpv_seekcontact vpv_wishes vpv_getalong vpv_interest vpv_talkconcerns vpv_adapt vpv_goodcontact vpv_learn
## 3 19 mspss_person_help mspss_person_joy mspss_family_help mspss_family_support mspss_person_comfort mspss_friends_help mspss_friends_count mspss_family_talk mspss_friends_joy mspss_person_feelings mspss_family_decide mspss_friends_talk vpv_meetfriends vpv_recogniseemotion vpv_class vpv_help vpv_easycontact vpv_hurt vpv_questions vpv_sensitivities vpv_standup vpv_said vpv_seekcontact vpv_wishes vpv_getalong vpv_interest vpv_talkconcerns vpv_adapt vpv_goodcontact vpv_learn
## 3 20 mspss_person_help mspss_person_joy mspss_family_help mspss_family_support mspss_person_comfort mspss_friends_help mspss_friends_count mspss_family_talk mspss_friends_joy mspss_person_feelings mspss_family_decide mspss_friends_talk vpv_meetfriends vpv_recogniseemotion vpv_class vpv_help vpv_easycontact vpv_hurt vpv_questions vpv_sensitivities vpv_standup vpv_said vpv_seekcontact vpv_wishes vpv_getalong vpv_interest vpv_talkconcerns vpv_adapt vpv_goodcontact vpv_learn
## 4 1 mspss_person_help mspss_person_joy mspss_family_help mspss_family_support mspss_person_comfort mspss_friends_help mspss_friends_count mspss_family_talk mspss_friends_joy mspss_person_feelings mspss_family_decide mspss_friends_talk vpv_meetfriends vpv_recogniseemotion vpv_class vpv_help vpv_easycontact vpv_hurt vpv_questions vpv_sensitivities vpv_standup vpv_said vpv_seekcontact vpv_wishes vpv_getalong vpv_interest vpv_talkconcerns vpv_adapt vpv_goodcontact vpv_learn
## 4 2 mspss_person_help mspss_person_joy mspss_family_help mspss_family_support mspss_person_comfort mspss_friends_help mspss_friends_count mspss_family_talk mspss_friends_joy mspss_person_feelings mspss_family_decide mspss_friends_talk vpv_meetfriends vpv_recogniseemotion vpv_class vpv_help vpv_easycontact vpv_hurt vpv_questions vpv_sensitivities vpv_standup vpv_said vpv_seekcontact vpv_wishes vpv_getalong vpv_interest vpv_talkconcerns vpv_adapt vpv_goodcontact vpv_learn
## 4 3 mspss_person_help mspss_person_joy mspss_family_help mspss_family_support mspss_person_comfort mspss_friends_help mspss_friends_count mspss_family_talk mspss_friends_joy mspss_person_feelings mspss_family_decide mspss_friends_talk vpv_meetfriends vpv_recogniseemotion vpv_class vpv_help vpv_easycontact vpv_hurt vpv_questions vpv_sensitivities vpv_standup vpv_said vpv_seekcontact vpv_wishes vpv_getalong vpv_interest vpv_talkconcerns vpv_adapt vpv_goodcontact vpv_learn
## 4 4 mspss_person_help mspss_person_joy mspss_family_help mspss_family_support mspss_person_comfort mspss_friends_help mspss_friends_count mspss_family_talk mspss_friends_joy mspss_person_feelings mspss_family_decide mspss_friends_talk vpv_meetfriends vpv_recogniseemotion vpv_class vpv_help vpv_easycontact vpv_hurt vpv_questions vpv_sensitivities vpv_standup vpv_said vpv_seekcontact vpv_wishes vpv_getalong vpv_interest vpv_talkconcerns vpv_adapt vpv_goodcontact vpv_learn
## 4 5 mspss_person_help mspss_person_joy mspss_family_help mspss_family_support mspss_person_comfort mspss_friends_help mspss_friends_count mspss_family_talk mspss_friends_joy mspss_person_feelings mspss_family_decide mspss_friends_talk vpv_meetfriends vpv_recogniseemotion vpv_class vpv_help vpv_easycontact vpv_hurt vpv_questions vpv_sensitivities vpv_standup vpv_said vpv_seekcontact vpv_wishes vpv_getalong vpv_interest vpv_talkconcerns vpv_adapt vpv_goodcontact vpv_learn
## 4 6 mspss_person_help mspss_person_joy mspss_family_help mspss_family_support mspss_person_comfort mspss_friends_help mspss_friends_count mspss_family_talk mspss_friends_joy mspss_person_feelings mspss_family_decide mspss_friends_talk vpv_meetfriends vpv_recogniseemotion vpv_class vpv_help vpv_easycontact vpv_hurt vpv_questions vpv_sensitivities vpv_standup vpv_said vpv_seekcontact vpv_wishes vpv_getalong vpv_interest vpv_talkconcerns vpv_adapt vpv_goodcontact vpv_learn
## 4 7 mspss_person_help mspss_person_joy mspss_family_help mspss_family_support mspss_person_comfort mspss_friends_help mspss_friends_count mspss_family_talk mspss_friends_joy mspss_person_feelings mspss_family_decide mspss_friends_talk vpv_meetfriends vpv_recogniseemotion vpv_class vpv_help vpv_easycontact vpv_hurt vpv_questions vpv_sensitivities vpv_standup vpv_said vpv_seekcontact vpv_wishes vpv_getalong vpv_interest vpv_talkconcerns vpv_adapt vpv_goodcontact vpv_learn
## 4 8 mspss_person_help mspss_person_joy mspss_family_help mspss_family_support mspss_person_comfort mspss_friends_help mspss_friends_count mspss_family_talk mspss_friends_joy mspss_person_feelings mspss_family_decide mspss_friends_talk vpv_meetfriends vpv_recogniseemotion vpv_class vpv_help vpv_easycontact vpv_hurt vpv_questions vpv_sensitivities vpv_standup vpv_said vpv_seekcontact vpv_wishes vpv_getalong vpv_interest vpv_talkconcerns vpv_adapt vpv_goodcontact vpv_learn
## 4 9 mspss_person_help mspss_person_joy mspss_family_help mspss_family_support mspss_person_comfort mspss_friends_help mspss_friends_count mspss_family_talk mspss_friends_joy mspss_person_feelings mspss_family_decide mspss_friends_talk vpv_meetfriends vpv_recogniseemotion vpv_class vpv_help vpv_easycontact vpv_hurt vpv_questions vpv_sensitivities vpv_standup vpv_said vpv_seekcontact vpv_wishes vpv_getalong vpv_interest vpv_talkconcerns vpv_adapt vpv_goodcontact vpv_learn
## 4 10 mspss_person_help mspss_person_joy mspss_family_help mspss_family_support mspss_person_comfort mspss_friends_help mspss_friends_count mspss_family_talk mspss_friends_joy mspss_person_feelings mspss_family_decide mspss_friends_talk vpv_meetfriends vpv_recogniseemotion vpv_class vpv_help vpv_easycontact vpv_hurt vpv_questions vpv_sensitivities vpv_standup vpv_said vpv_seekcontact vpv_wishes vpv_getalong vpv_interest vpv_talkconcerns vpv_adapt vpv_goodcontact vpv_learn
## 4 11 mspss_person_help mspss_person_joy mspss_family_help mspss_family_support mspss_person_comfort mspss_friends_help mspss_friends_count mspss_family_talk mspss_friends_joy mspss_person_feelings mspss_family_decide mspss_friends_talk vpv_meetfriends vpv_recogniseemotion vpv_class vpv_help vpv_easycontact vpv_hurt vpv_questions vpv_sensitivities vpv_standup vpv_said vpv_seekcontact vpv_wishes vpv_getalong vpv_interest vpv_talkconcerns vpv_adapt vpv_goodcontact vpv_learn
## 4 12 mspss_person_help mspss_person_joy mspss_family_help mspss_family_support mspss_person_comfort mspss_friends_help mspss_friends_count mspss_family_talk mspss_friends_joy mspss_person_feelings mspss_family_decide mspss_friends_talk vpv_meetfriends vpv_recogniseemotion vpv_class vpv_help vpv_easycontact vpv_hurt vpv_questions vpv_sensitivities vpv_standup vpv_said vpv_seekcontact vpv_wishes vpv_getalong vpv_interest vpv_talkconcerns vpv_adapt vpv_goodcontact vpv_learn
## 4 13 mspss_person_help mspss_person_joy mspss_family_help mspss_family_support mspss_person_comfort mspss_friends_help mspss_friends_count mspss_family_talk mspss_friends_joy mspss_person_feelings mspss_family_decide mspss_friends_talk vpv_meetfriends vpv_recogniseemotion vpv_class vpv_help vpv_easycontact vpv_hurt vpv_questions vpv_sensitivities vpv_standup vpv_said vpv_seekcontact vpv_wishes vpv_getalong vpv_interest vpv_talkconcerns vpv_adapt vpv_goodcontact vpv_learn
## 4 14 mspss_person_help mspss_person_joy mspss_family_help mspss_family_support mspss_person_comfort mspss_friends_help mspss_friends_count mspss_family_talk mspss_friends_joy mspss_person_feelings mspss_family_decide mspss_friends_talk vpv_meetfriends vpv_recogniseemotion vpv_class vpv_help vpv_easycontact vpv_hurt vpv_questions vpv_sensitivities vpv_standup vpv_said vpv_seekcontact vpv_wishes vpv_getalong vpv_interest vpv_talkconcerns vpv_adapt vpv_goodcontact vpv_learn
## 4 15 mspss_person_help mspss_person_joy mspss_family_help mspss_family_support mspss_person_comfort mspss_friends_help mspss_friends_count mspss_family_talk mspss_friends_joy mspss_person_feelings mspss_family_decide mspss_friends_talk vpv_meetfriends vpv_recogniseemotion vpv_class vpv_help vpv_easycontact vpv_hurt vpv_questions vpv_sensitivities vpv_standup vpv_said vpv_seekcontact vpv_wishes vpv_getalong vpv_interest vpv_talkconcerns vpv_adapt vpv_goodcontact vpv_learn
## 4 16 mspss_person_help mspss_person_joy mspss_family_help mspss_family_support mspss_person_comfort mspss_friends_help mspss_friends_count mspss_family_talk mspss_friends_joy mspss_person_feelings mspss_family_decide mspss_friends_talk vpv_meetfriends vpv_recogniseemotion vpv_class vpv_help vpv_easycontact vpv_hurt vpv_questions vpv_sensitivities vpv_standup vpv_said vpv_seekcontact vpv_wishes vpv_getalong vpv_interest vpv_talkconcerns vpv_adapt vpv_goodcontact vpv_learn
## 4 17 mspss_person_help mspss_person_joy mspss_family_help mspss_family_support mspss_person_comfort mspss_friends_help mspss_friends_count mspss_family_talk mspss_friends_joy mspss_person_feelings mspss_family_decide mspss_friends_talk vpv_meetfriends vpv_recogniseemotion vpv_class vpv_help vpv_easycontact vpv_hurt vpv_questions vpv_sensitivities vpv_standup vpv_said vpv_seekcontact vpv_wishes vpv_getalong vpv_interest vpv_talkconcerns vpv_adapt vpv_goodcontact vpv_learn
## 4 18 mspss_person_help mspss_person_joy mspss_family_help mspss_family_support mspss_person_comfort mspss_friends_help mspss_friends_count mspss_family_talk mspss_friends_joy mspss_person_feelings mspss_family_decide mspss_friends_talk vpv_meetfriends vpv_recogniseemotion vpv_class vpv_help vpv_easycontact vpv_hurt vpv_questions vpv_sensitivities vpv_standup vpv_said vpv_seekcontact vpv_wishes vpv_getalong vpv_interest vpv_talkconcerns vpv_adapt vpv_goodcontact vpv_learn
## 4 19 mspss_person_help mspss_person_joy mspss_family_help mspss_family_support mspss_person_comfort mspss_friends_help mspss_friends_count mspss_family_talk mspss_friends_joy mspss_person_feelings mspss_family_decide mspss_friends_talk vpv_meetfriends vpv_recogniseemotion vpv_class vpv_help vpv_easycontact vpv_hurt vpv_questions vpv_sensitivities vpv_standup vpv_said vpv_seekcontact vpv_wishes vpv_getalong vpv_interest vpv_talkconcerns vpv_adapt vpv_goodcontact vpv_learn
## 4 20 mspss_person_help mspss_person_joy mspss_family_help mspss_family_support mspss_person_comfort mspss_friends_help mspss_friends_count mspss_family_talk mspss_friends_joy mspss_person_feelings mspss_family_decide mspss_friends_talk vpv_meetfriends vpv_recogniseemotion vpv_class vpv_help vpv_easycontact vpv_hurt vpv_questions vpv_sensitivities vpv_standup vpv_said vpv_seekcontact vpv_wishes vpv_getalong vpv_interest vpv_talkconcerns vpv_adapt vpv_goodcontact vpv_learn
## 5 1 mspss_person_help mspss_person_joy mspss_family_help mspss_family_support mspss_person_comfort mspss_friends_help mspss_friends_count mspss_family_talk mspss_friends_joy mspss_person_feelings mspss_family_decide mspss_friends_talk vpv_meetfriends vpv_recogniseemotion vpv_class vpv_help vpv_easycontact vpv_hurt vpv_questions vpv_sensitivities vpv_standup vpv_said vpv_seekcontact vpv_wishes vpv_getalong vpv_interest vpv_talkconcerns vpv_adapt vpv_goodcontact vpv_learn
## 5 2 mspss_person_help mspss_person_joy mspss_family_help mspss_family_support mspss_person_comfort mspss_friends_help mspss_friends_count mspss_family_talk mspss_friends_joy mspss_person_feelings mspss_family_decide mspss_friends_talk vpv_meetfriends vpv_recogniseemotion vpv_class vpv_help vpv_easycontact vpv_hurt vpv_questions vpv_sensitivities vpv_standup vpv_said vpv_seekcontact vpv_wishes vpv_getalong vpv_interest vpv_talkconcerns vpv_adapt vpv_goodcontact vpv_learn
## 5 3 mspss_person_help mspss_person_joy mspss_family_help mspss_family_support mspss_person_comfort mspss_friends_help mspss_friends_count mspss_family_talk mspss_friends_joy mspss_person_feelings mspss_family_decide mspss_friends_talk vpv_meetfriends vpv_recogniseemotion vpv_class vpv_help vpv_easycontact vpv_hurt vpv_questions vpv_sensitivities vpv_standup vpv_said vpv_seekcontact vpv_wishes vpv_getalong vpv_interest vpv_talkconcerns vpv_adapt vpv_goodcontact vpv_learn
## 5 4 mspss_person_help mspss_person_joy mspss_family_help mspss_family_support mspss_person_comfort mspss_friends_help mspss_friends_count mspss_family_talk mspss_friends_joy mspss_person_feelings mspss_family_decide mspss_friends_talk vpv_meetfriends vpv_recogniseemotion vpv_class vpv_help vpv_easycontact vpv_hurt vpv_questions vpv_sensitivities vpv_standup vpv_said vpv_seekcontact vpv_wishes vpv_getalong vpv_interest vpv_talkconcerns vpv_adapt vpv_goodcontact vpv_learn
## 5 5 mspss_person_help mspss_person_joy mspss_family_help mspss_family_support mspss_person_comfort mspss_friends_help mspss_friends_count mspss_family_talk mspss_friends_joy mspss_person_feelings mspss_family_decide mspss_friends_talk vpv_meetfriends vpv_recogniseemotion vpv_class vpv_help vpv_easycontact vpv_hurt vpv_questions vpv_sensitivities vpv_standup vpv_said vpv_seekcontact vpv_wishes vpv_getalong vpv_interest vpv_talkconcerns vpv_adapt vpv_goodcontact vpv_learn
## 5 6 mspss_person_help mspss_person_joy mspss_family_help mspss_family_support mspss_person_comfort mspss_friends_help mspss_friends_count mspss_family_talk mspss_friends_joy mspss_person_feelings mspss_family_decide mspss_friends_talk vpv_meetfriends vpv_recogniseemotion vpv_class vpv_help vpv_easycontact vpv_hurt vpv_questions vpv_sensitivities vpv_standup vpv_said vpv_seekcontact vpv_wishes vpv_getalong vpv_interest vpv_talkconcerns vpv_adapt vpv_goodcontact vpv_learn
## 5 7 mspss_person_help mspss_person_joy mspss_family_help mspss_family_support mspss_person_comfort mspss_friends_help mspss_friends_count mspss_family_talk mspss_friends_joy mspss_person_feelings mspss_family_decide mspss_friends_talk vpv_meetfriends vpv_recogniseemotion vpv_class vpv_help vpv_easycontact vpv_hurt vpv_questions vpv_sensitivities vpv_standup vpv_said vpv_seekcontact vpv_wishes vpv_getalong vpv_interest vpv_talkconcerns vpv_adapt vpv_goodcontact vpv_learn
## 5 8 mspss_person_help mspss_person_joy mspss_family_help mspss_family_support mspss_person_comfort mspss_friends_help mspss_friends_count mspss_family_talk mspss_friends_joy mspss_person_feelings mspss_family_decide mspss_friends_talk vpv_meetfriends vpv_recogniseemotion vpv_class vpv_help vpv_easycontact vpv_hurt vpv_questions vpv_sensitivities vpv_standup vpv_said vpv_seekcontact vpv_wishes vpv_getalong vpv_interest vpv_talkconcerns vpv_adapt vpv_goodcontact vpv_learn
## 5 9 mspss_person_help mspss_person_joy mspss_family_help mspss_family_support mspss_person_comfort mspss_friends_help mspss_friends_count mspss_family_talk mspss_friends_joy mspss_person_feelings mspss_family_decide mspss_friends_talk vpv_meetfriends vpv_recogniseemotion vpv_class vpv_help vpv_easycontact vpv_hurt vpv_questions vpv_sensitivities vpv_standup vpv_said vpv_seekcontact vpv_wishes vpv_getalong vpv_interest vpv_talkconcerns vpv_adapt vpv_goodcontact vpv_learn
## 5 10 mspss_person_help mspss_person_joy mspss_family_help mspss_family_support mspss_person_comfort mspss_friends_help mspss_friends_count mspss_family_talk mspss_friends_joy mspss_person_feelings mspss_family_decide mspss_friends_talk vpv_meetfriends vpv_recogniseemotion vpv_class vpv_help vpv_easycontact vpv_hurt vpv_questions vpv_sensitivities vpv_standup vpv_said vpv_seekcontact vpv_wishes vpv_getalong vpv_interest vpv_talkconcerns vpv_adapt vpv_goodcontact vpv_learn
## 5 11 mspss_person_help mspss_person_joy mspss_family_help mspss_family_support mspss_person_comfort mspss_friends_help mspss_friends_count mspss_family_talk mspss_friends_joy mspss_person_feelings mspss_family_decide mspss_friends_talk vpv_meetfriends vpv_recogniseemotion vpv_class vpv_help vpv_easycontact vpv_hurt vpv_questions vpv_sensitivities vpv_standup vpv_said vpv_seekcontact vpv_wishes vpv_getalong vpv_interest vpv_talkconcerns vpv_adapt vpv_goodcontact vpv_learn
## 5 12 mspss_person_help mspss_person_joy mspss_family_help mspss_family_support mspss_person_comfort mspss_friends_help mspss_friends_count mspss_family_talk mspss_friends_joy mspss_person_feelings mspss_family_decide mspss_friends_talk vpv_meetfriends vpv_recogniseemotion vpv_class vpv_help vpv_easycontact vpv_hurt vpv_questions vpv_sensitivities vpv_standup vpv_said vpv_seekcontact vpv_wishes vpv_getalong vpv_interest vpv_talkconcerns vpv_adapt vpv_goodcontact vpv_learn
## 5 13 mspss_person_help mspss_person_joy mspss_family_help mspss_family_support mspss_person_comfort mspss_friends_help mspss_friends_count mspss_family_talk mspss_friends_joy mspss_person_feelings mspss_family_decide mspss_friends_talk vpv_meetfriends vpv_recogniseemotion vpv_class vpv_help vpv_easycontact vpv_hurt vpv_questions vpv_sensitivities vpv_standup vpv_said vpv_seekcontact vpv_wishes vpv_getalong vpv_interest vpv_talkconcerns vpv_adapt vpv_goodcontact vpv_learn
## 5 14 mspss_person_help mspss_person_joy mspss_family_help mspss_family_support mspss_person_comfort mspss_friends_help mspss_friends_count mspss_family_talk mspss_friends_joy mspss_person_feelings mspss_family_decide mspss_friends_talk vpv_meetfriends vpv_recogniseemotion vpv_class vpv_help vpv_easycontact vpv_hurt vpv_questions vpv_sensitivities vpv_standup vpv_said vpv_seekcontact vpv_wishes vpv_getalong vpv_interest vpv_talkconcerns vpv_adapt vpv_goodcontact vpv_learn
## 5 15 mspss_person_help mspss_person_joy mspss_family_help mspss_family_support mspss_person_comfort mspss_friends_help mspss_friends_count mspss_family_talk mspss_friends_joy mspss_person_feelings mspss_family_decide mspss_friends_talk vpv_meetfriends vpv_recogniseemotion vpv_class vpv_help vpv_easycontact vpv_hurt vpv_questions vpv_sensitivities vpv_standup vpv_said vpv_seekcontact vpv_wishes vpv_getalong vpv_interest vpv_talkconcerns vpv_adapt vpv_goodcontact vpv_learn
## 5 16 mspss_person_help mspss_person_joy mspss_family_help mspss_family_support mspss_person_comfort mspss_friends_help mspss_friends_count mspss_family_talk mspss_friends_joy mspss_person_feelings mspss_family_decide mspss_friends_talk vpv_meetfriends vpv_recogniseemotion vpv_class vpv_help vpv_easycontact vpv_hurt vpv_questions vpv_sensitivities vpv_standup vpv_said vpv_seekcontact vpv_wishes vpv_getalong vpv_interest vpv_talkconcerns vpv_adapt vpv_goodcontact vpv_learn
## 5 17 mspss_person_help mspss_person_joy mspss_family_help mspss_family_support mspss_person_comfort mspss_friends_help mspss_friends_count mspss_family_talk mspss_friends_joy mspss_person_feelings mspss_family_decide mspss_friends_talk vpv_meetfriends vpv_recogniseemotion vpv_class vpv_help vpv_easycontact vpv_hurt vpv_questions vpv_sensitivities vpv_standup vpv_said vpv_seekcontact vpv_wishes vpv_getalong vpv_interest vpv_talkconcerns vpv_adapt vpv_goodcontact vpv_learn
## 5 18 mspss_person_help mspss_person_joy mspss_family_help mspss_family_support mspss_person_comfort mspss_friends_help mspss_friends_count mspss_family_talk mspss_friends_joy mspss_person_feelings mspss_family_decide mspss_friends_talk vpv_meetfriends vpv_recogniseemotion vpv_class vpv_help vpv_easycontact vpv_hurt vpv_questions vpv_sensitivities vpv_standup vpv_said vpv_seekcontact vpv_wishes vpv_getalong vpv_interest vpv_talkconcerns vpv_adapt vpv_goodcontact vpv_learn
## 5 19 mspss_person_help mspss_person_joy mspss_family_help mspss_family_support mspss_person_comfort mspss_friends_help mspss_friends_count mspss_family_talk mspss_friends_joy mspss_person_feelings mspss_family_decide mspss_friends_talk vpv_meetfriends vpv_recogniseemotion vpv_class vpv_help vpv_easycontact vpv_hurt vpv_questions vpv_sensitivities vpv_standup vpv_said vpv_seekcontact vpv_wishes vpv_getalong vpv_interest vpv_talkconcerns vpv_adapt vpv_goodcontact vpv_learn
## 5 20 mspss_person_help mspss_person_joy mspss_family_help mspss_family_support mspss_person_comfort mspss_friends_help mspss_friends_count mspss_family_talk mspss_friends_joy mspss_person_feelings mspss_family_decide mspss_friends_talk vpv_meetfriends vpv_recogniseemotion vpv_class vpv_help vpv_easycontact vpv_hurt vpv_questions vpv_sensitivities vpv_standup vpv_said vpv_seekcontact vpv_wishes vpv_getalong vpv_interest vpv_talkconcerns vpv_adapt vpv_goodcontact vpv_learn

##INCLUDING THE IMPUTED VARIABLES IN THE PL AND ESM DATASETS##

dat_esm = list()
PLdat_complete = list()
imp.data.Wave.I = list()
imp.data.Wave.II = list()

for (i in 1:20) {
 #Computing the sum score of the SSL
 imp.data.Wave.I[[i]] = complete(data.imp.Wave.I,i)
 imp.data.Wave.I[[i]]$SSL = rowSums(imp.data.Wave.I[[i]][,c("ssl_invite", "ssl_visit","ssl_affection", "ssl_comfort", "ssl_compliment", "ssl_interest",
 "ssl_offerhelp", "ssl_ease", "ssl_advice", "ssl_confidence", "ssl_askhelp",
 "ssl_positivecharacter")])

 #Computing the sum score of the VPV T0
 imp.data.Wave.I[[i]]$VPV = rowSums(imp.data.Wave.I[[i]][,c("vpv_meetfriends", "vpv_recogniseemotion", "vpv_class", "vpv_help", "vpv_easycontact",
 "vpv_hurt", "vpv_questions", "vpv_sensitivities", "vpv_standup", "vpv_said", "vpv_seekcontact",
 "vpv_wishes", "vpv_getalong", "vpv_interest", "vpv_talkconcerns", "vpv_adapt",
 "vpv_goodcontact", "vpv_learn")])

 #Computing the sum score of the VPV T2
 imp.data.Wave.II[[i]] = complete(data.imp.Wave.II,i)
 imp.data.Wave.II[[i]]$VPV = rowSums(imp.data.Wave.II[[i]][,c("vpv_meetfriends", "vpv_recogniseemotion", "vpv_class", "vpv_help", "vpv_easycontact",
 "vpv_hurt", "vpv_questions", "vpv_sensitivities", "vpv_standup", "vpv_said", "vpv_seekcontact",
 "vpv_wishes", "vpv_getalong", "vpv_interest", "vpv_talkconcerns", "vpv_adapt",
 "vpv_goodcontact", "vpv_learn")])

 #Computing the sum score of the MSPSS
 imp.data.Wave.II[[i]]$MSPSS = rowSums(imp.data.Wave.II[[i]][,c("mspss_person_help", "mspss_person_joy", "mspss_family_help", "mspss_family_support",
 "mspss_person_comfort", "mspss_friends_help", "mspss_friends_count",
 "mspss_family_talk", "mspss_friends_joy", "mspss_person_feelings",
 "mspss_family_decide", "mspss_friends_talk")])


 n.ID = unique(dat$record_id)

 #Adding variables to the PL dataset
 PLdat_complete[[i]] = PLdat
 PLdat_complete[[i]]$SSL = rep(NA,nrow(PLdat))
 PLdat_complete[[i]]$VPV = rep(NA,nrow(PLdat))
 PLdat_complete[[i]]$MSPSS = rep(NA,nrow(PLdat))

 for (j in n.ID) {

 #SSL and VPV T0
 if (length(which(PLdat$record_id==j & PLdat$wave == 'wave1'))>0){

 T.i = which(PLdat$record_id==j)
 n.ID.Wave.I = which(imp.data.Wave.I[[i]][,"record_id"]==j)

 PLdat_complete[[i]]$SSL[T.i] = imp.data.Wave.I[[i]]$SSL[n.ID.Wave.I]
 PLdat_complete[[i]]$VPV[T.i] = imp.data.Wave.I[[i]]$VPV[n.ID.Wave.I]
 }

 #MSPSS and VPV T2
 T.i.waveII = which(PLdat$record_id==j & PLdat$wave == 'wave2')
 n.ID.Wave.II = which(imp.data.Wave.II[[i]][,"record_id"]==j)

 PLdat_complete[[i]]$MSPSS[T.i.waveII] = imp.data.Wave.II[[i]]$MSPSS[n.ID.Wave.II]
 PLdat_complete[[i]]$VPV[T.i.waveII] = imp.data.Wave.II[[i]]$VPV[n.ID.Wave.II]
 }

 #Adding variables to the ESM dataset
 dat_esm[[i]] = dat
 dat_esm[[i]]$SSL = rep(NA,nrow(dat))
 dat_esm[[i]]$VPV = rep(NA,nrow(dat))
 dat_esm[[i]]$MSPSS = rep(NA,nrow(dat))

 for (j in n.ID) {

 #SSL and VPV
 if (length(which(dat$record_id==j & dat$wave == 'wave1'))>0){

 T.i = which(dat$record_id==j)
 n.ID.Wave.I = which(imp.data.Wave.I[[i]][,"record_id"]==j)

 dat_esm[[i]]$SSL[T.i] = imp.data.Wave.I[[i]][n.ID.Wave.I,"SSL"]
 dat_esm[[i]]$VPV[T.i] = imp.data.Wave.I[[i]][n.ID.Wave.I,"VPV"]
 }

 #MSPSS
 T.i.waveII = which(dat$record_id==j & dat$wave == 'wave2')
 n.ID.Wave.II = which(imp.data.Wave.II[[i]][,"record_id"]==j)

 dat_esm[[i]]$MSPSS[T.i.waveII] = imp.data.Wave.II[[i]][n.ID.Wave.II,"MSPSS"]
 dat_esm[[i]]$VPV[T.i.waveII] = imp.data.Wave.II[[i]][n.ID.Wave.II,"VPV"]
 }}

######################################################################################################
##RQ4 PART 2: MODERATORS IN CHANGES FROM T0 TO T2 (IMPUTED MODERATORS)##

#Alpha per moderator = .007 (Bonferroni corrected, initial alpha of .05)

PLcovidDat = list()
ESMcovidDat = list()

for (i in 1:20){
 PLcovidDat[[i]] = PLdat_complete[[i]][PLdat_complete[[i]]$wave == 'wave2',]

 ESMcovidDat[[i]] = dat_esm[[i]][dat_esm[[i]]$wave == 'wave2',]
}

PLcovidDat.list <- as.mitml.list(PLcovidDat)
ESMcovidDat.list <- as.mitml.list(ESMcovidDat)
PLdat.list <- as.mitml.list(PLdat_complete)
dat.list <- as.mitml.list(dat_esm)

for (i in 1:20){
 PLdat.list[[i]]$wave.f = as.factor(PLdat.list[[i]]$wave.f)
 PLdat.list[[i]]$sex = as.factor(PLdat.list[[i]]$sex)
 dat.list[[i]]$wave.f = as.factor(dat.list[[i]]$wave.f)
 dat.list[[i]]$sex = as.factor(dat.list[[i]]$sex_ismale)
 PLcovidDat.list[[i]]$wave.f = as.factor(PLcovidDat.list[[i]]$wave.f)
 PLcovidDat.list[[i]]$sex = as.factor(PLcovidDat.list[[i]]$sex)
 ESMcovidDat.list[[i]]$wave.f = as.factor(ESMcovidDat.list[[i]]$wave.f)
 ESMcovidDat.list[[i]]$sex = as.factor(ESMcovidDat.list[[i]]$sex_ismale)
}

#SSL
H4a_ssl <- with(PLdat.list, lme(PL_aloneprop ~ wave.f + SSL + wave.f*SSL + c_age + sex,
 random = ~1| record_id, na.action = na.omit))
testEstimates(H4a_ssl)

##
## Call:
##
## testEstimates(model = H4a_ssl)
##
## Final parameter estimates and inferences obtained from 20 imputed data sets.
##
## Estimate Std.Error t.value df P(>|t|) RIV FMI
## (Intercept) 0.250 0.054 4.615 7.065e+04 0.000 0.017 0.016
## wave.f2 0.266 0.074 3.600 1.577e+04 0.000 0.036 0.035
## SSL -0.002 0.002 -0.886 5.740e+04 0.376 0.019 0.018
## c_age 0.020 0.005 3.783 3.501e+07 0.000 0.001 0.001
## sex2 -0.006 0.023 -0.273 3.473e+08 0.785 0.000 0.000
## wave.f2:SSL -0.001 0.003 -0.404 1.467e+04 0.686 0.037 0.036
##
## Unadjusted hypothesis test as appropriate in larger samples.

H4b_ssl <- with(dat.list, lme(momentPA ~ wave.f + SSL + wave.f*SSL + c_age + sex,
 random = ~1| record_id, method = "REML", na.action = na.omit, control = lmeControl(opt='optim')))
testEstimates(H4b_ssl)

##
## Call:
##
## testEstimates(model = H4b_ssl)
##
## Final parameter estimates and inferences obtained from 20 imputed data sets.
##
## Estimate Std.Error t.value df P(>|t|) RIV FMI
## (Intercept) 3.493 0.283 12.322 3.726e+04 0.000 0.023 0.023
## wave.fwave2 0.913 0.243 3.755 5.805e+02 0.000 0.221 0.184
## SSL 0.064 0.012 5.367 3.895e+04 0.000 0.023 0.022
## c_age -0.102 0.030 -3.454 3.217e+06 0.001 0.002 0.002
## sex1 0.532 0.135 3.940 3.302e+06 0.000 0.002 0.002
## wave.fwave2:SSL -0.050 0.010 -5.058 4.360e+02 0.000 0.264 0.212
##
## Unadjusted hypothesis test as appropriate in larger samples.

H4c_ssl <- with(dat.list, lme(momentNA ~ wave.f + SSL + wave.f*SSL + c_age + sex,
 random = ~1| record_id, method = "REML", na.action = na.omit, control = lmeControl(opt='optim')))
testEstimates(H4c_ssl)

##
## Call:
##
## testEstimates(model = H4c_ssl)
##
## Final parameter estimates and inferences obtained from 20 imputed data sets.
##
## Estimate Std.Error t.value df P(>|t|) RIV FMI
## (Intercept) 2.581 0.270 9.570 2.928e+04 0.000 0.026 0.026
## wave.fwave2 -0.029 0.203 -0.141 2.936e+02 0.888 0.341 0.259
## SSL -0.021 0.011 -1.821 2.590e+04 0.069 0.028 0.027
## c_age 0.071 0.028 2.515 2.328e+07 0.012 0.001 0.001
## sex1 -0.359 0.128 -2.809 9.657e+07 0.005 0.000 0.000
## wave.fwave2:SSL 0.005 0.008 0.562 2.080e+02 0.575 0.433 0.309
##
## Unadjusted hypothesis test as appropriate in larger samples.

H4d_ssl <- with(dat.list, lme(esm_soc_alone_pleasant ~ wave.f + SSL + wave.f*SSL + c_age + sex,
 random = ~1| record_id, method = "REML", na.action = na.omit, control = lmeControl(opt='optim')))
testEstimates(H4d_ssl)

##
## Call:
##
## testEstimates(model = H4d_ssl)
##
## Final parameter estimates and inferences obtained from 20 imputed data sets.
##
## Estimate Std.Error t.value df P(>|t|) RIV FMI
## (Intercept) 5.679 0.444 12.796 6.721e+03 0.000 0.056 0.053
## wave.fwave2 -1.088 0.349 -3.113 2.394e+02 0.002 0.392 0.288
## SSL -0.012 0.019 -0.640 5.693e+03 0.522 0.061 0.058
## c_age -0.041 0.046 -0.884 4.313e+06 0.377 0.002 0.002
## sex1 -0.432 0.208 -2.075 8.242e+08 0.038 0.000 0.000
## wave.fwave2:SSL 0.033 0.014 2.356 1.765e+02 0.020 0.488 0.336
##
## Unadjusted hypothesis test as appropriate in larger samples.

H4e_ssl <- with(dat.list, lme(esm_soc_alone_want ~ wave.f + SSL + wave.f*SSL + c_age + sex,
 random = ~1| record_id, method = "REML", na.action = na.omit, control = lmeControl(opt='optim')))
testEstimates(H4e_ssl)

##
## Call:
##
## testEstimates(model = H4e_ssl)
##
## Final parameter estimates and inferences obtained from 20 imputed data sets.
##
## Estimate Std.Error t.value df P(>|t|) RIV FMI
## (Intercept) 5.700 0.463 12.321 8.156e+03 0.000 0.051 0.048
## wave.fwave2 -1.200 0.371 -3.232 4.466e+02 0.001 0.260 0.210
## SSL -0.037 0.019 -1.891 6.750e+03 0.059 0.056 0.053
## c_age -0.052 0.048 -1.082 4.405e+06 0.279 0.002 0.002
## sex1 -0.532 0.218 -2.442 2.594e+09 0.015 0.000 0.000
## wave.fwave2:SSL 0.041 0.015 2.770 3.285e+02 0.006 0.317 0.245
##
## Unadjusted hypothesis test as appropriate in larger samples.

H4f_ssl <- with(dat.list, lme(esm_soc_alone_excluded ~ wave.f + SSL + wave.f*SSL + c_age + sex,
 random = ~1| record_id, method = "REML", na.action = na.omit, control = lmeControl(opt='optim')))
testEstimates(H4f_ssl)

##
## Call:
##
## testEstimates(model = H4f_ssl)
##
## Final parameter estimates and inferences obtained from 20 imputed data sets.
##
## Estimate Std.Error t.value df P(>|t|) RIV FMI
## (Intercept) 1.841 0.191 9.623 8.625e+04 0.000 0.015 0.015
## wave.fwave2 0.129 0.171 0.753 3.835e+04 0.452 0.023 0.022
## SSL -0.021 0.008 -2.583 8.144e+04 0.010 0.016 0.015
## c_age 0.016 0.020 0.812 1.890e+07 0.417 0.001 0.001
## sex1 -0.121 0.089 -1.351 1.106e+07 0.177 0.001 0.001
## wave.fwave2:SSL -0.003 0.007 -0.395 3.263e+04 0.692 0.025 0.024
##
## Unadjusted hypothesis test as appropriate in larger samples.

H4g_ssl <- with(dat.list, lme(esm_mood_lonely ~ wave.f + SSL + wave.f*SSL + c_age + sex,
 random = ~1| record_id, method = "REML", na.action = na.omit, control = lmeControl(opt='optim')))
testEstimates(H4g_ssl)

##
## Call:
##
## testEstimates(model = H4g_ssl)
##
## Final parameter estimates and inferences obtained from 20 imputed data sets.
##
## Estimate Std.Error t.value df P(>|t|) RIV FMI
[truncated: 19,163 more chars]
